# Supplementary material for: Apple CRISPR-Cas9—A Recipe for Successful Targeting of AGAMOUS-like Genes in Domestic Apple
Source: Plants (Basel). 2023 Oct 26;12(21):3693. doi: 10.3390/plants12213693 (PMC10649517; doi:10.3390/plants12213693)
Supplement: Supplementary file 1 [file plants-12-03693-s001.zip › apple supplemental figures v3.pptx]

## Slide 1
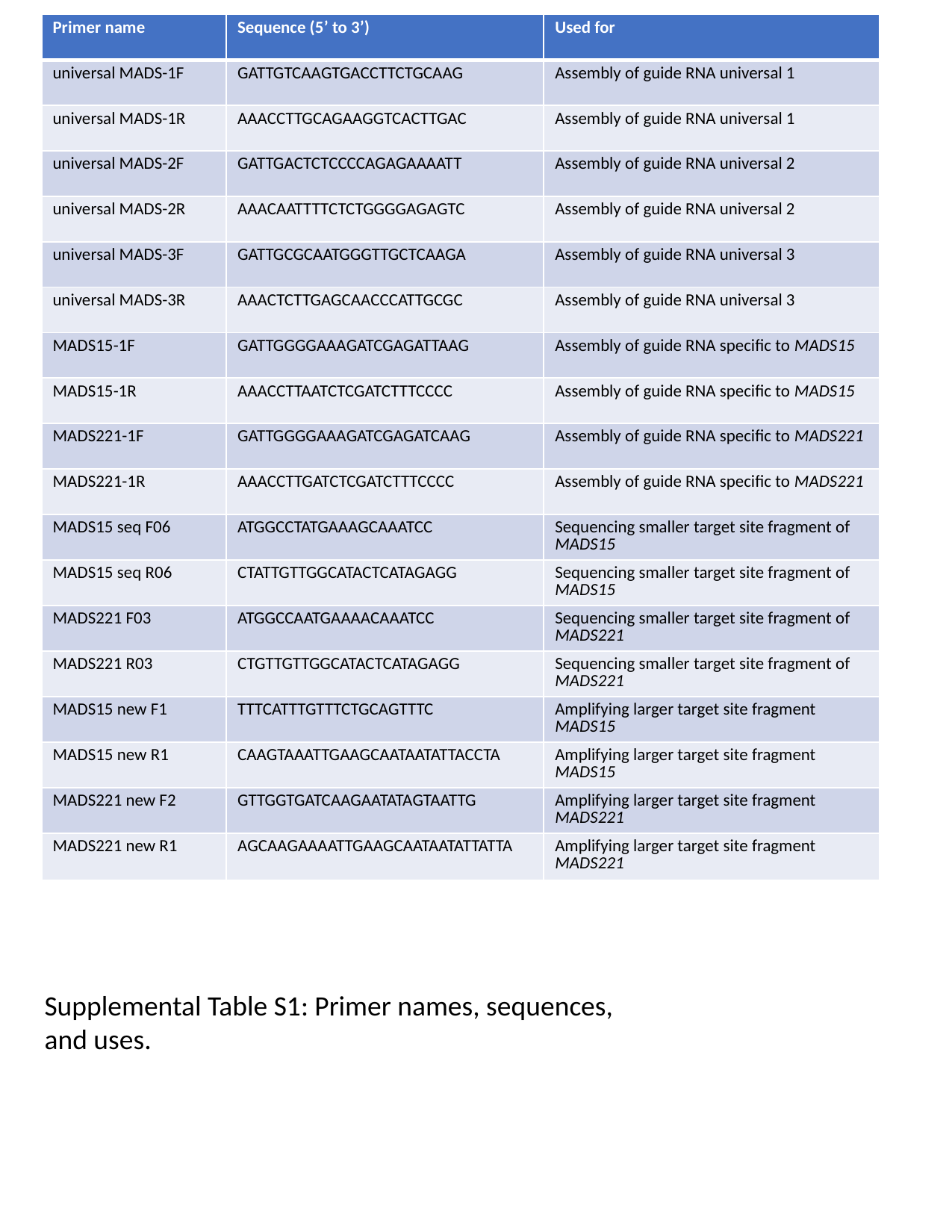

| Primer name | Sequence (5’ to 3’) | Used for |
| --- | --- | --- |
| universal MADS-1F | GATTGTCAAGTGACCTTCTGCAAG | Assembly of guide RNA universal 1 |
| universal MADS-1R | AAACCTTGCAGAAGGTCACTTGAC | Assembly of guide RNA universal 1 |
| universal MADS-2F | GATTGACTCTCCCCAGAGAAAATT | Assembly of guide RNA universal 2 |
| universal MADS-2R | AAACAATTTTCTCTGGGGAGAGTC | Assembly of guide RNA universal 2 |
| universal MADS-3F | GATTGCGCAATGGGTTGCTCAAGA | Assembly of guide RNA universal 3 |
| universal MADS-3R | AAACTCTTGAGCAACCCATTGCGC | Assembly of guide RNA universal 3 |
| MADS15-1F | GATTGGGGAAAGATCGAGATTAAG | Assembly of guide RNA specific to MADS15 |
| MADS15-1R | AAACCTTAATCTCGATCTTTCCCC | Assembly of guide RNA specific to MADS15 |
| MADS221-1F | GATTGGGGAAAGATCGAGATCAAG | Assembly of guide RNA specific to MADS221 |
| MADS221-1R | AAACCTTGATCTCGATCTTTCCCC | Assembly of guide RNA specific to MADS221 |
| MADS15 seq F06 | ATGGCCTATGAAAGCAAATCC | Sequencing smaller target site fragment of MADS15 |
| MADS15 seq R06 | CTATTGTTGGCATACTCATAGAGG | Sequencing smaller target site fragment of MADS15 |
| MADS221 F03 | ATGGCCAATGAAAACAAATCC | Sequencing smaller target site fragment of MADS221 |
| MADS221 R03 | CTGTTGTTGGCATACTCATAGAGG | Sequencing smaller target site fragment of MADS221 |
| MADS15 new F1 | TTTCATTTGTTTCTGCAGTTTC | Amplifying larger target site fragment MADS15 |
| MADS15 new R1 | CAAGTAAATTGAAGCAATAATATTACCTA | Amplifying larger target site fragment MADS15 |
| MADS221 new F2 | GTTGGTGATCAAGAATATAGTAATTG | Amplifying larger target site fragment MADS221 |
| MADS221 new R1 | AGCAAGAAAATTGAAGCAATAATATTATTA | Amplifying larger target site fragment MADS221 |
Supplemental Table S1: Primer names, sequences, and uses.

## Slide 2
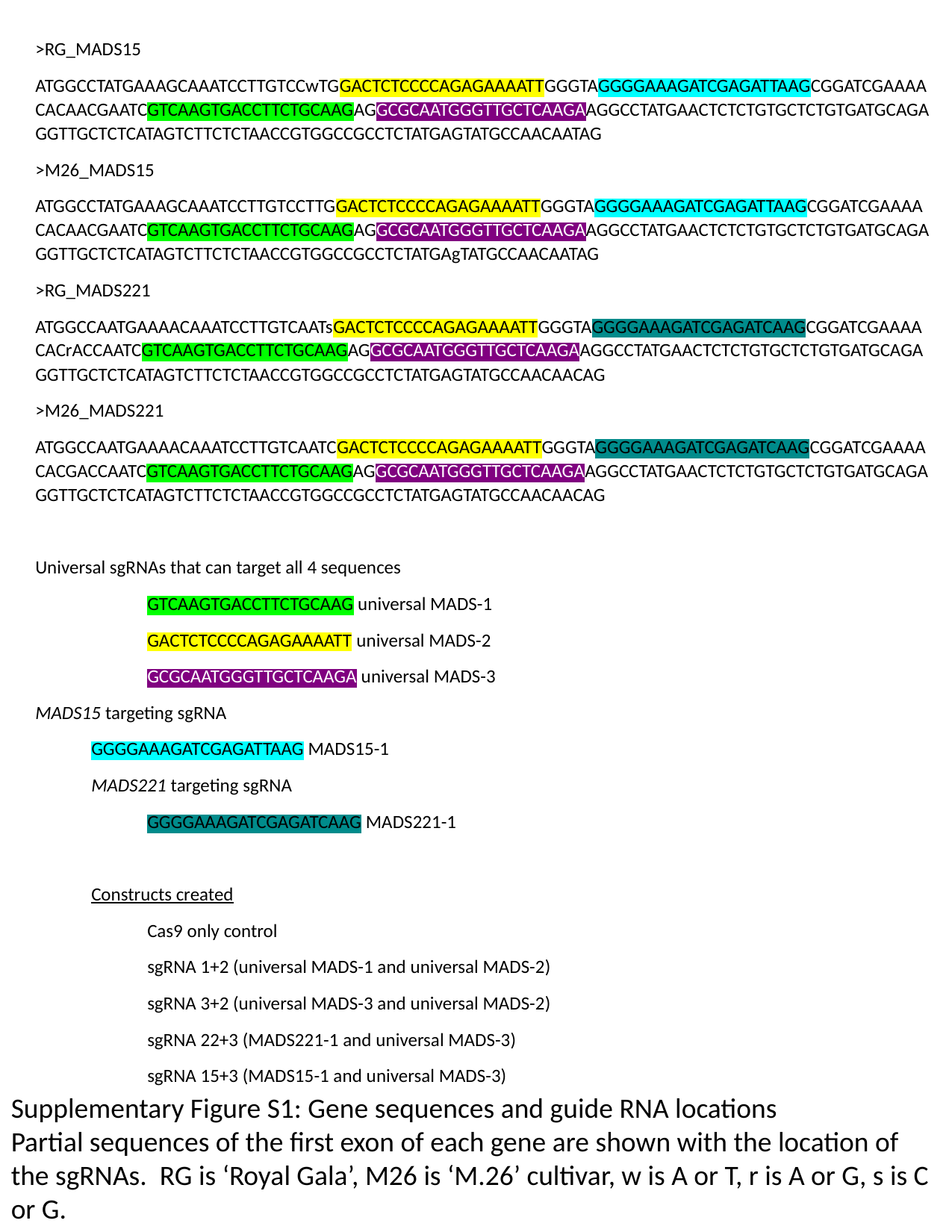

>RG_MADS15
ATGGCCTATGAAAGCAAATCCTTGTCCwTGGACTCTCCCCAGAGAAAATTGGGTAGGGGAAAGATCGAGATTAAGCGGATCGAAAACACAACGAATCGTCAAGTGACCTTCTGCAAGAGGCGCAATGGGTTGCTCAAGAAGGCCTATGAACTCTCTGTGCTCTGTGATGCAGAGGTTGCTCTCATAGTCTTCTCTAACCGTGGCCGCCTCTATGAGTATGCCAACAATAG
>M26_MADS15
ATGGCCTATGAAAGCAAATCCTTGTCCTTGGACTCTCCCCAGAGAAAATTGGGTAGGGGAAAGATCGAGATTAAGCGGATCGAAAACACAACGAATCGTCAAGTGACCTTCTGCAAGAGGCGCAATGGGTTGCTCAAGAAGGCCTATGAACTCTCTGTGCTCTGTGATGCAGAGGTTGCTCTCATAGTCTTCTCTAACCGTGGCCGCCTCTATGAgTATGCCAACAATAG
>RG_MADS221
ATGGCCAATGAAAACAAATCCTTGTCAATsGACTCTCCCCAGAGAAAATTGGGTAGGGGAAAGATCGAGATCAAGCGGATCGAAAACACrACCAATCGTCAAGTGACCTTCTGCAAGAGGCGCAATGGGTTGCTCAAGAAGGCCTATGAACTCTCTGTGCTCTGTGATGCAGAGGTTGCTCTCATAGTCTTCTCTAACCGTGGCCGCCTCTATGAGTATGCCAACAACAG
>M26_MADS221
ATGGCCAATGAAAACAAATCCTTGTCAATCGACTCTCCCCAGAGAAAATTGGGTAGGGGAAAGATCGAGATCAAGCGGATCGAAAACACGACCAATCGTCAAGTGACCTTCTGCAAGAGGCGCAATGGGTTGCTCAAGAAGGCCTATGAACTCTCTGTGCTCTGTGATGCAGAGGTTGCTCTCATAGTCTTCTCTAACCGTGGCCGCCTCTATGAGTATGCCAACAACAG
Universal sgRNAs that can target all 4 sequences
	GTCAAGTGACCTTCTGCAAG universal MADS-1
	GACTCTCCCCAGAGAAAATT universal MADS-2
	GCGCAATGGGTTGCTCAAGA universal MADS-3
MADS15 targeting sgRNA
GGGGAAAGATCGAGATTAAG MADS15-1
MADS221 targeting sgRNA
	GGGGAAAGATCGAGATCAAG MADS221-1
Constructs created
	Cas9 only control
	sgRNA 1+2 (universal MADS-1 and universal MADS-2)
	sgRNA 3+2 (universal MADS-3 and universal MADS-2)
	sgRNA 22+3 (MADS221-1 and universal MADS-3)
	sgRNA 15+3 (MADS15-1 and universal MADS-3)
Supplementary Figure S1: Gene sequences and guide RNA locations
Partial sequences of the first exon of each gene are shown with the location of the sgRNAs. RG is ‘Royal Gala’, M26 is ‘M.26’ cultivar, w is A or T, r is A or G, s is C or G.

## Slide 3
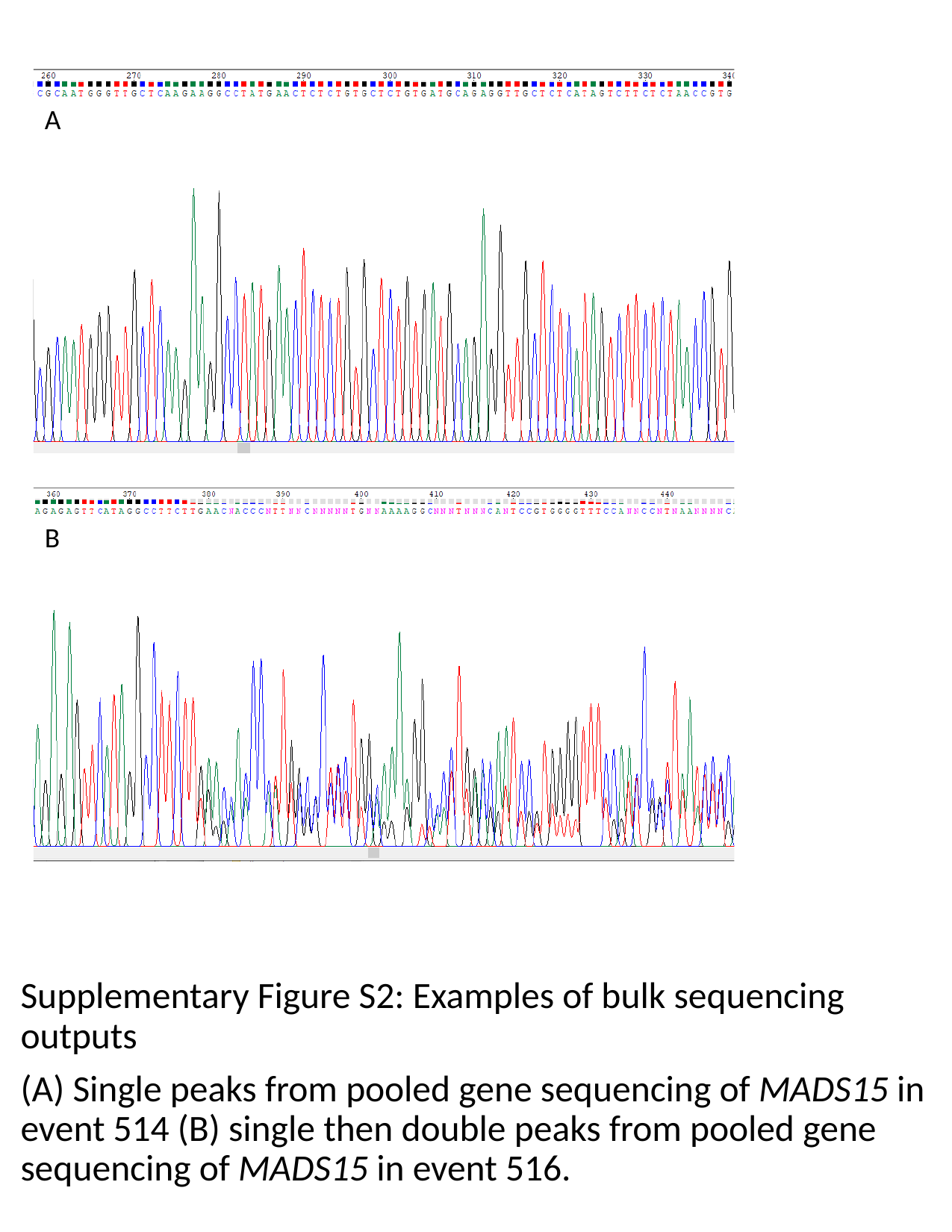

A
B
Supplementary Figure S2: Examples of bulk sequencing outputs
(A) Single peaks from pooled gene sequencing of MADS15 in event 514 (B) single then double peaks from pooled gene sequencing of MADS15 in event 516.

## Slide 4
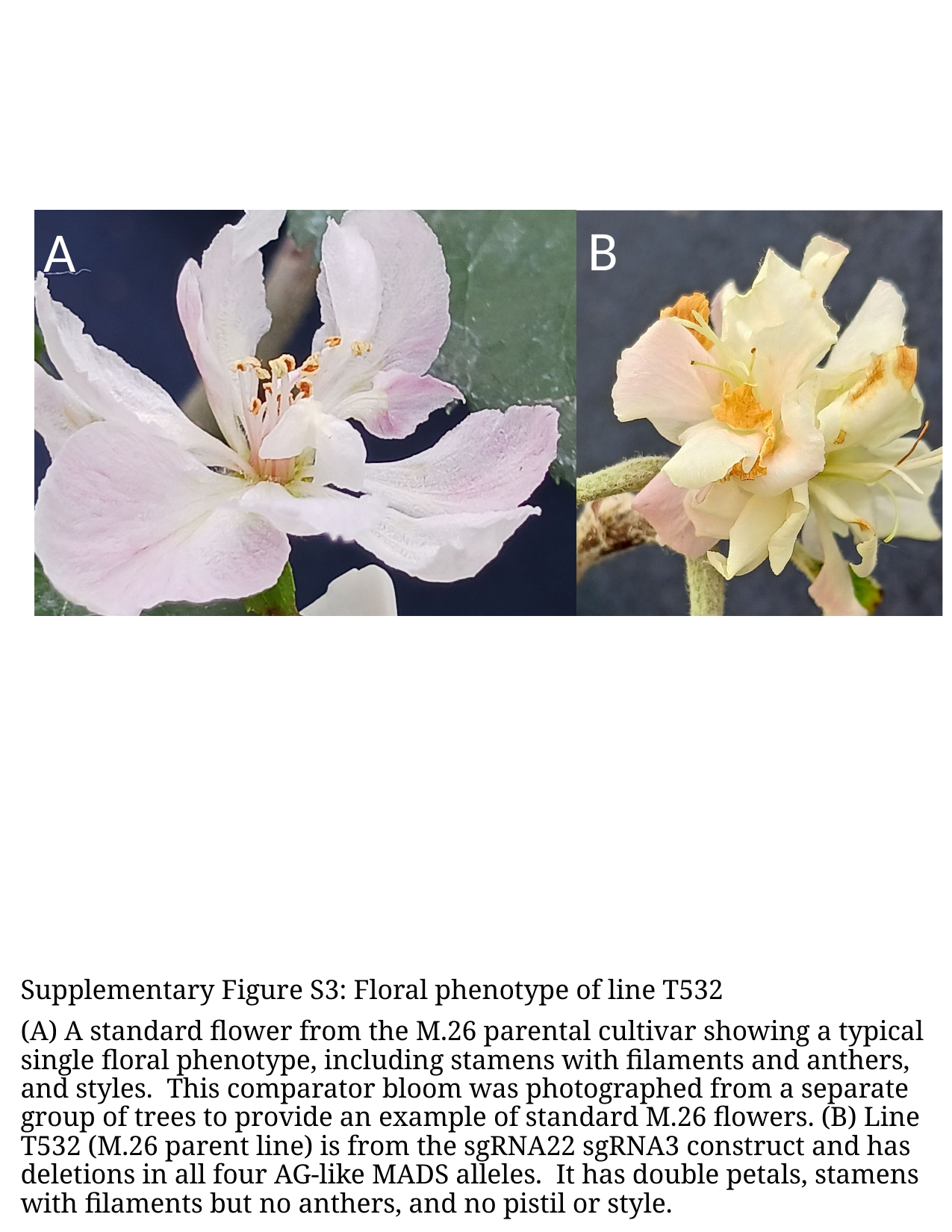

B
A
Supplementary Figure S3: Floral phenotype of line T532
(A) A standard flower from the M.26 parental cultivar showing a typical single floral phenotype, including stamens with filaments and anthers, and styles. This comparator bloom was photographed from a separate group of trees to provide an example of standard M.26 flowers. (B) Line T532 (M.26 parent line) is from the sgRNA22 sgRNA3 construct and has deletions in all four AG-like MADS alleles. It has double petals, stamens with filaments but no anthers, and no pistil or style.

## Slide 5
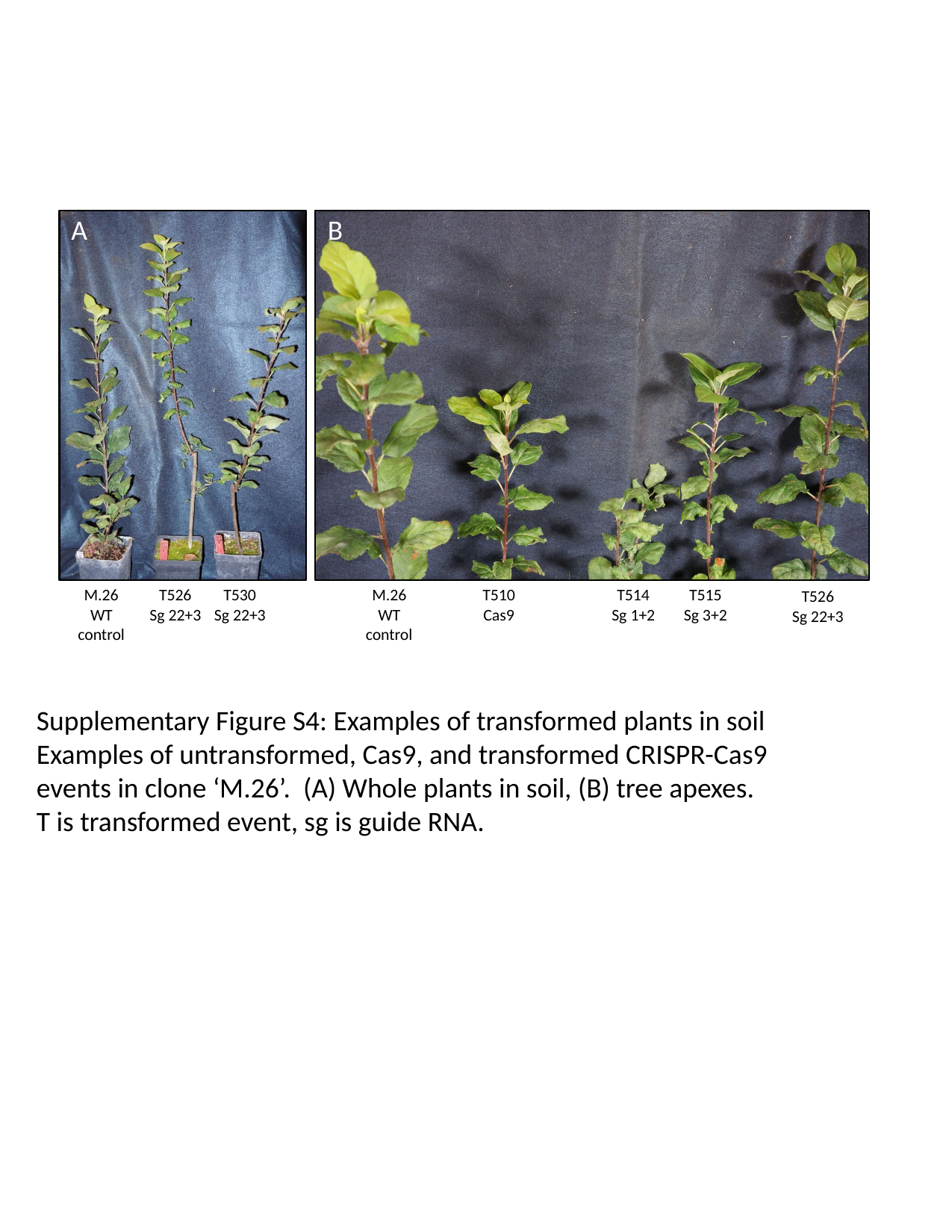

A
B
M.26
WT control
T526
Sg 22+3
T530
Sg 22+3
M.26
WT control
T510
Cas9
T514
Sg 1+2
T515
Sg 3+2
T526
Sg 22+3
Supplementary Figure S4: Examples of transformed plants in soil
Examples of untransformed, Cas9, and transformed CRISPR-Cas9 events in clone ‘M.26’. (A) Whole plants in soil, (B) tree apexes. T is transformed event, sg is guide RNA.
