# Supplementary material for: Apple CRISPR-Cas9—A Recipe for Successful Targeting of AGAMOUS-like Genes in Domestic Apple
Source: Plants (Basel). 2023 Oct 26;12(21):3693. doi: 10.3390/plants12213693 (PMC10649517; doi:10.3390/plants12213693)
Supplement: Supplementary file 1 [file plants-12-03693-s001.zip › supplemental File 1 all allele sequences v1.pdf]

Sequences for alleles from all events

Sequence data in FASTA format, data are in original format as downloaded from Azenta, gene fragments were cloned into pTOPO and sequenced with the M13F primer (5' – GTTTTCCCAGTCACGAC – 3').

WT sequences – raw reads

First exon is underlined

>RG_MADS15
NNNNNNNNGNANNNGGGGatNGtTATCTTAATTGTTACTTAATTATAGTTTAAGCTCCCTAGTTGTTGTAGTTGAA
TTGCACCAAGTTTTCATAATTTCATTTGTTTCTGCAAGTTTCAAACTTTTTGTTGGTGATCTrGAATATAGTAATTAAT
TAAAGTTCTGGGGTTTTTATTTTAATTAGAAAGGTGCTTAATTGATTGATCTCTTTGTTCTTATTGATTCAGyTTCCA
ACTATGGCCTATGAAAGCAAATCCTTGTCCwTGGACTCTCCCCAGAGAAAATTGGGTAGGGGAAAGATCGAGATT
AAGCGGATCGAAAACACAACGAATCGTCAAGTGACCTTCTGCAAGAGGCGCAATGGGTTGCTCAAGAAGGCCTAT
GAACTCTCTGTGCTCTGTGATGCAGAGGTTGCTCTCATAGTCTTCTCTAACCGTGGCCGCCTCTATGAGTATGCCAA
CAATAGGTAATATTATTGCTTCAATTTACTTGCTAAATTTTCCTCTTCTTGTTCTTCGTTTAATTTTTTAATGAAArAAT
TTTAGAAAATATTTCAGACCCAAGTTTTGTTCATTTTAAATTTACCTTTTAATACAAGTTCTTTGTTCTTGTTAAAGTT
CTTTTCTTTTTCTTTCTTCTTTTGTGCAAATTTTGGTCACAAATTTAGCATTTCAAAAAGTTATTGGTGTTTCAGTCCTC
AAGATTTTTTGCTGTGAGATAGATCTTTTAAGTA

>RG_MADS221
TGGGATTGCTAGNNNTGGNANTGCNAGCTTAATTAGCTAATCAATTATTTTTTAAGCTACCTAGTTGTTGTAGTTG
AATTGCTTTCATTTGTTTCTGCAAGTTAATTTCAAACTTTTTGTTGGTGATCAAGAATATAGTAATTGAAGTTCTGGG
GTTTTTGTTTTATTTAAAAAGGTGCTTAATTGATTGATCTCTTTGTTCTTATTGATTCAGCTTGCAACTATGGCCAAT
GAAAACAAATCCTTGTCAATCGACTCTCCCCAGAGAAAATTGGGTAGGGGAAAGATCGAGATCAAGCGGATCGAA
AACACGACCAATCGTCAAGTGACCTTCTGCAAGAGGCGCAATGGGTTGCTCAAGAAGGCCTATGAACTCTCTGTG
CTCTGTGATGCAGAGGTTGCTCTCATAGTCTTCTCTAACCGTGGCCGCCTCTATGAGTATGCCAACAACAGGTAATA
ATATTATTGCTTCAATTTTCTTGCTTAATTTTCTCTTCTTATTCTTTTATTTTTTCTAATGGAAGGCTTTTAGTATTATTT
TCAGACCCAAGTTTTATTTTCAATTTACCTTTTAATAAGATTTCTTTGTTCTTGTTAAAGTTCTTTTCTTTTTCTTTCTTC
TTCTGTGCAAANTTGTCACAATNANCNTNCNANNNNNT

>M26_MADS15
ANNNNNNNGNANNTGGGGANTGTTATCTTAATTGTTACTTAATTATAGTTTAAGCTCCCTAGTTGTTGTAGTTGA
ATTGCACCAAGTTTTCATAATTTCATTTGTTTCTGCAAGTTTCAAACTTTTTGTTGGTGATCTAGAATATAGTAATTA
ATTAAAGTTCTGGGGTTTTTATTTTAATTAGAAAGGTGCTTAATTGATTGATCTCTTTGTTCTTATTGATTCAGCTTC
CAACTATGGCCTATGAAAGCAAATCCTTGTCCTTGGACTCTCCCCAGAGAAAATTGGGTAGGGGAAAGATCGAGA
TTAAGCGGATCGAAAACACAACGAATCGTCAAGTGACCTTCTGCAAGAGGCGCAATGGGTTGCTCAAGAAGGCCT
ATGAACTCTCTGTGCTCTGTGATGCAGAGGTTGCTCTCATAGTCTTCTCTAACCGTGGCCGCCTCTATGAgTATGCC
AACAATAGGNAATATTATTGCTTCAATTTACTTGCTAAATTTTCCTCTTCTTGTTCTTCGTTTN

>M26_MADS221
ANNNNNNNNNNNNNCTACNTAGTTGTTGTAGTTGATTGCTTTCATTTGTTTCTGCAAGTTAATTTCAAACTTTTTG
TTGGTGATCAAGAATATAGTAATTGAAGTTCTGGGGTTTTTGTTTTATTTAAAAAGGTGCTTAATTGATTGATCTCT
TTGTTCTTATTGATTCAGCTTGCAACTATGGCCAATGAAAACAAATCCTTGTCAATCGACTCTCCCCAGAGAAAATT
GGGTAGGGGAAAGATCGAGATCAAGCGGATCGAAAACACGACCAATCGTCAAGTGACCTTCTGCAAGAGGCGCA

ATGGGTTGCTCAAGAAGGCCTATGAACTCTCTGTGCTCTGTGATGCAGAGGTTGCTCTCATAGTCTTCTCTAACCGT GGCCGCCTCTATGAGTATGCCAACAACAGGTAATAATATTATTGCTTCAATTTTCTTGCTTAATTTTCTCTTCTTATTC TTTTATTTTTTCTAATGGAAGGCTTTTAGTATTATTTTCAGACCCAAGTTTTATTTTCAATTTACCTTTTAATAAGATTT CTTTGTTCTTGTTAAAGTTCTTTTCTTTTTCTTTCTTCTTCTGTGCAAATTTTTGTCACAATTTAGCATTTCAAGAAGTT ATTGGCACTTCAGTCCCTCAAGNT

WT sequences – trimmed to first exon

>RG_MADS15

ATGGCCTATGAAAGCAAATCCTTGTCCwTGGACTCTCCCCAGAGAAAATTGGGTAGGGGAAAGATCGAGATTAAGC GGATCGAAAACACAACGAATCGTCAAGTGACCTTCTGCAAGAGGCGCAATGGGTTGCTCAAGAAGGCCTATGAACT CTCTGTGCTCTGTGATGCAGAGGTTGCTCTCATAGTCTTCTCTAACCGTGGCCGCCTCTATGAGTATGCCAACAATAG

>M26_MADS15

ATGGCCTATGAAAGCAAATCCTTGTCCTTGGACTCTCCCCAGAGAAAATTGGGTAGGGGAAAGATCGAGATTAAGCG GATCGAAAACACAACGAATCGTCAAGTGACCTTCTGCAAGAGGCGCAATGGGTTGCTCAAGAAGGCCTATGAACTC TCTGTGCTCTGTGATGCAGAGGTTGCTCTCATAGTCTTCTCTAACCGTGGCCGCCTCTATGAgTATGCCAACAATAG

>RG_MADS221

ATGGCCAATGAAAACAAATCCTTGTCAATsGACTCTCCCCAGAGAAAATTGGGTAGGGGAAAGATCGAGATCAAGCG GATCGAAAACACrACCAATCGTCAAGTGACCTTCTGCAAGAGGCGCAATGGGTTGCTCAAGAAGGCCTATGAACTCT CTGTGCTCTGTGATGCAGAGGTTGCTCTCATAGTCTTCTCTAACCGTGGCCGCCTCTATGAGTATGCCAACAACAG

>M26_MADS221

ATGGCCAATGAAAACAAATCCTTGTCAATCGACTCTCCCCAGAGAAAATTGGGTAGGGGAAAGATCGAGATCAAGC GGATCGAAAACACGACCAATCGTCAAGTGACCTTCTGCAAGAGGCGCAATGGGTTGCTCAAGAAGGCCTATGAACT CTCTGTGCTCTGTGATGCAGAGGTTGCTCTCATAGTCTTCTCTAACCGTGGCCGCCTCTATGAGTATGCCAACAACAG

Event 508

MADS15

>pTOPO-508-MAD15-colony-1-M13F

NNNNNNNNNNNNAGGGCGATTGGGCCCTCTAGATGCATGCTCGAGCGGCCGCCAGTGTGATGGATATCTGCAGAA TTCGCCCTTTTTCATTTGTTTCTGCAAGTTTCAAACTTTTTGTTGGTGATCTAGAATATAGTAATTAATTAAAGTTCTGG GGTTTTTATTTTAATTAGAAAGGTGCTTAATTGATTGATCTCTTTGTTCTTATTGATTCAGCTTCCAACTATGGCCTATGA AAGCAAATCCTTGTCCTTGGACTCTCCCCAGAGAAAATTGGGTAGGGGAAAGATCGAGATTAAGCGGATCGAAAAC

ACAACGAATCGTCAAGTGACCTTCTGCAAGAGGCGCAATGGGTTGCTCAAGAAGGCCTATGAACTCTCTGTGCTATG
TGATGCAGAGGTTGCTCTCATAGTCTTCTCTAACCGTGGCCGCCTCTATGAGTATGCCAACAATAGGTAATATTATTGCT
TCAATTTACTTGAAGGGCGAATTCCAGCACACTGGCGGCCGTTACTAGTGGATCCGAGCTCGGTACCAAGCTTGATG
CATAGCTTGAGTATTCTATAGTGTCACCTAAATAGCTTGGCGTAATCATGGTCATAGCTGTTTCCTGTGTGAAATTGTTA
TCCGCTCACAATTCCACACAACATACGAGCCGGAAGCATAAAGTGTAAAGCCTGGGGTGCCTAATGAGTGAGCTAAC
TCACATTAATTGCGTTGCGCTCACTGCCCGCTTTCCAGTCGGGAAACCTGTCGTGCCAGCTGCATTAATGAATCGGCC
AACGCGCGGGGAGAGGCGGTTTGCGTATTGGGCGCTCTTCCGCTTCCTCGCTCACTGACTCGCTGCGCTCGGTCGTT
CGGCTGCGGCGAGCGGTATCAGCTCACTCAAAGGCGGTAATACGGTTATCCACAGAATCAGGGGATAACGCNNNAA
GAACATGTGAGCAAAAGGCCAGCAAAGGCCAGGAACCGTN

>pTOPO-508-M15-colony6-M13F_F03.ab1
NNNNNNNNNNNNGGGCGATTGGGCCCTCTAGATGCATGCTCGAGCGGCCGCCAGTGTGATGGATATCTGCAGAAT
TCGCCCTTCTATTGTTGGCATACTCATAGAGGCGGCCACGGTTAGAGAAGACTATGAGAGCAACCTCTGCATCACAGA
GCACAGAGAGTTCATAGGCCTTCTTGAGCAACCCATTGCGCCTCTTGCAGAAGGTCACTTGACGATTCGTTGTGTTTT
CGATCCGCTTAATCTCGATCTTTCCCCTACCCAATTTTCTCTGGGGAGAGTCCAAGGACAAGGATTTGCTTTCATAGGC
CATAAGGGCGAATTCCAGCACACTGGCGGCCGTTACTAGTGGATCCGAGCTCGGTACCAAGCTTGATGCATAGCTTG
AGTATTCTATAGTGTCACCTAAATAGCTTGGCGTAATCATGGTCATAGCTGTTTCCTGTGTGAAATTGTTATCCGCTCAC
AATTCCACACAACATACGAGCCGGAAGCATAAAGTGTAAAGCCTGGGGTGCCTAATGAGTGAGCTAACTCACATTAA
TTGCGTTGCGCTCACTGCCCGCTTTCCAGTCGGGAAACCTGTCGTGCCAGCTGCATTAATGAATCGGCCAACGCGCG
GGGAGAGGCGGTTTGCGTATTGGGCGCTCTTCCGCTTCCTCGCTCACTGACTCGCTGCGCTCGGTCGTTCGGCTGCG
GCGAGCGGTATCAGCTCACTCAAAGGCGGTAATACGGTTATCCACAGAATCAGGGGATAACGCAGGAAAGAACATG
TGAGCAAAAGGNCAGCAAAAGGCCAGGAACCGTAAAAANNNNCGTTGCTGGCGTTTTTCCATAGGCTCCGCCCCC
CTGACGAGCATCACAAAATCGACGCTCAAGTCAGAGGTGGCGAAACCCGACAGGACTATAAAGATACCAGGCGTTT
CCCCNGGAAGCTCCCTCGTGCGCTCTCCTGTTCCGACCCTGCCGCTTACCGGATACCTGTCCGCCTTTCTCCCTTCGG
GA

MADS221

>pTOPO-508-M221-colony4-M13F_A03.ab1
NNNNNNNNNNNNNNGGGCGATTGGGCCCTCTAGATGCATGCTCGAGCGGCCGCCAGTGTGATGGATATCTGCAGA
ATTCGCCCTTCTGTTGTTGGCATACTCATAGAGGCGGCCACGGTTAGAGAAGACTATGAGAGCAACCTCTGCATCACA
GAGCACAGAGAGTTCATAGGCCTTCTTGAGCAACCCATTGCGCCTCTTGCAGAAGGTCACTTGACGATTGGTCGTGT
TTTCNATCCGCTTGATCTCGATCTTTCCCCTACCCAATTTTCTCTGGGGAGAGTCGATTGACAAGGATTTGTTTTCATT
GGCCATAAGGGCAAATTCCACCACACTGGCGGCCGTTACTAGTGGATCCAAGCTCGGTACCAAGCTTGATGCATAGC
TTGAGTATTCTATAGTGTCACCTAAATAGCTTGGCGTAATCATGGTCATAGCTGTTTCCTGTGTGAAATTGTTATCCGCT
CACAATTCCACACAACATACGAGCCGGAAGCATAAAGTGTAAAGCCTGGGGTGCCTAATGANTGAGCTAACTCACAT
TAATTGCGTTGCGCTCACTGCCCGCTTTCCAGTCGGGAAACCTGTCGTGCCAGCTGCATTAATGAATCGGCCAACGCG
CGGGGAGAGGCGGTTTGCGTATTGGGCGCTCTTCCGCTTCCTCGCTCACTGACTCNCTGCGCTCGGTCGTTCGGCTG
CGGCGAGCGGTATCANCTCACTCAAAGGCGGTAATACGGTTATCCACANAATCNNGNNATAACNNGGANANAACA
TGTGANCAAAAGGNCAGCAAAAGGNCAGGAACCGTAAAAAGGNCGCGTTGCTGGCGTTTTTCCATAGGCTCCGCC
CCCCTGACGANCATCACAAAAATCGACGCTCAGTCAGANGTGGCNAAACCCGACGGGACTATAAAGATNCCAGGC
GTTTCCCCCTGNAGCTCCCTCGTGCGCTCTCNNNTCCGACCCTGCCGNTTACCGNANNCCTGNCCGNNTTTCNNCN
NTCGGGAANNNNGGCGNNNNNATNN

>pTOPO-508-M221-colony6-M13F_B03.ab1

NNNNNNNNNNNNNNNGGGCGATTGGGCCCTCTAGATGCATGCTCGAGCGGCCGCCAGTGTGATGGATATCTGCAG
AATTCGCCCTTATGGCCAATGAAAACAAATCCTTGTCAATCGACTCTCCCCAGAGAAAATTGGGTAGGGGAAAGATC
GAGATCAAGCGGATCGAAAACACGACCAATCGTCAAGTGACCTTCTGCAAGAGGCGCAATGGGTTGCTCAAGAAG
GCCTATGAACTCTCTGTGCTCTGTGATGCATAGGTTGCTCTCATAGTCTTCTCTAACCGTGGCCGCCTCTATGAGTATGC
CAACAACAGAAGGGCGAATTCCAGCACACTGGCGGCCGTTACTAGTGGATCCGAGCTCGGTACCAAGCTTGATGCA
TAGCTTGAGTATTCTATAGTGTCACCTAAATAGCTTGGCGTAATCATGGTCATAGCTGTTTCCTGTGTGAAATTGTTATC
CGCTCACAATTCCACACAACATACGAGCCGGAAGCATAAAGTGTAAAGCCTGGGGTGCCTAATGAGTGAGCTAACTC
ACATTAATTGCGTTGCGCTCACTGCCCGCTTTCCAGTCGGGAAACCTGTCGTGCCAGCTGCATTAATGAATCGGCCAA
CGCGCGGGGAGAGGCGGTTTGCGTATTGGGCGCTCTTCCGCTTCCTCGCTCACTGACTCGCTGCGCTCGGTCGTTCG
GCTGCGGCGAGCGGTATCAGCTCACTCAAAGGCGGTAATACGGTTATCCACAGAATCANGGGATAACGCANGAAAG
ANNATGTGAGCAAAAGGNCAGCAAAANNCANGAACCGTAAAAANNCNCGTTTGCTGGCGTTTTTCCATANGCTCC
GCCCCCCTGACGAGCATCACAAAAATCGACGCTCAAGTCANNNNGGCGAAACCCGACANGACTATAANATACCAG
GCGTTTCCCCCTGGANCTCCCTCNTGCGCTCTNCTGTTCCGACCTGCNNTTACCGGATACCTGNCCGCCTTTCTNCN
NNNNNANCGTGGNGCTNNNCNTAGCTCANGCTGNNNNNNTCAGTTNGNGNN

Event 509

<u>MADS15</u>

>509-M150-colony4-M13F_F05.ab1
NNNNNNNNNNNNNNNNGCGANTGGGCCCTCTAGATGCATGCTCGAGCGGCCGCCAGTGTGATGGATATCTGCAGA
ATTCGCCCTTCTATTGTTGGCATACTCATAGAGGCGGCCACGGTTAGAGAAGACTATGAGAGCAACCTCTGCATCACA
GAGCACAGAGAGTTCATAGGCCTTCTTGAGCAACCCATTGCGCCTCTTGCAGAAGGTCACTTGACGATTCGTTGTGT
TTTCGATCCGCTTAATCTCGATCTTTCCCCTACCCAATTTTCTCTGGGGAGAGTCCAAGGACAAGGATTTGCTTTCATA
GGCCATAAGGGCGAATTCCAGCACACTGGCGGCCGTTACTAGTGGATCCGAGCTCGGTACCAAGCTTGATGCATAGC
TTGAGTATTCTATAGTGTCACCTAAATAGCTTGGCGTAATCATGGTCATAGCTGTTTCCTGTGTGAAATTGTTATCCGCT
CACAATTCCACACAACATACGAGCCGGAAGCATAAAGTGTAAAGCCTGGGGTGCCTAATGAGTGAGCTAACTCACAT
TAATTGCGTTGCGCTCACTGCCCGCTTTCCAGTCGGGAAACCTGTCGTGCCAGCTGCATTAATGAATCGGCCAACGCG
CGGGGAGAGGCGGTTTGCGTATTGGGCGCTCTTCCGCTTCCTCGCTCACTGACTCGCTGCGCTCGGTCGTTCGGCTG
CGGCGAGCGGTATCAGCTCACTCAAAGGCGGTAATACGGTTATCCACAGAATCAGGGGATAACGCAGGAAAGAACA
TGTGAGCAAAAGGNCAGCAAAAGGCCAGGAACCGTAAAAAGGCCGCGTTGCTGGCGTTTTTCCATNNCTCCGCCC
CCCTGACGAGCATCACAAAAATCGACGCTCAAGTCAGAGGTGGCGAAACCCGACAGGACTATAAAGATACCAGGCG
TTTCCCCCNGGAAGCTNCCNTCGTGCGCTCTCCTGTTCCGACCCTGCNCTTACCNNATACCTGTCCGNCTTTCTCCCT
TNGGNNCGNGGCGCTTTCTCATAGCTCCCGCTGTNGNATCTCANTCGGNGNNGNCNTNNNNNCNAGCTGGGNT
NNNNNGCNNCNAACCCCCN

>pTOPO_MADS515_509_colony_1-M13F_A07.ab1

NNNNNNNNNNNNNNNNCGATTGGGCCCTCTAGATGCATGCTCGAGCGGCCGCCAGTGTGATGGATATCTGCAGAAT
TCGCCCTTCATTTGTTTCTGCAAGTTTCAAACTTTTTGTTGGTGATCTAGAATATAGTAATTAATTAAAGTTCTGGGGTT
TTTATTTTAATTAGAAAGGTGCTTAATTGATTGATCTCTTTGTTCTTATTGATTCAGCTTCCAACTATGGCCTATGAAAGC
AAATCCTTGTCCTTGGACTCTCCCCAGAGAAAATTGGGTAGGGGAAAGATCGAGATTAAGCGGATCGAAAACACAA
CGAATCGTCAAGTGACCTTCTGCAAGAGGCGCAATGGGTTGCTCAAGAAGGCCTATGAACTCTCTGTGCTCTGTGAT
GCAGAGGTTGCTCTCATAGTCTTCTCTAACCGTGGCCGCCTCTATGAGTATGCCAACAATAGGTAATATTATTGCTTCAA
TTTACTTGAAGGGCGAATTCCAGCACACTGGCGGCCGTTACTAGTGGATCCGAGCTCGGTACCAAGCTTGATGCATA
GCTTGAGTATTCTATAGTGTCACCTAAATAGCTTGGCGTAATCATGGTCATAGCTGTTTCCTGTGTGAAATTGTTATCCG
CTCACAATTCCACACAACATACGAGCCGGAAGCATAAAGTGTAAAGCCTGGGGTGCCTAATGAGTGAGCTAACTCAC

ATTAATTGCGTTGCGCTCACTGCCCGCTTTCCAGTCGGGAAACCTGTCGTGCCAGCTGCATTAATGAATCGGCCAACG
CGCGGGGAGAGGCGGTTTGCGTATTGGGCGCTCTTCCGCTTCCTCGCTCACTGACTCGCTGCGCTCGGTCGTTCGGC
TGCGGCGAGCGGTATCAGCTCACTCAAAGGCGGTAATACGGTTATCCACAGAATCNGGGGATAACGCAGGAAAGAA
CATGTGAGCAAAAGGCAGCAAAANGCCAGGNACCGTAAAAGGCCNCGNTTGCTGGCNTTTTNCNNN

MADS221

>pTOPO-509-M221-colony1-M13F_C05.ab1
NNNNNNNNNNNNNNGGGCGATTGGGCCCTCTAGATGCATGCTCGAGCGGCCGCCAGTGTGATGGATATCTGCAGA
ATTCGCCCTTATGGCCAATGAAAACAAATCCTTGTCAATCGACTCTCCCCAGAGAAAATTGGGTAGGGGAAAGATCG
AGATCAAGCGGATCGAAAACACGACCAATCGTCAAGTGACCTTCTGCAAGAGGCGCAATGGGTTGCTCAAGAAGGC
CTATGAACTCTCTGTGCTCTGTGATGCAGAGGTTGCTCTCATAGTCTTCTCTAACCGTGGCCGCCTCTATGAGTATGCC
AACAACAGAAGGGCGAATTCCAGCACACTGGCGGCCGTTACTAGTGGATCCGAGCTCGGTACCAAGCTTGATGCAT
AGCTTGAGTATTCTATAGTGTCACCTAAATAGCTTGGCGTAATCATGGTCATAGCTGTTTCCTGTGTGAAATTGTTATCC
GCTCACAATTCCACACAACATACGAGCCGGAAGCATAAAGTGTAAAGCCTGGGGTGCCTAATGAGTGAGCTAACTCA
CATTAATTGCGTTGCGCTCACTGCCCGCTTTCCAGTCGGGAAACCTGTCGTGCCAGCTGCATTAATGAATCGGCCAAC
GCGCGGGGAGAGGCGGTTTGCGTATTGGGCGCTCTTCCGCTTCCTCGCTCACTGACTCGCTGCGCTCGGTCGTTCG
GCTGCGGCGAGCGGTATCAGCTCACTCAAAGGCGGTAATACGGTTATCCACAGAATCAGGGGATAACGCAGGAAAG
AACATGTGAGCAAAAGGCCAGCAAAAGGCCAGGAACCGTAAAAAGGCCGCGTTGCTGGCGTTTTTCCATAGGCTCC
GCCCCCCTGACGAGCATCACAAAAATCGACGCTCAAGTCAGANGTGGCGAAACCCGACAGGACTATAAAGATACCA
GGCGTTTCCCCCTGNAAGCTCCCTCGTGCGCTCTCCTGT
TCCGACCCTGCCGCTTACCGGATACCTGTCNCCTTTCTCCTTCGGGAGCGTGNGCTTTCTCATAGCTCANGCTGTNNN
ATCTCAGTTCGGNGTNGNCGTCGNNCNAGCTGGGNTGNGTGCNCGAACCCCCGTTCN

>pTOPO-509-M221-colony8-M13F_D05.ab1
NNNNNNTNNNNNGGGCGATTGGGCCCTCTAGATGCATGCTCGAGCGGCCGCCAGTGTGATGGATATCTGCAGAAT
TCGCCCTTATGGCCAATGAAAACAAATCCTTGTCAATCGACTCTCCCCAGAGAAAATTGGGTAGGGGAAAGATCGAG
ATCAAGCGGATCGAAAACACGACCAATCGTCAAGTGACCTTCTGCAAGAGGCGCAATGGGTTGCTCAAGAAGGCCT
ATGAACTCTCTGTGCTCTGTGATGCAGAGGTTGCTCTCATAGTCTTCTCTAACCGTGGCCGCCTCTATGAGTATGCCAA
CAACAAAAGGGGAAATTCCNCCCCCTGGGGGGCCNTTATTATGGNNTCCAACTCNGNACCNAACTTTNNGGCTAA
NTTNNNTNTTTTATTNNGGCCCCCTAATAANTTGGNGTAATCATGGNCNTAANTGGTTTCTGGGNNAAANTNNNN
TCCNNTCCNNNTTNCNNNCCANNTTANAACCCGAANNNTTAANNGGAAAANCCGGGGNGNCNNANGNNNNAA
CTNANTNNNTTNANTGGNNTNGNCTNNNTNGCCNNTTTNCNNNCCGGAAANCTGGNNNNNCNNNTGNNTNN
TGGNTNNGGCCACNNGCGNGGAAAGNGNTTNNNNAATGGGNNCTNTNNNNNTNNNNNTNACTGANTCNCTN
NNNTNNNNNTNNCNGNGNNANAGNNTCNNNNNCTNNNNNNNNNNNNNNGTNNNNNNNNNNNNNGNNNA
ANGCNNAANNANATGTGANNNNAAGNNNNNNANGNNNNNAANNNTAAAAGNNCGTTNNNNNNGNNNNNC
NNNNGNTNCNNCCCCTGANANNNNCNNNNAANNNNNNNNNNNNNTCNNAGNGNNNNNNNNNNNNNNNNN
NNNANNCNNNNNTNNCNNNANNNNCNNNNNNNNNNNNGTNNANNTGNNNNNNNGNNNNNNNNNTTCNNN
NNNNNNNNNNCNNNCNNNNGNTTCNNNNGNNNNNNNNNNNNGNNNNGNNNA

Event 510

MADS15

>pTOPO-510-M15-colony7-M13F_B04.ab1
NNNNNNNNNNNNNAGGGCGATTGGGCCCTCTAGATGCATGCTCGAGCGGCCGCCAGTGTGATGGATATCTGCAG
AATTCGCCCTTCTATTGTTGGCATACTCATAGAGGCGGCCACGGTTAGAGAAGACTATGAGAGCAACCTCTGCATCAC
AGAGCACAGAGAGTTCATAGGCCTTCTTGAGCAACCCATTGCGCCTCTTGCAGAAGGTCACTTGACGATTCGTTGTG
TTTTCGATCCGCTTAATCTCGATCTTTCCCCTACCCAATTTTCTCTGGGGAGAGTCCAAGGACAAGGATTTGCTTTCAT
AGGCCAAGGGCGAATTCCAGCACACTGGCGGCCGTTACTAGTGGATCCGAGCTCGGTACCAAGCTTGATGCATAGC
TTGAGTATTCTATAGTGTCACCTAAATAGCTTGGCGTAATCATGGTCATAGCTGTTTCCTGTGTGAAATTGTTATCCGCT
CACAATTCCACACAACATACGAGCCGGAAGCATAAAGTGTAAAGCCTGGGGTGCCTAATGAGTGAGCTAACTCACAT
TAATTGCGTTGCGCTCACTGCCCGCTTTCCAGTCGGGAAACCTGTCGTGCCAGCTGCATTAATGAATCGGCCAACGCG
CGGGGAGAGGCGGTTTGCGTATTGGGCGCTCTTCCGCTTCCTCGCTCACTGACTCGCTGCGCTCGGTCGTTCGGCTG
CGGCGAGCGGTATCAGCTCACTCAAAGGCGGTAATACGGTTATCCACAGAATCANGGGATAACGCANGAAAGAACA
TGTGAGCAAAANGNCANCAAAANGNNANNAACCGTAAAANNNCGCGTTGCTGGCGTTTTTCCATANGCTCCGCC
CCCCTGACGAGCATCACAAAAATCGACGCTCAAGTCANANGTGGCGAAACCCGACAGGACTATAAANATACCNNGC
GTTTCCCCCTGNANCTCCCTCNTGCGCTCTCCTGTTCCNACCCTGCNNCTTNNCGNATACCTGTCCGCCNTTNTCCCT
NNNGGAANGTGGNGCTTNCTCNTANCTCACGCTGTNGNNNNTCNNN

>pTOPO_MADS515_510_colony_1-M13F_E08.ab1

NNNNNNNNNNNNNNNNNGATTGGGCCNCTAGATGCATGCTCGAGCGGCCGCCAGTGTGATGGATATCTGCAGAA
TTCGCCCTTTTCATTTGTTTCTGCAAGTTTCAAACTTTTTGTTGGTGATCTAGAATATAGTAATTAATTAAAGTTCTGGG
GTTTTTATTTTAATTAGAAAGGTGCTTAATTGATTGATCTCTTTGTTCTTATTGATTCAGCTTCCAACTATGGCCTATGAA
AGCAAATCCTTGTCCTTGGACTCTCCCCAGAGAAAATTGGGTAGGGGAAAGATCGAGATTAAGCGGATCGAAAACA
CAACGAATCGTCAAGTGACCTTCTGCAAGAGGCGCAATGGGTTGCTCAAGAAGGCCTATGAACTCTCTGTGCTCTGT
GATGCAGAGGTTGCTCTCATAGTCTTCTCTAACCGTGGCCGCCTCTATGAGTATGCCAACAATAGGTAATATTATTGCTT
CAATTTACTTGAAGGGCGAATTCCAGCACACTGGCGGCCGTTACTAGTGGATCCGAGCTCGGTACCAAGCTTGATGC
ATAGCTTGAGTATTCTATAGTGTCACCTAAATAGCTTGGCGTAATCATGGTCATAGCTGTTTCCTGTGTGAAATTGTTAT
CCGCTCACAATTCCACACAACATACGAGCCGGAAGCATAAAGTGTAAAGCCTGGGGTGCCTAATGAGTGAGCTAACT
CACATTAATTGCGTTGCGCTCACTGCCCGCTTTCCAGTCGGGAAACCTGTCGTGCCAGCTGCATTAATGAATCGGCCA
ACGCGCGGGGAGAGGCGGTTTGCGTATTGGGCGCTCTTCCGCTTCCTCGCTCACTGACTCGCTGCGCTCGGTCGTTC
GGCTGCGGCGAGCGGTATCAGCTCACTCAAAGGCGGTAATACGGTTATCCACAGAATCNGGGGATAACGCAGGAAA
GAACATGTGAGCAAANGCCAGNNN

<u>MADS221</u>

>510-M221-colony8-M13F_H05.ab1
NNNNNNNNNNNNGGGCGATTGGGCCCTCTAGANGCATGCTCGAGCGGCCGCCAGTGTGATGGATATCTGCAGAA
TTCGCCCTTATGGCCAATGAAAACAAATCCTTGTCAATCGACTCTCCCCAGAGAAAATTGGGTAGGGGAAAGATCGA
GATCAAGCGGATCGAAAACACGACCAATCGTCAAGTGACCTTCTGCAAGAGGCGCAATGGGTTGCTCAAGAAGGCC
TATGAACTCTCTGTGCTCTGTGATGCAGAGGTTGCTCTCATAGTCTTCTCTAACCGTGGCCGCCTCTATGAGTATGCCA
ACAACAGAAGGGCGAATTCCAGCACACTGGCGGCCGTTACTAGTGGATCCGAGCTCGGTACCAAGCTTGATGCATA
GCTTGAGTATTCTATAGTGTCACCTAAATAGCTTGGCGTAATCATGGTCATAGCTGTTTCCTGTGTGAAATTGTTATCCG
CTCACAATTCCACACAACATACGAGCCGGAAGCATAAAGTGTAAAGCCTGGGGTGCCTAATGAGTGAGCTAACTCAC
ATTAATTGCGTTGCGCTCACTGCCCGCTTTCCAGTCGGGAAACCTGTCGTGCCAGCTGCATTAATGAATCGGCCAACG
CGCGGGGAGAGGCGGTTTGCGTATTGGGCGCTCTTCCGCTTCCTCGCTCACTGACTCGCTGCGCTCGGTCGTTCGGC
TGCGGCGAGCGGTATCAGCTCACTCAAAGGCGGTAATACGGTTATCCACAGAATCAGGGGATAACGCAGGAAAGAA
CATGTGAGCAAAAGGNCAGCAAAAGGNCAGGAACCGTAAAAAGGNCGCGTTGCTGGCGTTTTTCCATAGGCTCCG

CCCCCCTGACGAGCATCACAAAAATCGACGCTCAAGTCANAGGTGGCGAAACCCGACAGGACTATAANATACCAGG
CGTTTCCCCCTNNNGCTCCCTCGNGCGCTCTNCTGTTCCGACCCTGCCGCTTACCGGATACCTGTCCGCCTTTCTCCC
TTCGGGAAGNNGNGGCNCTTTCNNCATAGCTNNNNNTNNN

>pTOPO_MADS221_510_colony_3-M13F_C07.ab1

NNNNNNNNNNNNNNNNNGATTGGGCCCTCTAGATGCATGCTCGAGCGGCCGCCAGTGTGATGGATATCTGCAGAA
TTCGCCCTTAGCAAGAAAATTGAAGCAATAATATTATTACCTGTTGTTGGCATACTCATAGAGGCGGCCACGGTTAGA
GAAGACTATGAGAGCAACCTCTGCATCACAGAGCACAGAGAGTTCATAGGCCTTCTTGAGCAACCCATTGCGCCTCT
TGCAGAAGGTCACTTGACGATTGGTCGTGTTTTCGATCCGCTTGATCTCGATCTTTCCCCTACCCAATTTTCTCTGGGG
AGAGTCGATTGACAAGGATTTGTTTTCATTGGCCATAGTTGCAAGCTGAATCAATAAGAACAAAGAGATCAATCAATT
AAGCACCTTTTTAAATAAAACAAAAACCCCAGAACTTCAATTACTATATTCTTGATCACCAACAAAAAGTTTGAAATTA
ACTTGCAGAAACAAATGAAAAGGGCGAATTCCAGCACACTGGCGGCCGTTACTAGTGGATCCGAGCTCGGTACCAA
GCTTGATGCATAGCTTGAGTATTCTATAGTGTCACCTAAATAGCTTGGCGTAATCATGGTCATAGCTGTTTCCTGTGTGA
AATTGTTATCCGCTCACAATTCCACACAACATACGAGCCGGAAGCATAAAGTGTAAAGCCTGGGGTGCCTAATGAGTG
AGCTAACTCACATTAATTGCGTTGCGCTCACTGCCCGCTTTCCAGTCGGGAAACCTGTCGTGCCAGCTGCATTAATGA
ATCGGCCAACGCGCGGGGAGAGGCGGTTTGCGTATTGGGCGCTCTTCCGCTTCCTCGCTCACTGACTCGCTGCGCTC
GGTCGTTCGGCTGCGGCGAGCGGTATCAGCTCACTCAAAGGCGGTAATANNNTATCCACAGAATCAGGGGATAACG
CNNNAAGAACATGTGAGCAAANNCAGCAAAAGGCAGGAACCGTAAAAGGCCGCGTTGCTGCGTTTTCCNTAGCTC
CGCCCCTGACNAGCATCNNAAAATCGACNCTCAGTCNNAGGTGGNNAANCNACNGGACNNNAAGANNCAGNNT
TNCCCNGGAANNNNCCTCNNGNGNNTNNNNNNNNNCGACCNGCCNNN

Event 512

MADS15

>pTOPO-512-M15-colony4-M13F_F08.ab1
NNNNNNNNNNNNNNNNGGCGATTGGGCCCTCTAGATGCATGCTCGAGCGGCCGCCAGTGTGATGGATATCTGCAG
AATTCGCCCTTATGGCCTATGAAAGCAAATCCTTGTCCATGGACTCTCCCCAGAGAAAATTGGGTAGGGGAAAGATC
GAGATTAAGCGGATCGAAAACACAACGAATCGTCAAGTGACCTTCTGCAAGAGGCGCAATGGGTTGCTCAAGAAG
GCCTATGAACTCTCTGTGCTCTGTGATGCAGAGGTTGCTCTCATAGTCTTCTCTAACCGTGGCCGCCTCTATGAGTATG
CCAACAATAGAAGGGCGAATTCCAGCACACTGGCGGCCGTTACTAGTGGATCCGAGCTCGGTACCAAGCTTGATGCA
TAGCTTGAGTATTCTATAGTGTCACCTAAATAGCTTGGCGTAATCATGGTCATAGCTGTTTCCTGTGTGAAATTGTTATC
CGCTCACAATTCCACACAACATACGAGCCGGAAGCATAAAGTGTAAAGCCTGGGGTGCCTAATGAGTGAGCTAACTC
ACATTAATTGCGTTGCGCTCACTGCCCGCTTTCCAGTCGGGAAACCTGTCGTGCCAGCTGCATTAATGAATCGGCCAA
CGCGCGGGGAGAGGCGGTTTGCGTATTGGGCGCTCTTCCGCTTCCTCGCTCACTGACTCGCTGCGCTCGGTCGTTCG
GCTGCGGCGAGCGGTATCAGCTCACTCAAAGGCGGTAATACGGTTATCCACAGAATCAGGGGATAACGCANGAAAG
AACATGTGAGCAAAANGNCAGCAAAAGGCCAGGAACCGTAAAAANNCGCGTTG

>pTOPO-512-M15-colony5-M13F_A06.ab1
NNNNNNNNNNNNGGGCGATTGGGCCCTCTAGATGCATGCTCGAGCGGCCGCCAGTGTGATGGATATCTGCAGAAT
TCGCCCTTATGGCCTATGAAAGCAAATCCTTGTCCATGGACTCTCCCCAGAGAAAATTGGGTAGGGGAAAGATCGAG
ATTAAGCGGATCGAAAACACAACGAATCGTCAAGTGACCTTCTGCAAGAGGCGCAATGGGTTGCTCAAGAAGGCCT
ATGAACTCTCTGTGCTCTGTGATGCAGAGGTTGCTCTCATAGTCTTCTCTAACCGTGGCCGCCTCTATGAGTATGCCAA
CAATAGAAGGGCGAATTCCAGCACACTGGCGGCCGTTACTAGTGGATCCGAGCTCGGTACCAAGCTTGATGCATAGC
TTGAGTATTCTATAGTGTCACCTAAATAGCTTGGCGTAATCATGGTCATAGCTGTTTCCTGTGTGAAATTGTTATCCGCT

CACAATTCCACACAACATACGAGCCGGAAGCATAAAGTGTAAAGCCTGGGGTGCCTAATGAGTGAGCTAACTCACAT
TAATTGCGTTGCGCTCACTGCCCGCTTTCCAGTCGGGAAACCTGTCGTGCCAGCTGCATTAATGAATCGGCCAACGCG
CGGGGAGAGGCGGTTTGCGTATTGGGCGCTCTTCCGCTTCCTCGCTCACTGACTCGCTGCGCTCGGTCGTTCGGCTG
CGGCGAGCGGTATCAGCTCACTCAAAGGCGGTAATACGGTTATCCACAGAATCAGGGGATAACGCAGGAAAGAACA
TGTGAGCAAAAGGCCAGCAAAAGGCCAGGAACCGTAAAAAGGNCGCGTTGCTGGCGTTTTTCCATANGCTCCGCC
CCCCTGACGAGCATCACAAAAATCGACGCTCAAGTCAGAGGTGGCGAAACCCGACAGGACTATAAAGATACCAGGC
GTTTCCCCCNGNAAGCTCCCTCGTGCGCTCTCCTGTTCCGACCCTGCCGCTTACCGNATACCTGTCCGCCTTTCTCCCT
TCGGGAANCNNGNNCTTTCTCATAGCTCACGCTGTANNNTNNNNN

<u>MADS221</u>

>pTOPO-512-M221-colony-2-M13F_G04.ab1
NNNNNNNNNNNNNNNGGCGATTGGGCCCTCTAGATGCATGCTCGAGCGGCCGCCAGTGTGATGGATATCTGCAG
AATTCGCCCTTATGGCCAATGAAAACAAATCCTTGTCAATCGACTCTCCCCAGAGAAAATTGGGTAGGGGAAAGATC
GAGATCAAGCGGATCGAAAACACGACCAATCGTCAAGTGACCTTCTGCAAGAGGCGCAATGGGTTGCTCAAGAAG
GCCTATGAACTCTCTGTGCTCTGTGATGCAGAGGTTGCTCTCATAGTCTTCTCTAACCGTGGCCGCCTCTATGAGTATG
CCAACAACAGAAGGGCGAATTCCAGCACACTGGCGGCCGTTACTAGTGGATCCGAGCTCGGTACCAAGCTTGATGC
ATAGCTTGAGTATTCTATAGTGTCACCTAAATAGCTTGGCGTAATCATGGTCATAGCTGTTTCCTGTGTGAAATTGTTAT
CCGCTCACAATTCCACACAACATACGAGCCGGAAGCATAAAGTGTAAAGCCTGGGGTGCCTAATGAGTGAGCTAACT
CACATTAATTGCGTTGCGCTCACTGCCCGCTTTCCAGTCGGGAAACCTGTCGTGCCAGCTGCATTAATGAATCGGCCA
ACGCGCGGGGAGAGGCGGTTTGCGTATTGGGCGCTCTTCCGCTTCCTCGCTCACTGACTCGCTGCGCTCGGTCGTTC
GGCTGCGGCGAGCGGTATCAGCTCACTCAAAGGCGGTAATACGGTTATCCACAGAATCAGGGGATAACGCAGGAAA
GAACATGTGAGCAAAAGGCCAGCAAAAGGCCAGGAACCGTAAAAAGGCCGCGTTGCTGGCGTTTTTCCATNNCTC
CGCCCCCCTGACGAGCATCACAAAAATCGACGCTCAAGTCANAGGNGGCGAAACCCGACAGGACTATAAAGANAC
CAGGCGTTTCCCCCNNGAAGCTCCCTCGTGCGCTCTNCTGTTNNN

>pTOPO-512-M221-colony-7-M13F_H04.ab1
NNNNNNNNNNNNNNAGGGCGATTGGGCCNTCTAGATGCATGCTCGAGCGGCCGCCAGTGTGATGGATATCTGCA
GAATTCGCCCTTAGGCCAATGAAAACAAATCCTTGTCAATCGACTCTCCCCAGAGAAAATTGGGTAGGGGAAAGATC
GAGATCAAGCGGATCGAAAACACGACCAATCGTCAAGTGACCTTCTGCAAGAGGCGCAATGGGTTGCTCAAGAAG
GCCTATGAACTCTCTGTGCTCTGTGATGCAGAGGTTGCTCTCATAGTCTTCTCTAACCGTGGCCGCCTCTATGAGTATG
CCAACAACAGAAGGGCGAATTCCAGCACACTGGCGGCCGTTACTAGTGGATCCGAGCTCGGTACCAAGCTTGATGC
ATAGCTTGAGTATTCTATAGTGTCACCTAAATAGCTTGGCGTAATCATGGTCATAGCTGTTTCCTGTGTGAAATTGTTAT
CCGCTCACAATTCCACACAACATACGAGCCGGAAGCATAAAGTGTAAAGCCTGGGGTGCCTAATGAGTGAGCTAACT
CACATTAATTGCGTTGCGCTCACTGCCCGCTTTCCAGTCGGGAAACCTGTCGTGCCAGCTGCATTAATGAATCGGCCA
ACGCGCGGGGAGAGGCGGTTTGCGTATTGGGCGCTCTTCCGCTTCCTCGCTCACTGACTCGCTGCGCTCGGTCGTTC
GGCTGCGGCGAGCGGTATCAGCTCACTCAAAGGCGGTAATACGGTTATCCACAGAATCAGGGGATAACGCAGGAAA
GAACATGTGAGCAAAAGGCCAGCAAAAGGCCAGGAACCGTAAAAAGGCCGCGTTGCTGGCGTTTTTCCATAGGCT
CCGCCCCCCTGACGAGCATCACAAAAATCGACGCTCAAGTCAGANGTGGCGAAACCCGACAGGACTATAAAGANAC
CAGGCGTTTCCCCCTGGAAGCTC

Event 514

<u>MADS15</u>

>pTOPO-514-M15-colony5-M13F_B06.ab1
NNNNNNNNNNNNGGGCGATTGGGCCCTCTAGATGCATGCTCGAGCGGCCGCCAGTGTGATGGATATCTGCAGAAT
TCGCCCTTCTATTGTTGGCATACTCATAGAGGCGGCCACGGTTAGAGAAGACTATGAGAGCAACCTCTGCATCACAGA
GCACAGAGAGTTCATAGGCCTTCTTGAGCAACCCATTGCGCCTCTTGCAGAAGGTCACTTGACGATTCGTTGTGTTTT
CGATCCGCTTAATCTCGATCTTTCCCCTACCCAATTTTCTCTGGGGAGAGTCCAAGGACAAGGATTTGCTTTCATAGGC
CAAAGGGCGAATTCCAGCACACTGGCGGCCGTTACTAGTGGATCCGAGCTCGGTACCAAGCTTGATGCATAGCTTGA
GTATTCTATAGTGTCACCTAAATAGCTTGGCGTAATCATGGTCATAGCTGTTTCCTGTGTGAAATTGTTATCCGCTCACA
ATTCCACACAACATACGAGCCGGAAGCATAAAGTGTAAAGCCTGGGGTGCCTAATGAGTGAGCTAACTCACATTAATT
GCGTTGCGCTCACTGCCCGCTTTCCAGTCGGGAAACCTGTCGTGCCAGCTGCATTAATGAATCGGCCAACGCGCGGG
GAGAGGCGGTTTGCGTATTGGGCGCTCTTCCGCTTCCTCGCTCACTGACTCGCTGCGCTCGGTCGTTCGGCTGCGGC
GAGCGGTATCAGCTCACTCAAAGGCGGTAATACGGTTATCCACAGAATCAGGGGATAACGCAGGAAAGAACATGTG
AGCAAAAGGCCAGCAAAAGGCCAGGAACCGTAAAAAGGCCGCGTTGCTGGCGTTTTTCCATAGGCTCCGCCCCCCT
GACGAGCATCACAAAAATCGACGCTCAAGTCAGAGGTGGCGAAACCCGACAGGACTATAAAGATACCAGGCGTTTC
CCCCTNNNAGCTCCCTCGTGCGCTCTCCTGTTCCGACCCTGCCGCTTACCGGATACCTGTCCGCCTTTCTCCCTTCGG
GAAGCGTGNGCTTTCTCATAGCTCACGCTGTAGGNATCTCAGTTCGGNN

>pTOPO-514-M15-colony4-M13F_C06.ab1
NNNNNNNNNNNNGGGCGATTGGGCCCTCTAGATGCATGCTCGAGCGGCCGCCAGTGTGATGGATATCTGCAGAAT
TCGCCCTTATGGCCTATGAAAGCAAATCCTTGTCAATCGACTCTCCCCAGAGAAAATTGGGTAGGGGAAAGATCGAG
ATTAAGCGGATCGAAAACACAACGAATCGTCAAGTGACCTTCTGCAAGAGGCGCAATGGGTTGCTCAAGAAGGCCT
ATGAACTCTCTGTGCTCTGTGATGCAGAGGTTGCTCTCATAGTCTTCTCTAACCGTGGCCGCCTCTATGAGTATGCCAA
CAATAGAAGGGCGAATTCCAGCACACTGGCGGCCGTTACTAGTGGATCCGAGCTCGGTACCAAGCTTGATGCATAGC
TTGAGTATTCTATAGTGTCACCTAAATAGCTTGGCGTAATCATGGTCATAGCTGTTTCCTGTGTGAAATTGTTATCCGCT
CACAATTCCACACAACATACGAGCCGGAAGCATAAAGTGTAAAGCCTGGGGTGCCTAATGAGTGAGCTAACTCACAT
TAATTGCGTTGCGCTCACTGCCCGCTTTCCAGTCGGGAAACCTGTCGTGCCAGCTGCATTAATGAATCGGCCAACGCG
CGGGGAGAGGCGGTTTGCGTATTGGGCGCTCTTCCGCTTCCTCGCTCACTGACTCGCTGCGCTCGGTCGTTCGGCTG
CGGCGAGCGGTATCAGCTCACTCAAAGGCGGTAATACGGTTATCCACAGAATCAGGGGATAACGCAGGAAAGAACA
TGTGAGCAAAAGGCCAGCAAAAGGNCAGGAACCGTAAAAAGGNCGCGTTGCTGGCGTTTTTCCATAGGCTCCGCC
CCCCTGACGAGCATCACAAAAATCGACGCTCAAGTCAGAGNGGCGAAACCCGACAGGACTATAAAGATACCAGGCG
TTTCCCCTGGAAGCTCCCTCGTGCGCTCTCCTGTTCCGACCCTGCNGCTTACNGATACCTGTCGCTTNNTCCNTNGN
ANCGNGGCGCTTNCTCNTAGCTCNNGCTGTAGNATCTCAGTCGNGTAGTCNNCNNNNCAGCTGGGNNNNNNGC
ACGAN

MADS221

>pTOPO-514-M221-colony1-M13F_C03.ab1
NNNNNNNNNNNAGGGCGATTGGGCCCTCTAGATGCATGCTCGAGCGGCCGCCAGTGTGATGGATATCTGCAGAAT
TCGCCCTTATGGCCAATGAAAACAAATCCTTGTCAATCGACTCTCCCCAGAGAAAATTGGGTAGGGGAAAGATCGAG
ATCAAGCGGATCGAAAACACGACCAATCGTCAAGTGACCTTCTGCAAGAGGCGCAATGGGTTGCTCAAGAAGGCCT
ATGAACTCTCTGTGCTCTGTGATGCAGAGGTTGCTCTCATAGTCTTCTCTAACCGTGGCCGCCTCTATGAGTATGCCAA
CAACAGAAGGGCGAATTCCAGCACACTGGCGGCCGTTACTAGTGGATCCGAGCTCGGTACCAAGCTTGATGCATAG
CTTGAGTATTCTATAGTGTCACCTAAATAGCTTGGCGTAATCATGGTCATAGCTGTTTCCTGTGTGAAATTGTTATCCGC
TCACAATTCCACACAACATACGAGCCGGAAGCATAAAGTGTAAAGCCTGGGGTGCCTAATGAGTGAGCTAACTCACA
TTAATTGCGTTGCGCTCACTGCCCGCTTTCCAGTCGGGAAACCTGTCGTGCCAGCTGCATTAATGAATCGGCCAACGC
GCGGGGAGAGGCGGTTTGCGTATTGGGCGCTCTTCCGCTTCCTCGCTCACTGACTCGCTGCGCTCGGTCGTTCGGCT
GCGGCGAGCGGTATCAGCTCACTCAAAGGCGGTAATACGGTTATCCACAGAATCAGGGGATAACGCAGGNNNAACA
TGTGAGCAAAAGGCCAGCAAAAGGCCAGGAACCGTAAAAAGGNCGCGTTGCTGGCGTTTTTCCATAGGCTCCGCC

CCCCTGACGAGCATCACAAAAATCGACGCTCAAGTCAGANGTGGCGAAACCCGACAGGACTATAAAGATACCAGGC
GTTTCCCCCTGGAAGCTCCCTCGTGCGCTCTCCTGTTCCGACCCTGCCGCTTACCGGATACCTGTCCGCCTTTCTCCCT
TCGGGAAGCGTGGCGCTTTCTCATAGCTCACGCTGTNGNATCTCAGTTCGGNGTNNNCGTTCN

>pTOPO_MADS221_514_colony_4-M13F_C09.ab1

NNNNNNNNNGGGCGATTGGGCCCTCTAGATGCATGCTCGAGCGGCCGCCAGTGTGATGGATATCTGCAGAATTCG
CCCTTATGGCCAATGAAAACAAATCCTTGTCAATCGACTCTCCCCAGAGAAAATTGGGTAGGGGAAAGATCGAGATC
AAGAAGGCCTATGAACTCTCTGTGCTCTGTGATGCAGAGGTTGCTCTCATAGTCTTCTCTAACCGTGGCCGCCTCTATG
AGTATGCCAACAACAGAAGGGCGAATTCCAGCACACTGGCGGCCGTTACTAGTGGATCCGAGCTCGGTACCAAGCT
TGATGCATAGCTTGAGTATTCTATAGTGTCACCTAAATAGCTTGGCGTAATCATGGTCATAGCTGTTTCCTGTGTGAAAT
TGTTATCCGCTCACAATTCCACACAACATACGAGCCGGAAGCATAAAGTGTAAAGCCTGGGGTGCCTAATGAGTGAG
CTAACTCACATTAATTGCGTTGCGCTCACTGCCCGCTTTCCAGTCGGGAAACCTGTCGTGCCAGCTGCATTAATGAATC
GGCCAACGCGCGGGGAGAGGCGGTTTGCGTATTGGGCGCTCTTCCGCTTCCTCGCTCACTGACTCGCTGCGCTCGG
TCGTTCGGCTGCGGCGAGCGGTATCAGCTCACTCAAAGGCGGTAATACGGTTATCCACAGAATCNGGGGATAACGCA
NGAAAGAACATGTGAGCAAAANGCCAGCAAAAGGCCAGGAACCGTAAAAANNCCNNGTTTGCTGGCGTTTTTCC
ATAGGCTCCGCCCCCCNGACGAGCATCANAAAATCGACGCTCAGTCANNNNGNNAAACCCGACNGNCTATAAANN
TACCNNNNTTCCCCCNNNNNCNNNNN

Event 515

MADS15

>pTOPO-515-M15-colony4-M13F_A05.ab1
NNNNNANTNNNNNGGGCGATTGGGCCCTCTAGATGCATGCTCGAGCGGCCGCCAGTGTGATGGATATCTGCAGAA
TTCGCCCTTATGGCCTATGAAAGCAAATCCTTGTCCTTGGACTCTCCCCAGAGAAAATTGGGTAGGGGAAAGATCGA
GATTAAGCGGATCGAAAACACAACGAATCGTCAAGTGACCTTCTGCAAGAGGCGCAATGGGTTGCTCAAGAAGGCC
TATGAACTCTCTGTGCTCTGTGATGCAGAGGTTGCTCTCATAGTCTTCTCTAACCGTGGCCGCCTCTATGAGTATGCCA
ACAATAGAAGGGCGAATTCCAGCACACTGGCGGCCGTTACTAGTGGATCCGAGCTCGGTACCAAGCTTGATGCATAG
CTTGAGTATTCTATAGTGTCACCTAAATAGCTTGGCGTAATCATGGTCATAGCTGTTTCCTGTGTGAAATTGTTATCCGC
TCACAATTCCACACAACATACGAGCCGGAAGCATAAAGTGTAAAGCCTGGGGTGCCTAATGAGTGAGCTAACTCACA
TTAATTGCGTTGCGCTCACTGCCCGCTTTCCAGTCGGGAAACCTGTCGTGCCAGCTGCATTAATGAATCGGCCAACGC
GCGGGGAGAGGCGGTTTGCGTATTGGGCGCTCTTCCGCTTCCTCGCTCACTGACTCGCTGCGCTCGGTCGTTCGGCT
GCGGCGAGCGGTATCAGCTCACTCAAAGGCGGTAATACGGTTATCCACAGAATCAGGGGATAACGCAGGAAAGAAC
ATGTGAGCAAAAGGCCAGCAAAAGGCCAGGAACCGTAAAAAGGCCGCGTTGCTGGCGTTTTTCCATANGCTCCGCC
CCCCTGACGAGCATCACAAAAATCGACGCTCAAGTCAGAGGTGGCGAAACCCGACAGGACTATAAAGATACCAGGC
GTTTCCCCCTGGAAGCTCCCTCGTGCGCTCTCCTGT
TCCGACCCTGCCGCTTACCGGATACCTGTCCGCCTTTCTCCCTTCGGGAAGCGNNGCGCTTTCTCAN

>pTOPO-515-M15-colony7-M13F_B05.ab1
NNNNNNNNNNNNGGGCGATTGGGCCCTCTAGATGCATGCTCGAGCGGCCGCCAGTGTGATGGATATCTGCAGAAT
TCGCCCTTATGGCCTATGAAAGCAAATCCTTGTCCTTGGACTCTCCCCAGAGAAAATTGGGTAGGGGAAAGATCGAG
ATTAAGCGGATCGAAAACACAACGAATCGTCAAGTGACCTTCTGCAAGAGGCGCAATGGGTTGCTCAAGAAGGCCT
ATGAACTCTCTGTGCTCTGTGATGCAGAGGTTGCTCTCATAGTCTTCTCTAACCGTGGCCGCCTCTATGAGTATGCCAA
CAATAGAAGGGCGAATTCCAGCACACTGGCGGCCGTTACTAGTGGATCCGAGCTCGGTACCAAGCTTGATGCATAGC
TTGAGTATTCTATAGTGTCACCTAAATAGCTTGGCGTAATCATGGTCATAGCTGTTTCCTGTGTGAAATTGTTATCCGCT

CACAATTCCACACAACATACGAGCCGGAAGCATAAAGTGTAAAGCCTGGGGTGCCTAATGAGTGAGCTAACTCACAT
TAATTGCGTTGCGCTCACTGCCCGCTTTCCAGTCGGGAAACCTGTCGTGCCAGCTGCATTAATGAATCGGCCAACGCG
CGGGGAGAGGCGGTTTGCGTATTGGGCGCTCTTCCGCTTCCTCGCTCACTGACTCGCTGCGCTCGGTCGTTCGGCTG
CGGCGAGCGGTATCAGCTCACTCNAAGGCGGTAATACGGTTATCCACAGAATCANGGGATAACGCANGAAAGAACA
TGTGAGCAAAAGGCCAGCAAAAGGNCAGGAACCGTAAAAAGGCCGCGTTGCTGGCGTTTTTCCATANGCTCCGCC
CCCCTGACGAGCATCACAAAAATCGACGCTCAAGTCAGAGGTGGCGANACCCGACAGGACTATAAAGATACCANNC
GTTTCCCCTGNAAGCTCCCTCGTGCGCTCTCCTGNTCCGACCCTGCCGCTTACCGGANACCTGTCCGCTNTCTCCNTC
GGGAANCGTGGCGCTTTCTCATAGCTNNANNCTGNAGGNATNTNNGTTCGGNNGTANGNNN

MADS221

>pTOPO-515-M221-colony4-M13F_F05.ab1
GNNNNNNNNNNNAGGGCGATTGGGCCCTCTAGATGCATGCTCGAGCGGCCGCCAGTGTGATGGATATCTGCAGAA
TTCGCCCTTATGGCCAATGAAAACAAATCCTTGTCAATCGACTCTCCCCAGAGAAAATTGGGTAGGGGAAAGATCGA
GATCAAGCGGATCGAAAACACGACCAATCGTCAAGTGACCTTCTGCAAGAGGCGCAATGGGTTGCTCAAGAAGGCC
TATGAACTCTCTGTGCTCTGTGATGCAGAGGTTGCTCTCATAGTCTTCTCTAACCGTGGCCGCCTCTATGAGTATGCCA
ACAACAGAAGGGCGAATTCCAGCACACTGGCGGCCGTTACTAGTGGATCCGAGCTCGGTACCAAGCTTGATGCATA
GCTTGAGTATTCTATAGTGTCACCTAAATAGCTTGGCGTAATCATGGTCATAGCTGTTTCCTGTGTGAAATTGTTATCCG
CTCACAATTCCACACAACATACGAGCCGGAAGCATAAAGTGTAAAGCCTGGGGTGCCTAATGAGTGAGCTAACTCAC
ATTAATTGCGTTGCGCTCACTGCCCGCTTTCCAGTCGGGAAACCTGTCGTGCCAGCTGCATTAATGAATCGGCCAACG
CGCGGGGAGAGGCGGTTTGCGTATTGGGCGCTCTTCCGCTTCCTCGCTCACTGACTCGCTGCGCTCGGTCGTTCGGC
TGCGGCGAGCGGTATCAGCTCACTCAAAGGCGGTAATACGGTTATCCACAGAATCAGGGGATAACGCAGGNNNAAC
ATGTGAGCAAAAGGCCAGCAAAAGGCCAGGAACCGTAAAANNNNNNNNGNNNTNGGCGTTTTTCCATAGGCTCC
GCCCCCCTGACGAGCATCACAAAAATCGACGCTCAAGTCAGAGGTGGCGAAACCCGACAGGACTATAAAGATACCA
GGCGTTTCCCCCTGGAAGCTCCCTCGTGCGCTCTCCTGTTCCGACCCTGCCGCTTACCGGATACCTGTCGCTTTCTCCT
TCGGGAGCGTGNGCTTTCTCNTAGCTCACGCTGNNNNATCTCNGTTCGGNGTANNCGTNNNNNNNNCTGGGCTG
NGTGNACGAAN

>pTOPO_MADS221_515_colony_7-M13F_G08.ab1

NNNNNNNNNNNNGNNNATTGGGCCCTCTAGATGCATGCTCGAGCGGCCGCCAGTGTGATGGATATCTGCAGAATT
CGCCCTTTTCATTTGTTTCTGCAAGTTAATTTCAAACTTTTTGTTGGTGATCAAGAATATAGTAATTGAAGTTCTGGGG
TTTTTGTTTTATTTAAAAAGGTGCTTAATTGATTGATCTCTTTGTTCTTATTGATTCAGCTTGCAACTATGGCCAATGAA
AACAAATCCTTGTCAATCGACTCTCCCCAGAGAAAATTGGGTAGGGGAAAGATCGAGATCAAGCGGATCGAAAACA
CGACCAATCGTCAAGTGACCTTCTGCAAGAGGCGCAATGGGTTGCTCAAGAAGGCCTATGAACTCTCTGTGCTCTGT
GATGCAGAGGTTGCTCTCATAGTCTTCTCTAACCGTGGCCGCCTCTATGAGTATGCCAACAACAGGTAATAATATTATT
GCTTCAATTTTCTTGCTAAGGGCGAATTCCAGCACACTGGCGGCCGTTACTAGTGGATCCGAGCTCGGTACCAAGCT
TGATGCATAGCTTGAGTATTCTATAGTGTCACCTAAATAGCTTGGCGTAATCATGGTCATAGCTGTTTCCTGTGTGAAAT
TGTTATCCGCTCACAATTCCACACAACATACGAGCCGGAAGCATAAAGTGTAAAGCCTGGGGTGCCTAATGAGTGAG
CTAACTCACATTAATTGCGTTGCGCTCACTGCCCGCTTTCCAGTCGGGAAACCTGTCGTGCCAGCTGCATTAATGAATC
GGCCAACGCGCGGGGAGAGGCGGTTTGCGTATTGGGCGCTCTTCCGCTTCCTCGCTCACTGACTCGCTGCGCTCGG
TCGTTCGGCTGCGGCGAGCGGTATCAGCTCACTCAAAGGNGGTAATACGGTTATCCACAGAATCNNGGGATAACGC
NGAAAGAACATGTGAGCAAAAGGCCANN

Event 516

MADS15

>pTOPO-MADS15-516-colony-1-M13F_G08.ab1

NNGNNNNNNNNNNNNGGGCGATTGGGCCCTCTAGATGCATGCTCGAGCGGCCGCCAGTGTGATGGATATCTGCAG
AATTCGCCCTTATGGCCTATGAAAGCAAATCCTTGTCCTTGGACTCTCCCCAGAGAAAATTGGGTAGGGGAAAGATC
GAGATTAAGCGGATCGAAAACACAACGAATCGTCAAGTGACCTTCTGCAAGAGGCGCAATGGGTTGCTCAAGAAG
GCCTATGAACTCTCTGTGCTCTGTGATGCAGAGGTTGCTCTCATAGTCTTCTCTAACCGTGGCCGCCTCTATGAGTATG
CCAACAATAGAAGGGCGAATTCCAGCACACTGGCGGCCGTTACTAGTGGATCCGAGCTCGGTACCAAGCTTGATGCA
TAGCTTGAGTATTCTATAGTGTCACCTAAATAGCTTGGCGTAATCATGGTCATAGCTGTTTCCTGTGTGAAATTGTTATC
CGCTCACAATTCCACACAACATACGAGCCGGAAGCATAAAGTGTAAAGCCTGGGGTGCCTAATGAGTGAGCTAACTC
ACATTAATTGCGTTGCGCTCACTGCCCGCTTTCCAGTCGGGAAACCTGTCGTGCCAGCTGCATTAATGAATCGGCCAA
CGCGCGGGGAGAGGCGGTTTGCGTATTGGGCGCTCTTCCGCTTCCTCGCTCACTGACTCGCTGCGCTCGGTCGTTCG
GCTGCGGCGAGCGGTATCAGCTCACTCAAAGGCGGTAATACGGTTATCCACAGAATCAGGGGATAACGCAGGAAAG
AACATGTGAGCAAAAGGCCAGCAAAAGGCCAGGAACCGTAAAAAGGCCGCGTTGCTGGCGTTTTTCCATAGGCTCC
GCCCCCCTGACGAGCATCACAAAAATCGACGCTCAAGTCAGANGTGGCGAAACCCGACAGGACTATAAAGATACCA
GGCGTTTCCCCCTGGAAGCTCCCTCGTGCGCTCTCCTGTTCCGACCCTGCCGCTTACCGGATACCTGTCCGCCTTTCTC
CCTTCGGGAAGCGNNGNGCTTTCTCATAGCTCACGCTGTNNNATCTCANNNGGNGTNNNCNNNGNTCNAGCTGG
GCTGNGTGCACNACCCCCNNNANCCCGACGCTGCGCCTTATCCGGNAACTATCGTCTTGANTCCAACCCG

>pTOPO-MADS15-516-colony-4-M13F_H08.ab1

NNNNTNNNNNNNNNGGGCGATTGGGCCCTCTAGATGCATGCTCGAGCGGCCGCCAGTGTGATGGATATCTGCAGAA
TTCGCCCTTATGGCCTATGAAAGCAAATCCTTGTCCTTGGACTCTCCCCAGAGAAAATTGGGTAGGGGAAAGATCGA
GATTAAGCGGATCGAAAACACAACGAATCGTCAAGTGACCTTCTGCAAGAGGCGCAATGGGTTGCTCAAGAAGGCC
TATGAACTCTCTGTGCTCTGTGATGCAGAGGTTGCTCTCATAGTCTTCTCTAACCGTGGCCGCCTCTATGAGTATGCCA
ACAATAGAAGGGCGAATTCCAGCACACTGGCGGCCGTTACTAGTGGATCCGAGCTCGGTACCAAGCTTGATGCATAG
CTTGAGTATTCTATAGTGTCACCTAAATAGCTTGGCGTAATCATGGTCATAGCTGTTTCCTGTGTGAAATTGTTATCCGC
TCACAATTCCACACAACATACGAGCCGGAAGCATAAAGTGTAAAGCCTGGGGTGCCTAATGAGTGAGCTAACTCACA
TTAATTGCGTTGCGCTCACTGCCCGCTTTCCAGTCGGGAAACCTGTCGTGCCAGCTGCATTAATGAATCGGCCAACGC
GCGGGGAGAGGCGGTTTGCGTATTGGGCGCTCTTCCGCTTCCTCGCTCACTGACTCGCTGCGCTCGGTCGTTCGGCT
GCGGCGAGCGGTATCAGCTCACTCAAAGGCGGTAATACGGTTATCCACAGAATCAGGGGATAACGCAGGAAAGAAC
ATGTGAGCAAAAGGCCAGCAAAAGGCCAGGAACCGTAAAAAGGCCGCGTTGCTGGCGTTTTTCCATANGCTCCGCC
CCCCTGACGAGCATCACAAAAATCGACGCTCAAGTCAGANGTGGCGAAACCCGACAGGACTATAAAGATACCAGGC
GTTTCCCCCNGGNAGCTCCCTCGTGCGCTCTCCTGTTCCGACCCTGCCGCTTACCGGATACCTGTCCGCCTTTCTCCCT
TCGGGAAGCGNGNNCTTTCTCATAGCTCACGCTGTAGGTATCTCAGTTCGGNGTAGGTCGTTCGNNNCNAGCTGGG
NNNNNNGNNNNAANCCCCNNNANCCCGNN

MADS221

>pTOPO_MADS221_516_colony_5a-M13F_E09.ab1

NNNNNNNNNGGGCGATTGGGCCCTCTAGATGCATGCTCGAGCGGCCGCCAGTGTGATGGATATCTGCAGAATTCG
CCCTTATGGCCAATGAAAACAAATCCTTGTCAATCGACTCTCCCCAGAGAAAATTGGGTAGGGGAAAGATCGAGATC

AAGCGGATCGAAAACACGACCAATCGTCAAGTGACCTTCTAAGAGGCGCAATGGGTTGCTCAAGAAGGCCTATGAA
CTCTCTGTGCTCTGTGATGCAGAGGTTGCTCTCATAGTCTTCTCTAACCGTGGCCGCCTCTATGAGTATGCCAACAACA
GAAGGGCGAATTCCAGCACACTGGCGGCCGTTACTAGTGGATCCGAGCTCGGTACCAAGCTTGATGCATAGCTTGA
GTATTCTATAGTGTCACCTAAATAGCTTGGCGTAATCATGGTCATAGCTGTTTCCTGTGTGAAATTGTTATCCGCTCACA
ATTCCACACAACATACGAGCCGGAAGCATAAAGTGTAAAGCCTGGGGTGCCTAATGAGTGAGCTAACTCACATTAATT
GCGTTGCGCTCACTGCCCGCTTTCCAGTCGGGAAACCTGTCGTGCCAGCTGCATTAATGAATCGGCCAACGCGCGGG
GAGAGGCGGTTTGCGTATTGGGCGCTCTTCCGCTTCCTCGCTCACTGACTCGCTGCGCTCGGTCGTTCGGCTGCGGC
GAGCGGTATCAGCTCACTCAAAGGCGGTAATACNGTTATCCACAGAATCNGGGGATAACGCNNNANGANATGTGA
GCAAAANNCAGCAAAGNCNGANNTAAAANCNNNGTTGCTGGCNTTTTTCATAGNCTCGCCCCCNNNANNAGCAT
CACAAAAAATCGACGCTCAAGN

>pTOPO_MADS221_516_colony_5-M13F_D10.ab1

NNNNNNNNNGGGCGATTGGGCCCTCTAGATGCATGCTCGAGCGGCCGCCAGTGTGATGGATATCTGCAGAATTCG
CCCTTATGGCCAATGAAAACAAATCCTTGTCAATCGACTCTCCCCAGAGAAAATTGGGTAGGGGAAAGATCGAGATC
AAGCGGATCGAAAACACGACCAATCGTCAAGTGACCTTCTGCAAGAGGCGCAATGGGTTGCTCAGGCCTGTGAACT
CTCTGTGCTCTGTGATGCANAGGTTGCTCTCATACTCTTCTCTAACCGTGGCCGCCTCTATGAATATGCCAACAACAAA
AGGGCNAATTCAATCACACTGGCGGCCGTTACTAATGGATCCAAGCTCGGTACCAACCTTGATGCATACCTTGAGTAT
TCAATAGTGTCAGCCAAATAGCTTGGCGGAATCATGGTCATACCTGTTTCCTGTGTGAAATTGTTATCCGCTCACAATT
CCACACAACATACAAACCGGAAGCATAAAGTGTAAAGCCTGNGGTGCCTAANGAATGAGCTAACTCACATTAATTGC
NTTNCGCTCACTGCCCGCTTTCCATTCNGGAAAGCAGTCNNNCCAGCTGCATTAATGAATCNNCCAACGCGCGNGG
AGAGGCAGTTTGNNTATTGNGNGCNCTTNCGCTTCCTCNCTCACTGACTCACTGCGCTCGGTCGTTCGGCTGCCNG
CNAGCGATATCAACTCACTCANANGCGGTAATACAGTTATCCACANAATCANNNNNATNNCTNACGNNNGANCNN
GTGAGNNAANNNNNNNGCAANNNGNNNGCANCNNNNNNNNNNNNNNNTTNGCTNGCNNTNNTTNCNNTN
NNNNNNNNNNCCCCCCCTNNANNNANCNNNNNNANNAAAAANNNNAN

Event 517

<u>MADS15</u>

>pTOPO-517-M15-colony-1-M13F_A05.ab1
NNNNNNNNNNNNNNGGCGATTGGGCCCTCTAGATGCATGCTCGAGCGGCCGCCAGTGTGATGGATATCTGCAGAAT
TCGCCCTTCTATTGTTGGCATACTCATAGAGGCGGCCACGGTTAGAGAAGACTATGAGAGCAACCTCTGCATCACAGA
GCACAGAGAGTTCATAGGCCTTCTTTGAGCAACCCATTGCGCCTCTTGCAGAAGGTCACTTGACGATTCGTTGTGTTT
TCGATCCGCTTAATCTCGATCTTTCCCCTACCCAATTTTCTCTGGGGAGAGTCCAAGGACAAGGATTTGCTTTCATAGG
CCATAAGGGCGAATTCCAGCACACTGGCGGCCGTTACTAGTGGATCCGAGCTCGGTACCAAGCTTGATGCATAGCTT
GAGTATTCTATAGTGTCACCTAAATAGCTTGGCGTAATCATGGTCATAGCTGTTTCCTGTGTGAAATTGTTATCCGCTCA
CAATTCCACACAACATACGAGCCGGAAGCATAAAGTGTAAAGCCTGGGGTGCCTAATGAGTGAGCTAACTCACATTA
ATTGCGTTGCGCTCACTGCCCGCTTTCCAGTCGGGAAACCTGTCGTGCCAGCTGCATTAATGAATCGGCCAACGCGC
GGGGAGAGGCGGTTTGCGTATTGGGCGCTCTTCCGCTTCCTCGCTCACTGACTCGCTGCGCTCGGTCGTTCGGCTGC
GGCGAGCGGTATCAGCTCACTCAAAGGCGGTAATACGGTTATCCACAGAATCAGGGGATAACGCAGGAAAGAACAT
GTGAGCAAAAGGCCAGCAAAAGGCCAGGAACCGTAAAAAGGCCGCGTTGCTGGCGNNNTTCCATAGGCTCCGCCC

CCCTGACGAGCATCACAAAAATCGACGCTCAAGTCAGAGGTGGCGAAACCCGACAGGACTATAAAGATACCAGGCG
TTTCCCCCTGGAAGCTCCCTCGTGCGCTCTCCTGN

>pTOPO-517-M15-colony-7-M13F_B05.ab1
NNNNNNNNNNNNNNNGGCGATTGGGCCCTCTAGATGCATGCTCGAGCGGCCGCCAGTGTGATGGATATCTGCAGA
ATTCGCCCTTATGGCCTATGAAAGCAAATCCTTGTCAATCGACTCTCCCCAGAGAAGGCCTATGAACTCTCTGTGCTCT
GTGATGCAGAGGTTGCTCTCATAGTCTTCTCTAACCGTGGCCGCCTCTATGAGTATGCCAACAATAGAAGGGCGAATT
CCAGCACACTGGCGGCCGTTACTAGTGGATCCGAGCTCGGTACCAAGCTTGATGCATAGCTTGAGTATTCTATAGTGT
CACCTAAATAGCTTGGCGTAATCATGGTCATAGCTGTTTCCTGTGTGAAATTGTTATCCGCTCACAATTCCACACAACAT
ACGAGCCGGAAGCATAAAGTGTAAAGCCTGGGGTGCCTAATGAGTGAGCTAACTCACATTAATTGCGTTGCGCTCAC
TGCCCGCTTTCCAGTCGGGAAACCTGTCGTGCCAGCTGCATTAATGAATCGGCCAACGCGCGGGGAGAGGCGGTTT
GCGTATTGGGCGCTCTTCCGCTTCCTCGCTCACTGACTCGCTGCGCTCGGTCGTTCGGCTGCGGCGAGCGGTATCAG
CTCACTCAAAGGCGGTAATACGGTTATCCACAGAATCAGGGGATAACGCAGGAAAGAACATGTGAGCAAAAGGCCA
GCAAAAGGCCAGGAACCGTAAAAAGGCCGCGTTGCTGGCGTTTTTCCATAGGCTCCGCCCCCCTGACGAGCATCAC
AAAAATCGACGCTCAAGTCAGAGGTGGCGAAACCCGACAGGACTATAAAGATACCAGGCGTTTCCCCCTNNAGCTC
CCTCGTGCGCTCTCCTGTTCCGACCCTGCCGCTTACCGGATACCTGTCCGCCTTTCTCCCTTCGGGAAGCGTGGCGCT
TTCTCATAGCTCACGCTGTAGGNATCTCAGTTCGGNGTAGGNCGTTCGCTCCAAG

MADS221

>pTOPO-517-M221-colony4-M13F_E05.ab1
NNNNNNNNNNTNNNNNNNNNNTNNNGCCCNCTNNATGNTGNANNAGCGGTCGCCTNGTGATGGATATCTGCA
GAATTCCCCCTTCTGTTGTTGGCATACTCATACAGGCGGCCACGGTTAGAGAAGACTATGACAGCAACCTCTGCATCA
CAGAGCACAGAGAGTTCATAGGCCTTCTCTGGGGAGAGTCGATTGACAAGGATTTGTTTTCATTGGCCATAAGGGC
GAATTCCAGCACACTGGCGGCCGTTACTAGTGGATCCGAGCTCGGTACCAAGCTTGATGCATAGCTTGAGTATTCTAT
AGTGTCACCTAAATAGCTTGGCGTAATCATGGTCATAGCTGTTTCCTGTGTGAAATTGTTATCCGCTCACAATTCCACAC
AACATACGAGCCGGAAGCATAAAGTGTAAAGCCTGGGGTGCCTAATGAGTGAGCTAACTCACATTAATTGCGTTGCG
CTCACTGCCCGCTTTCCAGTCGGGAAACCTGTCGTGCCAGCTGCATTAATGAATCGGCCAACGCGCGGGGAGAGGC
GGTTTGCGTATTGGGCGCTCTTCCGCTTCCTCGCTCACTGACTCGCTGCGCTCGGTCGTTCGGCTGCGGCGAGCGGT
ATCAGCTCACTCAAAGGCGGTAATACGGTTATCCACAGAATCAGGGGATAACGCAGGAAAGAACATGTGAGCAAAA
GGCCAGCAAAAGGCCAGGAACCGTAAAAAGGCCGCGTTGCTGGCGTTTTTCCATANGCTCCGCCCCCCTGACGAGC
ATCACAAAAATCGACGCTCAAGTCAGANGTGGCGAAACCGACAGGACTATAAAGATACCAGGCGTTTCCCCCTGNA
AGCTCCCTCGTGCGCTCTCCTGTTCCGACCCTGCCGCTTACCGGATACCTGTCCGCCTTTCTCNCTTCGGGAANCGTG
GCGCTTTCTCATAGCTCACGCTGTANGTATCTCNTTNCGNNGTGAGTCNTNGNTNNNANCTGGNCNNGGGCACAA
NCCCCCNTTNAGNCAAACGNTGCTNNTNATCCGGNAATATNGNCTTGANCCAACCCNGGNAAANNNNACTNTNN
NNNNNNNNNAGNCNNN

>pTOPO_MADS221_517_colony_3A-M13F_D09.ab1

NNNNNNNNNNNNNNGGCGATTGGGCCTCTAGATGCATGCTCGAGCGGCCGCCAGTGTGATGGATATCTGCAGAATT
CGCCCTTTTCATTTGTTTCTGCAAGTTAATTTCAAACTTTTTGTTGGTGATCAAGAATATAGTAATTGAAGTTCTGGGG
TTTTTGTTTTATTTAAAAAGGTGCTTAATTGATTGATCTCTTTGTTCTTATTGATTCAGCTTGCAACTATGGCCAATGAA
AACAAATCCTTGTCAATCGACTCTCCCCAGAGAAGGCCTATGAACTCTCTGTGCTCTGTGATGCAGAGGTTGCTCTCA
TAGTCTTCTCTAACCGTGGCCGCCTCTATGAGTATGCCAACAACAGGTAATAATATTATTGCTTCAATTTTCTTGCTAAG
GGCGAATTCCAGCACACTGGCGGCCGTTACTAGTGGATCCGAGCTCGGTACCAAGCTTGATGCATAGCTTGAGTATT
CTATAGTGTCACCTAAATAGCTTGGCGTAATCATGGTCATAGCTGTTTCCTGTGTGAAATTGTTATCCGCTCACAATTCC

ACACAACATACGAGCCGGAAGCATAAAGTGTAAAGCCTGGGGTGCCTAATGAGTGAGCTAACTCACATTAATTGCGT
TGCGCTCACTGCCCGCTTTCCAGTCGGGAAACCTGTCGTGCCAGCTGCATTAATGAATCGGCCAACGCGCGGGGAG
AGGCGGTTTGCGTATTGGGCGCTCTTCCGCTTCCTCGCTCACTGACTCGCTGCGCTCGGTCGTTCGGCTGCGGCGAG
CGGTATCAGCTCACTCAAAGGCGGTAATACGGTTATCCACAGAATCANGGGATAACGCANGAAAGAACATGTGAGC
AAANNGCCAGCAAAAGGCCAGGAACCGTAAAAGGCCNCGTTGCTGGCGTTTTCCATAGGCTCGNCCCCNCTGACN
AGCATCACAAAAATCGACNCTCANGTCANAGNGGCNAAACCCGANN

Event 518

<u>MADS15</u>

>pTOPO_MADS515_518_colony_4-M13F_C12.ab1
NNNNNTNNNNNNNNNNGCGATTGGGCCCTCTAGATGCATGCTCGAGCGGCCGCCAGTGTGATGGATATCTGCAG
AATTCGCCCTTCTATTGTTGGCATACTCATAGAGGCGGCCACGGTTAGAGAAGACTATGAGAGCAACCTCTGCATCAC
AGAGCACAGAGAGTTCATAGGCCTTCTTGAGCAACCCATTGCGCCTCTTGCAGAAGGTCACTTGACGATTCGTTGTG
TTTTCGATCCGCTTAATCTCGATCTTTCCCCTACCCAATTTTCTCTGGGGAGAGTCCAAGGACAAGGATTTGCTTTCAT
AGGCCATAAGGGCGAATTCCAGCACACTGGCGGCCGTTACTAGTGGATCCGAGCTCGGTACCAAGCTTGATGCATAG
CTTGAGTATTCTATAGTGTCACCTAAATAGCTTGGCGTAATCATGGTCATAGCTGTTTCCTGTGTGAAATTGTTATCCGC
TCACAATTCCACACAACATACGAGCCGGAAGCATAAAGTGTAAAGCCTGGGGTGCCTAATGAGTGAGCTAACTCACA
TTAATTGCGTTGCGCTCACTGCCCGCTTTCCAGTCGGGAAACCTGTCGTGCCAGCTGCATTAATGAATCGGCCAACGC
GCGGGGAGAGGCGGTTTGCGTATTGGGCGCTCTTCCGCTTCCTCGCTCACTGACTCGCTGCGCTCGGTCGTTCGGCT
GCGGCGAGCGGTATCAGCTCACTCAAAGGCGGTAATACGGTTATCCACAGAATCNGGGGATAACGCNGGAAAGAAC
ATGTGAGCAAAAGGCNN

>pTOPO_MADS515_518_colony_6-M13F_D12.ab1

NNNNNNNNNNNNGGGCGATTGGGCCCTCTAGATGCATGCTCGAGCGGCCGCCAGTGTGATGGATATCTGCAGAAT
TCGCCCTTATGGCCTATGAAAGCAAATCCTTGTCCTTGGACTCTCCCCAGAGAAAATTGGGTAGGGGAAAGATCGAG
ATTAAGCGGATCGAAAACACAACGAATCGTCAAGTGACCTTCTGCAAGAGGCGCAATGGGTTGCTCAAGAAGGCCT
ATGAACTCTCTGTGCTCTGTGATGCAGAGGTTGCTCTCATAGTCTTCTCTAACCGTGGCCGCCTCTATGAGTATGCCAA
CAATAGAAGGGCGAATTCCAGCACACTGGCGGCCGTTACTAGTGGATCCGAGCTCGGTACCAAGCTTGATGCATAGC
TTGAGTATTCTATAGTGTCACCTAAATAGCTTGGCGTAATCATGGTCATAGCTGTTTCCTGTGTGAAATTGTTATCCGCT
CACAATTCCACACAACATACGAGCCGGAAGCATAAAGTGTAAAGCCTGGGGTGCCTAATGAGTGAGCTAACTCACAT
TAATTGCGTTGCGCTCACTGCCCGCTTTCCAGTCGGGAAACCTGTCGTGCCAGCTGCATTAATGAATCGGCCAACGCG
CGGGGAGAGGCGGTTTGCGTATTGGGCGCTCTTCCGCTTCCTCGCTCACTGACTCGCTGCGCTCGGTCGTTCGGCTG
CGGCGAGCGGTATCAGCTCACTCAAAGGCGGTAATACGGTTATCCACAGAATCNGGGGATAACGCNNNAAAGAACA
TGTGAGCAAAAGGNCANN

<u>MADS221</u>

>518-pTOPO-MADS221-colony-7-M13F_G02.ab1

NNNNNNNNNNNTNNGGCGATTGGGCCCTCTAGATGCATGCTCGAGCGGCCGCCAGTGTGATGGATATCTGCAGAA
TTCGCCCTTATGGCCAATGAAAACAAATCCTTGTCAATCGACTCTCCCCAGAGAAAATTGGGTAGGGGAAAGATCGA
GATCAAGCGGATCGAAAACACGACCAATCGTCAAGTGACCTTCTGCAAGAGGCGCAATGGGTTGCTCAAGAAGGCC
TATGAACTCTCTGTGCTCTGTGATGCAGAGGTTGCTCTCATAGTCTTCTCTAACCGTGGCCGCCTCTATGAGTATGCCA
ACAACAGAAGGGCGAATTCCAGCACACTGGCGGCCGTTACTAGTGGATCCGAGCTCGGTACCAAGCTTGATGCATA
GCTTGAGTATTCTATAGTGTCACCTAAATAGCTTGGCGTAATCATGGTCATAGCTGTTTCCTGTGTGAAATTGTTATCCG
CTCACAATTCCACACAACATACGAGCCGGAAGCATAAAGTGTAAAGCCTGGGGTGCCTAATGAGTGAGCTAACTCAC
ATTAATTGCGTTGCGCTCACTGCCCGCTTTCCAGTCGGGAAACCTGTCGTGCCAGCTGCATTAATGAATCGGCCAACG
CGCGGGGAGAGGCGGTTTGCGTATTGGGCGCTCTTCCGCTTCCTCGCTCACTGACTCGCTGCGCTCGGTCGTTCGGC
TGCGGCGAGCGGTATCAGCTCACTCAAAGGCGGTAATACGGTTATCCACAGAATCAGGGGATAACGCAGGAAAGAA
CATGTGAGCAAAAGGCCAGCAAAAGGCCAGGAACCGTAAAAAGGCCGCGTTGCTGGCGTTTTTCCATAGGCTCCGC
CCCCCTGACGAGCATCACAAAAATCGACGCTCAAGTCAGAGGTGGCGAAACCCGACAGGACTATAAAGATACCAGG
CGTTTCCCCCTGNAAGCTCCCTCGTGCGCTCTCCTGTTCCGACCCTGCCGCTTACCGGATACCTGTCCGCCTTTCTCCC
TTTCGGGAAGCGTGGCGCTTTCTCATAGCTCACGCTNNNNGGNNATNNNNNNNTTNNNNNNNNNNNGTCGTTC
GNNNNCAAGCTNN

>518-pTOPO-MADS221-colony-2-M13F_H02.ab1

NNNNNNNNNNNNNNGGCGATTGGGCCCTCTAGATGCATGCTCGAGCGGCCGCCAGTGTGATGGATATCTGCAGAAT
TCGCCCTTATGGCCAATGAAAACAAATCCTTGTCAATCGACTCTCCCCAGAGAAAATTGGGTAGGGGAAAGATCGAG
ATCAAGCGGATCGAAAACACGACCAATCGTCAAGTGACCTTCTGCAAGAGGCGCAATGGGTTGCTCAAGAAGGCCT
ATGAACTCTCTGTGCTCTGTGATGCAGAGGTTGCTCTCATAGTCTTCTCTAACCGTGGCCGCCTCTATGAGTATGCCAA
CAACAGAAGGGCGAATTCCAGCACACTGGCGGCCGTTACTAGTGGATCCGAGCTCGGTACCAAGCTTGATGCATAG
CTTGAGTATTCTATAGTGTCACCTAAATAGCTTGGCGTAATCATGGTCATAGCTGTTTCCTGTGTGAAATTGTTATCCGC
TCACAATTCCACACAACATACGAGCCGGAAGCATAAAGTGTAAAGCCTGGGGTGCCTAATGAGTGAGCTAACTCACA
TTAATTGCGTTGCGCTCACTGCCCGCTTTCCAGTCGGGAAACCTGTCGTGCCAGCTGCATTAATGAATCGGCCAACGC
GCGGGGAGAGGCGGTTTGCGTATTGGGCGCTCTTCCGCTTCCTCGCTCACTGACTCGCTGCGCTCGGTCGTTCGGCT
GCGGCGAGCGGTATCAGCTCACTCAAAGGCGGTAATACGGTTATCCACAGAATCAGGGGATAACGCAGGAAAGAAC
ATGTGAGCAAAAGGCCAGCAAAAGGCCAGGAACCGTAAAAAGGCCGCGTTGCTGGCGTTTTTCCATANGCTCCGCC
CCCCTGACGAGCATCACAAAAATCGACGCTCAAGTCAGANGTGGCGAAACCCGACAGGACTATAAAGATACCAGGC
GTTTCCCCCTGGAAGCTCCCTCGTGCGCTCTCCTGTTCCGANCCTGCCGCTTACCGGATACCTGTCNNCTTTCTCCCTT
CNGGAAGCGTGGCGCTTTCNNNATANCTCACGCTGNANGNN

Event 519

MADS15

>519-pTOPO-MADS15-colony-9-M13F_F06.ab1

NNNNNNNNNNNNNNNNGNNNANTTGGGCCNTCTAGATGCATGCTCGAGCGGCCGCCAGTGTGATGGATATCTGCA
GAATTCGCCCTTCTATTGTTGGCATACTCATAGAGGCGGCCACGGTTAGAGAAGACTATGAGAGCAACCTCTGCATCA
CAGAGCACAGAGAGTTCATAGGCCTTCTTGAGCAACCCATTGCGCCTCTTGCAGAAGGTCACTTGACGATTCGTTGT
GTTTTCGATCCGCTTAATCTCGATCTTTCCCCTACCCAATTTTCTCTGGGGAGAGTCCAAGGACAAGGATTTGCTTTCA
TAGGCCATAAGGGCGAATTCCAGCACACTGGCGGCCGTTACTAGTGGATCCGAGCTCGGTACCAAGCTTGATGCATA

GCTTGAGTATTCTATAGTGTCACCTAAATAGCTTGGCGTAATCATGGTCATAGCTGTTTCCTGTGTGAAATTGTTATCCG
CTCACAATTCCACACAACATACGAGCCGGAAGCATAAAGTGTAAAGCCTGGGGTGCCTAATGAGTGAGCTAACTCAC
ATTAATTGCGTTGCGCTCACTGCCCGCTTTCCAGTCGGGAAACCTGTCGTGCCAGCTGCATTAATGAATCGGCCAACG
CGCGGGGAGAGGCGGTTTGCGTATTGGGCGCTCTTCCGCTTCCTCGCTCACTGACTCGCTGCGCTCGGTCGTTCGGC
TGCGGCGAGCGGTATCAGCTCACTCAAAGGCGGTAATACGGTTATCCACAGAATCAGGGGATAACGCAGGAAAGAA
CATGTGAGCAAAAGGCCAGCAAAAGGCCAGGAACCGTAAAAAGGCCGCGTTGCTGGCGTTTTTCCATANGCTCCGC
CCCCCTGACGAGCATCACAAAAATCGACGCTCAAGTCAGANGTGGCGAAACCCGACAGGACTATAAAGATACCAGG
CGTTTCCCCCTGGAAGCTCCCTCGTGCGCTCTCCTGTTCCGACCCTGCCGCTTACCGGATACCTGTCCGCCTTTCTCCC
TTCGGGAAGCGTNNGCTTTCTCATAGCTCACGCTGNANNNATCTCANTTCNNNGTAGTCGTTCGCTNCANCTGGNT
GTGTGNACNANCCCCGNTCAGCCGACCGCTGCNCCTNNNNCGGNAANNNATCN

>519-pTOPO-MADS15-colony-10-M13F_G06.ab1

NNNNNNNNNNNNNNNNGGCGANTGGGCCNTCTAGATGCATGCTCGAGCGGCCGCCAGTGTGATGGATATCTGCA
GAATTCGCCCTTATGGCCTATGAAAGCAAATCCTTGTCCTTGGACTCTCCCCAGAGAAAATTGGGTAGGGGAAAGAT
CGAGATTAAGCGGATCGAAAACACAACGAATCGTCAAGTGACCTTCTGCAAGAGGCGCAATGGGTTGCTCAAGAAG
GCCTATGAACTCTCTGTGCTCTGTGATGCAGAGGTTGCTCTCATAGTCTTCTCTAACCGTGGCCGCCTCTATGAGTATG
CCAACAATAGAAGGGCGAATTCCAGCACACTGGCGGCCGTTACTAGTGGATCCGAGCTCGGTACCAAGCTTGATGCA
TAGCTTGAGTATTCTATAGTGTCACCTAAATAGCTTGGCGTAATCATGGTCATAGCTGTTTCCTGTGTGAAATTGTTATC
CGCTCACAATTCCACACAACATACGAGCCGGAAGCATAAAGTGTAAAGCCTGGGGTGCCTAATGAGTGAGCTAACTC
ACATTAATTGCGTTGCGCTCACTGCCCGCTTTCCAGTCGGGAAACCTGTCGTGCCAGCTGCATTAATGAATCGGCCAA
CGCGCGGGGAGAGGCGGTTTGCGTATTGGGCGCTCTTCCGCTTCCTCGCTCACTGACTCGCTGCGCTCGGTCGTTCG
GCTGCGGCGAGCGGTATCAGCTCACTCAAAGGCGGTAATACGGTTATCCACAGAATCAGGGGATAACGCAGGAAAG
AACATGTGAGCAAAAGGCCAGCAAAAGGCCAGGAACCGTAAAAAGGCCGCGTTGCTGGCGTTTTTCCATAGGCTCC
GCCCCCCTGACGAGCATCACAAAAATCGACGCTCAAGTCAGANGTGGCGAAACCCGACAGGACTATAAAGATACCA
GGCGTTTCCCCCTNNANGCTCCCTCGTGCGCTCTCCTGTTCCGACCCTGCCGCTTACCGGATACCTGTCCGCCTTTCT
CCCTTCNGGAAGCGTGNGCTTTCTCATAGCTCACGCTGNNNGTATNNNCANNNCGGGNGTAGGTCNNNGCTCCN
AGCTGGGNTGTGTGCACGAACCCCCCGTTCANCCCGACNGCTGCGNNNN

<u>MADS221</u>

>pTOPO-MADS21-519-colony-B-M13F_E07.ab1

NNNNNNNNNNNNNNGGGNNANNGGGCCCTCTAGATGCATGCTCGAGCGGCCGCCAGTGTGATGGATATCTGCAG
AATTCGCCCTTATGGCCAATGAAAACAAATCCTTGTCAATCGACTCTCCCCAGAGAAAAGAAGGCCTATGAACTCTCT
GTGCTCTGTGATGCAGAGGTTGCTCTCATAGTCTTCTCTAACCGTGGCCGCCTCTATGAGTATGCCAACAACAGAAGG
GCGAATTCCAGCACACTGGCGGCCGTTACTAGTGGATCCGAGCTCGGTACCAAGCTTGATGCATAGCTTGAGTATTCT
ATAGTGTCACCTAAATAGCTTGGCGTAATCATGGTCATAGCTGTTTCCTGTGTGAAATTGTTATCCGCTCACAATTCCAC
ACAACATACGAGCCGGAAGCATAAAGTGTAAAGCCTGGGGTGCCTAATGAGTGAGCTAACTCACATTAATTGCGTTG
CGCTCACTGCCCGCTTTCCAGTCGGGAAACCTGTCGTGCCAGCTGCATTAATGAATCGGCCAACGCGCGGGGAGAG
GCGGTTTGCGTATTGGGCGCTCTTCCGCTTCCTCGCTCACTGACTCGCTGCGCTCGGTCGTTCGGCTGCGGCGAGCG
GTATCAGCTCACTCAAAGGCGGTAATACGGTTATCCACAGAATCAGGGGATAACGCAGGAAAGAACATGTGAGCAA
AAGGCCAGCAAAAGGCCAGGAACCGTAAAAAGGCCGCGTTGCTGGCGTTTTTCCATAGGCTCCGCCCCCCTGACGA

GCATCACAAAAATCGACGCTCAAGTCAGAGGTGGCGAAACCCGACAGGACTATAAAGATACCAGGCGTTTCCCCCT
GGAAGCTCCCTCGTGCGCTCTCCTGTTCCGACCCTGCCGCTTACCGGATACCTGTCCGCCTTTCTCCCTTCGGGAAGC
GTGGCGCTTTCTCATAGCTCACGCTGTAGGNATCTCAGTTCGGTGTAGGTCGTTCGCTCCAAGCTGGGCTGTGTGCA
CGAACCCCCCGTTCAGCCCGACCGCTGCGCCTNATCCGGTAACTATCGTCTNAGTCCAACCCGGTANANACGACTTA
TCGCCACTGNNNCANCNCTNNNNNNNTAGCANANCNAGGNANGTAGNNGGNGCTACNNNNTTNNTGNANNN
NNNGNNTAACTACGGCTAN

>pTOPO-MADS21-519-colony-C-M13F_F07.ab1

NNNNNNNNNNNNNGGCGATTGGGCCCTCTAGATGCATGCTCGAGCGGCCGCCAGTGTGATGGATATCTGCAGAAT
TCGCCCTTCTGTTGTTGGCATACTCATAGAGGCGGCCACGGTTAGAGAAGACTATGAGAGCAACCTCTGCATCACAG
AGCACAGAGAGTTCATAGGCCTTCTTTTCTCTGGGGAGAGTCGATTGACAAGGATTTGTTTTCATTGGCCATAAGGG
CGAATTCCAGCACACTGGCGGCCGTTACTAGTGGATCCGAGCTCGGTACCAAGCTTGATGCATAGCTTGAGTATTCTA
TAGTGTCACCTAAATAGCTTGGCGTAATCATGGTCATAGCTGTTTCCTGTGTGAAATTGTTATCCGCTCACAATTCCACA
CAACATACGAGCCGGAAGCATAAAGTGTAAAGCCTGGGGTGCCTAATGAGTGAGCTAACTCACATTAATTGCGTTGC
GCTCACTGCCCGCTTTCCAGTCGGGAAACCTGTCGTGCCAGCTGCATTAATGAATCGGCCAACGCGCGGGGAGAGG
CGGTTTGCGTATTGGGCGCTCTTCCGCTTCCTCGCTCACTGACTCGCTGCGCTCGGTCGTTCGGCTGCGGCGAGCGG
TATCAGCTCACTCAAAGGCGGTAATACGGTTATCCACAGAATCAGGGGATAACGCAGGAAAGAACATGTGAGCAAA
AGGCCAGCAAAAGGCCAGGAACCGTAAAAAGGCCGCGTTGCTGGCGTTTTTCCATAGGCTCCGCCCCCCTGACGAG
CATCACAAAAATCGACGCTCAAGTCAGAGGTGGCGAAACCCGACAGGACTATAAAGATACCAGGCGTTTCCCCCTG
GAAGCTCCCTCGTGCGCTCTCCTGTTCCGACCCTGCCGCTTACCGGATACCTGTCCGCCTTTCTCCCTTCGGGAAGCG
TGGCGCTTTCTCATAGCTCACGCTGTAGGTATCTCAGTTCGGNGTNNNCGTTCGCTCCAAGCTGGGCTGTGTGCACG
AACCCCCCGTTCAGCCCGACCGCTGCGCCTTATCCGGTAACTANCGTCNTGANTCNNCNNNANACACGACTTATCN
CNCTNNNNCANCNCTGNNANNGNTTANCNNANCNAGGNANNNNNNNNGNGCTNNNNNTTCNTGANNNN

Event 520

MADS15

>pTOPO_MADS515_520_colony_4-M13F_B06.ab1

NNNNNNNNNNNNGGGCNNTTGGGCCCTCTAGATGCATGCTCGAGCGGCCGCCAGTGTGATGGATATCTGCAGAA
TTCGCCCTTATGGCCTATGAAAGCAAATCCTTGTCCTTGGACTCTCCCCAGAGAAAATTGGGTAGGGGAAAGATCGA
GATTAAGCGGATCGAAAACACAACGAATCGTCAAGTGACCTTCTGCAAGAGGCGCAATGGGTTGCTCAAGAAGGCC
TATGAACTCTCTGTGCTCTGTGATGCAGAGGTTGCTCTCATAGTCTTCTCTAACCGTGGCCGCCTCTATGAGTATGCCA
ACAATAGAAGGGCGAATTCCAGCACACTGGCGGCCGTTACTAGTGGATCCGAGCTCGGTACCAAGCTTGATGCATAG
CTTGAGTATTCTATAGTGTCACCTAAATAGCTTGGCGTAATCATGGTCATAGCTGTTTCCTGTGTGAAATTGTTATCCGC
TCACAATTCCACACAACATACGAGCCGGAAGCATAAAGTGTAAAGCCTGGGGTGCCTAATGAGTGAGCTAACTCACA
TTAATTGCGTTGCGCTCACTGCCCGCTTTCCAGTCGGGAAACCTGTCGTGCCAGCTGCATTAATGAATCGGCCAACGC
GCGGGGAGAGGCGGTTTGCGTATTGGGCGCTCTTCCGCTTCCTCGCTCACTGACTCGCTGCGCTCGGTCGTTCGGCT
GCGGCGAGCGGTATCAGCTCACTCAAAGGCGGTAATACGGTTATCCACAGAATCAGGGGATAACGCAGGAAAGAAC
ATGTGAGCAAAAGGCCAGCAAAAGGCCAGGAACCGTAAAAAGGCCGCGTTGCTGGCGTTTTTCCATAGGCTCCGCC
CCCCTGACGAGCATCACAAAAATCGACGCTCAAGTCAGAGGTGGCGAAACCCGACAGGACTNN

>pTOPO_MADS515_520_colony_5-M13F_C06.ab1

NNNNNNNNNNNNNGGCNNTTGGGCCCTCTAGATGCATGCTCGAGCGGCCGCCAGTGTGATGGATATCTGCAGAATT
CGCCCTTATGGCCTATGAAAGCAAATCCTTGTCAATCGACTCTCCCCAGAGAAAATTGGGTAGGGGAAAGATCGAGA
TAGAAGGCCTATGAACTCTCTGTGCTCTGTGATGCAGAGGTTGCTCTCATAGTCTTCTCTAACCGTGGCCGCCTCTATG
AGTATGCCAACAATAGAAGGGCGAATTCCAGCACACTGGCGGCCGTTACTAGTGGATCCGAGCTCGGTACCAAGCTT
GATGCATAGCTTGAGTATTCTATAGTGTCACCTAAATAGCTTGGCGTAATCATGGTCATAGCTGTTTCCTGTGTGAAATT
GTTATCCGCTCACAATTCCACACAACATACGAGCCGGAAGCATAAAGTGTAAAGCCTGGGGTGCCTAATGAGTGAGC
TAACTCACATTAATTGCGTTGCGCTCACTGCCCGCTTTCCAGTCGGGAAACCTGTCGTGCCAGCTGCATTAATGAATC
GGCCAACGCGCGGGGAGAGGCGGTTTGCGTATTGGGCGCTCTTCCGCTTCCTCGCTCACTGACTCGCTGCGCTCGG
TCGTTCGGCTGCGGCGAGCGGTATCAGCTCACTCAAAGGCGGTAATACGGTTATCCACAGAATCAGGGGATAACGCA
GGAAAGAACATGTGAGCAAAAGGCCAGCAAAAGGCCAGGAACCGTAAAAAGGCCGCGTTGCTGGCGTTTTTCCAT
AGGCTCCGCCCCCCTGACGAGCATCACAAAAATCGACGCTCAAGTCAGAGGTGGCGAAACCCGACAGGACTATAAA
GATACCAGGCGTTTCCCCCNGGNAGCTCCCTCGTGCGCTCTCCTGTTCCGACCCTGCCGCTTACCGGATACCTG

MADS221

>pTOPO-520-MADS221-colony1-M13F_A04.ab1
NNNNNNNNNNNNNNNGGGCGATTGGGCCCTCTAGATGCATGCTCGAGCGGCCGCCAGTGTGATGGATATCTGCAG
AATTCGCCCTTCTGTTGTTGGCATACTCATAGAGGCGGCCACGGTTAGAGAAGACTATGAGAGCAACCTCTGCATCAC
AGAGCACAGAGAGTTCATAGGCCTTCTTTTTCTCTGGGGAGAGTCGATTGACAAGGATTTGTTTTCATTGGCCATAA
GGGCGAATTCCAGCACACTGGCGGCCGTTACTAGTGGATCCGAGCTCGGTACCAAGCTTGATGCATAGCTTGAGTAT
TCTATAGTGTCACCTAAATAGCTTGGCGTAATCATGGTCATAGCTGTTTCCTGTGTGAAATTGTTATCCGCTCACAATTC
CACACAACATACGAGCCGGAAGCATAAAGTGTAAAGCCTGGGGTGCCTAATGAGTGAGCTAACTCACATTAATTGCG
TTGCGCTCACTGCCCGCTTTCCAGTCGGGAAACCTGTCGTGCCAGCTGCATTAATGAATCGGCCAACGCGCGGGGA
GAGGCGGTTTGCGTATTGGGCGCTCTTCCGCTTCCTCGCTCACTGACTCGCTGCGCTCGGTCGTTCGGCTGCGGCGA
GCGGTATCAGCTCACTCAAAGGCGGTAATACGGTTATCCACAGAATCAGGGGATAACGCAGGAAAGAACATGTGAG
CAAAAGGCCAGCAAAAGGCCAGGAACCGTAAAAAGGCCGCGTTGCTGGCGTTTTTCCATAGGCTCCGCCCCCCTGA
CGAGCATCACAAAAATCGACGCTCAAGTCAGAGGTGGCGAAACCCGACAGGACTATAAAGATACCAGGCGTTTCCC
CCTGGAAGCTCCCTCGTGCGCTCTCCTGTTCCGACCCTGCCGCTTACCGGATACCTGTCCGCCTTTCTCCCTTCGGGA
AGCGTGGCGCTTTCTCATAGCTCACGCTGTAGGNATCTCAGTTCGGTGTNNNCGTTCGCTCCAAGCTGGGCTGNGT
GCACGAACCCCCCGTTCAGCCCGACGCTGCGCTNATCCGGTANTATCGTCTTGANTCCAACCCGGTAGACACGACTT
ANCGCCNCTGGCAGCAGCNNCTGNNNACNNN

>pTOPO-520-MADS221-colony2-NB-M13F_B04.ab1
NNNNTNNNNNNNNNGGGCGATTGGGCCCTCTAGATGCATGCTCGAGCGGCCGCCAGTGTGATGGATATCTGCAGAA
TTCGCCCTTCTGTTGTTGGCATACTCATAGAGGCGGCCACGGTTAGAGAAGACTATGAGAGCAACCTCTGCATCACA
GAGCACAGAGAGTTCATAGGCCTTCTTTTTCTCTGGGGAGAGTCGATTGACAAGGATTTGTTTTCATTGGCCATAAG
GGCGAATTCCAGCACACTGGCGGCCGTTACTAGTGGATCCGAGCTCGGTACCAAGCTTGATGCATAGCTTGAGTATT
CTATAGTGTCACCTAAATAGCTTGGCGTAATCATGGTCATAGCTGTTTCCTGTGTGAAATTGTTATCCGCTCACAATTCC
ACACAACATACGAGCCGGAAGCATAAAGTGTAAAGCCTGGGGTGCCTAATGAGTGAGCTAACTCACATTAATTGCGT
TGCGCTCACTGCCCGCTTTCCAGTCGGGAAACCTGTCGTGCCAGCTGCATTAATGAATCGGCCAACGCGCGGGGAG
AGGCGGTTTGCGTATTGGGCGCTCTTCCGCTTCCTCGCTCACTGACTCGCTGCGCTCGGTCGTTCGGCTGCGGCGAG
CGGTATCAGCTCACTCAAAGGCGGTAATACGGTTATCCACAGAATCAGGGGATAACGCAGGAAAGAACATGTGAGC

AAAAGGCCAGCAAAAGGCCAGGAACCGTAAAAAGGCCGCGTTGCTGGCGTTTTTCCATAGGCTCCGCCCCCCTGAC
GAGCATCACAAAAATCGACGCTCAAGTCAGAGGTGGCGAAACCCGACAGGACTATAAAGATACCAGGCGTTTCCCC
CTGGAAGCTCCCTCGTGCGCTCTCCTGTTCCGACCCTGCCGCTTACCGGATACCTGTCCGCCTTTCTCCCTTCGGGAA
GCGTGGCGCTTTCTCATAGCTCACGCTGTAGGTATCTCAGTTCGGTGTNNNCGTTCGCTCCAAGCTGGGCTGTGTGC
ACGAACCCCCCGTTCAGCCCGACGCTGCGCTNATCNGTACTATCGTCTTGAGTCCANCCGGNAAGANNNGACTTAT
NGCCNCTGGNANCANCCACNGNNAACNGGATTANC

Event 521

MADS15

>pTOPO-521-MADS15-colony-2-M13F

NNNNNNTNNNNNNANNTGGGCCCTCTAGATGCATGCTCGAGCGGCCGCCAGTGTGATGGATATCTGCAGAATTCG
CCCTTTTTCATTTGTTTCTGCAAGTTTCAAACTTTTTGTTGGTGATCTAGAATATAGTAATTAATTAAAGTTCTGGGGTT
TTTATTTTAATTAGAAAGGTGCTTAATTGATTGATCTCTTTGTTCTTATTGATTCAGCTTCCAACTATGGCCTATGAAAGC
AAATCCTTGTCCTTGGACTCTCCCCAGAGAAAATTGGGTAGGGGAAAGATCGAGATTAAGCGGATCGAAAACACAA
CGAATCGTCAAGTGACCTTCTGCAAGAGGCGCAATGGGTTGCTCAAAGAAGGCCTATGAACTCTCTGTGCTCTGTGA
TGCAGAGGTTGCTCTCATAGTCTTCTCTAACCGTGGCCGCCTCTATGAGTATGCCAACAATAGGTAATATTATTGCTTCA
ATTTACTTGAAGGGCGAATTCCAGCACACTGGCGGCCGTTACTAGTGGATCCGAGCTCGGTACCAAGCTTGATGCAT
AGCTTGAGTATTCTATAGTGTCACCTAAATAGCTTGGCGTAATCATGGTCATAGCTGTTTCCTGTGTGAAATTGTTATCC
GCTCACAATTCCACACAACATACGAGCCGGAAGCATAAAGTGTAAAGCCTGGGGTGCCTAATGAGTGAGCTAACTCA
CATTAATTGCGTTGCGCTCACTGCCCGCTTTCCAGTCGGGAAACCTGTCGTGCCAGCTGCATTAATGAATCGGCCAAC
GCGCGGGGAGAGGCGGTTTGCGTATTGGGCGCTCTTCCGCTTCCTCGCTCACTGACTCGCTGCGCTCGGTCGTTCG
GCTGCGGCGAGCGGTATCAGCTCACTCAAAGGCGGTAATANNTTATCCACAGAATCNGGGGATAACGCNNNAAGA
NATGTGANCAAANNCAGCAAAGGCNGANNTAAAANNCGNNTTGCTGGNGTTTTCNTAGNNNCNNCCCCCCTGAC
NAGCATCNNAAAANCNACNCN

>pTOPO_MADS515_521_colony_1-M13F_F04.ab1

NNNNNNNNNNNNNNNNGGCGATTGGGCCCTCTAGATGCATGCTCGAGCGGCCGCCAGTGTGATGGATATCTGCAG
AATTCGCCCTTATGGCCTATGAAAGCAAATCCTTGTCCTTGGACTCTCCCCAGAGAAAATTGGGTAGGGGAAAGATC
GAGATTAAGCGGATCGAAAACACAACGAATCGTCAAGTGACCTTCTGCAAGAGGCGCAATGGGTTGCTCAAGAAG
GCCTATGAACTCTCTGTGCTCTGTGATGCAGAGGTTGCTCTCATAGTCTTCTCTAACCGTGGCCGCCTCTATGAGTATG
CCAACAATAGAAGGGCGAATTCCAGCACACTGGCGGCCGTTACTAGTGGATCCGAGCTCGGTACCAAGCTTGATGCA
TAGCTTGAGTATTCTATAGTGTCACCTAAATAGCTTGGCGTAATCATGGTCATAGCTGTTTCCTGTGTGAAATTGTTATC
CGCTCACAATTCCACACAACATACGAGCCGGAAGCATAAAGTGTAAAGCCTGGGGTGCCTAATGAGTGAGCTAACTC
ACATTAATTGCGTTGCGCTCACTGCCCGCTTTCCAGTCGGGAAACCTGTCGTGCCAGCTGCATTAATGAATCGGCCAA
CGCGCGGGGAGAGGCGGTTTGCGTATTGGGCGCTCTTCCGCTTCCTCGCTCACTGACTCGCTGCGCTCGGTCGTTCG
GCTGCGGCGAGCGGTATCAGCTCACTCAAAGGCGGTAATACGGTTATCCACAGAATCAGGGGATAACGCANGAAAG
AACATGTGAGCAAAAGGCCAGCAAAAGGCCAGGAACCGTAAAAAGGCCGCGTTGCTGGCGTTTTTCCATAGGCTCC
GCCCCCCTGACGAGCATCACAAAAATCGACGCTCAAGTCANAGGTGGCGAAACCCGACAGGACTATAAAGATACCA
GGCGTTTCCCCCTGGNANCTCCCTCGTGCGCTCTCCTGTTCCNACCTGCCGCTTACCNNNNCCTGTCCGCCTTTNTC
CTTCNGANCGTNNGCTTTCTCNNAGCTCNNNCTGTNGTATCTCANTCGNGTNGTCNTNGNCTNCAGCNGGNNNN

TNNNNNNNNACCCCCNNNANCCNNACNCNGCNNNNNNCGNACNANCGNNNNNANTCNANCCNGNAANANNC
GNNTNNCNNCCCNNNNN

MADS221

>521-pTOPO-MADS221-colony-3-M13F_E02.ab1

NNNNNNNNNNNNGGGCGATTGGGCCCTCTAGATGCATGCTCGAGCGGCCGCCAGTGTGATGGATATCTGCAGAAT
TCGCCCTTCTGTTGTTGGCATACTCATAGAGGCGGCCACGGTTAGAGAAGACTATGAGAGCAACCTCTGCATCACAG
AGCACAGAGAGTTCATAGGCCTTCTCTGGGGAGAGTCGATTGACAAGGATTTGTTTTCATTGGCCATAAGGGCGAAT
TCCAGCACACTGGCGGCCGTTACTAGTGGATCCGAGCTCGGTACCAAGCTTGATGCATAGCTTGAGTATTCTATAGTG
TCACCTAAATAGCTTGGCGTAATCATGGTCATAGCTGTTTCCTGTGTGAAATTGTTATCCGCTCACAATTCCACACAACA
TACGAGCCGGAAGCATAAAGTGTAAAGCCTGGGGTGCCTAATGAGTGAGCTAACTCACATTAATTGCGTTGCGCTCA
CTGCCCGCTTTCCAGTCGGGAAACCTGTCGTGCCAGCTGCATTAATGAATCGGCCAACGCGCGGGGAGAGGCGGTT
TGCGTATTGGGCGCTCTTCCGCTTCCTCGCTCACTGACTCGCTGCGCTCGGTCGTTCGGCTGCGGCGAGCGGTATCA
GCTCACTCAAAGGCGGTAATACGGTTATCCACAGAATCAGGGGATAACGCAGGAAAGAACATGTGAGCAAAAGGCC
AGCAAAAAGGCCAGGAACCGTAAAAAGGCCGCGTTGCTGGCGTTTTTCCATAGGCTCCGCCCCCCTGACGAGCATC
ACAAAAATCGACGCTCANGTCAGAGGTGGCGAAACCCGACAGGACTATAAAGATACCAGGCGTTTCCCCCTGGAAG
CTCCCTCGNGCGCTCTCCTGTTCCCGANCCTGCCGCTTACCGGNATACCTGTCCGCCTTTCTCCCTTTCGGGAAGCGT
GGNGNTTTCTCATNGCTCANGCTGTANGTATCTCAGTTNNGTGNNNGTCNTTCGNTTCCNNGCTGNGNNNTGNGC
ACNAACCNCNCNNTTCNNNCGNANNNTGCTGCTNNNTCNGGTANNTATCGTCNTNNANNCCANNNNGGTANNN
ANNNACTNANNNNAGNGGGCANNNNCNNNNNNNNNGACATANANANNNAGANNNNN

>521-pTOPO-MADS221-colony-4-M13F_F02.ab1

NNNNNNNNNNNNANGGCGATTGGGCCCTCTAGATGCATGCTCGAGCGGCCGCCAGTGTGATGGATATCTGCAGAAT
TCGCCCTTCTGTTGTTGGCATACTCATAGAGGCGGCCACGGTTAGAGAAGACTATGAGAGCAACCTCTGCATCACAG
AGCACAGAGAGTTCATAGGCCTTCTTGAGCAACCCATTGCGCCTCTTGCAGAAGGTCACTTGACGATTGGTCGTGTT
TTCGATCCGCTTGATCTCGATCTTTCCCCTACCCAATTTTCTCTGGGGAGAGTCGATTGACAAGGATTTGTTTTCATTG
GCCATAAGGGCGAATTCCAGCACACTGGCGGCCGTTACTAGTGGATCCGAGCTCGGTACCAAGCTTGATGCATAGCT
TGAGTATTCTATAGTGTCACCTAAATAGCTTGGCGTAATCATGGTCATAGCTGTTTCCTGTGTGAAATTGTTATCCGCTC
ACAATTCCACACAACATACGAGCCGGAAGCATAAAGTGTAAAGCCTGGGGTGCCTAATGAGTGAGCTAACTCACATT
AATTGCGTTGCGCTCACTGCCCGCTTTCCAGTCGGGAAACCTGTCGTGCCAGCTGCATTAATGAATCGGCCAACGCG
CGGGGAGAGGCGGTTTGCGTATTGGGCGCTCTTCCGCTTCCTCGCTCACTGACTCGCTGCGCNNCGGTCGTTCGGC
TGCGGCGAGCGGTATCAGCTCACTCAAAGGCGGTAATACGGTTATCCACAGAATCAGGGGATAACGCAGGAAAGAA
CATGTGAGCAAAAGGCCAGCAAAAGGCCAGGAACCGTAAAAAGGCCGCGTTGCTGGCGTTTTTCCATANGCTCCGC
CCCCCTGACGAGCATCACAAAAATCGACGCTCANGTCAGAGGTGNNGAAACCCCGACAGGACTATAAAGATACCAN
GCGTTTCCCCTNNAGCTCCTTCGTGCGCTCTCCNGTTCCGACCCNGCCGCTTACCGGATACCTGTCCNGCNTTTCNN
CCTTCNGGNNCGTGNGCTTTCTCNTAGCTCANGCTGNAGTATCTNAAGTTNNNNGNNNGNNGTNCNNNNNNAG
CNNNNCTGTNNNGNNNNANCCCNNNN’

Event 522

MADS15

>522-pTOPO-MADS15-colony-3-M13F_G03.ab1

NNNNNNNNNNNNNANGGCGANTGGGCCCTCTAGATGCATGCTCGAGCGGCCGCCAGTGTGATGGATATCTGCAG
AATTCGCCCTTCTATTGTTGGCATACTCATAGAGGCGGCCACGGTTAGAGAAGACTATGAGAGCAACCTCTGCATCAC
AGAGCACAGAGAGTTCATAGGCCTTCCCATTGCGCCTCTTGCAGAAGGTCACTTGACGATTCGTTGTGTTTTCGATCC
GCTTAATCTCGATCTTTCCCCTACCCAATTTTCTCTGGGGAGAGTCCAAGGACAAGGATTTGCTTTCATAGGCCATAAG
GGCGAATTCCAGCACACTGGCGGCCGTTACTAGTGGATCCGAGCTCGGTACCAAGCTTGATGCATAGCTTGAGTATT
CTATAGTGTCACCTAAATAGCTTGGCGTAATCATGGTCATAGCTGTTTCCTGTGTGAAATTGTTATCCGCTCACAATTCC
ACACAACATACGAGCCGGAAGCATAAAGTGTAAAGCCTGGGGTGCCTAATGAGTGAGCTAACTCACATTAATTGCGT
TGCGCTCACTGCCCGCTTTCCAGTCGGGAAACCTGTCGTGCCAGCTGCATTAATGAATCGGCCAACGCGCGGGGAG
AGGCGGTTTGCGTATTGGGCGCTCTTCCGCTTCCTCGCTCACTGACTCGCTGCGCTCGGTCGTTCGGCTGCGGCGAG
CGGTATCAGCTCACTCAAAGGCGGTAATACGGTTATCCACAGAATCAGGGGATAACGCAGGAAAGAACATGTGAGC
AAAAGGCCAGCAAAAGGCCAGGAACCGTAAAAAGGCCGCGTTTGCCTGGCGTTTTTCCATAGGCTCCGCCCCCCTG
ACGAGCATCACAAAAATCGACGCTCAAGTCAGANGTGGCGAAACCCGACAGGACTATAAAGATACCAGGCGTTTCC
CCCTNNNGCTCCCTCGTGCGCTCTCCTGTTCCGACCCTGCCGCTTACCGGATACCTGTCCGCCTTTCTCCCTTTCGGG
AAGCGTGNNCTTTCTCNTAGCTCACGCTGTAAGGTATCNNNCNNTTCGGNGNAGGTCGTTNNNNNCAAGCNGGG
NCTGNGTGCACGAANCCCCCNTNCANCCCGANNNN

>522-pTOPO-MADS15-colony-8-M13F_H03.ab1

NNNNNNNNNNNNNNNNGGCGATTGGGCCCTCTAGATGCATGCTCGAGCGGCCGCCAGTGTGATGGATATCTGCAG
AATTCGCCCTTCTATTGTTGGCATACTCATAGAGGCGGCCACGGTTAGAGAAGACTATGAGAGCAACCTCTGCATCAC
AGAGCACAGAGAGTTCATAGGCCTTCTCTGGGGAGAGTCGATTGACAAGGATTTGCTTTCATAGGCCATAAGGGCG
AATTCCAGCACACTGGCGGCCGTTACTAGTGGATCCGAGCTCGGTACCAAGCTTGATGCATAGCTTGAGTATTCTATA
GTGTCACCTAAATAGCTTGGCGTAATCATGGTCATAGCTGTTTCCTGTGTGAAATTGTTATCCGCTCACAATTCCACACA
ACATACGAGCCGGAAGCATAAAGTGTAAAGCCTGGGGTGCCTAATGAGTGAGCTAACTCACATTAATTGCGTTGCGC
TCACTGCCCGCTTTCCAGTCGGGAAACCTGTCGTGCCAGCTGCATTAATGAATCGGCCAACGCGCGGGGAGAGGCG
GTTTGCGTATTGGGCGCTCTTCCGCTTCCTCGCTCACTGACTCGCTGCGCTCGGTCGTTCGGCTGCGGCGAGCGGTAT
CAGCTCACTCAAAGGCGGTAATACGGTTATCCACAGAATCAGGGGATAACGCAGGAAAGAACATGTGAGCAAAAGG
CCAGCAAAAGGCCAGGAACCGTAAAAAGGCCGCGTTGCTGGCGTTTTTCCATAGGCTCCGCCCCCCTGACGAGCAT
CACAAAAATCGACGCTCAAGTCAGAGGTGGCGAAACCCGACAGGACTATAAAGATACCAGGCGTTTCCCCCTGGAA
GCTCCCTCGTGCGCTCTCCTGTTCCGACCCTGCCGCTTACCGGATACCTGTCCGCCTTTCTCCCTTCGGGAAGCGTGG
CGCTTTCTCATAGCTCACGCTGTNNGGTATCTCAGTTCGGGNGTAGGTCGTTNGCTCCAAGCTGGGCTGTGTGCACG
AACCCCCCGTTCAGCCCGACCGCTGCGCCTTANCCGGTAACTATCGNCTTGAGTCCAACCCGGGTAANNACN

MADS221

>pTOPO_MADS221_522_colony_3-M13F_E04.ab1

NNNNNNNNNNNGGGCGATTGGGCCCTCTAGATGCATGCTCGAGCGGCCGCCAGTGTGATGGATATCTGCAGAATT
CGCCCTTATGGCCAATGAAAACAAATCCTTGTCAATCGACTCTCCCCAGAGAAAATTGGGTAGGGGAAAGATCGAGA
TCAAGCGGATCGAAAACACGACCAATCGTCAAGTGACCTTCTGCAAGAGGCGCAATGGGTTGCTAGAAGGCCTATG

AACTCTCTGTGCTCTGTGATGCAGAGGTTGCTCTCATAGTCTTCTCTAACCGTGGCCGCCTCTATGAGTATGCCAACAA
CAGAAGGGCGAATTCCAGCACACTGGCGGCCGTTACTAGTGGATCCGAGCTCGGTACCAAGCTTGATGCATAGCTTG
AGTATTCTATAGTGTCACCTAAATAGCTTGGCGTAATCATGGTCATAGCTGTTTCCTGTGTGAAATTGTTATCCGCTCAC
AATTCCACACAACATACGAGCCGGAAGCATAAAGTGTAAAGCCTGGGGTGCCTAATGAGTGAGCTAACTCACATTAA
TTGCGTTGCGCTCACTGCCCGCTTTCCAGTCGGGAAACCTGTCGTGCCAGCTGCATTAATGAATCGGCCAACGCGCG
GGGAGAGGCGGTTTGCGTATTGGGCGCTCTTCCGCTTCCTCGCTCACTGACTCGCTGCGCTCGGTCGTTCGGCTGCG
GCGAGCGGTATCAGCTCACTCAAAGGCGGTAATACGGTTATCCACAGAATCAGGGGATAACGCANGAAAGAACATG
TGAGCAAAAGGCCAGCAAAAGGCCAGGAACCGTAAAAAGGCCGCGTTTGCTGGCGTTTTTCCATNGGCTCCGCCC
CCCTGACGAGCATCACAAAAATCGACGCTCAAGTCAGAGGTGGCGAAACCCGACAGGACTATAAAGATACCAGGCG
TTTN

>pTOPO_MADS221_522_colony_4-M13F_G12.ab1

NNNNNNNNNNNGGCGATTGGGCCCTCTAGATGCATGCTCGAGCGGCCGCCAGTGTGATGGATATCTGCAGAATTC
GCCCTTATGGCCAATGAAAACAAATCCTTGTCAATCGACTCTCCCCAGAGAAAATTGGGTAGGGGAAAGATCGAGAT
CAAGCGGATCGAAAACACGACCAATCGTCAAGTGACCTTCTGCAAGAGGCGCAATGGGTTGCTCCTATGAACTCTCT
GTGCTCTGTGATGCAGAGGTTGCTCTCATAGTCTTCTCTAACCGTGGCCGCCTCTATGAGTATGCCAACAACAGAAGG
GCGAATTCCAGCACACTGGCGGCCGTTACTAGTGGATCCGAGCTCGGTACCAAGCTTGATGCATAGCTTGAGTATTCT
ATAGTGTCACCTAAATAGCTTGGCGTAATCATGGTCATAGCTGTTTCCTGTGTGAAATTGTTATCCGCTCACAATTCCAC
ACAACATACGAGCCGGAAGCATAAAGTGTAAAGCCTGGGGTGCCTAATGAGTGAGCTAACTCACATTAATTGCGTTG
CGCTCACTGCCCGCTTTCCAGTCGGGAAACCTGTCGTGCCAGCTGCATTAATGAATCGGCCAACGCGCGGGGAGAG
GCGGTTTGCGTATTGGGCGCTCTTCCGCTTCCTCGCTCACTGACTCGCTGCGCTCGGTCGTTCGGCTGCGGCGAGCG
GTATCAGCTCACTCAAAGGCGGTAATACGGTTATCCACAGAATCAGGGGATAACGCNNGAAAGAACATGTGAGCAA
AAGGNCAGCAAAAGGCCAGNAACCGTAAAAAGGNCCNNNNN

Event 523

<u>MADS15</u>

>523-pTOPO-MADS15-colony-5-M13F_C02.ab1

NNNNNNNNNNNNNNGGGCGATTGGGCCCTCTAGATGCATGCTCGAGCGGCCGCCAGTGTGATGGATATCTGCAG
AATTCGCCCTTCTATTGTTGGCATACTCATAGAGGCGGCCACGGTTAGAGAAGACTATGAGAGCAACCTCTGCATCAC
AGAGCACAGAGAGTTCATAGGCCTTCTAGCAACCCATTGCGCCTCTTGCAGAAGGTCACTTGACGATTCGTTGTGTT
TTCGATCCGCTTAATCTCGATCTTTCCCCTACCCAATTTTCTCTGGGGAGAGTCCAAGGACAAGGATTTGCTTTCATAG
GCCATAAGGGCGAATTCCAGCACACTGGCGGCCGTTACTAGTGGATCCGAGCTCGGTACCAAGCTTGATGCATAGCT
TGAGTATTCTATAGTGTCACCTAAATAGCTTGGCGTAATCATGGTCATAGCTGTTTCCTGTGTGAAATTGTTATCCGCTC
ACAATTCCACACAACATACGAGCCGGAAGCATAAAGTGTAAAGCCTGGGGTGCCTAATGAGTGAGCTAACTCACATT
AATTGCGTTGCGCTCACTGCCCGCTTTCCAGTCGGGAAACCTGTCGTGCCAGCTGCATTAATGAATCGGCCAACGCG
CGGGGAGAGGCGGTTTGCGTATTGGGCGCTCTTCCGCTTCCTCGCTCACTGACTCGCTGCGCTCGGTCGTTCGGCTG
CGGCGAGCGGTATCAGCTCACTCAAAGGCGGTAATACGGTTATCCACAGAATCAGGGGATAACGCAGGAAAGAACA
TGTGAGCAAAAGGCCAGCAAAAGGCCAGGAACCGTAAAAAGGCCGCGTTGCTGGCGTTTTTCCATAGGCTCCGCC
CCCCTGACGAGCATCACAAAAATCGACGCTCAAGTCAGANGTGGCGAAACCCGACAGGACTATAAAGATACCAGGC
GTTTCCCCCTGGAAGCTCCCTCGTGCGCTCTCCTGTTCCNACCCTGCCGCTTACCGGATACCTGTCCGCCTTTCTCCCT

TCGGGAAGCGTGNNCTTTCTCATAGCTCACGCTGNNNGGTANNNNNCAGNTTNNNGNNGGTAGGGTCNNNNNC
TCCNAAGCCTGGGGNNGNGNNGNACGAACNCNCCCNTNCANNCCCGACCGNTGCN

>pTOPO_MADS515_523_colony_1-M13F_G11.ab1

NNNNNNNNNNNGGGCGATTGGGCCCTCTAGATGCATGCTCGAGCGGCCGCCAGTGTGATGGATATCTGCAGAATT
CGCCCTTATGGCCTATGAAAGCAAATCCTTGTCCTTGGACTCTCCCCAGAGAAAATTGGGTAGGGGAAAGATCGAGA
TTAAGCGGATCGAAAACACAACGAATCGTCAAGTGACCTTCTGCAAGAGGCGCAATGGGTTGCTCAAGAAGGCCTA
TGAACTCTCTGTGCTCTGTGATGCAGAGGTTGCTCTCATAGTCTTCTCTAACCGTGGCCGCCTCTATGAGTATGCCAAC
AATAGAAGGGCGAATTCCAGCACACTGGCGGCCGTTACTAGTGGATCCGAGCTCGGTACCAAGCTTGATGCATAGCT
TGAGTATTCTATAGTGTCACCTAAATAGCTTGGCGTAATCATGGTCATAGCTGTTTCCTGTGTGAAATTGTTATCCGCTC
ACAATTCCACACAACATACGAGCCGGAAGCATAAAGTGTAAAGCCTGGGGTGCCTAATGAGTGAGCTAACTCACATT
AATTGCGTTGCGCTCACTGCCCGCTTTCCAGTCGGGAAACCTGTCGTGCCAGCTGCATTAATGAATCGGCCAACGCG
CGGGGAGAGGCGGTTTGCGTATTGGGCGCTCTTCCGCTTCCTCGCTCACTGACTCGCTGCGCTCGGTCGTTCGGCTG
CGGCGAGCGGTATCAGCTCACTCAAAGGCGGTAATANNGTTATCCACAGAATCNNGGANANGCNNNANANATGTG
AGCAAAAGGCCAGCAAAAGGCCAGNANCGTAAAAGNCCNCGTNNCNGGGCGTTTTNCCATNN

MADS221

>pTOPO_MADS221_523_colony_4-M13F_E07.ab1

NNNNNNNNNNNNNNNNANGGGCCCTCTAGATGCATGCTCGAGCGGCCGCCAGTGTGATGGATATCTGCAGAATTC
GCCCTTTTCATTTGTTTCTGCAAGTTAATTTCAAACTTTTTGTTGGTGATCAAGAATATAGTAATTGAAGTTCTGGGGT
TTTTGTTTTATTTAAAAAGGTGCTTAATTGATTGATCTCTTTGTTCTTATTGATTCAGCTTGCAACTATGGCCAATGAAA
ACAAATCCTTGTCAATCGACTCTCCCCAGAGAAAATTGGGTAGGGGAAAGATCGAGATCAAGAAGGCCTATGAACTC
TCTGTGCTCTGTGATGCAGAGGTTGCTCTCATAGTCTTCTCTAACCGTGGCCGCCTCTATGAGTATGCCAACAACAGGT
AATAATATTATTGCTTCAATTTTCTTGCTAAGGGCGAATTCCAGCACACTGGCGGCCGTTACTAGTGGATCCGAGCTCG
GTACCAAGCTTGATGCATAGCTTGAGTATTCTATAGTGTCACCTAAATAGCTTGGCGTAATCATGGTCATAGCTGTTTCC
TGTGTGAAATTGTTATCCGCTCACAATTCCACACAACATACGAGCCGGAAGCATAAAGTGTAAAGCCTGGGGTGCCTA
ATGAGTGAGCTAACTCACATTAATTGCGTTGCGCTCACTGCCCGCTTTCCAGTCGGGAAACCTGTCGTGCCAGCTGCA
TTAATGAATCGGCCAACGCGCGGGGAGAGGCGGTTTGCGTATTGGGCGCTCTTCCGCTTCCTCGCTCACTGACTCGC
TGCGCTCGGTCGTTCGGCTGCGGCGAGCGGTATCAGCTCACTCAAAGGCGGTAATACGGTTATCCACAGAATCNNG
GGATAACGCANNAAAGANATGTGAGCAAAANNCAGCAAAAGGCCAGGAACCGTAAAAAGGCCGCGTTGCTGGCN
TTTTTCCATAGNNNNCNCCCCCTGACGAGCATCN

>pTOPO_MADS221_523_colony_6-M13F_F07.ab1

NNNNNNNNNNNNNNGGNGATTGGGCCCTCTAGATGCATGCTCGAGCGGCCGCCAGTGTGATGGATATCTGCAGAAT
TCGCCCTTTTCATTTGTTTCTGCAAGTTAATTTCAAACTTTTTGTTGGTGATCAAGAATATAGTAATTGAAGTTCTGGG
GTTTTTGTTTTATTTAAAAAGGTGCTTAATTGATTGATCTCTTTGTTCTTATTGATTCAGCTTGCAACTATGGCCAATGA
AAACAAATCCTTGTCAATCGACTCTCCCCAGAGAAAATTGGGTAGGGGAAAGATCGAGATGCGGATCGAAAACACG
ACCAATCGTCAAGTGACCTTCTGCAAGAGGCGCAATGGGTTGCTCAAAGAAGGCCTATGAACTCTCTGTGCTCTGTG
ATGCAGAGGTTGCTCTCATAGTCTTCTCTAACCGTGGCCGCCTCTATGAGTATGCCAACAACAGGTAATAATATTATTGC

TTCAATTTTCTTGCTAAGGGCGAATTCCAGCACACTGGCGGCCGTTACTAGTGGATCCGAGCTCGGTACCAAGCTTG
ATGCATAGCTTGAGTATTCTATAGTGTCACCTAAATAGCTTGGCGTAATCATGGTCATAGCTGTTTCCTGTGTGAAATTG
TTATCCGCTCACAATTCCACACAACATACGAGCCGGAAGCATAAAGTGTAAAGCCTGGGGTGCCTAATGAGTGAGCT
AACTCACATTAATTGCGTTGCGCTCACTGCCCGCTTTCCAGTCGGGAAACCTGTCGTGCCAGCTGCATTAATGAATCG
GCCAACGCGCGGGGAGAGGCGGTTTGCGTATTGGGCGCTCTTCCGCTTCCTCGCTCACTGACTCGCTGCGCTCGGT
CGTTCGGCTGCGGCGAGCGGTATCAGCTCACTCAAAGGCGGTAATACGGTTATCCACAGAATCNGGGGATAACGCN
NNAAGAACATGTGAGCAAAGGCCAGCAANN

Event 524

MADS15

>pTOPO-524-MADS15-colony1-M13F_G03.ab1

NNNNNNNNNNNNNNGGCGATTGGGCCCTCTAGATGCATGCTCGAGCGGCCGCCAGTGTGATGGATATCTGCAGAAT
TCGCCCTTATGCCTATGAAAGCAAATCCTTGTCCTTGGACTCTCCCCAGAGAAAATTGGGTAGGGGAAAGATCGAGA
TTAAGCGGATCGAAAACACAACGAATCGTCAAGTGACCTTCTGCAAGAGGCGCAATGGGTTGCAGAAGGCCTATGA
ACTCTCTGTGCTCTGTGATGCAGAGGTTGCTCTCATAGTCTTCTCTAACCGTGGCCGCCTCTATGAGTATGCCAACAAT
AGAAGGGCGAATTCCAGCACACTGGCGGCCGTTACTAGTGGATCCGAGCTCGGTACCAAGCTTGATGCATAGCTTGA
GTATTCTATAGTGTCACCTAAATAGCTTGGCGTAATCATGGTCATAGCTGTTTCCTGTGTGAAATTGTTATCCGCTCACA
ATTCCACACAACATACGAGCCGGAAGCATAAAGTGTAAAGCCTGGGGTGCCTAATGAGTGAGCTAACTCACATTAATT
GCGTTGCGCTCACTGCCCGCTTTCCAGTCGGGAAACCTGTCGTGCCAGCTGCATTAATGAATCGGCCAACGCGCGGG
GAGAGGCGGTTTGCGTATTGGGCGCTCTTCCGCTTCCTCGCTCACTGACTCGCTGCGCTCGGTCGTTCGGCTGCGGC
GAGCGGTATCAGCTCACTCAAAGGCGGTAATACGGTTATCCACAGAATCAGGGGATAACGCAGGAAAGAACATGTG
AGCAAAAGGCCAGCAAAAGGCCAGGAACCGTAAAAAGGCCGCGTTGCTGGCGTTTTTCCATAGGCTCCGCCCCCCT
GACGAGCATCACAAAAATCGACGCTCAAGTCAGAGGTGGCGAAACCCGACAGGACTATAAAGATACCAGGCGTTTC
CCCCNGGNAGCTCCCTCGTGCGCTCTCCTGTTCCGACCCTGCCGCTTACCGGATACCTGTCCGCCTTTCTCCCTTCGG
GANGCGTGNGCTTTCTCATAGCTCACGCTNNNNNNNNNCANTTCGNNGTNGNCGTTCGCNNCNAGCTNGGNNN
NNNNGNN

>524-pTOPO-MADS15-colony-3-M13F_A02.ab1

NNNNNNNNNNNNNANGGCNANTGGGCCCTCTAGATGCATGCTCGAGCGGCCGCCAGTGTGATGGATATCTGCAGA
ATTCGCCCTTCTATTGTTGGCATACTCATAGAGGCGGCCACGGTTAGAGAAGACTATGAGAGCAACCTCTGCATCACA
GAGCACAGAGAGTTCATAGGCCTTACCCATTGCGCCTCTTGCAGAAGGTCACTTGACGATTCGTTGTGTTTTCGATCC
GCTTAATCTCGATCTTTCCCCTACCCAATTTTCTCTGGGGAGAGTCCAAGGACAAGGATTTGCTTTCATAGGCCATAAG
GGCGAATTCCAGCACACTGGCGGCCGTTACTAGTGGATCCGAGCTCGGTACCAAGCTTGATGCATAGCTTGAGTATT
CTATAGTGTCACCTAAATAGCTTGGCGTAATCATGGTCATAGCTGTTTCCTGTGTGAAATTGTTATCCGCTCACAATTCC
ACACAACATACGAGCCGGAAGCATAAAGTGTAAAGCCTGGGGTGCCTAATGAGTGAGCTAACTCACATTAATTGCGT
TGCGCTCACTGCCCGCTTTCCAGTCGGGAAACCTGTCGTGCCAGCTGCATTAATGAATCGGCCAACGCGCGGGGAG
AGGCGGTTTGCGTATTGGGCGCTCTTCCGCTTCCTCGCTCACTGACTCGCTGCGCTCGGTCGTTCGGCTGCGGCGAG
CGGTATCAGCTCACTCAAAGGCGGTAATACGGTTATCCACAGAATCAGGGGATAACGCAGGAAAGAACATGTGAGC
AAAAGGCCAGCAAAAGGCCAGGAACCGTAAAAAGGCCGCGTTGCTGGCGTTTTTCCATAGGCTCCGCCCCCCTGAC
GAGCATCACAAAAATCGACGCTCAAGTCAGANGTGGCGAAACCCGACAGGACTATAAAGATACCAGGCGTTTCCCC

CTNNNGCTCCCTCGTGCGCTCTCCTGTTCCGACCCTGCCGCTTACCGGATACCTGTCCGCCTTTCTCCCNTCGGNAGC
GTGNGCTTTCTCATAGCTCACGCTGNNNNTATCTCAGTTCGGNGTAGGTCGNTCGCTCCNAGCTGGGN

MADS221

>pTOPO_MADS221_524_colony_1-M13F_D06.ab1

NNNNNNNNNNNGGGGCGATTGGGCCCTCTAGATGCATGCTCGAGCGGCCGCCAGTGTGATGGATATCTGCAGAAT
TCGCCCTTATGGCCAATGAAAACAAATCCTTGTCAATCGACTCTCCCCAGAGAAAATTGGGTAGGGGAAAGATCGAG
ATCAAGCGGATCGAAAACACGACCAATCGTCAAGTGACCTTCTGCAAGAGGCGCAATGGGTTGCTCAAGAAGGCCT
ATGAACTCTCTGTGCTCTGTGATGCAGAGGTTGCTCTCATAGTCTTCTCTAACCGTGGCCGCCTCTATGAGTATGCCAA
CAACAGAAGGGCGAATTCCAGCACACTGGCGGCCGTTACTAGTGGATCCGAGCTCGGTACCAAGCTTGATGCATAG
CTTGAGTATTCTATAGTGTCACCTAAATAGCTTGGCGTAATCATGGTCATAGCTGTTTCCTGTGTGAAATTGTTATCCGC
TCACAATTCCACACAACATACGAGCCGGAAGCATAAAGTGTAAAGCCTGGGGTGCCTAATGAGTGAGCTAACTCACA
TTAATTGCGTTGCGCTCACTGCCCGCTTTCCAGTCGGGAAACCTGTCGTGCCAGCTGCATTAATGAATCGGCCAACGC
GCGGGGAGAGGCGGTTTGCGTATTGGGCGCTCTTCCGCTTCCTCGCTCACTGACTCGCTGCGCTCGGTCGTTCGGCT
GCGGCGAGCGGTATCAGCTCACTCAAAGGCGGTAATACGGTTATCCACAGAATCAGGGGATAACGCANGAAAGAAC
ATGTGAGCAAAAGGCCAGCAAAAGGCCAGGAACCGTAAAAAGGCCGCGTTGCTGGCGTTTTTCCATAGGCTCCGCC
CCCCTGACGAGCATCACAAAAATCGACGCTCAAGTCAGAGGTGGCGAAACCCGACAGGACTATAAAGATACCNNNN

>pTOPO_MADS221_524_colony_3-M13F_F12.ab1

NNNNNNNNNNNNNNNGGCGATTGGGCCCTCTAGATGCATGCTCGAGCGGCCGCCAGTGTGATGGATATCTGCAGA
ATTCGCCCTTCTGTTGTTGGCATACTCATAGAGGCGGCCACGGTTAGAGAAGACTATGAGAGCAACCTCTGCATCACA
GAGCACAGAGAGTTCATAGGCCTTCTTTGAGCAACCCATTGCGCCTCTTGCAGAAGGTCACTTGACGATTGGTCGTG
TTTTCGATCCGCTTGTCTCGATCTTTCCCCTACCCAATTTTCTCTGGGGAGAGTCGATTGACAAGGATTTGTTTTCATT
GGCCATAAGGGCGAATTCCAGCACACTGGCGGCCGTTACTAGTGGATCCGAGCTCGGTACCAAGCTTGATGCATAGC
TTGAGTATTCTATAGTGTCACCTAAATAGCTTGGCGTAATCATGGTCATAGCTGTTTCCTGTGTGAAATTGTTATCCGCT
CACAATTCCACACAACATACGAGCCGGAAGCATAAAGTGTAAAGCCTGGGGTGCCTAATGAGTGAGCTAACTCACAT
TAATTGCGTTGCGCTCACTGCCCGCTTTCCAGTCGGGAAACCTGTCGTGCCAGCTGCATTAATGAATCGGCCAACGCG
CGGGGAGAGGCGGTTTGCGTATTGGGCGCTCTTCCGCTTCCTCGCTCACTGACTCGCTGCGCTCGGTCGTTCGGCTG
CGGCGAGCGGTATCAGCTCACTCAAAGGCGGTAATACGGTTATCCACAGAATCANGGGATAACGCAGNAAGAACAT
GTGAGCAAAAGGNNNGCAAAAGGCCNGGNNN

Event 525

MADS15

>pTOPO_MADS515_525_colony_4-M13F_C09.ab1

NNNNNNNNNNNANGGCGATTGGGCCCTCTAGATGCATGCTCGAGCGGCCGCCAGTGTGATGGATATCTGCAGAAT
TCGCCCTTTTTCATTTGTTTCTGCAAGTTTCAAACTTTTTGTTGGTGATCTAGAATATAGTAATTAATTAAAGTTCTGGG
GTTTTTATTTTAATTAGAAAGGTGCTTAATTGATTGATCTCTTTGTTCTTATTGATTCAGCTTCCAACTATGGCCTATGAA
AGCAAATCCTTGTCCTTGGACTCTCCCCAGAGAAAATTGGGTAGGGGAAAGATCGAGATTAAGCGGATCGAAAACA

CAACGAATCGTCAAGTGACCTTCTGCAAGAGGCGCAATGGGTTGCAGAAGGCCTATGAACTCTCTGTGCTCTGTGAT
GCAGAGGTTGCTCTCATAGTCTTCTCTAACCGTGGCCGCCTCTATGAGTATGCCAACAATAGGTAATATTATTGCTTCAA
TTTACTTGAAGGGCGAATTCCAGCACACTGGCGGCCGTTACTAGTGGATCCGAGCTCGGTACCAAGCTTGATGCATA
GCTTGAGTATTCTATAGTGTCACCTAAATAGCTTGGCGTAATCATGGTCATAGCTGTTTCCTGTGTGAAATTGTTATCCG
CTCACAATTCCACACAACATACGAGCCGGAAGCATAAAGTGTAAAGCCTGGGGTGCCTAATGAGTGAGCTAACTCAC
ATTAATTGCGTTGCGCTCACTGCCCGCTTTCCAGTCNGGAAACCTGTCGTGCCAGCTGCATTAATGAATCGGCCAACG
CGCGGGGAGAGGCGGTTTGCGTATTGGGCGCTCTTCCGCTTCCTCGCTCACTGACTCGCTGCGCTCGGTCGTTCGGC
TGCGGCGAGCGGTATCAGCTCACTCAAAGGCGGTAATACGGTNATCCACAGAATCNGGGGATNACGCNNNAAGAA
CATGTGAGCAAAAGGCCANCAAAAGGCCANGAACCGTAAAAANGCCNNN

>pTOPO_MADS515_525_colony_3-M13F_B09.ab1

NNNNNNNNNNNNNNNNGATTGGGCCCTCTAGATGCATGCTCGAGCGGCCGCCAGTGTGATGGATATCTGCAGAA
TTCGCCCTTATGGCCTATGAAAGCAAATCCTTGTCAATCGACTCTCCCCAGAGAAAATTGGGTAGGGGAAAGATCGA
GATAGAAGGCCTATGAACTCTCTGTGCTCTGTGATGCAGAGGTTGCTCTCATAGTCTTCTCTAACCGTGGCCGCCTCTA
TGAGTATGCCAACAATAGAAGGGCGAATTCCAGCACACTGGCGGCCGTTACTAGTGGATCCGAGCTCGGTACCAAGC
TTGATGCATAGCTTGAGTATTCTATAGTGTCACCTAAATAGCTTGGCGTAATCATGGTCATAGCTGTTTCCTGTGTGAAA
TTGTTATCCGCTCACAATTCCACACAACATACGAGCCGGAAGCATAAAGTGTAAAGCCTGGGGTGCCTAATGAGTGA
GCTAACTCACATTAATTGCGTTGCGCTCACTGCCCGCTTTCCAGTCGGGAAACCTGTCGTGCCAGCTGCATTAATGAA
TCGGCCAACGCGCGGGGAGAGGCGGTTTGCGTATTGGGCGCTCTTCCGCTTCCTCGCTCACTGACTCGCTGCGCTC
GGTCGTTCGGCTGCGGCGAGCGGTATCAGCTCACTCAAAGGCGGTAATACGGTTATCCACAGAATCAGGGGATAAC
GCANGAAAGAACATGTGAGCAAAAGGCCAGCAAAAGGCCAGGAACCGTAAAAAGGCCGCGTTGCTGGCGTTTTTC
CATAGGCTCCGCCCCCCTGACGAGCATCACAAAAATCGACGCTCAAGTCAGAGGTGGCGAAACCCGACAGGACTAT
AAAGATACCAGGCGTTTCCCCCTGGAAGCTCCCTCGTGCGCTCTCCTGTTCCGACCTGCCGCTTACCGGATACCTGTC
CGCCTTTCTCCCTTCGGGAAGCNNNCGCTTTCTCATAGCTCACGCTGTAGGTATCTCAGTTCGGNNTAGGTCN

MADS221

>525-pTOPO-MADS221-colony-3-M13F_G05.ab1

NNNNNNNNNNNNNNGGCGATTGNTTTAGCGGCCGCGAATTCGCCCTTATGGCCAATGAAAACAAATCCTTGTCAAT
CGACTCTCCCCAGAGAAAATTGGGTAGGGGAAAGATCGAGATCAAAGCGGATCGAAAACACGACCAATCGTCAAGT
GACCTTCTGCAAGAGGCGCAATGGGTTGCTCATAGAAGGCCTATGAACTCTCTGTGCTCTGTGATGCAGAGGTTGCT
CTCATAGTCTTCTCTAACCGTGGCCGCCTCTATGAGTATGCCAACAACAGAAGGGCGAATTCGTTTAAACCTGCAGGA
CTAGTCCCTTTAGTGAGGGTTAATTCTGAGCTTGGCGTAATCATGGTCATAGCTGTTTCCTGTGTGAAATTGTTATCCG
CTCACAATTCCACACAACATACGAGCCGGAAGCATAAAGTGTAAAGCCTGGGGTGCCTAATGAGTGAGCTAACTCAC
ATTAATTGCGTTGCGCTCACTGCCCGCTTTCCAGTCGGGAAACCTGTCGTGCCAGCTGCATTAATGAATCGGCCAACG
CGCGGGGAGAGGCGGTTTGCGTATTGGGCGCTCTTCCGCTTCCTCGCTCACTGACTCGCTGCGCTCGGTCGTTCGGC
TGCGGCGAGCGGTATCAGCTCACTCAAAGGCGGTAATACGGTTATCCACAGAATCAGGGGATAACGCAGGAAAGAA
CATGTGAGCAAAAGGCCAGCAAAAGGCCAGGAACCGTAAAAAGGCCGCGTTGCTGGCGTTTTTCCATAGGCTCCGC
CCCCCTGACGAGCATCACAAAAATCGACGCTCAAGTCAGAGGTGGCGAAACCCGACAGGACTATAAAGATACCAGG
CGTTTCCCCCTGNAAGCTCCCTCGTGCGCTCTCCTGTTCCGACCCTGCCGCTTACCGGATACCTGTCCGCCTTTCTCCC

TTCGGGAAGCGTGGCGCTTTCTCATAGCTCACGCTGTANGTATCTCAGTTCGGTGTAGGTCGTTCGCTCCAAGCTGG
GCTGTGTGCACGAACCCCCCGTTCAGCCCGACCGCTGCGCCTNATCCGGTAACTATCGTCTNGAGTCCNNCCCGGTA
NANACGACTTATCGCCACTGGNAGCAGCCACTGNNNACAGGANN

>525-pTOPO-MADS221-colony-7-M13F_H05.ab1

NNNNNNNNNNNNNGGCGATTGNNTTAGCGGCCGCGAATTCGCCCTTCTGTTGTTGGCATACTCATAGAGGCGGCC
ACGGTTAGAGAAGACTATGAGAGCAACCTCTGCATCACAGAGCACAGAGAGTTCATAGGCCTTCTTTGAGCAACCC
ATTGCGCCTCTTGCAGAAGGTCACTTGACGATTGGTCGTGTTTTCGATCCGCTTCTCGATCTTTCCCCTACCCAATTTT
CTCTGGGGAGAGTCGATTGACAAGGATTTGTTTTCATTGGCCATAAGGGCGAATTCGTTTAAACCTGCAGGACTAGT
CCCTTTAGTGAGGGTTAATTCTGAGCTTGGCGTAATCATGGTCATAGCTGTTTCCTGTGTGAAATTGTTATCCGCTCAC
AATTCCACACAACATACGAGCCGGAAGCATAAAGTGTAAAGCCTGGGGTGCCTAATGAGTGAGCTAACTCACATTAA
TTGCGTTGCGCTCACTGCCCGCTTTCCAGTCGGGAAACCTGTCGTGCCAGCTGCATTAATGAATCGGCCAACGCGCG
GGGAGAGGCGGTTTGCGTATTGGGCGCTCTTCCGCTTCCTCGCTCACTGACTCGCTGCGCTCGGTCGTTCGGCTGCG
GCGAGCGGTATCAGCTCACTCAAAGGCGGTAATACGGTTATCCACAGAATCAGGGGATAACGCAGGAAAGAACATG
TGAGCAAAAGGCCAGCAAAAGGCCAGGAACCGTAAAAAGGCCGCGTTGCTGGCGTTTTTCCATAGGCTCCGCCCCC
CTGACGAGCATCACAAAAATCGACGCTCAAGTCAGANGTGGCGAAACCCGACAGGACTATAAAGATACCAGGCGTT
TCCCCCTGGAAGCTCCCTCGTGCGCTCTCCTGTTCCGACCCTGCCGCTTACCGGATACCTGTCCGCCTTTCTCCCTTCG
GGAAGCGTGGCGCTTTCTCATAGCTCACGCTGTAGGTATCTCAGTTCGGTGTNNTCGTTCGCTCCAAGCTGGGCTGT
GTGCACGANCCCCCGTTCAGCCNNACCGCTGCGCCTNATCCNGGTAACTATCGTCTTGAGTCCAACCCNGGTAANA
NACGACTTATCN

Event 526

<u>MADS15</u>

>pTOPO-526-MADS15-colony-4-M13F

NNNNNNNNNNNNNGGNNNNNGGGCCCTCTAGATGCATGCTCGAGCGGCCGCCAGTGTGATGGATATCTGCAGA
ATTCGCCCTTTTTCATTTGTTTCTGCAAGTTTCAAACTTTTTGTTGGTGATCTAGAATATAGTAATTAATTAAAGTTCTG
GGGTTTTTATTTTAATTAGAAAGGTGCTTAATTGATTGATCTCTTTGTTCTTATTGATTCAGCTTCCAACTATGGCCTATG
AAAGCAAATCCTTGTCCTTGGACTCTCCCCAGAGAAAATTGGGTAGGGGAAAGATCGAGATTAAGCGGATCGAAAA
CACAACGAATCGTCAAGTGACCTTCTGCAAGAGGCGCAATGGGTTGCTCAAGAAGGCCTATGAACTCTCTGTGCTCT
GTGATGCAGAGGTTGCTCTCATAGTCTTCTCTAACCGTGGCCGCCTCTATGAGTATGCCAACAATAGGTAATATTATTGC
TTCAATTTACTTGAAGGGCGAATTCCAGCACACTGGCGGCCGTTACTAGTGGATCCGAGCTCGGTACCAAGCTTGAT
GCATAGCTTGAGTATTCTATAGTGTCACCTAAATAGCTTGGCGTAATCATGGTCATAGCTGTTTCCTGTGTGAAATTGTT
ATCCGCTCACAATTCCACACAACATACGAGCCGGAAGCATAAAGTGTAAAGCCTGGGGTGCCTAATGAGTGAGCTAA
CTCACATTAATTGCGTTGCGCTCACTGCCCGCTTTCCAGTCGGGAAACCTGTCGTGCCAGCTGCATTAATGAATCGGC
CAACGCGCGGGGAGAGGNGGTTTGCGTATTGGGCGCTCTTCCGCTTCCTCGCTCACTGACTCGCTGCGCTCGGTCG
TTCGGCTGCGGCGAGNNGTATCAGCTCACTCAANNN

>pTOPO-526-MADS15-colony-1-
M13FNNNNNNNNNNNTNNGGCGATTGGGCCCTCTAGATGCATGCTCGAGCGGCCGCCAGTGTGATGGATATCTGCA
GAATTCGCCCTTTTCATTTGTTTCTGCAAGTTTCAAACTTTTTGTTGGTGATCTAGAATATAGTAATTAATTAAAGTTCT
GGGGTTTTTATTTTAATTAGAAAGGTGCTTAATTGATTGATCTCTTTGTTCTTATTGATTCAGCTTCCAACTATGGCCTAT
GAAAGCAAATCCTTGTCCTTGGACTCTCCCCAGAGAAAATTGGGTAGGGGAAAGATCGAGATTAAGCGGATCGAAA
ACACAACGAATCGTCAAGTGACCTTCTGCAAGAGGCGCAATGGGTTGCTCAAGAAGGCCTATGAACTCTCTGTGCTC
TGTGATGCAGAGGTTGCTCTCATAGTCTTCTCTAACCGTGGCCGCCTCTATGAGTATGCCAACAATAGGTAATATTATTG
CTTCAATTTACTTGAAGGGCGAATTCCAGCACACTGGCGGCCGTTACTAGTGGATCCGAGCTCGGTACCAAGCTTGA
TGCATAGCTTGAGTATTCTATAGTGTCACCTAAATAGCTTGGCGTAATCATGGTCATAGCTGTTTCCTGTGTGAAATTGT
TATCCGCTCACAATTCCACACAACATACGAGCCGGAAGCATAAAGTGTAAAGCCTGGGGTGCCTAATGAGTGAGCTA
ACTCACATTAATTGCGTTGCGCTCACTGCCCGCTTTCCAGTCGGGAAACCTGTCGTGCCAGCTGCATTAATGAATCGG
CCAACGCGCGGGGAGAGGCGGTTTGCGTATTGGGCGCTCTTCCGCTTCCTCGCTCACTGACTCGCTGCGCTCGGTC
GTTCGGNN

MADS221

>pTOPO_MADS221_526_colony_5-M13F_E10.ab1

NNNNNNNNNNGGGCGATTGGGCCCTCTAGATGCATGCTCGAGCGGCCGCCAGTGTGATGGATATCTGCAGAATTC
GCCCTTATGGCCAATGAAAACAAATCCTTGTCAATCGACTCTCCCCAGAGAAAATTGGGTAGGGGAAAGATCGAGAT
CAAGCGGATCGAAAACACGACCAATCGTCAAGTGACCTTCTGCAAGAGGCGCAATGGGTTGCTCAAGAAGGCCTAT
GAACTCTCTGTGCTCTGTGATGCAGAGGTTGCTCTCATAGTCTTCTCTAACCGTGGCCGCCTCTATGAGTATGCCAACA
ACAGAAGGGCGAATTCCAGCACACTGGCGGCCGTTACTAGTGGATCCGAGCTCGGTACCAAGCTTGATGCATAGCTT
GAGTATTCTATAGTGTCACCTAAATAGCTTGGCGTAATCATGGTCATAGCTGTTTCCTGTGTGAAATTGTTATCCGCTCA
CAATTCCACACAACATACGAGCCGGAAGCATAAAGTGTAAAGCCTGGGGTGCCTAATGAGTGAGCTAACTCACATTA
ATTGCGTTGCGCTCACTGCCCGCTTTCCAGTCGGGAAACCTGTCGTGCCAGCTGCATTAATGAATCGGCCAACGCGC
GGGGAGAGGCGGTTTGCGTATTGGGCGCTCTTCCGCTTCCTCGCTCACTGACTCGCTGCGCTCGGTCGTTCGGCTGC
GGCGAGCGGTATCAGCTCACTCAAAGGCGGTAATACGNTTATCCACAGAATCNNGGANNNGCNNNAGANATGTGA
GCAAAANGCCAGCNNNNNNGNANNTAAAANNNCGTNGCTGNCGTTTTTCATAGNCTCGCCCCCCNGACNAGCAT
CAN

>526-pTOPO-MADS221-colony-1-M13F_E05.ab1

NNNNNNNNNNNNNANGNCGATTGGGCCCTCTAGATGCATGCTCGAGCGGCCGCCAGTGTGATGGATATCTGCAGA
ATTCGCCCTTATGGCCAATGAAAACAAATCCTTGTCAATCGACTCTCCCCAGAGAAAATTGGGTAGGGGAAAGATCG
AGATCAAGCGGATCGAAAACACGACCAATCGTCAAGTGACCTTCTGCAAGAGGCGCAATGGGTTGCTCAAGAAGGC
CTATGAACTCTCTGTGCTCTGTGATGCAGAGGTTGCTCTCATAGTCTTCTCTAACCGTGGCCGCCTCTATGAGTATGCC
AACAACAGAAGGGCGAATTCCAGCACACTGGCGGCCGTTACTAGTGGATCCGAGCTCGGTACCAAGCTTGATGCAT
AGCTTGAGTATTCTATAGTGTCACCTAAATAGCTTGGCGTAATCATGGTCATAGCTGTTTCCTGTGTGAAATTGTTATCC
GCTCACAATTCCACACAACATACGAGCCGGAAGCATAAAGTGTAAAGCCTGGGGTGCCTAATGAGTGAGCTAACTCA
CATTAATTGCGTTGCGCTCACTGCCCGCTTTCCAGTCGGGAAACCTGTCGTGCCAGCTGCATTAATGAATCGGCCAAC
GCGCGGGGAGAGGCGGTTTGCGTATTGGGCGCTCTTCCGCTTCCTCGCTCACTGACTCGCTGCGCTCGGTCGTTCG
GCTGCGGCGAGCGGTATCAGCTCACTCAAAGGCGGTAATACGGTTATCCACAGAATCAGGGGATAACGCAGGAAAG

AACATGTGAGCAAAAGGCCAGCAAAAGGCCAGGAACCGTAAAAAGGCCGCGTTGCTGGCGTTTTTCCATANGCTCC
GCCCCCCTGACGAGCATCACAAAAATCGACGCTCAAGTCAGAGGTGGCGAAACCCGACAGGACTATAAAGATACCA
GGCGTTTCCCCCNNGAAGCTCCCTCGTGCGCTCTCCTGTTCCGACCCTGCCGCTTACCGGATACCTGTCCGCCTTTCT
CCCTTCGGGAAGCGTGNNCTTTCTCNNAGCTCACGCTGNANGTATCTCAGTTCGGNGTAGGTCGTTCGCNNCNAG
CTGGNNGNGTGCACGAACCCCCCNNNANCCCGACGCTGCNNNNTCNGNAACTATCGTCNTGANNCNN

Event 527

MADS15

>527-pTOPO-MADS15-colony-A-M13F_C05.ab1

NNNNNNNNNNNGNNGCGANTGGGCCCTCTAGATGCATGCTCGAGCGGCCGCCAGTGTGATGGATATCTGCAGAA
TTCGCCCTTCTATTGTTGGCATACTCATAGAGGCGGCCACGGTTAGAGAAGACTATGAGAGCAACCTCTGCATCACAG
AGCACAGAGAGTTCATAGGCCTTCTTTGAGCAACCCATTGCGCCTCTTGCAGAAGGTCACTTGACGATTCGTTGTGT
TTTCGATCCGCTTAATCTCGATCTTTCCCCTACCCAATTTTCTCTGGGGAGAGTCCAAGGACAAGGATTTGCTTTCATA
GGCCATAAGGGCGAATTCCAGCACACTGGCGGCCGTTACTAGTGGATCCGAGCTCGGTACCAAGCTTGATGCATAGC
TTGAGTATTCTATAGTGTCACCTAAATAGCTTGGCGTAATCATGGTCATAGCTGTTTCCTGTGTGAAATTGTTATCCGCT
CACAATTCCACACAACATACGAGCCGGAAGCATAAAGTGTAAAGCCTGGGGTGCCTAATGAGTGAGCTAACTCACAT
TAATTGCGTTGCGCTCACTGCCCGCTTTCCAGTCGGGAAACCTGTCGTGCCAGCTGCATTAATGAATCGGCCAACGCG
CGGGGAGAGGCGGTTTGCGTATTGGGCGCTCTTCCGCTTCCTCGCTCACTGACTCGCTGCGCTCGGTCGTTCGGCTG
CGGCGAGCGGTATCAGCTCACTCAAAGGCGGTAATACGGTTATCCACAGAATCAGGGGATAACGCAGGAAAGAACA
TGTGAGCAAAAGGCCAGCAAAAGGCCAGGAACCGTAAAAAGGCCGCGTTGCTGGCGTTTTTCCATAGGCTCCGCC
CCCCTGACGAGCATCACAAAAATCGACGCTCAAGTCAGAGGTGGCGAAACCCGACAGGACTATAAAGATACCAGGC
GTTTCCCCCTGGAAGCTCCCTCGTGCGCTCTCCTGTTCCGACCCTGCCGCTTACCGGATACCTGTCCGCCTTTCTCCCT
TCGGGAAGCGTGNGCTTNCTCATAGCTCACGCTGTNNNATCTCANTTCGGNGTAGTCGTTCNNCTNCNNCTGGGCT
GTGTGCACGACCCCCNNNAGCCCGACGNNNCNCCTTATCCGGTACTATCGTCNTGANTCCAANCCGGNANNNN

>527-pTOPO-MADS15-colony-B-M13F_D05.ab1

NNNNNNNNNNNNNNNNNNGATTGGGCCNTCTAGATGCATGCTCGAGCGGCCGCCAGTGTGATGGATATCTGCAG
AATTCGCCCTTCTATTGTTGGCATACTCATAGAGGCGGCCACGGTTAGAGAAGACTATGAGAGCAACCTCTGCATCAC
AGAGCACAGAGAGTTCATAGGCCTTCTTTGAGCAACCCATTGCGCCTCTTGCAGAAGGTCACTTGACGATTCGTTGT
GTTTTCGATCCGCTTAATCTCGATCTTTCCCCTACCCAATTTTCTCTGGGGAGAGTCCAAGGACAAGGATTTGCTTTCA
TAGGCCATAAGGGCGAATTCCAGCACACTGGCGGCCGTTACTAGTGGATCCGAGCTCGGTACCAAGCTTGATGCATA
GCTTGAGTATTCTATAGTGTCACCTAAATAGCTTGGCGTAATCATGGTCATAGCTGTTTCCTGTGTGAAATTGTTATCCG
CTCACAATTCCACACAACATACGAGCCGGAAGCATAAAGTGTAAAGCCTGGGGTGCCTAATGAGTGAGCTAACTCAC
ATTAATTGCGTTGCGCTCACTGCCCGCTTTCCAGTCGGGAAACCTGTCGTGCCAGCTGCATTAATGAATCGGCCAACG
CGCGGGGAGAGGCGGTTTGCGTATTGGGCGCTCTTCCGCTTCCTCGCTCACTGACTCGCTGCGCTCGGTCGTTCGGC
TGCGGCGAGCGGTATCAGCTCACTCAAAGGCGGTAATACGGTTATCCACAGAATCAGGGGATAACGCAGGAAAGAA
CATGTGAGCAAAAGGCCAGCAAAAGGCCAGGAACCGTAAAAAGGCCGCGTTGCTGGCGTTTTTCCATAGGCTCCGC
CCCCCTGACGAGCATCACAAAAATCGACGCTCAAGTCAGAGGTGGCGAAACCCGACAGGACTATAAAGATACCAGG
CGTTTCCCCCTGGNAGCTCCCTCGTGCGCTCTCCTGTTCCGACCCTGCCGCTTACCGGATACCTGTCCGCCTTTCTCCC

TTCGGGAAGCGTGGCGCTTTCTCATAGCTCACGCTGTNNNATCTCANTTCGGNGTAGNCGTTCGCNNCNAGCTGGG
NTGNGNGCACGACCCCCNNNANCCCGACNNNNNCGCCTNATCCNGNAACTANTCGTCNNGANNNCNNN

MADS221

>pTOPO_MADS221_527_colony_4-M13F_C08.ab1

NNNNNNNNNGGGCGATTGGGCCCTCTAGATGCATGCTCGAGCGGCCGCCAGTGTGATGGATATCTGCAGAATTCG
CCCTTATGGCCAATGAAAACAAATCCTTGTCAATCGACTCTCCCCAGAGAAAATTGGGTAGGGGAAAGATCGAGATC
AGAAGGCCTATGAACTCTCTGTGCTCTGTGATGCAGAGGTTGCTCTCATAGTCTTCTCTAACCGTGGCCGCCTCTATGA
GTATGCCAACAACAGAAGGGCGAATTCCAGCACACTGGCGGCCGTTACTAGTGGATCCGAGCTCGGTACCAAGCTT
GATGCATAGCTTGAGTATTCTATAGTGTCACCTAAATAGCTTGGCGTAATCATGGTCATAGCTGTTTCCTGTGTGAAATT
GTTATCCGCTCACAATTCCACACAACATACGAGCCGGAAGCATAAAGTGTAAAGCCTGGGGTGCCTAATGAGTGAGC
TAACTCACATTAATTGCGTTGCGCTCACTGCCCGCTTTCCAGTCGGGAAACCTGTCGTGCCAGCTGCATTAATGAATC
GGCCAACGCGCGGGGAGAGGCGGTTTGCGTATTGGGCGCTCTTCCGCTTCCTCGCTCACTGACTCGCTGCGCTCGG
TCGTTCGGCTGCGGCGAGCGGTATCAGCTCACTCAAAGGCGGTAATACGGTTATCCACAGAATCNGGGGATAACGCA
NGAAAGAACATGTGAGCAAAAGGNCAGCAAAAGGCCAGGAACCGTAAAAAGGCCNCGTTTNCTGGCGTTTTTCC
ATAGGCTCCGCCCCCCNGACNAGCATCACAAANTCGACGCTCNAGTCANANNNGNGAAACCCGACAGGACTATAA
GNTACCNNNNNTTTCCCCCNGNNNGCNNCCNTNNNNNNNNCNNNNNN

>pTOPO_MADS221_527_colony_6-M13F_D08.ab1

NNNNNNNNNNGGGCGATTGGGCCCTCTAGATGCATGCTCGAGCGGCCGCCAGTGTGATGGATATCTGCAGAATTC
GCCCTTATGGCCAATGAAAACAAATCCTTGTCAATCGACTCTCCCCAGAGAAAATTGGGTAGGGGAAAGATCGAAAA
CACGACCAATCGTCAAGTGACCTTCTGCAAGAGGCGCAATGGGTTGCTCAAAGAAGGCCTATGAACTCTCTGTGCTC
TGTGATGCAGAGGTTGCTCTCATAGTCTTCTCTAACCGTGGCCGCCTCTATGAGTATGCCAACAACAGAAGGGCGAAT
TCCAGCACACTGGCGGCCGTTACTAGTGGATCCGAGCTCGGTACCAAGCTTGATGCATAGCTTGAGTATTCTATAGTG
TCACCTAAATAGCTTGGCGTAATCATGGTCATAGCTGTTTCCTGTGTGAAATTGTTATCCGCTCACAATTCCACACAACA
TACGAGCCGGAAGCATAAAGTGTAAAGCCTGGGGTGCCTAATGAGTGAGCTAACTCACATTAATTGCGTTGCGCTCA
CTGCCCGCTTTCCAGTCGGGAAACCTGTCGTGCCAGCTGCATTAATGAATCGGCCAACGCGCGGGGAGAGGCGGTT
TGCGTATTGGGCGCTCTTCCGCTTCCTCGCTCACTGACTCGCTGCGCTCGGTCGTTCGGCTGCGGCGAGCGGTATCA
GCTCACTCAAAGGCGGTAATACGGTTATCCACAGAATCNGGGGATAACGCNNNAAAGAACATGTGAGCAAAAGGC
CAGCAAAAGGCCAGGAACCGTAAAAANCNCGTTTGCTGGCNTTTTTNCATAGGCTCNCCCCCCNGACNAGCATCAC
AAAATCGACGCTCAAGNCANNNGNTNGNCGAAANCCCGACNNGGNNN

Event 528

MADS15

>pTOPO-528-MAD15-colony-7-M13F

NNNNNNNNNNNNAGGGCGATTGGGCCCTCTAGATGCATGCTCGAGCGGCCGCCAGTGTGATGGATATCTGCAGAA
TTCGCCCTTTTTCATTTGTTTCTGCAAGTTTCAAACTTTTTGTTGGTGATCTAGAATATAGTAATTAATTAAAGTTCTGG
GGTTTTTATTTTAATTAGAAAGGTGCTTAATTGATTGATCTCTTTGTTCTTATTGATTCAGCTTCCAACTATGGCCTATGA

AAGCAAATCCTTGTCCTTGGACTCTCCCCAGAGAAAATTGGGTAGGGGAAAGATCGAGATTAAGCGGATCGAAAAC
ACAACGAATCGTCAAGTGACCTTCTGCAAGAGGCGCAATGGGTTGCTCAAGAAGGCCTATGAACTCTCTGTGCTCTG
TGATGCAGAGGTTGCTCTCATAGTCTTCTCTAACCGTGGCCGCCTCTATGAGTATGCCAACAATAGGTAATATTATTGCT
TCAATTTACTTGAAGGGCGAATTCCAGCACACTGGCGGCCGTTACTAGTGGATCCGAGCTCGGTACCAAGCTTGATG
CATAGCTTGAGTATTCTATAGTGTCACCTAAATAGCTTGGCGTAATCATGGTCATAGCTGTTTCCTGTGTGAAATTGTTA
TCCGCTCACAATTCCACACAACATACGAGCCGGAAGCATAAAGTGTAAAGCCTGGGGTGCCTAATGAGTGAGCTAAC
TCACATTAATTGCGTTGCGCTCACTGCCCGCTTTCCAGTCGGGAAACCTGTCGTGCCAGCTGCATTAATGAATCGGCC
AACGCGCGGGGAGAGGCGGTTTGCGTATTGGGCGCTCTTCCGCTTCCTCGCTCACTGACTCGCTGCGCTCGGTCGTT
CGGCTGCGGCGAGCNGTATCAGNN

>pTOPO-528-MAD15-colony-1-M13F

NNNNNNNNNNNNNGGGCGATTGGGCCCTCTAGATGCATGCTCGAGCGGCCGCCAGTGTGATGGATATCTGCAGA
ATTCGCCCTTTTTCATTTGTTTCTGCAAGTTTCAAACTTTTTGTTGGTGATCTAGAATATAGTAATTAATTAAAGTTCTG
GGGTTTTTATTTTAATTAGAAAGGTGCTTAATTGATTGATCTCTTTGTTCTTATTGATTCAGCTTCCAACTATGGCCTATG
AAAGCAAATCCTTGTCCTTGGACTCTCCCCAGAGAAAATTGGGTAGGGGAAAGATCGAGATTAAGCGGATCGAAAA
CACAACGAATCGTCAAGTGACCTTCTGCAAGAGGCGCAATGGGTTGGGCCTATGAACTCTCTGTGCTCTGTGATGCA
GAGGTTGCTCTCATAGTCTTCTCTAACCGTGGCCGCCTCTATGAGTATGCCAACAATAGGTAATATTATTGCTTCAATTT
ACTTGAAGGGCGAATTCCAGCACACTGGCGGCCGTTACTAGTGGATCCGAGCTCGGTACCAAGCTTGATGCATAGCT
TGAGTATTCTATAGTGTCACCTAAATAGCTTGGCGTAATCATGGTCATAGCTGTTTCCTGTGTGAAATTGTTATCCGCTC
ACAATTCCACACAACATACGAGCCGGAAGCATAAAGTGTAAAGCCTGGGGTGCCTAATGAGTGAGCTAACTCACATT
AATTGCGTTGCGCTCACTGCCCGCTTTCCAGTCGGGAAACCTGTCGTGCCAGCTGCATTAATGAATCGGCCAACGCG
CGGGGAGAGGCGGTTTGCGTATTGGGCGCTCTTCCGCTTCCTCGCTCACTGACTCGCTGCGCTCGGTCGTTCGGCTG
CGGCGAGCGGTATCAGCTCACTCAAAGGCGGTAATACGGTTATCCACAGAATCNGGGGATAACGCNNAAAGAACAT
GTGAGCAAAAGGNCAGCAAAAGGNCAGNNN

MADS221

>528-pTOPO-MADS221-colony-1-M13F_E03.ab1

NNNNNNNNNNNNNNNNGGNNANTGGGCCCTCTAGATGCATGCTCGAGCGGCCGCCAGTGTGATGGATATCTGCAG
AATTCGCCCTTCTGTTGTTGGCATACTCATAGAGGCGGCCACGGTTAGAGAAGACTATGAGAGCAACCTCTGCATCAC
AGAGCACAGAGAGTTCATAGGCCTTCTGATCTCGATCTTTCCCCTACCCAATTTTCTCTGGGGAGAGTCGATTGACAA
GGATTTGTTTTCATTGGCCATAAGGGCGAATTCCAGCACACTGGCGGCCGTTACTAGTGGATCCGAGCTCGGTACCA
AGCTTGATGCATAGCTTGAGTATTCTATAGTGTCACCTAAATAGCTTGGCGTAATCATGGTCATAGCTGTTTCCTGTGTG
AAATTGTTATCCGCTCACAATTCCACACAACATACGAGCCGGAAGCATAAAGTGTAAAGCCTGGGGTGCCTAATGAGT
GAGCTAACTCACATTAATTGCGTTGCGCTCACTGCCCGCTTTCCAGTCGGGAAACCTGTCGTGCCAGCTGCATTAATG
AATCGGCCAACGCGCGGGGAGAGGCGGTTTGCGTATTGGGCGCTCTTCCGCTTCCTCGCTCACTGACTCGCTGCGC
TCGGTCGTTCGGCTGCGGCGAGCGGTATCAGCTCACTCAAAGGCGGTAATACGGTTATCCACAGAATCAGGGGATAA
CGCAGGAAAGAACATGTGAGCAAAAGGCCAGCAAAAGGCCAGGAACCGTAAAAAGGCCGCGTTGCTGGCGTTTTT
CCATAGGCTCCGCCCCCCTGACGAGCATCACAAAAATCGACGCTCAAGTCAGAGGTGGCGAAACCCGACAGGACTA
TAAAGATACCAGGCGTTTCCCCCTGGAAGCTCCCTCGTGCGCTCTCCTGTTCCGACCCTGCCGCTTACCGGATACCTG

TCCGCCTTTCTCCCTTCGGGAAGCGTGGCGCTTTCTCATAGCTCACGCTGTAGGTATCTCAGTTCGGTGTAGGTCGTT
CGCTCCAAGCTGGGCTGTGTGCACGAACCCCCCGTTCAGCCCGACCGCTGCGCCTTATCCNGGTAACTATCGTCTTG
ANTCNNCCNGNANACACGACTTATCNCCNCTGGNANCANCNCTGNNANCNGGATTANCNNANCNAG

>528-pTOPO-MADS221-colony-2-M13F_F03.ab1

NNNNNNNNNNNNNNNNNGGCGATTGGGCCCTCTAGATGCATGCTCGAGCGGCCGCCAGTGTGATGGATATCTGC
AGAATTCGCCCTTCTGTTGTTGGCATACTCATAGAGGCGGCCACGGTTAGAGAAGACTATGAGAGCAACCTCTGCAT
CACAGAGCACAGAGAGTTCATAGGCCTTCTTTGAGCAACCCATTGCGCCTCTTGCAGAAGGTCACTTGACGATTGGT
CGTGTTTTCGATCTTTCCCCTACCCAATTTTCTCTGGGGAGAGTCGATTGACAAGGATTTGTTTTCATTGGCCATAAGG
GCGAATTCCAGCACACTGGCGGCCGTTACTAGTGGATCCGAGCTCGGTACCAAGCTTGATGCATAGCTTGAGTATTCT
ATAGTGTCACCTAAATAGCTTGGCGTAATCATGGTCATAGCTGTTTCCTGTGTGAAATTGTTATCCGCTCACAATTCCAC
ACAACATACGAGCCGGAAGCATAAAGTGTAAAGCCTGGGGTGCCTAATGAGTGAGCTAACTCACATTAATTGCGTTG
CGCTCACTGCCCGCTTTCCAGTCGGGAAACCTGTCGTGCCAGCTGCATTAATGAATCGGCCAACGCGCGGGGAGAG
GCGGTTTGCGTATTGGGCGCTCTTCCGCTTCCTCGCTCACTGACTCGCTGCGCTCGGTCGTTCGGCTGCGGCGAGCG
GTATCAGCTCACTCAAAGGCGGTAATACGGTTATCCACAGAATCAGGGGATAACGCAGGAAAGAACATGTGAGCAA
AAGGCCAGCAAAAGGCCAGGAACCGTAAAAAGGCCGCGTTGCTGGCGTTTTTCCATAGGCTCCGCCCCCCTGACGA
GCATCACAAAAATCGACGCTCAAGTCAGANGTGGCGAAACCCGACAGGACTATAAAGATACCAGGCGTTTCCCCCT
GNAAGCTCCCTCGTGCGCTCTCCTGTTCCGACCCTGCCGCTTACCGGATACCTGTCCGCCTTTCTCCCTTCGGGAAGC
GTNNGCTTTCTCATAGCTCACGCTGNAAGGTATCTCANTTCGGNGTAGNCGTCGCNCCNAGCTGGGCTGNGTGCAC
GAACCCCCCCNTNCANCCCGACCGCTGCGCCTTATCCNGGTNNN

Event 529

<u>MADS15</u>

>pTOPO_MADS15_529_colony_2-M13F_H07.ab1

NNNNNNNNNNNGGCGATTGGGCCCTCTAGATGCATGCTCGAGCGGCCGCCAGTGTGATGGATATCTGCAGAATTCG
CCCTTATGGCCTATGAAAGCAAATCCTTGTCCTTGGACTCTCCCCAGAGAAAATTGGGTAGGGGAAAGATCGAGATT
AAGCGGATCGAAAACACAACGAATCGTCAAGTGACCTTCTGCAAGAGGCGCAATGGGTTGCTCTATGAACTCTCTGT
GCTCTGTGATGCAGAGGTTGCTCTCATAGTCTTCTCTAACCGTGGCCGCCTCTATGAGTATGCCAACAATAGAAGGGC
GAATTCCAGCACACTGGCGGCCGTTACTAGTGGATCCGAGCTCGGTACCAAGCTTGATGCATAGCTTGAGTATTCTAT
AGTGTCACCTAAATAGCTTGGCGTAATCATGGTCATAGCTGTTTCCTGTGTGAAATTGTTATCCGCTCACAATTCCACAC
AACATACGAGCCGGAAGCATAAAGTGTAAAGCCTGGGGTGCCTAATGAGTGAGCTAACTCACATTAATTGCGTTGCG
CTCACTGCCCGCTTTCCAGTCGGGAAACCTGTCGTGCCAGCTGCATTAATGAATCGGCCAACGCGCGGGGAGAGGC
GGTTTGCGTATTGGGCGCTCTTCCGCTTCCTCGCTCACTGACTCGCTGCGCTCGGTCGTTCGGCTGCGGCGAGCGGT
ATCAGCTCACTCAAAGGCGGTAATACGGTTATCCACAGAATCNGGGATAACGCNNNNNNNATGTGAGCAAAANNC
AGCAAAAGNCCAGGAACCGTAAAAAGGCCNCGTTGCTGGNCNTTTTTCCATNGGCNNCCNNCCCCCCCNNACNN
AGCN

>pTOPO_MADS15_529_colony_4-M13F_A08.ab1

NNNNNNNNNNGGGCGATTGGGCCCTCTAGATGCATGCTCGAGCGGCCGCCAGTGTGATGGATATCTGCAGAATTC
GCCCTTCTATTGTTGGCATACTCATAGAGGCGGCCACGGTTAGAGAAGACTATGAGAGCAACCTCTGCATCACAGAG
CACAGAGAGTTCATAGGCCTTCTTGATCTCGATCTTTCCCCTACCCAATTTTCTCTGGGGAGAGTCGATTGACAAGGA
TTTGCTTTCATAGGCCATAAGGGCGAATTCCAGCACACTGGCGGCCGTTACTAGTGGATCCGAGCTCGGTACCAAGC
TTGATGCATAGCTTGAGTATTCTATAGTGTCACCTAAATAGCTTGGCGTAATCATGGTCATAGCTGTTTCCTGTGTGAAA
TTGTTATCCGCTCACAATTCCACACAACATACGAGCCGGAAGCATAAAGTGTAAAGCCTGGGGTGCCTAATGAGTGA
GCTAACTCACATTAATTGCGTTGCGCTCACTGCCCGCTTTCCAGTCGGGAAACCTGTCGTGCCAGCTGCATTAATGAA
TCGGCCAACGCGCGGGGAGAGGCGGTTTGCGTATTGGGCGCTCTTCCGCTTCCTCGCTCACTGACTCGCTGCGCTC
GGTCGTTCGGCTGCGGCGAGCGGTATCAGCTCACTCAAAGGCGGTAATACGGTTATCCACAGAATCAGGGGATAAC
GCNNAAAGAACATGTGAGCAAAAGGCCAGCAAAAGGCCAGGAACCGTAAAAAGGCCGCGTTGCTGGCGTTTTTCC
ATAGGCTCCGCCCCCCTGACGAGCATCACAAAAATCGACGCTCAAGTCAGAGGTGGNGAAACCCGNNNGGACTAN
NNAANNAANACCNN

MADS221

>pTOPO_MADS221_529_colony_1-M13F_C07.ab1

NNNNNNNNNNNNNGGCGATTGGGCCCTCTAGATGCATGCTCGAGCGGCCGCCAGTGTGATGGATATCTGCAGAATT
CGCCCTTATGGCCAATGAAAACAAATCCTTGTCAATCGACTCTCCCCAGAGAAAATTGGGTAGGGGAAAGATCGAGA
TCAAGCGGATCGAAAACACGACCAATCGTCAAGTGACCTTCTGCAAGAGGCGCAATGGGTTGCTCAAGAAGGCCTA
TGAACTCTCTGTGCTCTGTGATGCAGAGGTTGCTCTCATAGTCTTCTCTAACCGTGGCCGCCTCTATGAGTATGCCAAC
AACAGAAGGGCGAATTCCAGCACACTGGCGGCCGTTACTAGTGGATCCGAGCTCGGTACCAAGCTTGATGCATAGCT
TGAGTATTCTATAGTGTCACCTAAATAGCTTGGCGTAATCATGGTCATAGCTGTTTCCTGTGTGAAATTGTTATCCGCTC
ACAATTCCACACAACATACGAGCCGGAAGCATAAAGTGTAAAGCCTGGGGTGCCTAATGAGTGAGCTAACTCACATT
AATTGCGTTGCGCTCACTGCCCGCTTTCCAGTCGGGAAACCTGTCGTGCCAGCTGCATTAATGAATCGGCCAACGCG
CGGGGAGAGGCGGTTTGCGTATTGGGCGCTCTTCCGCTTCCTCGCTCACTGACTCGCTGCGCTCGGTCGTTCGGCTG
CGGCGAGCGGTATCAGCTCACTCAAAGGCGGTAATACGGTTATCCACAGAATCANGGGATAACGCANGAAAGAACA
TGTGAGCAAAAGGCCAGCAAANNGNCAGGAACCGTAAAAAGGCCGCGTTGCTGGCGTTTTTCCATAGGCTCCGCC
CCCCTGACGAGCATCACAAAAATCGACGCTCAAGTCAGAGGTGGCGAAACCCGACAGGACTATAAAGATACCAGGC
GTTTCCCCCNGGAAGCTNCCNCGTGCGCTCTNCTGTTCCGACCCTGCNNTNNNNATACNGTCGNNNCNNCCNNN
GGANCNNGNNNTTTCNCANAGCTCACGCTGTNGNANNTCANTNGNNGTNNNNNNNGNNNNCNNCTGGGNNG
NNNNTGCACNAN

>pTOPO_MADS221_529_colony_5-M13F_D07.ab1

NNNNNNNNNNNNNNNNNNNNTGGGCCNTCTAGATGCATGCTCGAGCGGCCGCCAGTGTGATGGATATCTGCAGA
ATTCGCCCTTCTGTTGTTGGCATACTCATAGAGGCGGCCACGGTTAGAGAAGACTATGAGAGCAACCTCTGCATCACA
GAGCACAGAGAGTTCATAGGCCTTCTTTGAGCAACCCATTGCGCCTCTTGCAGAAGGTCACTTGACGATTGGTCGTG
TTTTCGATCTTTCCCCTACCCAATTTTCTCTGGGGAGAGTCGATTGACAAGGATTTGTTTTCATTGGCCATAAGGGCG
AATTCCAGCACACTGGCGGCCGTTACTAGTGGATCCGAGCTCGGTACCAAGCTTGATGCATAGCTTGAGTATTCTATA
GTGTCACCTAAATAGCTTGGCGTAATCATGGTCATAGCTGTTTCCTGTGTGAAATTGTTATCCGCTCACAATTCCACACA
ACATACGAGCCGGAAGCATAAAGTGTAAAGCCTGGGGTGCCTAATGAGTGAGCTAACTCACATTAATTGCGTTGCGC

TCACTGCCCGCTTTCCAGTCGGGAAACCTGTCGTGCCAGCTGCATTAATGAATCGGCCAACGCGCGGGGAGAGGCG
GTTTGCGTATTGGGCGCTCTTCCGCTTCCTCGCTCACTGACTCGCTGCGCTCGGTCGTTCGGCTGCGGCGAGCGGTAT
CAGCTCACTCAAAGGCGGTAATACGGTTATCCACAGAATCAGGGGATAACGCANGAAAGAACATGTGAGCAAAAGG
CCAGCAAAAGGCCANGAACCGTAAAAAGGCCGCGTTGCTGGCGTTTTTCCATAGGCTCCGCCCCCCTGACGAGCAT
CACAAAAATCGACGCTCAAGTCAGAGGTGGCGAAACCCGACAGGACTATAAAGATACCAGGCGTTTCCCCCTGGAA
GCTCCCTCNNGCGCNCTCCTGTTCCGACCTGCCGCTTACCGGGANACCTGTCCGCCTTTCTCCCTNNGGGAANN

Event 530

MADS15

>pTOPO_MADS15_530_colony_5-M13F_B08.ab1

NNNNNNNNNNNNNNNCGATTGGGCCCTCTAGATGCATGCTCGAGCGGCCGCCAGTGTGATGGATATCTGCAGAATT
CGCCCTTATGGCCTATGAAAGCAAATCCTTGTCCTTGGACTCTCCCCAGAGAAAATTGGGTAGGGGAAAGATCGAGA
TTAAGCGGATCGAAAACACAACGAATCGTCAAGTGACCTTCTGCAAGAGGCGCAATGGGTTGCTCTCATAGTCTTCT
CTAACCGTGGCCGCCTCTATGAGTATGCCAACAATAGAAGGGCGAATTCCAGCACACTGGCGGCCGTTACTAGTGGA
TCCGAGCTCGGTACCAAGCTTGATGCATAGCTTGAGTATTCTATAGTGTCACCTAAATAGCTTGGCGTAATCATGGTCA
TAGCTGTTTCCTGTGTGAAATTGTTATCCGCTCACAATTCCACACAACATACGAGCCGGAAGCATAAAGTGTAAAGCC
TGGGGTGCCTAATGAGTGAGCTAACTCACATTAATTGCGTTGCGCTCACTGCCCGCTTTCCAGTCGGGAAACCTGTCG
TGCCAGCTGCATTAATGAATCGGCCAACGCGCGGGGAGAGGCGGTTTGCGTATTGGGCGCTCTTCCGCTTCCTCGCT
CACTGACTCGCTGCGCTCGGTCGTTCGGCTGCGGCGAGCGGTATCAGCTCACTCAAAGGCGGTAATACGGTTATCCA
CAGAATCAGGGGATAACGCANGAAAGAACATGTGAGCAAAAGGCCAGCAAAAGGCCAGGAACCGTAAAAAGGCC
GCGTTGCTGGCGTTTTTCCATAGGCTCCGCCCCCCTGACGAGCATCACAAAAATCGACGCTCAAGTCAGAGGTGGCG
AAACCCGACAGGACTATAAANATACCAGNCGTTTCCCCCTGGAAGCTCCCNCGTGCGCTCTCCTGTTCCGACCCTGC
CGCTTACCGGATACCTGTCCGCCTTTCTCCCNN

>pTOPO_MADS15_530_colony_8-M13F_C08.ab1

NNNNNNNNNNNNNNNGGCGATTGGGCCCTCTAGATGCATGCTCGAGCGGCCGCCAGTGTGATGGATATCTGCAGA
ATTCGCCCTTATGGCCTATGAAAGCAAATCCTTGTCCTTGGACTCTCCCCAGAGAAAATTGGGTAGGGGAAAGATCG
AGATTAAGCGGATCGAAAACACAACGAATCGTCAAGTGACCTTCTGCAAGAGGCGCAATGGGTTGCTCTCATAGTCT
TCTCTAACCGTGGCCGCCTCTATGAGTATGCCAACAATAGAAGGGCGAATTCCAGCACACTGGCGGCCGTTACTAGTG
GATCCGAGCTCGGTACCAAGCTTGATGCATAGCTTGAGTATTCTATAGTGTCACCTAAATAGCTTGGCGTAATCATGGT
CATAGCTGTTTCCTGTGTGAAATTGTTATCCGCTCACAATTCCACACAACATACGAGCCGGAAGCATAAAGTGTAAAG
CCTGGGGTGCCTAATGAGTGAGCTAACTCACATTAATTGCGTTGCGCTCACTGCCCGCTTTCCAGTCGGGAAACCTGT
CGTGCCAGCTGCATTAATGAATCGGCCAACGCGCGGGGAGAGGCGGTTTGCGTATTGGGCGCTCTTCCGCTTCCTCG
CTCACTGACTCGCTGCGCTCGGTCGTTCGGCTGCGGCGAGCGGTATCAGCTCACTCAAAGGCGGTAATACGGTTATC
CACAGAATCAGGGGATAACGCANGAAAGAACATGTGAGCAAAAGGCCAGCAAAAGGCCAGGAACCGTAAAAAGG
CCGCGTTGCTGGCGTTTTTCCATAGGCTCCGCCCCCCTGACGAGCATCACAAAAATCGACGCTCAAGTCAGAGGTGG
CGAAACCCGACAGGACTATAAAGATACCAGGCGTTTCCCCCTGGAAGCTCCNCGTGCGCTCTCCTGTTCCGACCCTG
CCGCTTACCGGATACCTGTCCGCCTTTNTCCTTCGGGANCGTGNNCTTTCNCATAGCTCANGCTGTAGTATCTCAGTT

CGNNGTAGNCNTNGCTCNGCTGGNTGTGTGCACGACCCCCGTTCANCCCGACNCTNCNNCNNNNCCGGTAACNA
TCN

MADS221

>pTOPO_MADS221_530_colony_4-M13F_A02.ab1

NNNNNNNNNNNNNGGGCGANTGGGCCCTCTAGATGCATGCTCGAGCGGCCGCCAGTGTGATGGATATCTGCAGA
ATTCGCCCTTATGGCCAATGAAAACAAATCCTTGTCAATCGACTCTCCCCAGAGAAAATTGGGTAGGGGAAAGATCG
AGATCAAGAAGGCCTATGAACTCTCTGTGCTCTGTGATGCAGAGGTTGCTCTCATAGTCTTCTCTAACCGTGGCCGCC
TCTATGAGTATGCCAACAACAGAAGGGCGAATTCCAGCACACTGGCGGCCGTTACTAGTGGATCCGAGCTCGGTACC
AAGCTTGATGCATAGCTTGAGTATTCTATAGTGTCACCTAAATAGCTTGGCGTAATCATGGTCATAGCTGTTTCCTGTGT
GAAATTGTTATCCGCTCACAATTCCACACAACATACGAGCCGGAAGCATAAAGTGTAAAGCCTGGGGTGCCTAATGA
GTGAGCTAACTCACATTAATTGCGTTGCGCTCACTGCCCGCTTTCCAGTCGGGAAACCTGTCGTGCCAGCTGCATTAA
TGAATCGGCCAACGCGCGGGGAGAGGCGGTTTGCGTATTGGGCGCTCTTCCGCTTCCTCGCTCACTGACTCGCTGC
GCTCGGTCGTTCGGCTGCGGCGAGCGGTATCAGCTCACTCAAAGGCGGTAATACGGTTATCCACAGAATCAGGGGAT
AACGCAGGAAAGAACATGTGAGCAAAAGGCCAGCAAAAGGCCAGGAACCGTAAAAAGGCCGCGTTGCTGGCGTT
TTTCCATAGGCTCCGCCCCCCTGACGAGCATCACAAAAATCGACGCTCAAGTCAGAGGTGGCGAAACCCGACAGGA
CTATAAAGATACCAGGCGTTTCCCCCTGGAAGCTCCCTCGTGCGCTCTCCTGTTCCGACCCTGCCGCTTACCGGATACC
TGTCCGCCTTTCTCCCTTCGGGAAGCGNNNNGCTTTCTCATAGCTCNNGCTGTAGGTANCTCANTTCGGTGTAGGTC
GTTCGCNCNAGCTGGGCNGNGTGCACGAACCCCCCGTTCAGCCCGACNCTGCNNNNANNNNACTATCGTCNNNA
GTCANCCNGNAANNNACGACTTANCNCCNCNGGNAGCANCNNNNGNNN

>pTOPO_MADS221_530_colony_6-M13F_B02.ab1

NNNNNNNNNNNNGGGCNNTTGGGCCCTCTAGATGCATGCTCGAGCGGCCGCCAGTGTGATGGATATCTGCAGAA
TTCGCCCTTCTGTTGTTGGCATACTCATAGAGGCGGCCACGGTTAGAGAAGACTATGAGAGCAACCTCTGCATCACA
GAGCACAGAGAGTTCATAGGCCTTCTTGAGCAACCCATTGCGCCTCTTGGCAGAAGGTCACTTGACGATTGGTCGTG
TTTTCGATCCGCTTGATCTCGATCTTTCCCCTACCCAATTTTCTCTGGGGAGAGTCGATTGACAAGGATTTGTTTTCATT
GGCCAAAGGGCGAATTCCAGCACACTGGCGGCCGTTACTAGTGGATCCGAGCTCGGTACCAAGCTTGATGCATAGC
TTGAGTATTCTATAGTGTCACCTAAATAGCTTGGCGTAATCATGGTCATAGCTGTTTCCTGTGTGAAATTGTTATCCGCT
CACAATTCCACACAACATACGAGCCGGAAGCATAAAGTGTAAAGCCTGGGGTGCCTAATGAGTGAGCTAACTCACAT
TAATTGCGTTGCGCTCACTGCCCGCTTTCCAGTCGGGAAACCTGTCGTGCCAGCTGCATTAATGAATCGGCCAACGCG
CGGGGAGAGGCGGTTTGCGTATTGGGCGCTCTTCCGCTTCCTCGCTCACTGACTCGCTGCGCTCGGTCGTTCGGCTG
CGGCGAGCGGTATCAGCTCACTCAAAGGCGGTAATACGGTTATCCACAGAATCAGGGGATAACGCANGAAAGAACA
TGTGAGCAAAAGGCCAGCAAAAGGCCAGGAACCGTAAAAAGGCCGCGTTGCTGGCGTTTTTCCATAGGCTCCGCC
CCCCTGACGAGCATCACAAAAATCGACGCTCAAGTCAGAGGTGGCGAAACCCGACAGGACTATAAAGATACCAGGC
GTTTCCCCCTGGNAGCTCCCTCGTGCGCTCTCCTGTTCCGACCCTGCCGCTTACCGGANACCTGTCCGCCTTTCTNCC
NTCGGNAGCGTGNGCTTTCTCATAGCTCACGCTGTNGNATCTCANTCGNNTNGNCGTNGCTCNNCTTGGNNGNN
NNCACGACCCCCNTNCANCCNGACGCNNCNCCNNNNCCGGNNANNNNNCNNNN

Event 531

MADS15

>pTOPO-531-MADS15-colony6-M13F

NNNNNNNNGNNNNNNNNNGGGCCCTCTAGATGCATGCTCGAGCGGCCGCCAGTGTGATGGATATCTGCAGAATTC
GCCCTTTTTCATTTGTTTCTGCAAGTTTCAAACTTTTTGTTGGTGATCTAGAATATAGTAATTAATTAAAGTTCTGGGGT
TTTTATTTTAATTAGAAAGGTGCTTAATTGATTGATCTCTTTGTTCTTATTGATTCAGCTTCCAACTATGGCCTATGAAAG
CAAATCCTTGTCCTTGGACTCTCCCCAGAGAAAATTGGGTAGGGGAAAGATCGAGATTAAGCGGATCGAAAACACA
ACGAATCGTCAAGTGACCTTCTGCAAGAGGCGCAATGGGTTGCTCAAGAAGGCCTATGAACTCTCTGTGCTCTGTGA
TGCAGAGGTTGCTCTCATAGTCTTCTCTAACCGTGGCCGCCTCTATGAGTATGCCAACAATAGGTAATATTATTGCTTCA
ATTTACTTGAAGGGCGAATTCCAGCACACTGGCGGCCGTTACTAGTGGATCCGAGCTCGGTACCAAGCTTGATGCAT
AGCTTGAGTATTCTATAGTGTCACCTAAATAGCTTGGCGTAATCATGGTCATAGCTGTTTCCTGTGTGAAATTGTTATCC
GCTCACAATTCCACACAACATACGAGCCGGAAGCATAAAGTGTAAAGCCTGGGGTGCCTAATGAGTGAGCTAACTCA
CATTAATTGCGTTGCGCTCACTGCCCGCTTTCCAGTCGGGAAACCTGTCGTGCCAGCTGCATTAATGAATCGGCCAAC
GCGCGGGGAGAGGCGGTTTGCGTATTGGGCGCTCTTCCGCTTCCTCGCTCACTGACTCGCTGCGCTCGGTCGTTCG
GCTGCGGCGAGCGGTATCAGCTCACTCAAAGGCGGN

>pTOPO_MADS515_531_colony_4a-M13F_C07.ab1

NNNNNNNNNNNNNNNGGCNNTTGGGCCCTCTAGATGCATGCTCGAGCGGCCGCCAGTGTGATGGATATCTGCAGA
ATTCGCCCTTATGGCCTATGAAAGCAAATCCTTGTCCTTGGACTCTCCCCAGAGAAAATTGGGTAGGGGAAAGATCG
AGATTAGAAGGCCTATGAACTCTCTGTGCTCTGTGATGCAGAGGTTGCTCTCATAGTCTTCTCTAACCGTGGCCGCCTC
TATGAGTATGCCAACAATAGAAGGGCGAATTCCAGCACACTGGCGGCCGTTACTAGTGGATCCGAGCTCGGTACCAA
GCTTGATGCATAGCTTGAGTATTCTATAGTGTCACCTAAATAGCTTGGCGTAATCATGGTCATAGCTGTTTCCTGTGTGA
AATTGTTATCCGCTCACAATTCCACACAACATACGAGCCGGAAGCATAAAGTGTAAAGCCTGGGGTGCCTAATGAGTG
AGCTAACTCACATTAATTGCGTTGCGCTCACTGCCCGCTTTCCAGTCGGGAAACCTGTCGTGCCAGCTGCATTAATGA
ATCGGCCAACGCGCGGGGAGAGGCGGTTTGCGTATTGGGCGCTCTTCCGCTTCCTCGCTCACTGACTCGCTGCGCTC
GGTCGTTCGGCTGCGGCGAGCGGTATCAGCTCACTCAAAGGCGGTAATACGGTTATCCACAGAATCAGGGGATAAC
GCAGGAAAGAACATGTGAGCAAAAGGCCAGCAAAAGGCCAGGAACCGTAAAAAGGCCGCGTTGCTGGCGTTTTTC
CATAGGCTCCGCCCCCCTGACGAGCATCACAAAAATCGACGCTCAAGTCAGAGGTGGCGAAACCCGACAGGACTAT
AAAGATACCAGGCGTTTCCCCCTGGAAGCTCCCTCGTGCGCTCTCCTGTTCCGACCCTGCCGNN

MADS221

>pTOPO-MADS21-531-colony-1-M13F_C08.ab1

NNNNNNNNNNNNNNGGGCGATTGGGCCCTCTAGATGCATGCTCGAGCGGCCGCCAGTGTGATGGATATCTGCAGA
ATTCGCCCTTATGGCCAATGAAAACAAATCCTTGTCAATCGACTCTCCCCAGAGAAAATTGGGTAGGGGAAAGATCG
AGATCAAGCGGATCGAAAACACGACCAATCGTCAAGTGACCTTCTGCAAGAGGCGCAATGGGTTGCTCAAGAAGGC
CTATGAACTCTCTGTGCTCTGTGATGCAGAGGTTGCTCTCATAGTCTTCTCTAACCGTGGCCGCCTCTATGAGTATGCC
AACAACAGAAGGGCGAATTCCAGCACACTGGCGGCCGTTACTAGTGGATCCGAGCTCGGTACCAAGCTTGATGCAT

AGCTTGAGTATTCTATAGTGTCACCTAAATAGCTTGGCGTAATCATGGTCATAGCTGTTTCCTGTGTGAAATTGTTATCC
GCTCACAATTCCACACAACATACGAGCCGGAAGCATAAAGTGTAAAGCCTGGGGTGCCTAATGAGTGAGCTAACTCA
CATTAATTGCGTTGCGCTCACTGCCCGCTTTCCAGTCGGGAAACCTGTCGTGCCAGCTGCATTAATGAATCGGCCAAC
GCGCGGGGAGAGGCGGTTTGCGTATTGGGCGCTCTTCCGCTTCCTCGCTCACTGACTCGCTGCGCTCGGTCGTTCG
GCTGCGGCGAGCGGTATCAGCTCACTCAAAGGCGGTAATACGGTTATCCACAGAATCAGGGGATAACGCAGGAAAG
AACATGTGAGCAAAAGGCCAGCAAAAGGCCAGGAACCGTAAAAAGGCCGCGTTGCTGGCGTTTTTCCATAGGCTCC
GCCCCCCTGACGAGCATCACAAAAATCGACGCTCAAGTCAGANGTGGCGAAACCCGACAGGACTATAAAGATACCA
GGCGTTTCCCCCTGGNAGCTCCCTCGTGCGCTCTCCTGTTCCGACCCTGCCGCTTACCGGATACCTGTCCGCCTTTCT
CCCTTCGGGAAGCGNNGNGCTTTCTCATAGCTCACGCTGTNNNATCTCAGTTNNNGTNNNCNNNGCTCNAGCTGG
GCTGNGTGCACNACCCCCNNNAGCCCGACGCTNCNNNNNCNGTACTATCGTCTNNANTCCANCCGGNAANNNN
NACTNNNCGCCNNNGNNNN

>pTOPO_MADS515_531_colony_3-M13F_E07.ab1

NNNNNNNNNNNNNNNNNGATTGGGCCCTCTAGATGCATGCTCGAGCGGCCGCCAGTGTGATGGATATCTGCAGAA
TTCGCCCTTCTATTGTTGGCATACTCATAGAGGCGGCCACGGTTAGAGAAGACTATGAGAGCAACCTCTGCATCACAG
AGCACAGAGAGTTCATAGGCCTTCTGCAACCCATTGCGCCTCTTGCAGAAGGTCACTTGACGATTCGTTGTGTTTTCG
ATCCGCTTAATCTCGATCTTTCCCCTACCCAATTTTCTCTGGGGAGAGTCGATTGACAAGGATTTGCTTTCATAGGCCA
TAAGGGCGAATTCCAGCACACTGGCGGCCGTTACTAGTGGATCCGAGCTCGGTACCAAGCTTGATGCATAGCTTGAG
TATTCTATAGTGTCACCTAAATAGCTTGGCGTAATCATGGTCATAGCTGTTTCCTGTGTGAAATTGTTATCCGCTCACAAT
TCCACACAACATACGAGCCGGAAGCATAAAGTGTAAAGCCTGGGGTGCCTAATGAGTGAGCTAACTCACATTAATTG
CGTTGCGCTCACTGCCCGCTTTCCAGTCGGGAAACCTGTCGTGCCAGCTGCATTAATGAATCGGCCAACGCGCGGGG
AGAGGCGGTTTGCGTATTGGGCGCTCTTCCGCTTCCTCGCTCACTGACTCGCTGCGCTCGGTCGTTCGGCTGCGGCG
AGCGGTATCAGCTCACTCAAAGGCGGTAATACGGTTATCCACAGAATCANGGGATAACGCANGAAAGAACATGTGA
GCAAAAGGCCAGCAAAAGGCCAGGAACCGTAAAAAGGCCGCGTTGCTGGCGTTTTTCCATAGGCTCCGCCCCCCTG
ACGAGCATCACAAAAATCGACGCTCAAGTCAGAGGTGGCGAAACCCGACAGGACTATAAAGATACCAGGCGTTTCC
CCCNGGNNGCTCCCTCGTGCGCTCTCCTGTTCCGACCCTGCCGNTTANCNGNATACCTGTCCGCNNNN

Event 532

<u>MADS15</u>

>pTOPO_MADS515_532_colony_4a-M13F_H09.ab1

NNNNNNNNNNGGGCGATTGGGCCCTCTAGATGCATGCTCGAGCGGCCGCCAGTGTGATGGATATCTGCAGAATTC
GCCCTTATGGCCTATGAAAGCAAATCCTTGTCAATCGACTCTCCCCAGAGAAAATTGGGTAGGGGAAAGATCGAGAT
CAGAAGGCCTATGAACTCTCTGTGCTCTGTGATGCAGAGGTTGCTCTCATAGTCTTCTCTAACCGTGGCCGCCTCTATG
AGTATGCCAACAATAGAAGGGCGAATTCCAGCACACTGGCGGCCGTTACTAGTGGATCCGAGCTCGGTACCAAGCTT
GATGCATAGCTTGAGTATTCTATAGTGTCACCTAAATAGCTTGGCGTAATCATGGTCATAGCTGTTTCCTGTGTGAAATT
GTTATCCGCTCACAATTCCACACAACATACGAGCCGGAAGCATAAAGTGTAAAGCCTGGGGTGCCTAATGAGTGAGC
TAACTCACATTAATTGCGTTGCGCTCACTGCCCGCTTTCCAGTCGGGAAACCTGTCGTGCCAGCTGCATTAATGAATC
GGCCAACGCGCGGGGAGAGGCGGTTTGCGTATTGGGCGCTCTTCCGCTTCCTCGCTCACTGACTCGCTGCGCTCGG
TCGTTCGGCTGCGGCGAGCGGTATCAGCTCACTCAAAGGCGGTAATACGGTTATCCACAGAATCANGGGATAACGCA

CGAAAGAACATGTGAGCAAAANGCCAGCAAAAGGCCAGGAACCGTAAAAANNCCNCGTTTGCTGGCGTTTTTCCA
TNNNTCGCCCCCCNGACGAGCATCACAAAAATCGACGCTCAANTCAGAGNNGNCGAAACCCGACAGGNCTAN

>pTOPO_MADS515_532_colony_3-M13F_A12.ab1

NNNNNNNNNNNNNNNNNNGCGATTGGGCCCTCTAGATGCATGCTCGAGCGGCCGCCAGTGTGATGGATATCTGC
AGAATTCGCCCTTCTATTGTTGGCATACTCATAGAGGCGGCCACGGTTAGAGAAGACTATGAGAGCAACCTCTGCATC
ACAGAGCACAGAGAGTTCATAGGCCTTCCCATTGCGCCTCTTGCAGAAGGTCACTTGACGATTCGTTGTGTTTTCGAT
CCGCTTAATCTCGATCTTTCCCCTACCCAATTTTCTCTGGGGAGAGTCCAAGGACAAGGATTTGCTTTCATAGGCCATA
AGGGCGAATTCCAGCACACTGGCGGCCGTTACTAGTGGATCCGAGCTCGGTACCAAGCTTGATGCATAGCTTGAGTA
TTCTATAGTGTCACCTAAATAGCTTGGCGTAATCATGGTCATAGCTGTTTCCTGTGTGAAATTGTTATCCGCTCACAATT
CCACACAACATACGAGCCGGAAGCATAAAGTGTAAAGCCTGGGGTGCCTAATGAGTGAGCTAACTCACATTAATTGC
GTTGCGCTCACTGCCCGCTTTCCAGTCGGGAAACCTGTCGTGCCAGCTGCATTAATGAATCGGCCAACGCGCGGGG
AGAGGCGGTTTGCGTATTGGGCGCTCTTCCGCTTCCTCGCTCACTGACTCGCTGCGCTCGGTCGTTCGGCTGCGGCG
AGCGGTATCAGCTCACTCAAAGGCGGTAATACNGTTATCCACAGAATCAGGGGATAACGCNNGAANN

MADS221

>pTOPO-MADS21-532-colony-9-M13F_E08.ab1

NNNNNNNTNNNNAGGGCGATTGGGCCCTCTAGATGCATGCTCGAGCGGCCGCCAGTGTGATGGATATCTGCAGAA
TTCGCCCTTATGGCCAATGAAAACAAATCCTTGTCAATCGACTCTCCCCAGAGAAAATTGGGTAGGGGAAAGATCGA
GAAAGCGGATCGAAAACACGACCAATCGTCAAGTGACCTTCTGCAAGAGGCGCAATGGGTTAAGGCCTATGAACTC
TCTGTGCTCTGTGATGCAGAGGTTGCTCTCATAGTCTTCTCTAACCGTGGCCGCCTCTATGAGTATGCCAACAACAGAA
GGGCGAATTCCAGCACACTGGCGGCCGTTACTAGTGGATCCGAGCTCGGTACCAAGCTTGATGCATAGCTTGAGTAT
TCTATAGTGTCACCTAAATAGCTTGGCGTAATCATGGTCATAGCTGTTTCCTGTGTGAAATTGTTATCCGCTCACAATTC
CACACAACATACGAGCCGGAAGCATAAAGTGTAAAGCCTGGGGTGCCTAATGAGTGAGCTAACTCACATTAATTGCG
TTGCGCTCACTGCCCGCTTTCCAGTCGGGAAACCTGTCGTGCCAGCTGCATTAATGAATCGGCCAACGCGCGGGGA
GAGGCGGTTTGCGTATTGGGCGCTCTTCCGCTTCCTCGCTCACTGACTCGCTGCGCTCGGTCGTTCGGCTGCGGCGA
GCGGTATCAGCTCACTCAAAGGCGGTAATACGGTTATCCACAGAATCAGGGGATAACGCAGGAAAGAACATGTGAG
CAAAAGGCCAGCAAAAGGCCAGGAACCGTAAAAAGGCCGCGTTGCTGGCGTTTTTCCATAGGCTCCGCCCCCCTGA
CGAGCATCACAAAAATCGACGCTCAAGTCAGANGTGGCGAAACCCGACAGGACTATAAAGATACCAGGCGTTTCCC
CCTGGAAGCTCCCTCGTGCGCTCTCCTGTTCCGACCCTGCCGCTTACCGGATACCTGTCCGCCTTTCTCCCTTCGGGA
AGCGTGNGCTTTCTCATAGCTCACGCTGTNGGTATCTCAGTTCGGNGTAGNCNNCGCTCNNCTGGGNTGNGTGCAC
GAACCCCCGTTCANCCCGACGCTGCNNCTTANCNGTAACTATCNNCNTGNNNTCCAACCNNGNAANANNNNANT
N

>pTOPO-MADS21-532-colony-10-M13F_F08.ab1

NNNNNNNNNNNNNNGNNNANNGGGCCCTCTAGATGCATGCTCGAGCGGCCGCCAGTGTGATGGATATCTGCAG
AATTCGCCCTTATGGCCAATGAAAACAAATCCTTGTCAATCGACTCTCCCCAGAGAAAATTGGGTAGGGGAAAGATC

GAGATCAGAAGGCCTATGAACTCTCTGTGCTCTGTGATGCAGAGGTTGCTCTCATAGTCTTCTCTAACCGTGGCCGCC
TCTATGAGTATGCCAACAACAGAAGGGCGAATTCCAGCACACTGGCGGCCGTTACTAGTGGATCCGAGCTCGGTACC
AAGCTTGATGCATAGCTTGAGTATTCTATAGTGTCACCTAAATAGCTTGGCGTAATCATGGTCATAGCTGTTTCCTGTGT
GAAATTGTTATCCGCTCACAATTCCACACAACATACGAGCCGGAAGCATAAAGTGTAAAGCCTGGGGTGCCTAATGA
GTGAGCTAACTCACATTAATTGCGTTGCGCTCACTGCCCGCTTTCCAGTCGGGAAACCTGTCGTGCCAGCTGCATTAA
TGAATCGGCCAACGCGCGGGGAGAGGCGGTTTGCGTATTGGGCGCTCTTCCGCTTCCTCGCTCACTGACTCGCTGC
GCTCGGTCGTTCGGCTGCGGCGAGCGGTATCAGCTCACTCAAAGGCGGTAATACGGTTATCCACAGAATCAGGGGAT
AACGCAGGAAAGAACATGTGAGCAAAAGGCCAGCAAAAGGCCAGGAACCGTAAAAAGGCCGCGTTGCTGGCGTT
TTTCCATAGGCTCCGCCCCCCTGACGAGCATCACAAAAATCGACGCTCAAGTCAGANGTGGCGAAACCCGACAGGA
CTATAAAGATACCAGGCGTTTCCCCCTNGAANCTCCCTCGTGCGCTCTCCTGTTCCGACCCTGCCGCTTACCGGATAC
CTGTCCGCCTTTCTCCCTTCGGGAAGCGTGGCGCTTTCTCATAGCTCACGCTGTAGGNATCTCAGTTCGGTGTAGGTC
GTTCGNNNCANCTGGGCTGTGTGCACGAACCCCCCGTTCAGCCCGACCGCTGCGCCTTATCCGGTACTATCGTCTTG
ANTCCAACCCGGTANANNCGACTTATCNCNCTGNAGCANCCACNGGNANNGGATTANNNGANCNNNNNNTGTA
GNNGGNNGCTACNNANTTNNTNN

Event 533

<u>MADS15</u>

>pTOPO_MADS15_533_colony_5-M13F_H07.ab1

NNNNNNNNNNNNGGCGATTGGGCCCTCTAGATGCATGCTCGAGCGGCCGCCAGTGTGATGGATATCTGCAGAATT
CGCCCTTTATTGTTGGCATACTCATAGAGGCGGCCACGGTTAGAGAAGACTATGAGAGCAACCTCTGCATCACAGAG
CACAGAGAGTTCATAGGCCTTCTTGATCTCGATCTTTCCCCTACCCAATTTTCTCTGGGGAGAGTCGATTGACAAGGA
TTTGCTTTCATAGGCCATAAGGGCGAATTCCAGCACACTGGCGGCCGTTACTAGTGGATCCGAGCTCGGTACCAAGC
TTGATGCATAGCTTGAGTATTCTATAGTGTCACCTAAATAGCTTGGCGTAATCATGGTCATAGCTGTTTCCTGTGTGAAA
TTGTTATCCGCTCACAATTCCACACAACATACGAGCCGGAAGCATAAAGTGTAAAGCCTGGGGTGCCTAATGAGTGA
GCTAACTCACATTAATTGCGTTGCGCTCACTGCCCGCTTTCCAGTCGGGAAACCTGTCGTGCCAGCTGCATTAATGAA
TCGGCCAACGCGCGGGGAGAGGCGGTTTGCGTATTGGGCGCTCTTCCGCTTCCTCGCTCACTGACTCGCTGCGCTC
GGTCGTTCGGCTGCGGCGAGCGGTATCAGCTCACTCAAAGGCGGTAATACGGTTATCCACAGAATCAGGGGATAAC
GCANGAAAGAACATGTGAGCAAAAGGCCAGCAAAAGGCCAGGAACCGTAAAAAGGCCGCGTTGCTGGCGTTTTTC
CATAGGCTCCGCCCCCCTGACGAGCATCACAAAAATCGACGCTCAAGTCAGAGGTGGCGAAACCCGACAGGACTAT
AAAGATACCAGGCGTTTCCCCCNGGAAGCTCCCTCGTGCGCTCTCCTGTTCCGACCCTGCCGCTTACCGGATACCTGT
CCGCCTTTCTCCCTTCGGGAAGNN

>pTOPO_MADS15_533_colony_8-M13F_A08.ab1

NNNNNNNNNNNGGNCGATTGGGCCCTCTAGATGCATGCTCGAGCGGCCGCCAGTGTGATGGATATCTGCAGAATT
CGCCCTTCTATTGTTGGCATACTCATAGAGGCGGCCACGGTTAGAGAAGACTATGAGAGCAACCTCTGCATCACAGA
GCACAGAGAGTTCATAGGCCTTCTTTGAGCAACCCATTGCGCCTCTTGCAGAAGGTCACTTGACGATTCGTTGTGTTT
TCGATCCGCTTAATCTCGATCTTTCCCCTACCCAATTTTCTCTGGGGAGAGTCCAAGGACAAGGATTTGCTTTCATAGG
CCATAAGGGCGAATTCCAGCACACTGGCGGCCGTTACTAGTGGATCCGAGCTCGGTACCAAGCTTGATGCATAGCTT
GAGTATTCTATAGTGTCACCTAAATAGCTTGGCGTAATCATGGTCATAGCTGTTTCCTGTGTGAAATTGTTATCCGCTCA
CAATTCCACACAACATACGAGCCGGAAGCATAAAGTGTAAAGCCTGGGGTGCCTAATGAGTGAGCTAACTCACATTA

ATTGCGTTGCGCTCACTGCCCGCTTTCCAGTCGGGAAACCTGTCGTGCCAGCTGCATTAATGAATCGGCCAACGCGC
GGGGAGAGGCGGTTTGCGTATTGGGCGCTCTTCCGCTTCCTCGCTCACTGACTCGCTGCGCTCGGTCGTTCGGCTGC
GGCGAGCGGTATCAGCTCACTCAAAGGCGGTAATACGGTTATCCACAGAATCANGGGATAACGCANGAAAGAACAT
GTGAGCAAAAGGCCAGCAAAAGGCCAGGAACCGTAAAAAGGNCGCGTTGCTGGCGTTTTTCCATNGGCTCCGCCC
CCCTGACGAGCATCACAAAAATCGACGCTCAAGTCAGAGNTGGCGAAACCCGACAGGACTATAAAGATACCAGGCG
TTTCCCCCNGGNNGCTCCCTCGTGCGCTCTCCTGTTCCGACCTGCNCTTANNNATACTGTCNNNTCTCCTNCGGGNA
GCNNNGNNCTTTCTCATAGCTCANGCTGTAGGTATCTCANTTCGGNNNTA

MADS221

>pTOPO-MADS-21-533-colony-9-M13F_A08.ab1

NNNNNNNNNNNNGGGCGATTGGGCCCTCTAGATGCATGCTCGAGCGGCCGCCAGTGTGATGGATATCTGCAGAAT
TCGCCCTTATGGCCAATGAAAACAAATCCTTGTCAATCGACTCTCCCCAGAGAAAATTGGGTAGGGGAAAGATCGAG
ATCAAGAAGGCCTATGAACTCTCTGTGCTCTGTGATGCAGAGGTTGCTCTCATAGTCTTCTCTAACCGTGGCCGCCTCT
ATGAGTATGCCAACAACAGAAGGGCGAATTCCAGCACACTGGCGGCCGTTACTAGTGGATCCGAGCTCGGTACCAA
GCTTGATGCATAGCTTGAGTATTCTATAGTGTCACCTAAATAGCTTGGCGTAATCATGGTCATAGCTGTTTCCTGTGTGA
AATTGTTATCCGCTCACAATTCCACACAACATACGAGCCGGAAGCATAAAGTGTAAAGCCTGGGGTGCCTAATGAGTG
AGCTAACTCACATTAATTGCGTTGCGCTCACTGCCCGCTTTCCAGTCGGGAAACCTGTCGTGCCAGCTGCATTAATGA
ATCGGCCAACGCGCGGGGAGAGGCGGTTTGCGTATTGGGCGCTCTTCCGCTTCCTCGCTCACTGACTCGCTGCGCTC
GGTCGTTCGGCTGCGGCGAGCGGTATCAGCTCACTCAAAGGCGGTAATACGGTTATCCACAGAATCAGGGGATAAC
GCAGGAAAGAACATGTGAGCAAAAGGCCAGCAAAAGGCCAGGAACCGTAAAAAGGCCGCGTTGCTGGCGTTTTTC
CATAGGCTCCGCCCCCCTGACGAGCATCACAAAAATCGACGCTCAAGTCAGAGGTGGCGAAACCCGACAGGACTAT
AAAGATACCAGGCGTTTCCCCCTGGAAGCTCCCTCGTGCGCTCTCCTGTTCCGACCCTGCCGCTTACCGGATACCTGT
CCGCCTTTCTCCCTTCGGGAAGCGTGGCGCTTTCTCATAGCTCACGCTGTAGGTATCTCAGTTCGGTGTNNTCGTTCG
CTCCNNGCTGGGCTGTGTGCACGAACCCCCCGTTCAGCCCGACCGCTGCNNNNNNNNANTATCGTCTNAGTCNNC
NGNANANNCGACTTATCNCACTGNNNCANCCACNGGNAACNGGATNNCNNANNNANGNNNNNN

>pTOPO-MADS-21-533-colony-10-M13F_B08.ab1

NNNNNNNNNNNNANNGGCGATTGGGCCCTCTAGATGCATGCTCGAGCGGCCGCCAGTGTGATGGATATCTGCAGAATTC
GCCCTTATGGCCAATGAAAACAAATCCTTGTCAATCGACTCTCCCCAGAGAAAATTGGGTAGGGGAAAGATCGAGATCAAG
AAGGCCTATGAACTCTCTGTGCTCTGTGATGCAGAGGTTGCTCTCATAGTCTTCTCTAACCGTGGCCGCCTCTATGAGTATG
CCAACAACAGAAGGGCGAATTCCAGCACACTGGCGGCCGTTACTAGTGGATCCGAGCTCGGTACCAAGCTTGATGCATAG
CTTGAGTATTCTATAGTGTCACCTAAATAGCTTGGCGTAATCATGGTCATAGCTGTTTCCTGTGTGAAATTGTTATCCGCTCA
CAATTCCACACAACATACGAGCCGGAAGCATAAAGTGTAAAGCCTGGGGTGCCTAATGAGTGAGCTAACTCACATTAATTG
CGTTGCGCTCACTGCCCGCTTTCCAGTCGGGAAACCTGTCGTGCCAGCTGCATTAATGAATCGGCCAACGCGCGGGGAGA
GGCGGTTTGCGTATTGGGCGCTCTTCCGCTTCCTCGCTCACTGACTCGCTGCGCTCGGTCGTTCGGCTGCGGCGAGCGGT
ATCAGCTCACTCAAAGGCGGTAATACGGTTATCCACAGAATCAGGGGATAACGCAGGAAAGAACATGTGAGCAAAAGGCC
AGCAAAAGGCCAGGAACCGTAAAAAGGCCGCGTTGCTGGCGTTTTTCCATAGGCTCCGCCCCCCTGACGAGCATCACAAA
AATCGACGCTCAAGTCAGANGTGGCGAAACCCGACAGGACTATAAAGATACCAGGCGTTTCCCCCTGGAAGCTCCCTCGT
GCGCTCTCCTGTTCCGACCCTGCCGCTTACCGGATACCTGTCCGCCTTTCTCCCTTCGGGAAGCGTGGCGCTTTCTCATAGC
TCACGCTGTAGGNATCTCAGTTCGGTGTNNTCGTTCGCTCCAAGCTGGGCTGTGTGCACGAACCCCCCGTTCAGCCCGAC
CGCTGCGCNTATCCGGTANTATCGTCTGAGTCCAACCCGGTANACACGACTATCNCNCTGNAGCANCNCTNNNNCNGAT
NNCANANCNNNNNNNNNAGGNGGNGCTNNNNANNNNN

Event 534

MADS15

>pTOPO-MADS15-534-colony-2-M13F_B11.ab1

NNNNNNNNNNNNNNGGGCGATTGGGCCCTCTAGATGCATGCTCGAGCGGCCGCCAGTGTGATGGATATCTGCAGAATTC
GCCCTTATGGCCTATGAAAGCAAATCCTTGTCAATCGACTCTCCCCAGAGAAAATTGGGTAGGGGAAAGATCGAGATAGAA
GGCCTATGAACTCTCTGTGCTCTGTGATGCAGAGGTTGCTCTCATAGTCTTCTCTAACCGTGGCCGCCTCTATGAGTATGCC
AACAATAGAAGGGCGAATTCCAGCACACTGGCGGCCGTTACTAGTGGATCCGAGCTCGGTACCAAGCTTGATGCATAGCTT
GAGTATTCTATAGTGTCACCTAAATAGCTTGGCGTAATCATGGTCATAGCTGTTTCCTGTGTGAAATTGTTATCCGCTCACAA
TTCCACACAACATACGAGCCGGAAGCATAAAGTGTAAAGCCTGGGGTGCCTAATGAGTGAGCTAACTCACATTAATTGCGT
TGCGCTCACTGCCCGCTTTCCAGTCGGGAAACCTGTCGTGCCAGCTGCATTAATGAATCGGCCAACGCGCGGGGAGAGGC
GGTTTGCGTATTGGGCGCTCTTCCGCTTCCTCGCTCACTGACTCGCTGCGCTCGGTCGTTCGGCTGCGGCGAGCGGTATCA
GCTCACTCAAAGGCGGTAATACGGTTATCCACAGAATCAGGGGATAACGCAGGAAAGAACATGTGAGCAAAAGGCCAGCA
AAAGGCCAGGAACCGTAAAAAGGCCGCGTTGCTGGCGTTTTTCCATAGGCTCCGCCCCCCTGACGAGCATCACAAAAATCG
ACGCTCAAGTCAGAGGTGGCGAAACCCGACAGGACTATAAAGATACCAGGCGTTTCCCCCTGGAAGCTCCCTCGTGCGCT
CTCCTGTTCCGACCCTGCCGCTTACCGGATACCTGTCCGCCTTTCTCCCTTCGGGAAGCGTGGCGCTTTCTCATAGCTCACG
CTGTAGGTATCTCAGTTCGGTGTAGGTCGTTCGCTCCNNCTGGGCTGNGTGCACNACCCCCCGTTCAGCCCGACNGCTGC
GCCTNNTCCGGTAACTATCGTCTGAGTCCNNCCCGGTAGANACGAN

>pTOPO-MADS15-534-colony-5-M13F_C11.ab1

NNNNNNNNNNNNNNGGGCGATTGGGCCCTCTAGATGCATGCTCGAGCGGCCGCCAGTGTGATGGATATCTGCAGAATTC
GCCCTTCTATTGTTGGCATACTCATAGAGGCGGCCACGGTTAGAGAAGACTATGAGAGCAACCTCTGCATCACAGAGCACA
GAGAGTTCATAGGCCTTCTGCAACCCATTGCGCCTCTTGCAGAAGGTCACTTGACGATTCGTTGTGTTTTCGATCCGCTTAA
TCTCGATCTTTCCCCTACCCAATTTTCTCTGGGGAGAGTCGATTGACAAGGATTTGCTTTCATAGGCCATAAGGGCGAATTC
CAGCACACTGGCGGCCGTTACTAGTGGATCCGAGCTCGGTACCAAGCTTGATGCATAGCTTGAGTATTCTATAGTGTCACC
TAAATAGCTTGGCGTAATCATGGTCATAGCTGTTTCCTGTGTGAAATTGTTATCCGCTCACAATTCCACACAACATACGAGCC
GGAAGCATAAAGTGTAAAGCCTGGGGTGCCTAATGAGTGAGCTAACTCACATTAATTGCGTTGCGCTCACTGCCCGCTTTC
CAGTCGGGAAACCTGTCGTGCCAGCTGCATTAATGAATCGGCCAACGCGCGGGGAGAGGCGGTTTGCGTATTGGGCGCT
CTTCCGCTTCCTCGCTCACTGACTCGCTGCGCTCGGTCGTTCGGCTGCGGCGAGCGGTATCAGCTCACTCAAAGGCGGTAA
TACGGTTATCCACAGAATCAGGGGATAACGCAGGAAAGAACATGTGAGCAAAAGGCCAGCAAAAGGCCAGGAACCGTAAA
AAGGCCGCGTTGCTGGCGTTTTTCCATAGGCTCCGCCCCCCTGACGAGCATCACAAAAATCGACGCTCAAGTCAGANGTG
GCGAAACCCGACAGGACTATAAAGATACCAGGCGTTTCCCCCNNNNAGCTCCCTCGTGCGCTCTCCTGTTCCGACCCTGC
CGCTTACCGGATACCTGTCCGCCTTTCTCCCTTCGGAAGCGTGNGCTTTCTCATAGCTCACGCTGTNGNATCTCAGTTCGG
NGTNGNCGTTCGCTCNAGCTGGGCTGTGNGCACGAACCCCCNTTCANCCCGACCGNN

MADS221

>pTOPO-MADS21-534-colony-6-M13F_A12.ab1
NNNNNNNNNNNNNNGGGCGATTGGGCCCTCTAGATGCATGCTCGAGCGGCCGCCAGTGTGATGGATATCTGCAGAATTC
GCCCTTATGGCCAATGAAAACAAATCCTTGTCAATCGACTCTCCCCAGAGAAAATTGGGTAGGGGAAAGATCGAGATCAGA
AGGCCTATGAACTCTCTGTGCTCTGTGATGCAGAGGTTGCTCTCATAGTCTTCTCTAACCGTGGCCGCCTCTATGAGTATGC

CAACAACAGAAGGGCGAATTCCAGCACACTGGCGGCCGTTACTAGTGGATCCGAGCTCGGTACCAAGCTTGATGCATAGC
TTGAGTATTCTATAGTGTCACCTAAATAGCTTGGCGTAATCATGGTCATAGCTGTTTCCTGTGTGAAATTGTTATCCGCTCAC
AATTCCACACAACATACGAGCCGGAAGCATAAAGTGTAAAGCCTGGGGTGCCTAATGAGTGAGCTAACTCACATTAATTGC
GTTGCGCTCACTGCCCGCTTTCCAGTCGGGAAACCTGTCGTGCCAGCTGCATTAATGAATCGGCCAACGCGCGGGGAGAG
GCGGTTTGCGTATTGGGCGCTCTTCCGCTTCCTCGCTCACTGACTCGCTGCGCTCGGTCGTTCGGCTGCGGCGAGCGGTAT
CAGCTCACTCAAAGGCGGTAATACGGTTATCCACAGAATCAGGGGATAACGCAGGAAAGAACATGTGAGCAAAAGGCCAG
CAAAAGGCCAGGAACCGTAAAAAGGCCGCGTTGCTGGCGTTTTTCCATAGGCTCCGCCCCCCTGACGAGCATCACAAAAAT
CGACGCTCAAGTCAGAGGTGGCGAAACCCGACAGGACTATAAAGATACCAGGCGTTTCCCCCTGNNAGCTCCCTCGTGCG
CTCTCCTGTTCCGACCCTGCCGCTTACCGGATACCTGTCCGCCTTTCTCCCTTCGGGAAGCGTGGCGCTTTCTCATAGCTCA
CGCTGTNNTATCTCAGTTCGNNGTNNNCGTTCGCTCCNAGCTGGGCTGTGTGCACGAACCCCCCGTTCAGCCCGACCGCT
GCGCNTTATCCGGTAACTN

>pTOPO_MADS221_534_colony_4-M13F_D01.ab1

NNNNNNNNNNNNNNGGGCGATTGGGCCCTCTAGATGCATGCTCGAGCGGCCGCCAGTGTGATGGATATCTGCAGAATTC
GCCCTTATGGCCAATGAAAACAAATCCTTGTCAATCGACTCTCCCCAGAGAAAATTGGGTAGGGGAAAGATCGAGATCAGA
AGGCCTATGAACTCTCTGTGCTCTGTGATGCAGAGGTTGCTCTCATAGTCTTCTCTAACCGTGGCCGCCTCTATGAGTATGC
CAACAACAGAAGGGCGAATTCCAGCACACTGGCGGCCGTTACTAGTGGATCCGAGCTCGGTACCAAGCTTGATGCATAGC
TTGAGTATTCTATAGTGTCACCTAAATAGCTTGGCGTAATCATGGTCATAGCTGTTTCCTGTGTGAAATTGTTATCCGCTCAC
AATTCCACACAACATACGAGCCGGAAGCATAAAGTGTAAAGCCTGGGGTGCCTAATGAGTGAGCTAACTCACATTAATTGC
GTTGCGCTCACTGCCCGCTTTCCAGTCGGGAAACCTGTCGTGCCAGCTGCATTAATGAATCGGCCAACGCGCGGGGAGAG
GCGGTTTGCGTATTGGGCGCTCTTCCGCTTCCTCGCTCACTGACTCGCTGCGCTCGGTCGTTCGGCTGCGGCGAGCGGTAT
CAGCTCACTCAAAGGCGGTAATACGGTTATCCACAGAATCAGGGGATAACGCAGGAAAGAACATGTGAGCAAAAGGCCAG
CAAAAGGCCAGGAACCGTAAAAAGGCCGCGTTGCTGGCGTTTTTCCATAGGCTCCGCCCCCCTGACGAGCATCACAAAAAT
CGACGCTCAAGTCAGAGGTGGCGAAACCCGACAGGACTATAAAGATACCAGGCGTTTCCCCCTGGAAGCTCCCTCGTGCG
CTCTCCTGTTCCGACCCTGCCGCTTACCGGATACCTGTCCGCCNTTTCTCCCTTCGGGAAGCGTGNGCTTTCTCATAGCTCA
CGCTGTNNNATCTCAGTTCGGTGTNNNCGTTCGCTCNAGCTGGGNTGTGNGCACGAACCCCCCGTTCANCCNACNGCTG
CNNNNNNNNNTACTATCGNNNNNNTCANNNNNANAANNCGANTTANCNCCNNNNGNNNCAGCCNNNNN

Event 535

<u>MADS15</u>

>pTOPO_MADS15_535_colony_4-M13F_D08.ab1

NNNNNNNNNNNNNNGGGCGATTGGGCCCTCTAGATGCATGCTCGAGCGGCCGCCAGTGTGATGGATATCTGCAGAATTC
GCCCTTATGGCCAATGAAAACAAATCCTTGTCAATCGACTCTCCCCAGAGAAAAGAAGGCCTATGAACTCTCTGTGCTCTG
TGATGCAGAGGTTGCTCTCATAGTCTTCTCTAACCGTGGCCGCCTCTATGAGTATGCCAACAACAGAAGGGCGAATTCCAG
CACACTGGCGGCCGTTACTAGTGGATCCGAGCTCGGTACCAAGCTTGATGCATAGCTTGAGTATTCTATAGTGTCACCTAA
ATAGCTTGGCGTAATCATGGTCATAGCTGTTTCCTGTGTGAAATTGTTATCCGCTCACAATTCCACACAACATACGAGCCGG
AAGCATAAAGTGTAAAGCCTGGGGTGCCTAATGAGTGAGCTAACTCACATTAATTGCGTTGCGCTCACTGCCCGCTTTCCA
GTCGGGAAACCTGTCGTGCCAGCTGCATTAATGAATCGGCCAACGCGCGGGGAGAGGCGGTTTGCGTATTGGGCGCTCTT
CCGCTTCCTCGCTCACTGACTCGCTGCGCTCGGTCGTTCGGCTGCGGCGAGCGGTATCAGCTCACTCAAAGGCGGTAATAC
GGTTATCCACAGAATCAGGGGATAACGCANGAAAGAACATGTGAGCAAAAGGCCAGCAAAAGGCCAGGAACCGTAAAAA
GGCCGCGTTGCTGGCGTTTTTCCATAGGCTCCGCCCCCCTGACGAGCATCACAAAAATCGACGCTCAAGTCAGANGTGGC
GAAACCCGACAGGACTATAAAGATACCAGGCGTTTCCCCCTGGNAGCTCCCTCGTGCGCTCTCCTGTTCCGACCCTGCCGC

TTACCGGATACCTGTCCGCCTTTCTCCCTTCGGGAAGCGTGGCGCTTTCTCATAGCTCACGCTGTNGGTATCTCAGTTCGGT
GTNGNTCGTTCGNNNN

>pTOPO_MADS15_535_colony_5-M13F_E08.ab1

NNNNNNNNNNNNNNGGGCGATTGGGCCCTCTAGATGCATGCTCGAGCGGCCGCCAGTGTGATGGATATCTGCAGAATTC
GCCCTTCTATTGTTGGCATACTCATAGAGGCGGCCACGGTTAGAGAAGACTATGAGAGCAACCTCTGCATCACAGAGCACA
GAGAGTTCATAGGCCTTCTTGAGCAACCCATTGCGCCTCTTGCAGAAGGTCACTTGACGATTCGTTGTGTTTTCGATCCGCT
TAATCTCGATCTTTCCCCTACCCAATTTTCTCTGGGGAGAGTCCAAGGACAAGGATTTGCTTTCATAGGCCATAAGGGCGAA
TTCCAGCACACTGGCGGCCGTTACTAGTGGATCCGAGCTCGGTACCAAGCTTGATGCATAGCTTGAGTATTCTATAGTGTC
ACCTAAATAGCTTGGCGTAATCATGGTCATAGCTGTTTCCTGTGTGAAATTGTTATCCGCTCACAATTCCACACAACATACGA
GCCGGAAGCATAAAGTGTAAAGCCTGGGGTGCCTAATGAGTGAGCTAACTCACATTAATTGCGTTGCGCTCACTGCCCGCT
TTCCAGTCGGGAAACCTGTCGTGCCAGCTGCATTAATGAATCGGCCAACGCGCGGGGAGAGGCGGTTTGCGTATTGGGCG
CTCTTCCGCTTCCTCGCTCACTGACTCGCTGCGCTCGGTCGTTCGGCTGCGGCGAGCGGTATCAGCTCACTCNNGGCGGT
AATACGGTTATCCACAGAATCAGGGGATAACGCANGAAAGAACATGTGAGCAAAAGGNCAGCAAAAGGNCAGGAACCGT
AAAAAGGCCGCGTTGCTGGCGTTTTTCCATAGGCTCCGCCCCCCTGACGAGCATCACAAAAATCGACGCTCAAGTCAGAGN
TGGCGAAACCCGACAGNACTATAAAGATACCAGGCGTTTCCCCCTGGAAGCTCCNCGTGCGCTCTCCTGNTCCGACCCTG
CNCTTACNNATACCTGTCCGCCTTTCTCNNNNNNANNNNGNNCTTTCTCATAGCTCANGCTGTNNNANCTCANTNGGTG
TNNNCNNNGNNNCNAGCTGGGNNNNNN

MADS221

>pTOPO-535-MADS221-colony-1-M13F

NNNNNNNNNGGGCGANTGGGCCCTCTAGATGCATGCTCGAGCGGCCGCCAGTGTGATGGATATCTGCAGAATTCG
CCCTTGTTGGTGATCAAGAATATAGTAATTGAAGTTCTGGGGTTTTTGTTTTATTTAAAAAGGTGCTTAATTGATTGAT
CTCTTTGTTCTTATTGATTCAGCTTGCAACTATGGCCAATGAAAACAAATCCTTGTCAATCGACTCTCCCCAGAGAAAA
TTGGGTAGGGGAAAGATCGAGATCAAGCGGATCGAAAACACGACCAATCGTCAAGTGACCTTCTGCAAGAGGCGC
AATGGGTTGCTCAAGAAGGCCTATGAACTCTCTGTGCTCTGTGATGCAGAGGTTGCTCTCATAGTCTTCTCTAACCGT
GGCCGCCTCTATGAGTATGCCAACAACAGGTAATAATATTATTGCTTCAATTTTCTTGCTAAGGGCGAATTCCAGCACA
CTGGCGGCCGTTACTAGTGGATCCGAGCTCGGTACCAAGCTTGATGCATAGCTTGAGTATTCTATAGTGTCACCTAAAT
AGCTTGGCGTAATCATGGTCATAGCTGTTTCCTGTGTGAAATTGTTATCCGCTCACAATTCCACACAACATACGAGCCG
GAAGCATAAAGTGTAAAGCCTGGGGTGCCTAATGAGTGAGCTAACTCACATTAATTGCGTTGCGCTCACTGCCCGCTT
TCCAGTCGGGAAACCTGTCGTGCCAGCTGCATTAATGAATCGGCCAACGCGCGGGGAGAGGCGGTTTGCGTATTGG
GCGCTCTTCCGCTTCCTCGCTCACTGACTCGCTGCGCTCGGTCGTTCGGCTGCGGCGAGCGGTATCAGCTCACTCAA
AGGCGGTAATACNGTTATCCACAGAATCACGGGATAACGCNNNAAAGAACATGTGAGCAAANNNCAGCAAAAGGN
CAGNAACCGTAAAAAGGNCNCGTTGCTGGCNT

>pTOPO-MADS21-535-colony-2-M13F_F10.ab1

NNNNNNNNNNNNNNGGGCGATTGGGCCCTCTAGATGCATGCTCGAGCGGCCGCCAGTGTGATGGATATCTGCAGAATTC
GCCCTTATGGCCAATGAAAACAAATCCTTGTCAATCGACTCTCCCCAGAGAAAATTGGGTAGGGGAAAGATCGAGATCAAG
CGGATCGAAAACACGACCAATCGTCAAGTGACCTTCTAAGAGGCGCAATGGGTTGCTCAAGAAGGCCTATGAACTCTCTGT

GCTCTGTGATGCAGAGGTTGCTCTCATAGTCTTCTCTAACCGTGGCCGCCTCTATGAGTATGCCAACAACAGAAGGGCGAA
TTCCAGCACACTGGCGGCCGTTACTAGTGGATCCGAGCTCGGTACCAAGCTTGATGCATAGCTTGAGTATTCTATAGTGTC
ACCTAAATAGCTTGGCGTAATCATGGTCATAGCTGTTTCCTGTGTGAAATTGTTATCCGCTCACAATTCCACACAACATACGA
GCCGGAAGCATAAAGTGTAAAGCCTGGGGTGCCTAATGAGTGAGCTAACTCACATTAATTGCGTTGCGCTCACTGCCCGCT
TTCCAGTCGGGAAACCTGTCGTGCCAGCTGCATTAATGAATCGGCCAACGCGCGGGGAGAGGCGGTTTGCGTATTGGGCG
CTCTTCCGCTTCCTCGCTCACTGACTCGCTGCGCTCGGTCGTTCGGCTGCGGCGAGCGGTATCAGCTCACTCAAAGGCGGT
AATACGGTTATCCACAGAATCAGGGGATAACGCAGGAAAGAACATGTGAGCAAAAGGCCAGCAAAAGGCCAGGAACCGTA
AAAAGGCCGCGTTGCTGGCGTTTTTCCATAGGCTCCGCCCCCCTGACGAGCATCACAAAAATCGACGCTCAAGTCAGAGGT
GGCGAAACCCGACAGGACTATAAAGATACCAGGCGTTTCCCCCNNGAAGCTCCCTCGTGCGCTCTCCTGTTCCGACCCTGC
CGCTTACCGGATACCTGTCCGCCTTTCTCCTTCGGGAAGCGTNGNGCTTTCTCATAGCTCACGCTGTAGGTATCTCAGTTCG
GNGTAGNCNNNGCTCNAGCTGGGCTGNNNGCACGAACCCCCCGTTCAGCCCGACGCTGCNCCTTATCCGGNAACTAN

Event 536

<u>MADS15</u>

>pTOPO-536-MADS15-colony-1-M13F

NNNNNNNNNNNGGGNNNNTGGGCCCTCTAGATGCATGCTCGAGCGGCCGCCAGTGTGATGGATATCTGCAGAAT
TCGCCCTTTTTCATTTGTTTCTGCAAGTTTCAAACTTTTTGTTGGTGATCTAGAATATAGTAATTAATTAAAGTTCTGGG
GTTTTTATTTTAATTAGAAAGGTGCTTAATTGATTGATCTCTTTGTTCTTATTGATTCAGCTTCCAACTATGGCCTATGAA
AGCAAATCCTTGTCCTTGGACTCTCCCCAGAGAAAATTGGGTAGGGGAAAGATCGAGATTAAGCGGATCGAAAACA
CAACGAATCGTCAAGTGACCTTCTGCAAGAGGCGCAATGGGTTGCTCAAGAAGGCCTATGAACTCTCTGTGCTCTGT
GATGCAGAGGTTGCTCTCATAGTCTTCTCTAACCGTGGCCGCCTCTATGAGTATGCCAACAATAGGTAATATTATTGCTT
CAATTTACTTGAAGGGCGAATTCCAGCACACTGGCGGCCGTTACTAGTGGATCCGAGCTCGGTACCAAGCTTGATGC
ATAGCTTGAGTATTCTATAGTGTCACCTAAATAGCTTGGCGTAATCATGGTCATAGCTGTTTCCTGTGTGAAATTGTTAT
CCGCTCACAATTCCACACAACATACGAGCCGGAAGCATAAAGTGTAAAGCCTGGGGTGCCTAATGAGTGAGCTAACT
CACATTAATTGCGTTGCGCTCACTGCCCGCTTTCCAGTCGGGAAACCTGTCGTGCCAGCTGCATTAATGAATCGGCCA
ACGCGCGGGGAGAGGCGGTTTGCGTATTGGGCGCTCTTCCGCTTCCTCGCTCACTGACTCGCTGCGCTCGGTCGTTC
NGCTGCGGCNAGCGGTNATCAGCTCACTCANNGGCGGTAATACGGNTANCCNNNNNANNCNGGGGANNAACCN
CANGNAANNNNNNNTTNNNCAAANGCCAGCAAAAGGGCCNGGNAACCGTAAAAANGNCGNCGTTNNCTTGGCN
NTTTTTCCNN

>pTOPO_MADS515_536_colony_2-M13F_H03.ab1

NNNNNNNTNNNNGGCGATTGGGCNNCTAGATGCATGCTCGAGCGGCCGCCAGTGTGATGGATATCTGCAGAATTCGCC
CTTCTATTGTTGGCATACTCATAGAGGCGGCCACGGTTAGAGAAGACTATGAGAGCAACCTCTGCATCACAGAGCACAGAG
AGTTCATAGGCCTTCTTGAGCAACCCATTGCGCCTCTTGCAGAAGGTCACTTGACGATTCGTTGTGTTTTCGATCCGCTTAAT
CTCGATCTTTCCCCTACCCAATTTTCTCTGGGGAGAGTCCAAGGACAAGGATTTGCTTTCATAGGCCATAAGGGCGAATTCC
AGCACACTGGCGGCCGTTACTAGTGGATCCGAGCTCGGTACCAAGCTTGATGCATAGCTTGAGTATTCTATAGTGTCACCT
AAATAGCTTGGCGTAATCATGGTCATAGCTGTTTCCTGTGTGAAATTGTTATCCGCTCACAATTCCACACAACATACGAGCC
GGAAGCATAAAGTGTAAAGCCTGGGGTGCCTAATGAGTGAGCTAACTCACATTAATTGCGTTGCGCTCACTGCCCGCTTTC
CAGTCGGGAAACCTGTCGTGCCAGCTGCATTAATGAATCGGCCAACGCGCGGGGAGAGGCGGTTTGCGTATTGGGCGCT
CTTCCGCTTCCTCGCTCACTGACTCGCTGCGCTCGGTCGTTCGGCTGCGGCGAGCGGTATCAGCTCACTCAAAGGCGGTAA
TACGGTTATCCACAGAATCAGGGGATAACGCANGAAAGAACATGTGAGCAAAAGGCCAGCAAAAGGCCAGGAACCGTAAA

AAGGCCGCGTTGCTGGCGTTTTTCCATAGGCTCCGCCCCCCTGACGAGCATCACAAAAATCGACGCTCAAGTCAGAGGTG
GCGA

MADS221

>pTOPO_MADS221_536_colony_8-M13F_D03.ab1

NNNNNNNNNNNNNNNNGGGCGATTGGGCCCTCTAGATGCATGCTCGAGCGGCCGCCAGTGTGATGGATATCTGCAGAA
TTCGCCCTTATGGCCAATGAAAACAAATCCTTGTCAATCGACTCTCCCCAGAGAAAATTGGGTAGGGGAAAGATCGAGATC
AAGCGGATCGAAAACACGACCAATCGTCAAGTGACCTTCTGCAAGAGGCGCAATGGGTTGCTCAAGAAGGCCTATGAACT
CTCTGTGCTCTGTGATGCAGAGGTTGCTCTCATAGTCTTCTCTAACCGTGGCCGCCTCTATGAGTATGCCAACAACAGAAGG
GCGAATTCCAGCACACTGGCGGCCGTTACTAGTGGATCCGAGCTCGGTACCAAGCTTGATGCATAGCTTGAGTATTCTAT
AGTGTCACCTAAATAGCTTGGCGTAATCATGGTCATAGCTGTTTCCTGTGTGAAATTGTTATCCGCTCACAATTCCACAC
AACATACGAGCCGGAAGCATAAAGTGTAAAGCCTGGGGTGCCTAATGAGTGAGCTAACTCACATTAATTGCGTTGCGCTC
ACTGCCCGCTTTCCAGTCGGGAAACCTGTCGTGCCAGCTGCATTAATGAATCGGCCAACGCGCGGGGAGAGGCGGTTTGC
GTATTGGGCGCTCTTCCGCTTCCTCGCTCACTGACTCGCTGCGCTCGGTCGTTCGGCTGCGGCGAGCGGTATCAGCTCAC
TCAAAGGCGGTAATACGGTTATCCACAGAATCAGGGGATAACGCAGGAAAGAACATGTGAGCAAAAGGCCAGCAAAAGGC
CAGGAACCGTAAAAAGGCCGCGTTGCTGGCGTTTTTCCATAGGCTCCGCCCCCCTGACGAGCATCACAAAAATCGACGCT
CAAGTCAGAGGTGGCGAAACCCGACAGGACTATAAAGATACCAGGCGTTTCCCCCTGGAAGCTCCCTCGTGCGCTCTCCT
GTTCCGACCCTGCCGCTTACCGGATACCTGTCCGCCTTTCTCCCNTNNNGAAGCGTGNGCTTTCTCATAGCTCNNGCTGN
NNNNNNTCAGTCGNNTNGTCNTNGNTCNAGCNGGNNNNNNNTNNNCNACCCCCNNNANNNNCNCTGCNCCNNNN
NNNACTATCNNNNGNNNCNANCNNGTANNNNNNCGACTNNN

>pTOPO_MADS221_536_colony_6-M13F_C03.ab1

NNNNNNNNNNNNNNGGGCGNTTGGGCCCTCTAGATGCATGCTCGAGCGGCCGCCAGTGTGATGGATATCTGCAGAATTC
GCCCTTATGGCCAATGAAAACAAATCCTTGTCAATCGACTCTCCCCAGAGAAAATTGGGTAGGGGAAAGATCGAGATCAAG
CGGATCGAAAACACGACCAATCGTCAAGTGACCTTCTGCAAGAGGCGCAATGGGTTGCTCAAGAAGGCCTATGAACTCTCT
GTGCTCTGTGATGCAGAGGTTGCTCTCATAGTCTTCTCTAACCGTGGCCGCCTCTATGAGTATGCCAACAACAGAAGGGC
GAATTCCAGCACACTGGCGGCCGTTACTAGTGGATCCGAGCTCGGTACCAAGCTTGATGCATAGCTTGAGTATTCTATAG
TGTCACCTAAATAGCTTGGCGTAATCATGGTCATAGCTGTTTCCTGTGTGAAATTGTTATCCGCTCACAATTCCACACAA
CATACGAGCCGGAAGCATAAAGTGTAAAGCCTGGGGTGCCTAATGAGTGAGCTAACTCACATTAATTGCGTTGCGCTCAC
TGCCCGCTTTCCAGTCGGGAAACCTGTCGTGCCAGCTGCATTAATGAATCGGCCAACGCGCGGGGAGAGGCGGTTTGCGT
ATTGGGCGCTCTTCCGCTTCCTCGCTCACTGACTCGCTGCGCTCGGTCGTTCGGCTGCGGCGAGCGGTATCAGCTCACTC
AAAGGCGGTAATACGGTTATCCACAGAATCAGGGGATAACGCANGNNNAACATGTGAGCAAAAGGCCAGCAAAAGGCCA
GGAACCGTAAAAAGGCCGCGTTGCTGGCGTTTTTCCATAGGCTCCGCCCCCCTGACGAGCATCACAAAAATCGACGCTCAA
GTCANAGGTGGCGAAACCCGACAGGACTATAAAGATACCAGGCGTTTCCCCCTGGAAGCTCCCTCGTGCGCTCTCCTGTT
CCGACCCTGCCGCTTACCGGATACCTGTCCGCCTTTCTNCCTTCGGGANCNNNGNNCTTTCTCANAGCTNNNCGCTGTNG
NATNNNCANTNGNGTNGTCNTNGCTCNGCTGGGNNNNNNNNNNCNNACCCCCNNNANNNNCNNCTGNNNNNNNN
CNNACNNNTCNNCTTGNANTCNNNCNNNAANNNNCNNCTTATCNNCCNCNG

Event 537

MADS15

>pTOPO-537-MADS15-colony-1a-M13F

NNNNNNNNNGGGCGATTGGGCCCTCTAGATGCATGCTCGAGCGGCCGCCAGTGTGATGGATATCTGCAGAATTCG
CCCTTTTTCATTTGTTTCTGCAAGTTTCAAACTTTTTGTTGGTGATCTAGAATATAGTAATTAATTAAAGTTCTGGGGTT
TTTATTTTAATTAGAAAGGTGCTTAATTGATTGATCTCTTTGTTCTTATTGATTCAGCTTCCAACTATGGCCTATGAAAGC
AAATCCTTGTCCTTGGACTCTCCCCAGAGAAAATTGGGTAGGGGAAAGATCGAGATTAAGCGGATCGAAAACACAA
CGAATCGTCAAGTGACCTTCTGCAAGAGGCGCAATGGGTTGCTCAAGAAGGCCTATGAACTCTCTGTGCTCTGTGAT
GCAGAGGTTGCTCTCATAGTCTTCTCTAACCGTGGCCGCCTCTATGAGTATGCCAACAATAGGTAATATTATTGCTTCAA
TTTACTTGAAGGGCGAATTCCAGCACACTGGCGGCCGTTACTAGTGGATCCGAGCTCGGTACCAAGCTTGATGCATA
GCTTGAGTATTCTATAGTGTCACCTAAATAGCTTGGCGTAATCATGGTCATAGCTGTTTCCTGTGTGAAATTGTTATCCG
CTCACAATTCCACACAACATACGAGCCGGAAGCATAAAGTGTAAAGCCTGGGGTGCCTAATGAGTGAGCTAACTCAC
ATTAATTGCGTTGCGCTCACTGCCCGCTTTCCAGTCGGGAAACCTGTCGTGCCAGCTGCATTAATGAATCGGCCAACG
CGCGGGGAGAGGCGGTTTGCGTATTGGGCGCTCTTCCGCTTCCTCGCTCACTGACTCGCTGCGCTCGGTCGTTCGGC
TGCGGCGAGCGGTATCAGCTCACTCAAAGGNNTAATANNNTATCCACAGAATCNGGGATAACGCNGAAAGACATGT
GANCAAANNCAGCAAAGNCNGANCGTAAAANNNCGTTGCNGCGTTTTCCATNGCNNCGCCCCCNGACNAGCATC
NNAAAANCGANGCTCAGTNNNN

>pTOPO-MADS15-537-colony-1b-M13F_C12.ab1

NNNNNNNNNNNNNNNGGCGANTGGGCCCTCTAGATGCATGCTCGAGCGGCCGCCAGTGTGATGGATATCTGCAGAATT
CGCCCTTATGGCCTATGAAAGCAAATCCTTGTCCTTGGACTCTCCCCAGAGAAAATTGGGTAGGGGAAAGATCGAGATTAA
GCGGATCGAAAACACAACGAATCGTCAAGTGACCTTCTGCAAGAGGCGCAATGGGTTGCTCAAGAAGGCCTATGAACTCT
CTGTGCTCTGTGATGCAGAGGTTGCTCTCATAGTCTTCTCTAACCGTGGCCGCCTCTATGAGTATGCCAACAATAGAAGGG
CGAATTCCAGCACACTGGCGGCCGTTACTAGTGGATCCGAGCTCGGTACCAAGCTTGATGCATAGCTTGAGTATTCTATAG
TGTCACCTAAATAGCTTGGCGTAATCATGGTCATAGCTGTTTCCTGTGTGAAATTGTTATCCGCTCACAATTCCACACAACAT
ACGAGCCGGAAGCATAAAGTGTAAAGCCTGGGGTGCCTAATGAGTGAGCTAACTCACATTAATTGCGTTGCGCTCACTGCC
CGCTTTCCAGTCGGGAAACCTGTCGTGCCAGCTGCATTAATGAATCGGCCAACGCGCGGGGAGAGGCGGTTTGCGTATTG
GGCGCTCTTCCGCTTCCTCGCTCACTGACTCGCTGCGCTCGGTCGTTCGGCTGCGGCGAGCGGTATCAGCTCACTCAAAGG
CGGTAATACGGTTATCCACAGAATCAGGGGATAACGCAGGAAAGAACATGTGAGCAAAAGGNCAGCAAAAGGNAGGAAC
CGTAAAAAGGCCGCGTTGCTGGCGTTTTTCCATANGCTCCGCCCCCCTGACGAGCATCACAAAAATCGACGCTCAAGTCAG
ANGTGGCGAAACCCGACAGGACTATAAAGATACCAGGCGTTTCCCCCTGGNAGCTCCCTCGTGCGCTCTCCTGTTCCGACC
CTGCCGCTTACCGGATACCTGTCCGCCTTTCTCCCTTCGGGAAGCGTGGCGCTTTCTCATAGCTCACGCTGTAGGNATCTCA
GTTCGGTNTAGGTCGTTCNNCTCCAANN

MADS221

>pTOPO_MADS221_537_colony_4-M13F_F10.ab1

NNNNNNNNNNNNGGCGATTGGGCCCTCTAGATGCATGCTCGAGCGGCCGCCAGTGTGATGGATATCTGCAGAATTCGCC
CTTATGGCCAATGAAAACAAATCCTTGTCAATCGACTCTCCCCAGAGAAAATTGGGTAGGGGAAAGATCGAGATCAAGCGG
ATCGAAAACACGACCAATCGTCAAGTGACCTTCTGCAAGAGGCGCAATGGGTTGCTCAAGAAGGCCTATGAACTCTCTGTG
CTCTGTGATGCAGAGGTTGCTCTCATAGTCTTCTCTAACCGTGGCCGCCTCTATGAGTATGCCAACAACAGAAGGGCGAAT
TCCAGCACACTGGCGGCCGTTACTAGTGGATCCGAGCTCGGTACCAAGCTTGATGCATAGCTTGAGTATTCTATAGTGTCA
CCTAAATAGCTTGGCGTAATCATGGTCATAGCTGTTTCCTGTGTGAAATTGTTATCCGCTCACAATTCCACACAACATACGAG
CCGGAAGCATAAAGTGTAAAGCCTGGGGTGCCTAATGAGTGAGCTAACTCACATTAATTGCGTTGCGCTCACTGCCCGCTT
TCCAGTCGGGAAACCTGTCGTGCCAGCTGCATTAATGAATCGGCCAACGCGCGGGGAGAGGCGGTTTGCGTATTGGGCG

CTCTTCCGCTTCCTCGCTCACTGACTCGCTGCGCTCGGTCGTTCGGCTGCGGCGAGCGGTATCAGCTCACTCAAAGGCGGT
AATACGGTTATCCACAGAATCNNGGGATAACGCNNNAGAACATGTGAGCAAAANNCCAGCAAANNNNNAGNNNNAAAA
ANNNNGTTGCTGGNNTTTTNCATAGGCTCCNCCCCCCNNACNANCATCNNNAAAAANTCNNN

>pTOPO-MADS21-537-colony-7-M13F_G07.ab1
NNNNNNNNNNNNGGGCGATTGGGCCCTCTAGATGCATGCTCGAGCGGCCGCCAGTGTGATGGATATCTGCAGAATTCG
CCCTTATGGCCAATGAAAACAAATCCTTGTCAATCGACTCTCCCCAGAGAAAATTGGGTAGGGGAAAGATCGAGATCAAGC
GGATCGAAAACACGACCAATCGTCAAGTGACCTTCTGCAAGAGGCGCAATGGGTTGCTCAAGAAGGCCTATGAACTCTCTG
TGCTCTGTGATGCAGAGGTTGCTCTCATAGTCTTCTCTAACCGTGGCCGCCTCTATGAGTATGCCAACAACAGAAGGGCGA
ATTCCAGCACACTGGCGGCCGTTACTAGTGGATCCGAGCTCGGTACCAAGCTTGATGCATAGCTTGAGTATTCTATAGTGT
CACCTAAATAGCTTGGCGTAATCATGGTCATAGCTGTTTCCTGTGTGAAATTGTTATCCGCTCACAATTCCACACAACATACG
AGCCGGAAGCATAAAGTGTAAAGCCTGGGGTGCCTAATGAGTGAGCTAACTCACATTAATTGCGTTGCGCTCACTGCCCGC
TTTCCAGTCGGGAAACCTGTCGTGCCAGCTGCATTAATGAATCGGCCAACGCGCGGGGAGAGGCGGTTTGCGTATTGGGC
GCTCTTCCGCTTCCTCGCTCACTGACTCGCTGCGCTCGGTCGTTCGGCTGCGGCGAGCGGTATCAGCTCACTCAAAGGCGG
TAATACGGTTATCCACAGAATCAGGGGATAACGCAGGAAAGAACATGTGAGCAAAAGGCCAGCAAAAGGCCAGGAACCGT
AAAAAGGCCGCGTTGCTGGCGTTTTTCCATAGGCTCCGCCCCCCTGACGAGCATCACAAAAATCGACGCTCAAGTCAGANG
TGGCGAAACCCGACAGGACTATAAAGATACCAGGCGTTTCCCCCTGGAAGCTCCCTCGTGCGCTCTCCTGTTCCGACCCTG
CCGCTTACCGGATACCTGTCCGCCTTTCTCCCTTCGGGAAGCGTGNGCTTTCTCATAGCTCACGCTGNANGTATCTCANTN
NGNGTAGNCNTCGCTCNAGCTGGGNTGNGTGNACGAACCCCCGNTCANCCCGACGCTGCGCCTNATCCGGNAACTANN
GNCTTGNNNN

Event 538

MADS15

>pTOPO-538-MADS15-colony-8-M13F

NNNNNNNNNNNNGATTGGGCCCTCTAGATGCATGCTCGAGCGGCCGCCAGTGTGATGGATATCTGCAGAATTCGC
CCTTTTTCATTTGTTTCTGCAAGTTTCAAACTTTTTGTTGGTGATCTAGAATATAGTAATTAATTAAAGTTCTGGGGTTT
TTATTTTAATTAGAAAGGTGCTTAATTGATTGATCTCTTTGTTCTTATTGATTCAGCTTCCAACTATGGCCTATGAAAGCA
AATCCTTGTCCTTGGACTCTCCCCAGAGAAAATTGGGTAGGGGAAAGATCGAGATTAAGCGGATCGAAAACACAAC
GAATCGTCAAGTGACCTTCTGCAAGAGGCGCAATGGGTTGCAGAAGGCCTATGAACTCTCTGTGCTCTGTGATGCAG
AGGTTGCTCTCATAGTCTTCTCTAACCGTGGCCGCCTCTATGAGTATGCCAACAATAGGTAATATTATTGCTTCAATTTA
CTTGAAGGGCGAATTCCAGCACACTGGCGGCCGTTACTAGTGGATCCGAGCTCGGTACCAAGCTTGATGCATAGCTT
GAGTATTCTATAGTGTCACCTAAATAGCTTGGCGTAATCATGGTCATAGCTGTTTCCTGTGTGAAATTGTTATCCGCTCA
CAATTCCACACAACATACGAGCCGGAAGCATAAAGTGTAAAGCCTGGGGTGCCTAATGAGTGAGCTAACTCACATTA
ATTGCGTTGCGCTCACTGCCCGCTTTCCAGTCGGGAAACCTGTCGTGCCAGCTGCATTAATGAATCGGCCAACGCGC
GGGGAGAGGCGGTTTGCGTATTGGGCGCTCTTCCGCTTCCTCGCTCACTGACTCGCTGCGCTCGGTCGTTCGGCTGC
GGCGAGCGGTATCAGCTCACTCAAAGGN

>pTOPO_MADS515_538_colony_4-M13F_E05.ab1

NNNNNNNNNNNNNNNGGCNNNTGGGCCCTCTAGATGCATGCTCGAGCGGCCGCCAGTGTGATGGATATCTGCAGAATT
CGCCCTTATGGCCTATGAAAGCAAATCCTTGTCCTTGGACTCTCCCCAGAGAAAATTGGGTAGGGGAAAGATCGAGATTAA
GCGGATCGAAAACACAACGAATCGTCAAGTGACCTTCTGCAAGAGGCGCAATGGGTTGCTCAAGAAGGCCTATGAACTCT

CTGTGCTCTGTGATGCAGAGGTTGCTCTCATAGTCTTCTCTAACCGTGGCCGCCTCTATGAGTATGCCAACAATAGAAGGG
CGAATTCCAGCACACTGGCGGCCGTTACTAGTGGATCCGAGCTCGGTACCAAGCTTGATGCATAGCTTGAGTATTCTATAG
TGTCACCTAAATAGCTTGGCGTAATCATGGTCATAGCTGTTTCCTGTGTGAAATTGTTATCCGCTCACAATTCCACACAACAT
ACGAGCCGGAAGCATAAAGTGTAAAGCCTGGGGTGCCTAATGAGTGAGCTAACTCACATTAATTGCGTTGCGCTCACTGCC
CGCTTTCCAGTCGGGAAACCTGTCGTGCCAGCTGCATTAATGAATCGGCCAACGCGCGGGGAGAGGCGGTTTGCGTATTG
GGCGCTCTTCCGCTTCCTCGCTCACTGACTCGCTGCGCTCGGTCGTTCGGCTGCGGCGAGCGGTATCAGCTCACTCAAAGG
CGGTAATACGGTTATCCACAGAATCAGGGGATAACGCANGAAAGAACATGTGAGCAAAAGGCCAGCAAAAGGCCAGGAA
CCGTAAAAAGGCCGCGTTTGCTGGCGTTTTTCCATAGGCTCCGCCCCCCTGACGAGCATCACAAAAATCGACGCTCAAGTC
ANANGTGGCGAAACCCGACAGGACTATAAAGATACCAGGCGT

MADS221

>pTOPO-538-MADS221-colony1-M13F_D02.ab1

NNNNNNNNNNNNNNNGGCGATNGNNNTAGCGGCCGCGAATTCGCCCTTATGGCCAATGAAAACAAATCCTTGTCAATC
GACTCTCCCCAGAGAAAAGAAGGCCTATGAACTCTCTGTGCTCTGTGATGCAGAGGTTGCTCTCATAGTCTTCTCTAACCGT
GGCCGCCTCTATGAGTATGCCAACAACAGAAGGGCGAATTCGTTTAAACCTGCAGGACTAGTCCCTTTAGTGAGGGTTAA
TTCTGAGCTTGGCGTAATCATGGTCATAGCTGTTTCCTGTGTGAAATTGTTATCCGCTCACAATTCCACACAACATACGA
GCCGGAAGCATAAAGTGTAAAGCCTGGGGTGCCTAATGAGTGAGCTAACTCACATTAATTGCGTTGCGCTCACTGCCCGC
TTTCCAGTCGGGAAACCTGTCGTGCCAGCTGCATTAATGAATCGGCCAACGCGCGGGGAGAGGCGGTTTGCGTATTGGGC
GCTCTTCCGCTTCCTCGCTCACTGACTCGCTGCGCTCGGTCGTTCGGCTGCGGCGAGCGGTATCAGCTCACTCAAAGGCG
GTAATACGGTTATCCACAGAATCAGGGGATAACGCAGGAAAGAACATGTGAGCAAAAGGCCAGCAAAAGGCCAGGAACC
GTAAAAAGGCCGCGTTGCTGGCGTTTTTCCATAGGCTCCGCCCCCCTGACGAGCATCACAAAAATCGACGCTCAAGTCAGA
GGTGGCGAAACCCGACAGGACTATAAAGATACCAGGCGTTTCCCCCTGGAAGCTCCCTCGTGCGCTCTCCTGTTCCGACC
CTGCCGCTTACCGGATACCTGTCCGCCTTTCTCCCTTCGGGAAGCGTGGCGCTTTCTCATAGCTCACGCTGTAGGTATCT
CAGTTCGGTGTAGGTCGTTCGCTCCAAGCTGGGCTGTGTGCACGAACCCCCCGTTCAGCCCGACCGCTGCGCCTTATCCG
GTAACTATCGTCTTGAGTCCAACCCGGTAANACACGACTTATCNCNCTGGNAGCANCNCTNNNAACNNNTTAGCANANC
GAGNATGTNGNNGNGCTANNNANTTCTNGAAGNGGNNNNTAACTACNGCTACNCTNN

>pTOPO-538-MADS221-colony2-M13F_E02.ab1

NNNNNNNNNNNNNNGGCGANTGATTTAGCGGCCGCGAATTCGCCCTTCTGTTGTTGGCATACTCATAGAGGCGGCCAC
GGTTAGAGAAGACTATGAGAGCAACCTCTGCATCACAGAGCACAGAGAGTTCATAGGCCTTCTTTGAGCAACCCATTGCGC
CTCTTGCAGAAGGTCACTTGACGATTGGTCGTGTTTTCGATCCGCTTGATCTCGATCTTTCCCCTACCCAATTTTCTCTGGGG
AGAGTCGATTGACAAGGATTTGTTTTCATTGGCCATAAGGGCGAATTCGTTTAAACCTGCAGGACTAGTCCCTTTAGTGAGG
GTTAATTCTGAGCTTGGCGTAATCATGGTCATAGCTGTTTCCTGTGTGAAATTGTTATCCGCTCACAATTCCACACAACATAC
GAGCCGGAAGCATAAAGTGTAAAGCCTGGGGTGCCTAATGAGTGAGCTAACTCACATTAATTGCGTTGCGCTCACTGCCCG
CTTTCCAGTCGGGAAACCTGTCGTGCCAGCTGCATTAATGAATCGGCCAACGCGCGGGGAGAGGCGGTTTGCGTATTGGG
CGCTCTTCCGCTTCCTCGCTCACTGACTCGCTGCGCTCGGTCGTTCGGCTGCGGCGAGCGGTATCAGCTCACTCAAAGGCG
GTAATACGGTTATCCACAGAATCAGGGGATAACGCAGGAAAGAACATGTGAGCAAAAGGCCAGCAAAAGGCCAGGAACC
GTAAAAAGGCCGCGTTGCTGGCGTTTTTCCATAGGCTCCGCCCCCCTGACGAGCATCACAAAAATCGACGCTCAAGTCAGA
GGTGGCGAAACCCGACAGGACTATAAAGATACCAGGCGTTTCCCCCTGGAAGCTCCCTCGTGCGCTCTCCTGTTCCGACCC
TGCCGCTTACCGGATACCTGTCCGCCTTTCTCCCTTCGGGAAGCGTGGCGCTTTCTCATAGCTCACGCTGTAGGTATCTCAG
TTCGGTGTNNNCGTTCGCTCCAAGCTGGGCTGTGTGCACGAACCCCCCGTTCAGCCCGACGCTGCGNCTTATCCGGTAAC
TATCGTCTTGAGTCCAACCCGGTAANAANNNGACTTANNGCCNNTGG

Event 540

MADS15

>pTOPO_MADS515_540_colony_4-M13F_G07.ab1

NNNNNNNNNNNGGGGCGATTGGGCCCTCTAGATGCATGCTCGAGCGGCCGCCAGTGTGATGGATATCTGCAGAATTCG
CCCTTTTTCATTTGTTTCTGCAAGTTTCAAACTTTTTGTTGGTGATCTGGAATATAGTAATTAATTAAAGTTCTGGGGTTTT
TATTTTAATTAGAAAGGTGCTTAATTGATTGATCTCTTTGTTCTTATTGATTCAGTTTCCAACTATGGCCTATGAAAGCA
AATCCTTGTCCATGGACTCTCCCCAGAGAAAATTGGGTAGGGGAAAGATCGAGATTAGAAGGCCTATGAACTCTCTGTGC
TCTGTGATGCAGAGGTTGCTCTCATAGTCTTCTCTAACCGTGGCCGCCTCTATGAGTATGCCAACAATAGGTAATATTAT
TGCTTCAATTTACTTGAAGGGCGAATTCCAGCACACTGGCGGCCGTTACTAGTGGATCCGAGCTCGGTACCAAGCTTGAT
GCATAGCTTGAGTATTCTATAGTGTCACCTAAATAGCTTGGCGTAATCATGGTCATAGCTGTTTCCTGTGTGAAATTGTT
ATCCGCTCACAATTCCACACAACATACGAGCCGGAAGCATAAAGTGTAAAGCCTGGGGTGCCTAATGAGTGAGCTAACTC
ACATTAATTGCGTTGCGCTCACTGCCCGCTTTCCAGTCGGGAAACCTGTCGTGCCAGCTGCATTAATGAATCGGCCAACG
CGCGGGGAGAGGCGGTTTGCGTATTGGGCGCTCTTCCGCTTCCTCGCTCACTGACTCGCTGCGCTCGGTCGTTCGGCTGC
GGCGAGCGGTATCAGCTCACTCAAAGGCGGTAATANNGTTATCCACAGAATCNNGGGATAACGCANGAAAGAACATGTG
AGCAAAANNCAGCAAANGCCAGGAACCGTAAAAAGGCCGCGTTGCTGGCGTTTTTCCATAGGCTCCGCCCCCTGACGAGC
ATCANAANAN

>pTOPO_MADS515_540_colony_5-M13F_H07.ab1

NNNNNNNNNNNNNNNGGGCGATTGGGCCCTCTAGATGCATGCTCGAGCGGCCGCCAGTGTGATGGATATCTGCAGAATT
CGCCCTTTTTCATTTGTTTCTGCAAGTTTCAAACTTTTTGTTGGTGATCTGGAATATAGTAATTAATTAAAGTTCTGGGGTT
TTTATTTTAATTAGAAAGGTGCTTAATTGATTGATCTCTTTGTTCTTATTGATTCAGTTTCCAACTATGGCCTATGAAAG
CAAATCCTTGTCCATGGACTCTCCCCAGAGAAAATTGGGTAGGGGAAAGATCGAGATTAAGCGGATCGAAAACACAACGA
ATCGTCAAGTGACCTTCTGCAAGAGGCGCAATGGGTTGCTCAAGAAGGCCTATGAACTCTCTGTGCTCTGTGATGCAGAG
GTTGCTCTCATAGTCTTCTCTAACCGTGGCCGCCTCTATGAGTATGCCAACAATAGGTAATATTATTGCTTCAATTTACT
TGAAGGGCGAATTCCAGCACACTGGCGGCCGTTACTAGTGGATCCGAGCTCGGTACCAAGCTTGATGCATAGCTTGAGTA
TTCTATAGTGTCACCTAAATAGCTTGGCGTAATCATGGTCATAGCTGTTTCCTGTGTGAAATTGTTATCCGCTCACAATT
CCACACAACATACGAGCCGGAAGCATAAAGTGTAAAGCCTGGGGTGCCTAATGAGTGAGCTAACTCACATTAATTGCGTT
GCGCTCACTGCCCGCTTTCCAGTCAGGAAACCTGTCGTGCCAGCTGCATTAATGAATCGGCCAACGCGCNGGGGANANG
NNNTTGCNNATTGGGCGCTCNTCNCTTCNCGCTCANTGACTCGCTGCGCTCGNNCGTNCGGCTGNGNNNNNNANNAG
CTNNNNCAAAGNNNGTAANNNGGTNNTCNNNNAATCNGGGNTAACCNCNGAANANNNTNNNNNCAAANNNGCAAA
GNNGACNNANNNCCGTTNCTGGNNTTTTCNNNGCNCCNNNCCCNGNNNNNNNNNNNAANNNN

MADS221

>pTOPO_MADS221_540_colony_2-M13F_D03.ab1

NNNNNNNNNNNNNNGGCGATTGGGCCCTCTAGATGCATGCTCGAGCGGCCGCCAGTGTGATGGATATCTGCAGAATTCG
CCCTTATGGCCAATGAAAACAAATCCTTGTCAATCGACTCTCCCCAGAGAAAATTGGGTAGGGGAAAGATCGAGATCAAGC
GGATCGAAAACACGACCAATCGTCAAGTGACCTTCTGCAAGAGGCGCAATGGGTTGCTCAAGAAGGCCTATGAACTCTCTG

TGCTCTGTGATGCAGAGGTTGCTCTCATAGTCTTCTCTAACCGTGGCCGCCTCTATGAGTATGCCAACAACAGAAGGGCG
AATTCCAGCACACTGGCGGCCGTTACTAGTGGATCCGAGCTCGGTACCAAGCTTGATGCATAGCTTGAGTATTCTATAGT
GTCACCTAAATAGCTTGGCGTAATCATGGTCATAGCTGTTTCCTGTGTGAAATTGTTATCCGCTCACAATTCCACACAAC
ATACGAGCCGGAAGCATAAAGTGTAAAGCCTGGGGTGCCTAATGAGTGAGCTAACTCACATTAATTGCGTTGCGCTCACT
GCCCGCTTTCCAGTCGGGAAACCTGTCGTGCCAGCTGCATTAATGAATCGGCCAACGCGCGGGGAGAGGCGGTTTGCGTA
TTGGGCGCTCTTCCGCTTCCTCGCTCACTGACTCGCTGCGCTCGGTCGTTCGGCTGCGGCGAGCGGTATCAGCTCACTCA
AAGGCGGTAATACGGTTATCCACAGAATCAGGGGATAACGCANGAAAGAACATGTGAGCAAAAGGCCAGCAAAAGGCCA
GGAACCGTAAAAAGGCCGCGTTGCTGGCGTTTTTCCATAGGCTCCGCCCCCCTGACGAGCATCACAAAAATCGACGCTCAA
GTCAGAGGTGGCGAAACCCGACAGGACTATN

>pTOPO_MADS221_540_colony_4-M13F_F03.ab1

NNNNNNNNNNNNGGCNATTGGGCCCTCTAGATGCATGCTCGAGCGGCCGCCAGTGTGATGGATATCTGCAGAATTCGC
CCTTATGGCCAATGAAAACAAATCCTTGTCAATCGACTCTCCCCAGAGAAAATTGGGTAGGGGAAAGATCGAGATCAAGCG
GATCGAAAACACGACCAATCGTCAAGTGACCTTCTGCAAGAGGCGCAATGGGTTGCTCAAGAAGGCCTATGAACTCTCTGT
GCTCTGTGATGCAGAGGTTGCTCTCATAGTCTTCTCTAACCGTGGCCGCCTCTATGAGTATGCCAACAACAGAAGGGCGA
ATTCCAGCACACTGGCGGCCGTTACTAGTGGATCCGAGCTCGGTACCAAGCTTGATGCATAGCTTGAGTATTCTATAGTG
TCACCTAAATAGCTTGGCGTAATCATGGTCATAGCTGTTTCCTGTGTGAAATTGTTATCCGCTCACAATTCCACACAACA
TACGAGCCGGAAGCATAAAGTGTAAAGCCTGGGGTGCCTAATGAGTGAGCTAACTCACATTAATTGCGTTGCGCTCACTG
CCCGCTTTCCAGTCGGGAAACCTGTCGTGCCAGCTGCATTAATGAATCGGCCAACGCGCGGGGAGAGGCGGTTTGCGTAT
TGGGCGCTCTTCCGCTTCCTCGCTCACTGACTCGCTGCGCTCGGTCGTTCGGCTGCGGCGAGCGGTATCAGCTCACTCAA
AGGCGGTAATACGGTTATCCACAGAATCANGGGATAACGCNNAAAGAACATGTGAGCAAAAGGCCAGCAAAAGGCCAGG
AACCGTAAAAAGGCCGCGTTGCTGGCGTTTTTCCATAGGCTCCGCCCCCCTGACGAGCATCACAAAAATCGACGCTCAAGT
CAGANNGGCGAAACCCGACAGGAN

Event 542

<u>MADS15</u>

>pTOPO-542-MAD15-colony-8-M13F

NNNNNNNNNNNNNGGGCGNNTTGGGCCCTCTAGATGCATGCTCGAGCGGCCGCCAGTGTGATGGATATCTGCAG
AATTCGCCCTTTTTCATTTGTTTCTGCAAGTTTCAAACTTTTTGTTGGTGATCTGGAATATAGTAATTAATTAAAGTTCT
GGGGTTTTTATTTTAATTAGAAAGGTGCTTAATTGATTGATCTCTTTGTTCTTATTGATTCAGTTTCCAACTATGGCCTAT
GAAAGCAAATCCTTGTCCATGGACTCTCCCCAGAGAAAAAGAGGCGCAATGGGTTGCTCAAGAAGGCCTATGAACT
CTCTGTGCTCTGTGATGCAGAGGTTGCTCTCATAGTCTTCTCTAACCGTGGCCGCCTCTATGAGTATGCCAACAATAGG
TAATATTATTGCTTCAATTTACTTGAAGGGCGAATTCCAGCACACTGGCGGCCGTTACTAGTGGATCCGAGCTCGGTA
CCAAGCTTGATGCATAGCTTGAGTATTCTATAGTGTCACCTAAATAGCTTGGCGTAATCATGGTCATAGCTGTTTCCTGT
GTGAAATTGTTATCCGCTCACAATTCCACACAACATACGAGCCGGAAGCATAAAGTGTAAAGCCTGGGGTGCCTAAT
GAGTGAGCTAACTCACATTAATTGCGTTGCGCTCACTGCCCGCTTTCCAGTCGGGAAACCTGTCGTGCCAGCTGCATT
AATGAATCGGCCAACGCGCGGGGAGAGGCGGTTTGCGTATTGGGCGCTCTTCCGCTTCCTCGCTCACTGACTCGCTG
CGCTCGGTCGTTCGGCTGCGGCGAGCGGTATCAGCTCACTCAAAGGCGGTAATANNGTTATCCACAGAATCANGGG
ATAACGCNNAAAGAACATGTGAGCAAANNNCAGCAAAAGCCAGGAACCGTAAAAAGCCGCGTTGCTGGCGTTTTT
CCATAGCTCCGCCCCCTGACGAGCATC

>pTOPO_MADS515_542_colony_4-M13F_H03.ab1

NNNNNNNNNNNNNNNNGGCNNTTGGGCCCTCTAGATGCATGCTCGAGCGGCCGCCAGTGTGATGGATATCTGCAGAAT
TCGCCCTTATGGCCAATGAAAACAAATCCTTGTCAATCGACTCTCCCCAGAGAAAAAGAAGGCCTATGAACTCTCTGTGCTC
TGTGATGCAGAGGTTGCTCTCATAGTCTTCTCTAACCGTGGCCGCCTCTATGAGTATGCCAACAACAGAAGGGCGAATTCC
AGCACACTGGCGGCCGTTACTAGTGGATCCGAGCTCGGTACCAAGCTTGATGCATAGCTTGAGTATTCTATAGTGTCACC
TAAATAGCTTGGCGTAATCATGGTCATAGCTGTTTCCTGTGTGAAATTGTTATCCGCTCACAATTCCACACAACATACGA
GCCGGAAGCATAAAGTGTAAAGCCTGGGGTGCCTAATGAGTGAGCTAACTCACATTAATTGCGTTGCGCTCACTGCCCGC
TTTCCAGTCGGGAAACCTGTCGTGCCAGCTGCATTAATGAATCGGCCAACGCGCGGGGAGAGGCGGTTTGCGTATTGGGC
GCTCTTCCGCTTCCTCGCTCACTGACTCGCTGCGCTCGGTCGTTCGGCTGCGGCGAGCGGTATCAGCTCACTCAAAGGCG
GTAATACGGTTATCCACAGAATCAGGGGATAACGCAGGAAAGAACATGTGAGCAAAAGGCCAGCAAAAGGCCAGGAACC
GTAAAAAGGCCGCGTTGCTGGCGTTTTTCCATAGGCTCCGCCCCCCTGACGAGCATCACAAAAATCGACGCTCAAGTCAGA
GGTGGCGAAACCCGACAGGACTATAAAGATACCAGGCGTTTCCCCCTGGAAGCTCCCTCGTGCGCTCTCCTGTTCCGACC
CTGCCGCTTACCGGATACCTGTCCGCCTTTCTCCCTTCGGGAAGCGTGNGCTTTCTCATAGCTCACGCTGTAGGTATCTC
AGTTCGGTGTAGGTCGTTCGCTCCAAGCTGGGCTGTGTGCACGANCCCCCNNNCAGCCCGANNCTGCGCCNNNNCCNN
NACNATCGTCNGNNTCANCNGNANANNNGACTANCNCCNCNGNNNCANCNNCNGNNANNNNTTANCANNNCNNNG
NANGNNNGNCGGNNGCNNNNNNANN

<u>MADS221</u>

>pTOPO-542-M221-colony-1-M13F_G05.ab1

NNNNNNNNNNNNNNNNGGCGATTGNNTTTAGCGGCCGCGAATTCGCCCTTCTGTTGTTGGCATACTCATAGAGGCGGCC
AC
GGTTAGAGAAGACTATGAGAGCAACCTCTGCATCACAGAGCACAGAGAGTTCATAGGCCTTCTTGAGCAACCCATTGCGC
CTCTTTTCTCTGGGGAGAGTCGATTGACAAGGATTTGTTTTCATTGGCCATAAGGGCGAATTCGTTTAAACCTGCAGGAC
TAGTCCCTTTAGTGAGGGTTAATTCTGAGCTTGGCGTAATCATGGTCATAGCTGTTTCCTGTGTGAAATTGTTATCCGCT
CACAATTCCACACAACATACGAGCCGGAAGCATAAAGTGTAAAGCCTGGGGTGCCTAATGAGTGAGCTAACTCACATTAA
TTGCGTTGCGCTCACTGCCCGCTTTCCAGTCGGGAAACCTGTCGTGCCAGCTGCATTAATGAATCGGCCAACGCGCGGGG
AGAGGCGGTTTGCGTATTGGGCGCTCTTCCGCTTCCTCGCTCACTGACTCGCTGCGCTCGGTCGTTCGGCTGCGGCGAGC
GGTATCAGCTCACTCAAAGGCGGTAATACGGTTATCCACAGAATCAGGGGATAACGCAGGAAAGAACATGTGAGCAAAAG
GCCAGCAAAAGGCCAGGAACCGTAAAAAGGCCGCGTTGCTGGCGTTTTTCCATAGGCTCCGCCCCCCTGACGAGCATCAC
AAAAATCGACGCTCAAGTCAGANGTGGCGAAACCCGACAGGACTATAAAGATACCAGGCGTTTCCCCCTGNAAGCTCCCT
CGTGCGCTCTCCTGTTCCGACCCTGCCGCTTACCGGATACCTGTCCGCCTTTCTCCCTTCGGGAAGCGTGGCGCTTTCTC
ATAGCTCACGCTGTNNTATCTCAGTTCGGTGTNNTCGTTCGCTCCAAGCTGGGCTGTGTGCACGAACCCCCCGTTCAGCCC
GACCGCTN

>pTOPO-542-M221-colony-6-M13F_H05.ab1

NNNNNNNNNNNNNNGGCGATTGATTTAGCGGCCGCGAATTCGCCCTTATGGCCAATGAAAACAAATCCTTGTCAATCGAC
T
CTCCCCAGAGAAAAGAGGCGCAATGGGTTGCTCAAGAAGGCCTATGAACTCTCTGTGCTCTGTGATGCAGAGGTTGCTCT
CATAGTCTTCTCTAACCGTGGCCGCCTCTATGAGTATGCCAACAACAGAAGGGCGAATTCGTTTAAACCTGCAGGACTAG
TCCCTTTAGTGAGGGTTAATTCTGAGCTTGGCGTAATCATGGTCATAGCTGTTTCCTGTGTGAAATTGTTATCCGCTCAC
AATTCCACACAACATACGAGCCGGAAGCATAAAGTGTAAAGCCTGGGGTGCCTAATGAGTGAGCTAACTCACATTAATTG
CGTTGCGCTCACTGCCCGCTTTCCAGTCGGGAAACCTGTCGTGCCAGCTGCATTAATGAATCGGCCAACGCGCGGGGAGA

GGCGGTTTGCGTATTGGGCGCTCTTCCGCTTCCTCGCTCACTGACTCGCTGCGCTCGGTCGTTCGGCTGCGGCGAGCGGT
ATCAGCTCACTCAAAGGCGGTAATACGGTTATCCACAGAATCAGGGGATAACGCAGGAAAGAACATGTGAGCAAAAGGCC
AGCAAAAGGCCAGGAACCGTAAAAAGGCCGCGTTGCTGGCGTTTTTCCATAGGCTCCGCCCCCCTGACGAGCATCACAAA
AATCGACGCTCAAGTCAGANGTGGCGAAACCCGACAGGACTATAAAGATACCAGGCGTTTCCCCCTGNAAGCTCCCTCGT
GCGCTCTCCTGTTCCGACCCTGCCGCTTACCGGATACCTGTCCGCCTTTCTCCCTTCGGGAAGCGTGGCGCTTTCTCATA
GCTCACGCTGTANNTATCTCAGTTCGGTGTNNNCGTTCGCTCCAAGCTGGGCTGTGTGCACGAA

Event 544

MADS15

>pTOPO-544-MADS15-colony-4-M13F

NNNNNNNNNNNNNNGGGCGATTGGGCCCTCTAGATGCATGCTCGAGCGGCCGCCAGTGTGATGGATATCTGCAGA
ATTCGCCCTTTTTCATTTGTTTCTGCAAGTTTCAAACTTTTTGTTGGTGATCTGGAATATAGTAATTAATTAAAGTTCTG
GGGTTTTTATTTTAATTAGAAAGGTGCTTAATTGATTGATCTCTTTGTTCTTATTGATTCAGTTTCCAACTATGGCCTATG
AAAGCAAATCCTTGTCCATGGACTCTCCCCAGAGAAAATTGGGTAGGGGAAAGATCGAGATAAGAAGGCCTATGAA
CTCTCTGTGCTCTGTGATGCAGAGGTTGCTCTCATAGTCTTCTCTAACCGTGGCCGCCTCTATGAGTATGCCAACAATA
GGTAATATTATTGCTTCAATTTACTTGAAGGGCGAATTCCAGCACACTGGCGGCCGTTACTAGTGGATCCGAGCTCGG
TACCAAGCTTGATGCATAGCTTGAGTATTCTATAGTGTCACCTAAATAGCTTGGCGTAATCATGGTCATAGCTGTTTCCT
GTGTGAAATTGTTATCCGCTCACAATTCCACACAACATACGAGCCGGAAGCATAAAGTGTAAAGCCTGGGGTGCCTA
ATGAGTGAGCTAACTCACATTAATTGCGTTGCGCTCACTGCCCGCTTTCCAGTCGGGAAACCTGTCGTGCCAGCTGCA
TTAATGAATCGGCCAACGCGCGGGGAGAGGCGGTTTGCGTATTGGGCGCTCTTCCGCTTCCTCGCTCACTGACTCGC
TGCGCTCGGTCG

>pTOPO-544-MADS15-colony-1-M13F

NNNNNNNNNNNNNAGGNNNNNNNNGGGCCNTCTAGATGCNTGCTCGAGCGGCCGCCAGTGTGATGGATATCTGCA
GAATTCGCCCTTTTTCATTTGTTTCTGCAAGTTTCAAACTTTTTGTTGGTGATCTAGAATATAGTAATTAATTAAAGTTCT
GGGGTTTTTATTTTAATTAGAAAGGTGCTTAATTGATTGATCTCTTTGTTCTTATTGATTCAGCTTCCAACTATGGCCTAT
GAAAGCAAATCCTTGTCCTTGGACTCTCCCCAGAGAAAATTGGGTAGGGGAAAGATCGAGAAGGCCTATGAACTCT
CTGTGCTCTGTGATGCAGAGGTTGCTCTCATAGTCTTCTCTAACCGTGGCCGCCTCTATGAGTATGCCAACAATAGGTA
ATATTATTGCTTCAATTTACTTGAAGGGCGAATTCCAGCACACTGGCGGCCGTTACTAGTGGATCCGAGCTCGGTACC
AAGCTTGATGCATAGCTTGAGTATTCTATAGTGTCACCTAAATAGCTTGGCGTAATCATGGTCATAGCTGTTTCCTGTGT
GAAATTGTTATCCGCTCACAATTCCACACAACATACGAGCCGGAAGCATAAAGTGTAAAGCCTGGGGTGCCTAATGA
GTGAGCTAACTCACATTAATTGCGTTGCGCTCACTGCCCGCTTTCCAGTCGGGAAACCTGTCGTGCCAGCTGCATTAA
TGAATCGGCCAACGCGCGGGGAGAGGCGGTTTGCGTATTGGGCGCTCTTCCGCTTCCTCGCTCACTGACTCGCTGC
GCTCGGTCGTTCGGCTGCGGCGAGCGGTATCAGCTCACTCAAAGGCGGTAATACGGNTNATCCACAGAATCAGGGG
ATAACGCNNNAAGAACATGT

MADS221

>pTOPO_MADS221_544_colony_2-M13F_H05.ab1

NNNNNNNNNNNNNGGGNNNNNGGGCCCTCTAGATGCATGCTCGAGCGGCCGCCAGTGTGATGGATATCTGCAGAATTC
GCCCTTCTGTTGTTGGCATACTCATAGAGGCGGCCACGGTTAGAGAAGACTATGAGAGCAACCTCTGCATCACAGAGCACA
GAGAGTTCATAGGCCTTCTTGAGCAACCCATTGCGCCTCTTGGCAGAAGGTCACTTGACGATTGGTCGTGTTTTCGATCCG
CTTGATCTCGATCTTTCCCCTACCCAATTTTCTCTGGGGAGAGTCGATTGACAAGGATTTGTTTTCATTGGCCAAAGGGCGA
ATTCCAGCACACTGGCGGCCGTTACTAGTGGATCCGAGCTCGGTACCAAGCTTGATGCATAGCTTGAGTATTCTATAGTGT
CACCTAAATAGCTTGGCGTAATCATGGTCATAGCTGTTTCCTGTGTGAAATTGTTATCCGCTCACAATTCCACACAACATACG
AGCCGGAAGCATAAAGTGTAAAGCCTGGGGTGCCTAATGAGTGAGCTAACTCACATTAATTGCGTTGCGCTCACTGCCCGC
TTTCCAGTCGGGAAACCTGTCGTGCCAGCTGCATTAATGAATCGGCCAACGCGCGGGGAGAGGCGGTTTGCGTATTGGGC
GCTCTTCCGCTTCCTCGCTCACTGACTCGCTGCGCTCGGTCGTTCGGCTGCGGCGAGCGGTATCAGCTCACTCAAAGGCGG
TAATACGGTTATCCACAGAATCAGGGGATAACGCNNGAAAGAACATGTGAGCAAAAGGCCAGCAAAAGGNCAGGAACCG
TAAAAAGGCCGCGTTGCTGGCGTTTTTCCATAGGCTCCGCCCCCCTGACGAGCATCACAAAAATCGACGCTCAAGTCANAG
GTGGCGAAN

>pTOPO_MADS221_544_colony_5-M13F_A06.ab1

NNNNNNNNNNNNNNNNNGATTGGGCCCTCTAGATGCATGCTCGAGCGGCCGCCAGTGTGATGGATATCTGCAGAATTCG
CCCTTATGGCCTATGAAAGCAAATCCTTGTCCTTGGACTCTCCCCAGAGAAAATTGGGTAGGGGAAAGATCGAGATTAAGA
AGGCCTATGAACTCTCTGTGCTCTGTGATGCAGAGGTTGCTCTCATAGTCTTCTCTAACCGTGGCCGCCTCTATGAGTATG
CCAACAATAGAAGGGCGAATTCCAGCACACTGGCGGCCGTTACTAGTGGATCCGAGCTCGGTACCAAGCTTGATGCATAG
CTTGAGTATTCTATAGTGTCACCTAAATAGCTTGGCGTAATCATGGTCATAGCTGTTTCCTGTGTGAAATTGTTATCCGC
TCACAATTCCACACAACATACGAGCCGGAAGCATAAAGTGTAAAGCCTGGGGTGCCTAATGAGTGAGCTAACTCACATTA
ATTGCGTTGCGCTCACTGCCCGCTTTCCAGTCGGGAAACCTGTCGTGCCAGCTGCATTAATGAATCGGCCAACGCGCGGG
GAGAGGCGGTTTGCGTATTGGGCGCTCTTCCGCTTCCTCGCTCACTGACTCGCTGCGCTCGGTCGTTCGGCTGCGGCGAG
CGGTATCAGCTCACTCAAAGGCGGTAATACGGTTATCCACAGAATCAGGGGATAACGCAGGAAAGAACATGTGAGCAAAA
GGCCAGCAAAAGGCCAGGAACCGTAAAAAGGCCGCGTTGCTGGCGTTTTTCCATAGGCTCCGCCCCCCTGACGAGCATCA
CAAAAATCGACGCTCAAGTCAGAGGTGGCGAAACCCGACAGGACTATAAAGATACCAGGCGTTTCCCCCTGGAAGCTCCC
TCGTGCGCTCTCCTGTTCCGAC

Event 546

<u>MADS15</u>

>pTOPO_MADS515_546_colony_1-M13F_C02.ab1

NNNNNNNNNNNNNNNNGGGCNNTTGGGCCCTCTAGATGCATGCTCGAGCGGCCGCCAGTGTGATGGATATCTGCAGAA
TTCGCCCTTATGGCCTATGAAAGCAAATCCTTGTCAATCGACTCTCCCCAGAGAAAATTGGGTAGGGGAAAGATCGAGATA
GAAGGCCTATGAACTCTCTGTGCTCTGTGATGCAGAGGTTGCTCTCATAGTCTTCTCTAACCGTGGCCGCCTCTATGAGTAT
GCCAACAATAGAAGGGCGAATTCCAGCACACTGGCGGCCGTTACTAGTGGATCCGAGCTCGGTACCAAGCTTGATGCATA
GCTTGAGTATTCTATAGTGTCACCTAAATAGCTTGGCGTAATCATGGTCATAGCTGTTTCCTGTGTGAAATTGTTATCCG
CTCACAATTCCACACAACATACGAGCCGGAAGCATAAAGTGTAAAGCCTGGGGTGCCTAATGAGTGAGCTAACTCACATT
AATTGCGTTGCGCTCACTGCCCGCTTTCCAGTCGGGAAACCTGTCGTGCCAGCTGCATTAATGAATCGGCCAACGCGCGG
GGAGAGGCGGTTTGCGTATTGGGCGCTCTTCCGCTTCCTCGCTCACTGACTCGCTGCGCTCGGTCGTTCGGCTGCGGCGA
GCGGTATCAGCTCACTCAAAGGCGGTAATACGGTTATCCACAGAATCAGGGGATAACGCAGGAAAGAACATGTGAGCAAA
AGGCCAGCAAAAGGCCAGGAACCGTAAAAAGGCCGCGTTGCTGGCGTTTTTCCATAGGCTCCGCCCCCCTGACGAGCATC

ACAAAAATCGACGCTCAAGTCAGAGGTGGCGAAACCCGACAGGACTATAAAGATACCAGGCGTTTCCCCCTGGAAGCTCC
CTCGTGCGCTCTCCTGTTCCGACCCTGCCGCTTACCGGATACCTGTCCGCCTTTCTCCCTTCGGGAAGCGNNGNGCTTTC
TCATAGCTCACGCTGTNNNATCTCAGTTCGGTGTAGGTCGTTCGCTCNAGCTGGGNTGTGNGCACGAACCCCCGTTCAGC
CNGACNCTGCNNNNNCNGTACTATCGTCTGAGTCNNCNNNANNNNNACTNNNCNCCACNGGNANCNNCNCNGNAN
CNNGNNTANCANNANNNNNGN

>pTOPO_MADS515_546_colony_3-M13F_D02.ab1

NNNNNNNNNNNTNNNNGNNNNNGGGCCCTCTAGATGCATGCTCGAGCGGCCGCCAGTGTGATGGATATCTGCAGAAT
TCGCCCTTATGGCCTATGAAAGCAAATCCTTGTCCTTGGACTCTCCCCAGAGAAAATTGGGTAGGGGAAAGATCGAGATTA
AGCGGATCGAAAACACAACGAATCGTCAAGTGACCTTCTGCAAGAGGCGCAATGGGTTGCTCAAGAAGGCCTATGAACTC
TCTGTGCTCTGTGATGCAGAGGTTGCTCTCATAGTCTTCTCTAACCGTGGCCGCCTCTATGAGTATGCCAACAATAGAAGGG
CGAATTCCAGCACACTGGCGGCCGTTACTAGTGGATCCGAGCTCGGTACCAAGCTTGATGCATAGCTTGAGTATTCTATA
GTGTCACCTAAATAGCTTGGCGTAATCATGGTCATAGCTGTTTCCTGTGTGAAATTGTTATCCGCTCACAATTCCACACA
ACATACGAGCCGGAAGCATAAAGTGTAAAGCCTGGGGTGCCTAATGAGTGAGCTAACTCACATTAATTGCGTTGCGCTCA
CTGCCCGCTTTCCAGTCGGGAAACCTGTCGTGCCAGCTGCATTAATGAATCGGCCAACGCGCGGGGAGAGGCGGTTTGCG
TATTGGGCGCTCTTCCGCTTCCTCGCTCACTGACTCGCTGCGCTCGGTCGTTCGGCTGCGGCGAGCGGTATCANCTCACT
CAAAGGCGGTAATACGGTTATCCACAGAATCAGGGGATAACGCANGAAAGAACATGTGAGCAAAAGGCCAGCAAAAGGC
CAGGAACCGTAAAAAGGCCGCGTTGCTGGCGTTTTTCCATAGGCTCCGCCCCCCTGACGAGCATCACAAAAATCGACGCTC
AAGTCANAGGTGGCGAAACCCGACAGGACTATAAAGATACCAGGCGTTTCCCCCTGGAAGCTCCCTCGTGCGCTCTCCTG
TTCCGACCCTGCCGCTTACCGGATACCTGTCCGCCTTTCTCCCNTCGGGAAGCGTGNGCTTTNTCANAGCTCANGCTGTA
GNNNCTCANTNGGNGTNGTCNTNNNNNNNNGCNGGNNGTGTGCACGACCCCCNTCANCCCGACGCTGCNNCNNNNC
GNNANNNNNTCGTNNTGNNNNCNN

MADS221

>pTOPO_MADS221_546_colony_4-M13F_E07.ab1

NNNNNNNNNNNGGGCGATTGGGCCCTCTAGATGCATGCTCGAGCGGCCGCCAGTGTGATGGATATCTGCAGAATTCGCC
CTTATGGCCAATGAAAACAAATCCTTGTCAATCGACTCTCCCCAGAGAAAATTGGGTAGGGGAAAGATCGAGATCAAGCGG
ATCGAAAACACGACCAATCGTCAAGTGACCTTCTGCAAGAGGCGCAATGGGTTGCTCAAGAAGGCCTATGAACTCTCTGTG
CTCTGTGATGCAGAGGTTGCTCTCATAGTCTTCTCTAACCGTGGCCGCCTCTATGAGTATGCCAACAACAGAAGGGCGAA
TTCCAGCACACTGGCGGCCGTTACTAGTGGATCCGAGCTCGGTACCAAGCTTGATGCATAGCTTGAGTATTCTATAGTGT
CACCTAAATAGCTTGGCGTAATCATGGTCATAGCTGTTTCCTGTGTGAAATTGTTATCCGCTCACAATTCCACACAACAT
ACGAGCCGGAAGCATAAAGTGTAAAGCCTGGGGTGCCTAATGAGTGAGCTAACTCACATTAATTGCGTTGCGCTCACTGC
CCGCTTTCCAGTCGGGAAACCTGTCGTGCCAGCTGCATTAATGAATCGGCCAACGCGCGGGGAGAGGCGGTTTGCGTATT
GGGCGCTCTTCCGCTTCCTCGCTCACTGACTCGCTGCGCTCGGTCGTTCGGCTGCGGCGAGCGGTATCAGCTCACTCAAA
GGCGGTAATACGGTTATCCACAGAATCNGGGANANGCNNNNGAACATGTGAGCAAAANNCAGCAAANNCNGANNTAAA
ANNNCGTTTNCTGGNNTTTTTCATAGNNTCGCCCCCCNGANNAGCNNCACAAAAATCGACGCTCAANNCAGANNNN

>pTOPO-546-M221-colony-2-M13F_C05.ab1
NNNNNNNNNNNNNNGGGGCGATTGANTTTAGCGGCCGCGAATTCGCCCTTATGGCCAATGAAAACAAATCCTTGTCAATC
G
ACTCTCCCCAGAGAAAATTGGGTAGGGGAAAGATCGAGATCAAGCGGATCGAAAACACGACCAATCGTCAAGTGACCTTC

TGCAAGAGGCGCAATGGGTTGCTCAAGAAGGCCTATGAACTCTCTGTGCTCTGTGATGCAGAGGTTGCTCTCATAGTCTT
CTCTAACCGTGGCCGCCTCTATGAGTATGCCAACAACAGAAGGGCGAATTCGTTTAAACCTGCAGGACTAGTCCCTTTAG
TGAGGGTTAATTCTGAGCTTGGCGTAATCATGGTCATAGCTGTTTCCTGTGTGAAATTGTTATCCGCTCACAATTCCACA
CAACATACGAGCCGGAAGCATAAAGTGTAAAGCCTGGGGTGCCTAATGAGTGAGCTAACTCACATTAATTGCGTTGCGCT
CACTGCCCGCTTTCCAGTCGGGAAACCTGTCGTGCCAGCTGCATTAATGAATCGGCCAACGCGCGGGGAGAGGCGGTTTG
CGTATTGGGCGCTCTTCCGCTTCCTCGCTCACTGACTCGCTGCGCTCGGTCGTTCGGCTGCGGCGAGCGGTATCAGCTCA
CTCAAAGGCGGTAATACGGTTATCCACAGAATCAGGGGATAACGCAGGAAAGAACATGTGAGCAAAAGGCCAGCAAAAGG
CCAGGAACCGTAAAAAGGCCGCGTTGCTGGCGTTTTTCCATAGGCTCCGCCCCCCTGACGAGCATCACAAAAATCGACGC
TCAAGTCAGAGGTGGCGAAACCCGACAGGACTATAAAGATACCAGGCGTTTCCCCCTGGAAGCTCCCTCGTGCGCTCTCC
TGTTCCGANCCTGCCGCTTACCGGATACCTGTCCGCCTTTCTCCCTTCGGGAAGCGTGGCGCTTTCTCATAGCTCACGCT
GTAGGNATCTCAGTTCGGTGTANGN

Event 550

<u>MADS15</u>

>pTOPO_MADS515_550_colony_10-M13F_G10.ab1

NNNNNNNNNNGGGCGATTGGGCCCTCTAGATGCATGCTCGAGCGGCCGCCAGTGTGATGGATATCTGCAGAATTCGCC
CTTATGGCCTATGAAAGCAAATCCTTGTCCTTGGACTCTCCCCAGAGAAAATTGGGTAGGGGAAAGATCGAGATTAGAAGG
CCTATGAACTCTCTGTGCTCTGTGATGCAGAGGTTGCTCTCATAGTCTTCTCTAACCGTGGCCGCCTCTATGAGTATGCCAA
CAATAGAAGGGCGAATTCCAGCACACTGGCGGCCGTTACTAGTGGATCCGAGCTCGGTACCAAGCTTGATGCATAGCTTG
AGTATTCTATAGTGTCACCTAAATAGCTTGGCGTAATCATGGTCATAGCTGTTTCCTGTGTGAAATTGTTATCCGCTCACAAT
TCCACACAACATACGAGCCGGAAGCATAAAGTGTAAAGCCTGGGGTGCCTAATGAGTGAGCTAACTCACATTAATTGCGTT
GCGCTCACTGCCCGCTTTCCAGTCGGGAAACCTGTCGTGCCAGCTGCATTAATGAATCGGCCAACGCGCGGGGAGAGGCG
GTTTGCGTATTGGGCGCTCTTCCGCTTCCTCGCTCACTGACTCGCTGCGCTCGGTCGTTCGGCTGCGGCGAGCGGTATCAG
CTCACTCAAAGGCGGTAATACGGTTATCCACAGAATCANGGGATAACGCANGAAAGAACATGTGAGCAAAANNNCAGCAA
AANGCCAGGAACCGTAAAANNNCGCGTTTGCTGGCGTTTTTCCATAGGCTCCGCCCCCCNGACNAGCATCANAAAANCG
ACGCTCANTCANAGNNNGAAACCCGACNGNCTATNANGNNNNCNGNNNTTTNNNN

>pTOPO-MADS15-550-colony-15-M13F_E09.ab1

NNNNNNNNNNNNNNNNNNNNGATTGGGCCCTCTAGATGCATGCTCGAGCGGCCGCCAGTGTGATGGATATCTGCAGAA
TTCGCCCTTATGGCCTATGAAAGCAAATCCTTGTCCTTGGACTCTCCCCAGAGAAAATTGGGTAGGGGAAAGATCGAGATT
AGAAGGCCTATGAACTCTCTGTGCTCTGTGATGCAGAGGTTGCTCTCATAGTCTTCTCTAACCGTGGCCGCCTCTATGAGTA
TGCCAACAATAGAAGGGCGAATTCCAGCACACTGGCGGCCGTTACTAGTGGATCCGAGCTCGGTACCAAGCTTGATGCAT
AGCTTGAGTATTCTATAGTGTCACCTAAATAGCTTGGCGTAATCATGGTCATAGCTGTTTCCTGTGTGAAATTGTTATCCGCT
CACAATTCCACACAACATACGAGCCGGAAGCATAAAGTGTAAAGCCTGGGGTGCCTAATGAGTGAGCTAACTCACATTAAT
TGCGTTGCGCTCACTGCCCGCTTTCCAGTCGGGAAACCTGTCGTGCCAGCTGCATTAATGAATCGGCCAACGCGCGGGGA
GAGGCGGTTTGCGTATTGGGCGCTCTTCCGCTTCCTCGCTCACTGACTCGCTGCGCTCGGTCGTTCGGCTGCGGCGAGCG
GTATCAGCTCACTCAAAGGCGGTAATACGGTTATCCACAGAATCAGGGGATAACGCAGGAAAGAACATGTGAGCAAAAGG
CCAGCAAAAGGCCAGGAACCGTAAAAAGGCCGCGTTGCTGGCGTTTTTCCATAGGCTCCGCCCCCCTGACGAGCATCACA
AAAATCGACGCTCAAGTCAGANGTGGCGAAACCCGACAGGACTATAAAGATACCAGGCGTTTCCCCCTGGAAGCTCCCTC
GTGCGCTCTCCTGTTCCGACCCTGCCGCTTACCGGATACCTGTCCGCCTTTCTCCCTTCGGGAAGCGTGGCGCTTTCTCATA
GCTCACGCTGTAGGTATCTCAGTTCGGTGTNNTCGTTCGCTCCAAGCTGGGCTGTGTGCACGAACCCCCCGTTCAGCCCGA
CNGCTGCGCCTTATCCGGNAACTATCGTCTTGAGTCCAACCCNGNANNAANNGACTNTCNCNCTNNNNCANCNCTNNN
NNNGNTTANCNNANCGANGNANNNNGGNGGNGCTACNNNNNNNN

<u>MADS221</u>

>pTOPO_MADS221_550_colony_9-M13F_C05.ab1

NNNNNNNNNNNNNGNNNCGATTGGGCCCTCTAGATGCATGCTCGAGCGGCCGCCAGTGTGATGGATATCTGCAGAATT
CGCCCTTATGGCCAATGAAAACAAATCCTTGTCAATCGACTCTCCCCAGAGAAAATTGGGTAGGGGAAAGATCGAGATCAA
GAAGGCCTATGAACTCTCTGTGCTCTGTGATGCANAGGTTGCTCTCATAGTCTTCTCTAACCGTGGCCGCCTCTATGAGTAT
GCCAACAACAGAAGGGCGAATTCCAGCACACTGGCGGCCGTTACTAGTGGATCCGAGCTCGGTACCAAGCTTGATGCATA
ACTTGAGTATTCTATAGTGTCACCTAAATAGCTTGGCGTAATCATGGTCATANCTGTTTCCTGTGTGAAATTGTTATCCGCTC
ACAATTCCACACNACATACGAGCCGGAAGCATAAANTGTAAAGCCTGGGGTGCCTAATGAGTGAGCTAACTCNNANNAAT
TGCNTTGCNCTCACTGNCCGCTTTCCANTCGGGAAACCNGTCCGNNCNACCTCCNTTAATNAANCNNCCCAACCCCNGG
GNANAGNCNGTTTNNCGNNNTGGGNNNNNNNNNNTTCNNNNNAANNACCNNNNCCNNNNNNNTTNNNTNNNNAN
GGNNTNNNNNCNNNNNGGGNNNAANCGNTTNNCCNNNNNNNNNGGGNAANCCNNNNNNANANNNTNNNNNNNN
NNNNNNANNNNNNNNNNNNNNAANNGGNNNNNNNNNNNNNTTTTNNNNNNNNNNNCNCNCNNNNNNNNNNNN
NNNNNNNNNNNNNNNNNGNGNGNNNNNCNNNNNNNNNNNNNNANNNNNNNNNNNNNCNNNNNNNNNNNNNN
NNNNGTNTNNNNNNNNNNNNNNNNNNNCNNNNNCNCNNNNNCNNNNNNNNNNNNNNNNNTNNNNNNNNNNNNN
NNNNNNNNNNNNNNNNNNNNCNNNNNNNNNNNNNNNNNNNNNNNNNCNNNNNNNNNNTNNNNNNNNTNNNNN
NNNNNNNNNNNNNNNN

>pTOPO_MADS221_550_colony_11-M13F_E05.ab1

NNNNNNNNNNNNGGCGATTGGGCCNTCTAGATGCATGCTCGAGCGGCCGCCAGTGTGATGGATATCTGCAGAATTCGC
CCTTATGGCCAATGAAAACAAATCCTTGTCAATCGACTCTCCCCAGAGAAAATTGGGTAGGGGAAAGATCGAGATCAAGCG
GATCGAAAACACGACCAATCGTCAAGTGACCTTCTGCAAGAGGCGCAATGGGTTGCTAGAAGGCCTATGAACTCTCTGTGC
TCTGTGATGCAGAGGTTGCTCTCATAGTCTTCTCTAACCGTGGCCGCCTCTATGAGTATGCCAACAACAGAAGGGCGAATT
CCAGCACACTGGCGGCCGTTACTAGTGGATCCGAGCTCGGTACCAAGCTTGATGCATAGCTTGAGTATTCTATAGTGTCAC
CTAAATAGCTTGGCGTAATCATGGTCATAGCTGTTTCCTGTGTGAAATTGTTATCCGCTCACAATTCCACACAACATACGAGC
CGGAAGCATAAAGTGTAAAGCCTGGGGTGCCTAATGAGTGAGCTAACTCACATTAATTGCGTTGCGCTCACTGCCCGCTTT
CCAGTCGGGAAACCTGTCGTGCCAGCTGCATTAATGAATCGGCCAACGCGCGGGGAGAGGCGGTTTGCGTATTGGGCGC
TCTTCCGCTTCCTCGCTCACTGACTCGCTGCGCTCGGTCGTTCGGCTGCGGCGAGCGGTATCAGCTCACTCAAAGGCGGTA
ATACGGTTATCCACAGAATCAGGGGATAACGCANGAAAGAACATGTGAGCAAAAGGCCAGCAAAAGGNCAGGAACCGTA
AAAAGGCCGCGTTGCTGGCGTTTTTCCATAGGCTCCGCCCCCCTGACGAGCATCACAAAAATCGACGCTCAAGTCAGAGGT
GGCGAAACCCGACAGGACTATAAAGATACCAGGCGTTTCCCCCTGGNAGCTCCCTCGTGCGCTCTCCTGTTCCGACCCTGC
CGCTTACCGGATACCTGTCCGCCTTTCTCCCTNNNGNAAGCGNNGNGCTTTCTCANNAGCN

Event 552

<u>MADS15</u>

>pTOPO_MADS515_552_colony_3-M13F_A10.ab1

NNNNNNNNNNGGGCGANTGGGCCCTCTAGATGCATGCTCGAGCGGCCGCCAGTGTGATGGATATCTGCAGAATTCGCC
CTTATGGCCTATGAAAGCAAATCCTTGTCCTTGGACTCTCCCCAGAGAAAATTGGGTAGGGGAAAGATCGAGATAAGAAGG
CCTATGAACTCTCTGTGCTCTGTGATGCAGAGGTTGCTCTCATAGTCTTCTCTAACCGTGGCCGCCTCTATGAGTATGCCA
ACAATAGAAGGGCGAATTCCAGCACACTGGCGGCCGTTACTAGTGGATCCGAGCTCGGTACCAAGCTTGATGCATAGCTT

GAGTATTCTATAGTGTCACCTAAATAGCTTGGCGTAATCATGGTCATAGCTGTTTCCTGTGTGAAATTGTTATCCGCTCA
CAATTCCACACAACATACGAGCCGGAAGCATAAAGTGTAAAGCCTGGGGTGCCTAATGAGTGAGCTAACTCACATTAATT
GCGTTGCGCTCACTGCCCGCTTTCCAGTCGGGAAACCTGTCGTGCCAGCTGCATTAATGAATCGGCCAACGCGCGGGGAG
AGGCGGTTTGCGTATTGGGCGCTCTTCCGCTTCCTCGCTCACTGACTCGCTGCGCTCGGTCGTTCGGCTGCGGCGAGCGG
TATCAGCTCACTCAAAGGCGGTAATACGGTTATCCACAGAATCAGGGGATAACGCANGAAAGAACATGTGAGCAAAAGCC
AGCAAAANCCAGGAACCGTAAAAANNCNCGTTTGCTGGCGTTTTTCCATNNNTNCGCCCCCCNGACGAGCATCACAAAAN
TCGACGCTCAAGTCANANGNNGCGAAACCCCGACNNGGACTNNNN

>pTOPO-552-MADS15-colony2-M13F_G02.ab1

NNNNNNNNNNNNNGGGNNNNTGGGCCCTCTAGATGCATGCTCGAGCGGCCGCCAGTGTGATGGATATCTGCAGAATTC
GCCCTTCTATTGTTGGCATACTCATAGAGGCGGCCACGGTTAGAGAAGACTATGAGAGCAACCTCTGCATCACAGAGCACA
GAGAGTTCATAGGCCTTCTTATCTCGATCTTTCCCCTACCCAATTTTCTCTGGGGAGAGTCCAAGGACAAGGATTTGCTTTC
ATAGGCCATAAGGGCGAATTCCAGCACACTGGCGGCCGTTACTAGTGGATCCGAGCTCGGTACCAAGCTTGATGCATAGC
TTGAGTATTCTATAGTGTCACCTAAATAGCTTGGCGTAATCATGGTCATAGCTGTTTCCTGTGTGAAATTGTTATCCGCT
CACAATTCCACACAACATACGAGCCGGAAGCATAAAGTGTAAAGCCTGGGGTGCCTAATGAGTGAGCTAACTCACATTAA
TTGCGTTGCGCTCACTGCCCGCTTTCCAGTCGGGAAACCTGTCGTGCCAGCTGCATTAATGAATCGGCCAACGCGCGGGG
AGAGGCGGTTTGCGTATTGGGCGCTCTTCCGCTTCCTCGCTCACTGACTCGCTGCGCTCGGTCGTTCGGCTGCGGCGAGC
GGTATCAGCTCACTCAAAGGCGGTAATACGGTTATCCACAGAATCAGGGGATAACGCAGGAAAGAACATGTGAGCAAAAG
GCCAGCAAAAGGCCAGGAACCGTAAAAAGGCCGCGTTGCTGGCGTTTTTCCATAGGCTCCGCCCCCCTGACGAGCATCAC
AAAAATCGACGCTCAAGTCAGAGGTGGCGAAACCCGACAGGACTATAAAGATACCAGGCGTTTCCCCCTGGAAGCTCCCT
CGTGCGCTCTCCTGTTCCGACCCTGCCGCTTACCGGATACCTGTCCGCCTTTCTCCCTTCGGGAAGCGTGGCGCTTTCTC
ATAGCTCACGCTGTNNNATCTCAGTTCGNTGTNNNCGTTCGCNNCNAGCTGGGCTGNGTGCACGAACCCCCCGTTCAGC
CCGACCGCTGCGCCTTATCCNNN

MADS221

>pTOPO_MADS221_552_colony_3-M13F_B09.ab1
NNNNNNNNNNNNNNGGCGATTGGGCCCTCTAGATGCATGCTCGAGCGGCCGCCAGTGTGATGGATATCTGCAGAATTCG
CC
CTTCTGTTGTTGGCATACTCATAGAGGCGGCCACGGTTAGAGAAGACTATGAGAGCAACCTCTGCATCACAGAGCACAGA
GAGTTCATAGGCCTTCTGAGCAACCCATTGCGCCTCTTGCAGAAGGTCACTTGACGATTGGTCGTGTTTTCGATCCGCTT
GATCTCGATCTTTCCCCTACCCAATTTTCTCTGGGGAGAGTCGATTGACAAGGATTTGTTTTCATTGGCCATAAGGGCGA
ATTCCAGCACACTGGCGGCCGTTACTAGTGGATCCGAGCTCGGTACCAAGCTTGATGCATAGCTTGAGTATTCTATAGTG
TCACCTAAATAGCTTGGCGTAATCATGGTCATAGCTGTTTCCTGTGTGAAATTGTTATCCGCTCACAATTCCACACAACA
TACGAGCCGGAAGCATAAAGTGTAAAGCCTGGGGTGCCTAATGAGTGAGCTAACTCACATTAATTGCGTTGCGCTCACTG
CCCGCTTTCCAGTCGGGAAACCTGTCGTGCCAGCTGCATTAATGAATCGGCCAACGCGCGGGGAGAGGCGGTTTGCGTAT
TGGGCGCTCTTCCGCTTCCTCGCTCACTGACTCGCTGCGCTCGGTCGTTCGGCTGCGGCGAGCGGTATCAGCTCACTCAA
AGGCGGTAATACGGTTATCCACAGAATCNGGGGATAACGCANGAAAGAACATGTGAGCAAAAGGCCAGCAAAAGGCCAG
G
AACCGTAAAAAGGCCGCGTTGCTGGCGTTTTTCCATAGGCTCCGCCCCCCTGACGAGCATCACAAAAATCGACGCTCAAG
TCAGAGGTGGCGAAACCCGACAGGACTATAAAGATACCAGGCGTTTCCCCCNGGAAGCTCCCNCGTGCGNN

>pTOPO-552-MADS221-colony2-M13F_G02.ab1
NNNNNNNNNNNNNGGGNNNNTGGGCCCTCTAGATGCATGCTCGAGCGGCCGCCAGTGTGATGGATATCTGCAGAATTC
GCCCTTCTATTGTTGGCATACTCATAGAGGCGGCCACGGTTAGAGAAGACTATGAGAGCAACCTCTGCATCACAGAGCACA
GAGAGTTCATAGGCCTTCTTATCTCGATCTTTCCCCTACCCAATTTTCTCTGGGGAGAGTCCAAGGACAAGGATTTGCTTTC
ATAGGCCATAAGGGCGAATTCCAGCACACTGGCGGCCGTTACTAGTGGATCCGAGCTCGGTACCAAGCTTGATGCATAGC
TTGAGTATTCTATAGTGTCACCTAAATAGCTTGGCGTAATCATGGTCATAGCTGTTTCCTGTGTGAAATTGTTATCCGCT
CACAATTCCACACAACATACGAGCCGGAAGCATAAAGTGTAAAGCCTGGGGTGCCTAATGAGTGAGCTAACTCACATTAA
TTGCGTTGCGCTCACTGCCCGCTTTCCAGTCGGGAAACCTGTCGTGCCAGCTGCATTAATGAATCGGCCAACGCGCGGGG
AGAGGCGGTTTGCGTATTGGGCGCTCTTCCGCTTCCTCGCTCACTGACTCGCTGCGCTCGGTCGTTCGGCTGCGGCGAGC
GGTATCAGCTCACTCAAAGGCGGTAATACGGTTATCCACAGAATCAGGGGATAACGCAGGAAAGAACATGTGAGCAAAAG
GCCAGCAAAAGGCCAGGAACCGTAAAAAGGCCGCGTTGCTGGCGTTTTTCCATAGGCTCCGCCCCCCTGACGAGCATCAC
AAAAATCGACGCTCAAGTCAGAGGTGGCGAAACCCGACAGGACTATAAAGATACCAGGCGTTTCCCCCTGGAAGCTCCCT
CGTGCGCTCTCCTGTTCCGACCCTGCCGCTTACCGGATACCTGTCCGCCTTTCTCCCTTCGGGAAGCGTGGCGCTTTCTC
ATAGCTCACGCTGTNNNATCTCAGTTCGNTGTNNNCGTTCGCNNCNAGCTGGGCTGNGTGCACGAACCCCCCGTTCAGC
CCGACCGCTGCGCCTTATCCNNN

Event 553

MADS15

>pTOPO_MADS515_553_colony_12-M13F_C11.ab1

NNNNNNNNNNNGGGCGATTGGGCCCTCTAGATGCATGCTCGAGCGGCCGCCAGTGTGATGGATATCTGCAGAATTCGCC
CTTCTATTGTTGGCATACTCATAGAGGCGGCCACGGTTAGAGAAGACTATGAGAGCAACCTCTGCATCACAGAGCACAGAG
AGTTCATAGGCCTTCTTGAGCAACCCATTGCGCCTCTTGCAGAAGGTCACTTGACGATTCGTTGTGTTTTCGATCCGCTTA
ATCTCGATCTTTCCCCTACCCAATTTTCTCTGGGGAGAGTCCAAGGACAAGGATTTGCTTTCATAGGCCATAAGGGCGAA
TTCCAGCACACTGGCGGCCGTTACTAGTGGATCCGAGCTCGGTACCAAGCTTGATGCATAGCTTGAGTATTCTATAGTGT
CACCTAAATAGCTTGGCGTAATCATGGTCATAGCTGTTTCCTGTGTGAAATTGTTATCCGCTCACAATTCCACACAACAT
ACGAGCCGGAAGCATAAAGTGTAAAGCCTGGGGTGCCTAATGAGTGAGCTAACTCACATTAATTGCGTTGCGCTCACTGC
CCGCTTTCCAGTCGGGAAACCTGTCGTGCCAGCTGCATTAATGAATCGGCCAACGCGCGGGGAGAGGCGGTTTGCGTATT
GGGCGCTCTTCCGCTTCCTCGCTCACTGACTCGCTGCGCTCGGTCGTTCGGCTGCGGCGAGCGGTATCAGCTCACTCAAA
GGCGGTAATANNNTTATCCACAGAATCNGGGGANACGCNNNANANATGTGAGCAAAANNCAGCAAAAGGCCAGGAACC
GTAAAAANNCNCNNTTGCTGGCNN

>pTOPO-MADS15-553-colony-13-M13F_E11.ab1

NNNNNNNNNNNNNNGGCGATTGGGCCCTCTAGATGCATGCTCGAGCGGCCGCCAGTGTGATGGATATCTGCAGAATTCG
CC
CTTCTATTGTTGGCATACTCATAGAGGCGGCCACGGTTAGAGAAGACTATGAGAGCAACCTCTGCATCACAGAGCACAGA
GAGTTCATAGGCCTTCTTGAGCAACCCATTGCGCCTCTTGCAGAAGGTCACTTGACGATTCGTTGTGTTTTCGATCCGCT
TAATCTCGATCTTTCCCCTACCCAATTTTCTCTGGGGAGAGTCCAAGGACAAGGATTTGCTTTCATAGGCCATAAGGGCG
AATTCCAGCACACTGGCGGCCGTTACTAGTGGATCCGAGCTCGGTACCAAGCTTGATGCATAGCTTGAGTATTCTATAGT
GTCACCTAAATAGCTTGGCGTAATCATGGTCATAGCTGTTTCCTGTGTGAAATTGTTATCCGCTCACAATTCCACACAAC
ATACGAGCCGGAAGCATAAAGTGTAAAGCCTGGGGTGCCTAATGAGTGAGCTAACTCACATTAATTGCGTTGCGCTCACT
GCCCGCTTTCCAGTCGGGAAACCTGTCGTGCCAGCTGCATTAATGAATCGGCCAACGCGCGGGGAGAGGCGGTTTGCGTA
TTGGGCGCTCTTCCGCTTCCTCGCTCACTGACTCGCTGCGCTCGGTCGTTCGGCTGCGGCGAGCGGTATCAGCTCACTCA
AAGGCGGTAATACGGTTATCCACAGAATCAGGGGATAACGCAGGAAAGAACATGTGAGCAAAAGGCCAGCAAAAGGCCA

G
GAACCGTAAAAAGGCCGCGTTGCTGGCGTTTTTCCATAGGCTCCGCCCCCCTGACGAGCATCACAAAAATCGACGCTCAA
GTCAGANGTGGCGAAACCCGACAGGACTATAAAGATACCAGGCGTTTCCCCCTGGNAGCTCCCTCGTGCGCTCTCCTGTT
CCGACCCTGCCGCTTACCGGATACCTGTCCGCCTTTCTCCCTTCGGGANCGTNNGCTTTCTCATAGCTCACGCTGNNGTA
TCTCAGTTCGGNGTNGNCNTCGCTCNAGCTGGGNTGNGNGCACGAACCCCNGTTCAGCCCGACGCNNNNNN

MADS221

>pTOPO_MADS221_553_colony_6-M13F_A11.ab1

NNNNNNNNGGGCGATTGGGCCCTCTAGATGCATGCTCGAGCGGNNGCCAGTGTGATGGATATCTGCAGAATTCGCCCT
TATGGCCAATGAAAACAAATCCTTGTCAATCGACTCTCCCCAGAGAAAATTGGGTAGGGGAAAGATCGAGATCAAGCGGAT
CGAAAACACGACCAATCGTCAAGTGACCTTCTGCCAAGAGGCGCAATGGGTTGCTCAAGAAGGCCTATGAACTCTCTGTGC
TCTGTGATGCAGAGGTTGCTCTCATAGTCTTCTCTAACCGTGGCCGCCTCTATGAGTATGCCAACAACAGAAGGGCGAAT
TCCAGCACACTGGCGGCCGTTACTAGTGGATCCGAGCTCGGTACCAAGCTTGATGCATAGCTTGAGTATTCTATAGTGTC
ACCTAAATAGCTTGGCGTAATCATGGTCATAGCTGTTTCCTGTGTGAAATTGTTATCCGCTCACAATTCCACACAACATA
CGAGCCGGAAGCATAAAGTGTAAAGCCTGGGGTGCCTAATGAGTGAGCTAACTCACATTAATTGCGTTGCGCTCACTGCC
CGCTTTCCAGTCGGGAAACCTGTCGTGCCAGCTGCATTAATGAATCGGCCAACGCGCGGGGAGAGGCGGTTTGCGTATTG
GGCGCTCTTCCGCTTCCTCGCTCACTGACTCGCTGCGCTCGGTCGTTCGGCTGCGGCGAGCGGTATCAGCTCACTCAAAG
GCGGTAATANGGTTATCCACAGAATCNNGGGATAACGCNNNAAGAACATGTGAGCAAAAGGNCAGCAAAAGGCCANN

>pTOPO-MADS21-553-colony-3-M13F_G09.ab1

NNNNNNNNNNNNNNGGCGATTGGGCCCTCTAGATGCATGCTCGAGCGGCCGCCAGTGTGATGGATATCTGCAGAATTCG
CC
CTTATGGCCAATGAAAACAAATCCTTGCGCAATGGGTTGCAGAAGGCCTATGAACTCTCTGTGCTCTGTGATGCAGAGGT
TGCTCTCATAGTCTTCTCTAACCGTGGCCGCCTCTATGAGTATGCCAACAACAGAAGGGCGAATTC CAGCACACTGGCGG
CCGTTACTAGTGGATCCGAGCTCGGTACCAAGCTTGATGCATAGCTTGAGTATTCTATAGTGTCACCTAAATAGCTTGGC
GTAATCATGGTCATAGCTGTTTCCTGTGTGAAATTGTTATCCGCTCACAATTCCACACAACATACGAGCCGGAAGCATAA
AGTGTAAAGCCTGGGGTGCCTAATGAGTGAGCTAACTCACATTAATTGCGTTGCGCTCACTGCCCGCTTTCCAGTCGGGA
AACCTGTCGTGCCAGCTGCATTAATGAATCGGCCAACGCGCGGGGAGAGGCGGTTTGCGTATTGGGCGCTCTTCCGCTTC
CTCGCTCACTGACTCGCTGCGCTCGGTCGTTCGGCTGCGGCGAGCGGTATCAGCTCACTCAAAGGCGGTAATACGGTTAT
CCACAGAATCAGGGGATAACGCAGGAAAGAACATGTGAGCAAAAGGCCAGCAAAAGGCCAGGAACCGTAAAAAGGCCGC
G
TTGCTGGCGTTTTTCCATAGGCTCCGCCCCCCTGACGAGCATCACAAAAATCGACGCTCAAGTCAGAGGTGGCGAAACCC
GACAGGACTATAAAGATACCAGGCGTTTCCCCCTGGAAGCTCCCTCGTGCGCTCTCCTGTTCCGACCCTGCCGCTTACCG
GATACCTGTCCGCCTTTCTCCCTTCGGGAAGCGTGGCGCTTTCTCATAGCTCACGCTGTAGGTATCTCAGTTCGGTGTAG
GTCGTTCGCTCCAAGCTGGGCTGTGTGCACGAACCCCCCGTTCAGCCCGACCGCTGCGCCTTATCCGGTAACTATCGTCT
TGAGTCNACCCGGTAGACACGACTTATCNCNCTGNNGCANCNCTGGNANCNGNTTANCAGANCNNNGNATGNNGNNG
GGN
GCTAN

Event 554

MADS15

>pTOPO_MADS515_554_colony_1-M13F_H07.ab1

NNNNNNNNNNNNNNNGGGCGATTGGGCCCTCTAGATGCATGCTCGAGCGGCCGCCAGTGTGATGGATATCTGCAGAATT
CGCCCTTATGGCCTATGAAAGCAAATCCTTGTCCTTGGACTCTCCCCAGAGAAAATTGGGTAGGGGAAAGATCGAGATTAA
GCGGATCGAAAACACAACGAATCGTCAAGTGACCTTCTGCAAGAGGCGCAATGGGTTGCTCAAGAAGGCCTATGAACTCT
CTGTGCTCTGTGATGCAGAGGTTGCTCTCATAGTCTTCTCTAACCGTGGCCGCCTCTATGAGTATGCCAACAATAGAAGGG
CGAATTCCAGCACACTGGCGGCCGTTACTAGTGGATCCGAGCTCGGTACCAAGCTTGATGCATAGCTTGAGTATTCTATA
GTGTCACCTAAATAGCTTGGCGTAATCATGGTCATAGCTGTTTCCTGTGTGAAATTGTTATCCGCTCACAATTCCACACA
ACATACGAGCCGGAAGCATAAAGTGTAAAGCCTGGGGTGCCTAATGAGTGAGCTAACTCACATTAATTGCGTTGCGCTCA
CTGCCCGCTTTCCAGTCGGGAAACCTGTCGTGCCAGCTGCATTAATGAATCGGCCAACGCGCGGGGAGAGGCGGTTTGCG
TATTGGGCGCTCTTCCGCTTCCTCGCTCACTGACTCGCTGCGCTCGGTCGTTCGGCTGCGGCGAGCGGTATCAGCTCACT
CAAAGGCGGTAATACGGTTATCCACAGAATCAGGGGATAACGCNNAAAGAACATGTGAGCAAAAGGCCAGCAAAAGGCC
AGGAACCGTAAAAAGGCCGCGTTGCTGGCGTTTTTCCATAGGCTCCGCCCCCCTGACGAGCATCACAAAAATCGACGCTCA
AGTCANAGGTGGNNAAACCCGACNN

>pTOPO_MADS515_554_colony_2-M13F_E03.ab1

NNNNNNNNNNNNNNNNGGGCGATTGGGCCCTCTAGATGCATGCTCGAGCGGCCGCCAGTGTGATGGATATCTGCAGAA
TTCGCCCTTATGGCCTATGAAAGCAAATCCTTGTCCTTGGACTCTCCCCAGAGAAAATTGGGTAGGGGAAAGATCGAGATT
AAGCGGATCGAAAACACAACGAATCGTCAAGTGACCTTCTGCAAGAGGCGCAATGGGTTGCTCAAGAAGGCCTATGAACT
CTCTGTGCTCTGTGATGCAGAGGTTGCTCTCATAGTCTTCTCTAACCGTGGCCGCCTCTATGAGTATGCCAACAATAGAAGG
GCGAATTCCAGCACACTGGCGGCCGTTACTAGTGGATCCGAGCTCGGTACCAAGCTTGATGCATAGCTTGAGTATTCTAT
AGTGTCACCTAAATAGCTTGGCGTAATCATGGTCATAGCTGTTTCCTGTGTGAAATTGTTATCCGCTCACAATTCCACAC
AACATACGAGCCGGAAGCATAAAGTGTAAAGCCTGGGGTGCCTAATGAGTGAGCTAACTCACATTAATTGCGTTGCGCTC
ACTGCCCGCTTTCCAGTCGGGAAACCTGTCGTGCCAGCTGCATTAATGAATCGGCCAACGCGCGGGGAGAGGCGGTTTGC
GTATTGGGCGCTCTTCCGCTTCCTCGCTCACTGACTCGCTGCGCTCGGTCGTTCGGCTGCGGCGAGCGGTATCAGCTCAC
TCAAAGGCGGTAATACGGTTATCCACAGAATCAGGGGATAACGCANGAAAGAACATGTGAGCAAAAGGCCAGCAAAAGG
CCAGGAACCGTAAAAAGGCCGCGTTGGCTGGCGTTNNTTCCATAGGCTCCGCCCCCCTGACGAGCATCACAAAAATCGAC
GCTCAAGTCANAGGTGGCGAAACCCGACAGGACTATAAAGATACCAGGCGTTTCCCCCNGGAAGCTCCCTCGTGCGCTCT
CCTGTTCCGACCTGCGCTTACCGNATACCTGTCCGCCTTTNTCCNNGGNNCGTGNNCTTTCTCNTAGCTCNNNCTGTAGN
ATCTCANTCGNGTNGTCGTNGNNNCNGCTGGNNGNNTNNNNNACCCCCNNTCANCNNANNNCTGNNNNNNNCNNA
CNANCGTNNTGNNNCNANCNNAANNANNNNNNNTNNCGCCCACNN

MADS221

>pTOPO_MADS221_554_colony_1-M13F_C08.ab1

NNNNNNNNNNNNGGGGCNATTGGGCCCTCTAGATGCATGCTCGAGCGGCCGCCAGTGTGATGGATATCTGCAGAATTCG
CCCTTATGGCCAATGAAAACAAATCCTTGTCAATCGACTCTCCCCAGAGAAAAAGAAGGCCTATGAACTCTCTGTGCTCTGT
GATGCAGAGGTTGCTCTCATAGTCTTCTCTAACCGTGGCCGCCTCTATGAGTATGCCAACAACAGAAGGGCGAATTCCAG
CACACTGGCGGCCGTTACTAGTGGATCCGAGCTCGGTACCAAGCTTGATGCATAGCTTGAGTATTCTATAGTGTCACCTA
AATAGCTTGGCGTAATCATGGTCATAGCTGTTTCCTGTGTGAAATTGTTATCCGCTCACAATTCCACACAACATACGAGC
CGGAAGCATAAAGTGTAAAGCCTGGGGTGCCTAATGAGTGAGCTAACTCACATTAATTGCGTTGCGCTCACTGCCCGCTT
TCCAGTCGGGAAACCTGTCGTGCCAGCTGCATTAATGAATCGGCCAACGCGCGGGGAGAGGCGGTTTGCGTATTGGGCG

CTCTTCCGCTTCCTCGCTCACTGACTCGCTGCGCTCGGTCGTTCGGCTGCGGCGAGCGGTATCAGCTCACTCAAAGGCGGT
AATACGGTTATCCACAGAATCAGGGGATAACGCAGGAAAGAACATGTGAGCAAAAGGCCAGCAAAAGGCCAGGAACCGTA
AAAAGGCCGCGTTGCTGGCGTTTTTCCATAGGCTCCGCCCCCCTGACGAGCATCACAAAAATCGACGCTCAAGTCAGAGG
TGGCGAAACCCGACAGGACTATAAAGATACCAGGCGTTTCCCCCTGGAAGCTCCCTCGTGCGCTCTCCTGTTCCGACCCT
GCCGCTTACCGGATACCTGTCCGCCTTTCTCCTTCGGGAAGCGNNNNCTTTNTCATAGCTCACGCTGNNGGTATCNNN

>pTOPO_MADS221_554_colony_4-M13F_D08.ab1

NNNNNNNNNNNNNGNNCGANTGGGCCCTCTAGATGCATGCTCGAGCGGCCGCCAGTGTGATGGATATCTGCAGAATTC
GCCCTTATGGCCAATGAAAACAAATCCTTGTCAATCGACTCTCCCCAGAGAAAATTGGGTAGGGGAAAGATCGAGATCAAG
CGGATCGAAAACACGACCAATCGTCAAGTGACCTTCTGCAAGAGGCGCAATGGGTTGCTCAAGAAGGCCTATGAACTCTCT
GTGCTCTGTGATGCAGAGGTTGCTCTCATAGTCTTCTCTAACCGTGGCCGCCTCTATGAGTATGCCAACAACAGAAGGGCG
AATTCCAGCACACTGGCGGCCGTTACTAGTGGATCCGAGCTCGGTACCAAGCTTGATGCATAGCTTGAGTATTCTATAGT
GTCACCTAAATAGCTTGGCGTAATCATGGTCATAGCTGTTTCCTGTGTGAAATTGTTATCCGCTCACAATTCCACACAAC
ATACGAGCCGGAAGCATAAAGTGTAAAGCCTGGGGTGCCTAATGAGTGAGCTAACTCACATTAATTGCGTTGCGCTCACT
GCCCGCTTTCCAGTCGGGAAACCTGTCGTGCCAGCTGCATTAATGAATCGGCCAACGCGCGGGGAGAGGCGGTTTGCGTA
TTGGGCGCTCTTCCGCTTCCTCGCTCACTGACTCGCTGCGCTCGGTCGTTCGGCTGCGGCGAGCGGTATCAGCTCACTCA
AAGGCGGTAATACGGTTATCCACAGAATCAGGGGATAACGCANGAAAGAACATGTGAGCAAAAGGCCAGCAAAAGGCCA
GGAACCGTAAAAAGGCCGCGTTGCTGGCGTTTTTCCATAGNCTCCGCCCCCCTGACGAGCATCACAAAAATCGACGCTCAA
GTCAGAGGTGGCGAAACCCGACAGGACTATAAAGATACCANNNN

Event 7152

<u>MADS15</u>

>pTOPO-7152-MADS15-colony-3-M13F

NNNNNNNNNNNGGGCGATTGGGCCCTCTAGATGCATGCTCGAGCGGCCGCCAGTGTGATGGATATCTGCAGAATT
CGCCCTTTTTCATTTGTTTCTGCAAGTTTCAAACTTTTTGTTGGTGATCTAGAATATAGTAATTAATTAAAGTTCTGGGG
TTTTTATTTTAATTAGAAAGGTGCTTAATTGATTGATCTCTTTGTTCTTATTGATTCAGCTTCCAACTATGGCCTATGAAA
GCAAATCCTTGTCCTTGGACTCTCCCCAGAGAAAATTGGGTAGGGGAAAGATCGAGATTTAAGCGGATCGAAAACA
CAACGAATCGTCAAGTGACCTTCTGCAAGAGGCGCAATGGGTTGCTCAAGAAGGCCTATGAACTCTCTGTGCTCTGT
GATGCAGAGGTTGCTCTCATAGTCTTCTCTAACCGTGGCCGCCTCTATGAGTATGCCAACAATAGGTAATATTATTGCTT
CAATTTACTTGAAGGGCGAATTCCAGCACACTGGCGGCCGTTACTAGTGGATCCGAGCTCGGTACCAAGCTTGATGC
ATAGCTTGAGTATTCTATAGTGTCACCTAAATAGCTTGGCGTAATCATGGTCATAGCTGTTTCCTGTGTGAAATTGTTAT
CCGCTCACAATTCCACACAACATACGAGCCGGAAGCATAAAGTGTAAAGCCTGGGGTGCCTAATGAGTGAGCTAACT
CACATTAATTGCGTTGCGCTCACTGCCCGCTTTCCAGTCGGGAAACCTGTCGTGCCAGCTGCATTAATGAATCGGCCA
ACGCGCGGGGAGANGGCGGTTTGCGTATTGGGCGCTCTTCCGCTTCCTCGCTCACTGANTCGCTGCGCTCGGTCGT
TCGGCTGCGGCGANNNGNTATCNNCTCANTNNAGGCNGGTNNTACGGTTANCCACANAATNNNGGGANANCGC
AAGGAAANACNNTGTGAGCCAAAGGCCNNNAAAGGCCAGGNACCGGNNAAAAGGGCCNCGTTGCTGGCGTTTT
TCCAGTAGNNNN

>pTOPO_MADS515_7152_colony_6-M13F_G06.ab1

NNNNNNNNNNNNNNGGCGATTGGGCCCTCTAGATGCATGCTCGAGCGGCCGCCAGTGTGATGGATATCTGCAGAAT
TCGCCCTTATGGCCTATGAAAGCAAATCCTTGTCCTTGGACTCTCCCCAGAGAAATTGGGTAGGGGAAAGATCGAGA
TTAAGCGGATCGAAAACACAACGAATCGTCAAGTGACCTTCTGCAAGAGGCGCAATGGGTTGAGCACAGAGGTTGC
TCTCATAGTCTTCTCTAACCGTGGCCGCCTCTATGAGTATGCCAACAATAGAAGGGCGAATTCCAGCACACTGGCGGC
CGTTACTAGTGGATCCGAGCTCGGTACCAAGCTTGATGCATAGCTTGAGTATTCTATAGTGTCACCTAAATAGCTTGGC
GTAATCATGGTCATAGCTGTTTCCTGTGTGAAATTGTTATCCGCTCACAATTCCACACAACATACGAGCCGGAAGCATA
AAGTGTAAAGCCTGGGGTGCCTAATGAGTGAGCTAACTCACATTAATTGCGTTGCGCTCACTGCCCGCTTTCCAGTCG
GGAAACCTGTCGTGCCAGCTGCATTAATGAATCGGCCAACGCGCGGGGAGAGGCGGTTTGCGTATTGGGCGCTCTT
CCGCTTCCTCGCTCACTGACTCGCTGCGCTCGGTCGTTCGGCTGCGGCGAGCGGTATCAGCTCACTCAAAGGCGGTA
ATACGGTTATCCACAGAATCAGGGGATAACGCANGAAAGAACATGTGAGCAAAAGGCCAGCAAAAGGCCAGGAAC
CGTAAAAAGGCCGCGTTGCTGGCGTTTTTCCATAGGCTCCGCCCCCCTGACGAGCATCACAAAAATCGACGCTCAAG
TCAGANGTGGCGAAACCCGACAGGACTATAAAGATACCAGGCGTTTCCCCCTGGNAGCTCCCTCGTGCGCTCTCCTG
TTCCGACCCTGCCGCTTACCGGGATACCTGTCCGCCTTTCTCCCTTCGGGAAGCGNNNNNCTTTCTCNNNANCT

MADS221

>pTOPO-7152-MADS221-colony-7-M13F

NNNNNNNNNNNGGCGATTGGGCCCTCTAGATGCATGCTCGAGCGGCCGCCAGTGTGATGGATATCTGCAGAATTC
GCCCTTGTTGGTGATCAAGAATATAGTAATTGAAGTTCTGGGGTTTTTGTTTTATTTAAAAAGGTGCTTAATTGATTGA
TCTCTTTGTTCTTATTGATTCAGCTTGCAACTATGGCCAATGAAAACAAATCCTTGTCAATCGACTCTCCCCAGAGAAA
ATTGGGTAGGGGAAAGATCGAGATCAAGCGGATCGAAAACACGACCAATCGTCAAGTGACCTTCTGCAAGAGGCG
CAATGGGTTGCTCAAGAAGGCCTATGAACTCTCTGTGCTCTGTGATGCAGAGGTTGCTCTCATAGTCTTCTCTAACCG
TGGCCGCCTCTATGAGTATGCCAACAACAGGTAATAATATTATTGCTTCAATTTTCTTGCTAAGGGCGAATTCCAGCAC
ACTGGCGGCCGTTACTAGTGGATCCGAGCTCGGTACCAAGCTTGATGCATAGCTTGAGTATTCTATAGTGTCACCTAA
ATAGCTTGGCGTAATCATGGTCATAGCTGTTTCCTGTGTGAAATTGTTATCCGCTCACAATTCCACACAACATACGAGC
CGGAAGCATAAAGTGTAAAGCCTGGGGTGCCTAATGAGTGAGCTAACTCACATTAATTGCGTTGCGCTCACTGCCCG
CTTTCCAGTCGGGAAACCTGTCGTGCCAGCTGCATTAATGAATCGGCCAACGCGCGGGGAGAGGCGGTTTGCGTAT
TGGGCGCTCTTCCGCTTCCTCGCTCACTGACTCGCTGCGCTCGGTCGTTCGGCTGCGGCGAGCGGTATCAGCTCACT
CAAAGGCGGTAATN

>pTOPO_MADS221_7152_colony_1-M13F_G08.ab1

NNNNNNNNNNAGGGCGATTGGGCCCTCTAGATGCATGCTCGAGCGGCCGCCAGTGTGATGGATATCTGCAGAATT
CGCCCTTATGGCCAATGAAAACAAATCCTTGTCAATCGACTCTCCCCAGAGAAAATTGGGTAGGGGAAAGATCGAGA
TCAAGAAGGCCTATGAACTCTCTGTGCTCTGTGATGCAGAGGTTGCTCTCATAGTCTTCTCTAACCGTGGCCGCCTCTA
TGAGTATGCCAACAACAGAAGGGCGAATTCCAGCACACTGGCGGCCGTTACTAGTGGATCCGAGCTCGGTACCAAG
CTTGATGCATAGCTTGAGTATTCTATAGTGTCACCTAAATAGCTTGGCGTAATCATGGTCATAGCTGTTTCCTGTGTGAA
ATTGTTATCCGCTCACAATTCCACACAACATACGAGCCGGAAGCATAAAGTGTAAAGCCTGGGGTGCCTAATGAGTGA
GCTAACTCACATTAATTGCGTTGCGCTCACTGCCCGCTTTCCAGTCGGGAAACCTGTCGTGCCAGCTGCATTAATGAA
TCGGCCAACGCGCGGGGAGAGGCGGTTTGCGTATTGGGCGCTCTTCCGCTTCCTCGCTCACTGACTCGCTGCGCTC
GGTCGTTCGGCTGCGGCGAGCGGTATCAGCTCACTCAAAGGCGGTAATACGGTTATCCACAGAATCAGGGGATAAC
GCAGGAAAGAACATGTGAGCAAAAGGCCAGCAAAAGGCCAGGAACCGTAAAAAGGCCGCGTTGCTGGCGTTTTTC

CATAGGCTCCGCCCCCCTGACGAGCATCACAAAAATCGACGCTCAAGTCAGAGGTGGCGAAACCCGACAGGACTAT
AAAGATACCAGGCGTTTCCCCCTGGAAGCTCCCTCGTGCGCTCTCCTGTTCCNACCCTGN

Event 7241

MADS15

>7241-MADS15-5-M13F_E02.ab1
NNNNNNNNNNNNNNNNNGGNNNNNGATTTAGCGGCCGCGAATTCGCCCTTCTATTGTTGGCATACTCATAGAGGC
GGCCACGGTTAGAGAAGACTATGAGAGCAACCTCTGCATCACAGAGCACAGAGAGTTCATAGGCCTTCTCTGGGGA
GAGTCGATTGACAAGGATTTGCTTTCATAGGCCATAAGGGCGAATTCGTTTAAACCTGCAGGACTAGTCCCTTTAGTG
AGGGTTAATTCTGAGCTTGGCGTAATCATGGTCATAGCTGTTTCCTGTGTGAAATTGTTATCCGCTCACAATTCCACAC
AACATACGAGCCGGAAGCATAAAGTGTAAAGCCTGGGGTGCCTAATGAGTGAGCTAACTCACATTAATTGCGTTGCG
CTCACTGCCCGCTTTCCAGTCGGGAAACCTGTCGTGCCAGCTGCATTAATGAATCGGCCAACGCGCGGGGAGAGGC
GGTTTGCGTATTGGGCGCTCTTCCGCTTCCTCGCTCACTGACTCGCTGCGCTCGGTCGTTCGGCTGCGGCGAGCGGT
ATCAGCTCACTCAAAGGCGGTAATACGGTTATCCACAGAATCAGGGGATAACGCAGGAAAGAACATGTGAGCAAAA
GGCCAGCAAAAGGCCAGGAACCGTAAAAAGGCCGCGTTGCTGGCGTTTTTCCATAGGCTCCGCCCCCCTGACGAGC
ATCACAAAAATCGACGCTCAAGTCAGAGGTGGCGAAACCCGACAGGACTATAAAGATACCAGGCGTTTCCCCCTGG
AAGCTCCCTCGTGCGCTCTCCTGTTCCGACCCTGCCGCTTACCGGATACCTGTCCGCCTTTCTCCCTTCGGGAAGCGT
GGCGCTTTCTCATAGCTCACGCTGTANGTATCTCAGTTCGGTGTNNTCGTTCGCTCCAAGCTGGGCTGTGTGCACGA
ACCCCCCGTTCAGCCCGACCGCTGCGCCTTATCCGGTAAC

>pTOPO_MADS515_7241_colony_1-M13F_A08.ab1

NNNNNNNNNNNNNNNGGCGATTGGGCCCTCTAGATGCATGCTCGAGCGGCCGCCAGTGTGATGGATATCTGCAGAATTC
GCCCTTCAAGTAAATTGAAGCAATAATATTACCTATTGTTGGCATACTCATAGAGGCGGCCACGGTTAGAGAAGACTATGAG
AGCAACCTCTGCATCACAGAGCACAGAGAGTTCATAGGCCTTCTGCAACCCATTGCGCCTCTTGCAGAAGGTCACTTGAC
GATTCCTGAAACTTGCAGAAACAAATGAAAAAGGGCGAATTCCAGCACACTGGCGGCCGTTACTAGTGGATCCGAGCTCG
GTACCAAGCTTGATGCATAGCTTGAGTATTCTATAGTGTCACCTAAATAGCTTGGCGTAATCATGGTCATAGCTGTTTCC
TGTGTGAAATTGTTATCCGCTCACAATTCCACACAACATACGAGCCGGAAGCATAAAGTGTAAAGCCTGGGGTGCCTAAT
GAGTGAGCTAACTCACATTAATTGCGTTGCGCTCACTGCCCGCTTTCCAGTCGGGAAACCTGTCGTGCCAGCTGCATTAA
TGAATCGGCCAACGCGCGGGGAGAGGCGGTTTGCGTATTGGGCGCTCTTCCGCTTCCTCGCTCACTGACTCGCTGCGCTC
GGTCGTTCGGCTGCGGCGAGCGGTATCAGCTCACTCAAAGGCGGTAATACGGTTATCCACAGAATCAGGGGATAACGCAN
GAAAGAACATGTGAGCAAAAGGCCAGCAAAAGGCCAGGAACCGTAAAAAGGCCGCGTTGCTGGCGTTTTTCCATAGGCTC
CGCCCCCCTGACGAGCATCACAAAAATCGACGCTCAAGTCAGAGGTGGCGAAACCCGACAGGACTATAAAGATACCAGGC
GTTTCCCCCTGGAAGCTCCCTCGTGCGCTCTCCTGTTCCGACCCTGCCGCTTACCGGATACCTGTCCGCCTTTCTCCCTT
CGGGAAGCGTGCGCTTTCTCATAGCTCACGCTGTAGGNATCTCAGTTCGNTGTNNNCGTCGCTCAAGCTGGGCTGTGTGC
ACGAACCCCCGTTCANCCNACCGCTGNNNCTTATCCGGTAANNNTCGNCNNN

MADS221

>pTOPO-7241-MADS221-colony3-M13F

NNNNNNNNNGGCGATTGGGCCCTCTAGATGCATGCTCGAGCGGCCGCCAGTGTGATGGATATCTGCAGAATTCGCCC
TTAGCAAGAAAATTGAAGCAATAATATTATTACCTGTTGTTGGCATACTCATAGAGGCGGCCACGGTTAGAGAAGACT
ATGAGAGCAACCTCTGCATCACAGAGCACAGAGAGTTCATAGGCCTTCTTAGCAACCCATTGCGCCTCTTGCAGAAG
GTCACTTGACGATTGGTCGTGTTTTCGATCCGCTTGATCTCGATCTTTCCCCTACCCAATTTTCTCTGGGGAGAGTCGA
TTGACAAGGATTTGTTTTCATTGGCCATAGTTGCAAGCTGAATCAATAAGAACAAAGAGATCAATCAATTAAGCACCT
TTTTAAATAAAACAAAAACCCCAGAACTTCAATTACTATATTCTTGATCACCAACAAGGGCGAATTCCAGCACACTGG
CGGCCGTTACTAGTGGATCCGAGCTCGGTACCAAGCTTGATGCATAGCTTGAGTATTCTATAGTGTCACCTAAATAGCT
TGGCGTAATCATGGTCATAGCTGTTTCCTGTGTGAAATTGTTATCCGCTCACAATTCCACACAACATACGAGCCGGAAG
CATAAAGTGTAAAGCCTGGGGTGCCTAATGAGTGAGCTAACTCACATTAATTGCGTTGCGCTCACTGCCCGCTTTCCA
GTCGGGAAACCTGTCGTGCCAGCTGCATTAATGAATCGGCCAACGCGCGGGGAGAGGCGGTTTGCGTATTGGGCGC
TCTTCCGCTTCCTCGCTCACTGACTCGCTGCGCTCGGTCGTTCGGCTGCGGCGAGCGGTATCAGCTCACTCAAAGGC
GGTAATANGGTTATCCACAGAATCNGGGGATAACGCNNAAAGAACATGTGAGCAAAAGGNCAGNN

>7241MADS221colony3-M13F_D09.ab1
NNNNNNNNNNNNGGGCGATTGGNCCCTCTAGATGCATGCTCGAGCGGCCGCCAGTGTGATGGATATCTGCAGAAT
TCGCCCTTATGGCCAATGAAAACAAATCCTTGCGCAATGGGTTGCAGAAGGCCTATGAACTCTCTGTGCTCTGTGATG
CAGAGGTTGCTCTCATAGTCTTCTCTAACCGTGGCCGCCTCTATGAGTATGCCAACAACAGAAGGGCGAATTCCAGCA
CACTGGCGGCCGTTACTAGTGGATCCGAGCTCGGTACCAAGCTTGATGCATAGCTTGAGTATTCTATAGTGTCACCTA
AATAGCTTGGCGTAATCATGGTCATAGCTGTTTCCTGTGTGAAATTGTTATCCGCTCACAATTCCACACAACATACGAG
CCGGAAGCATAAAGTGTAAAGCCTGGGGTGCCTAATGAGTGAGCTAACTCACATTAATTGCGTTGCGCTCACTGCCC
GCTTTCCAGTCGGGAAACCTGTCGTGCCAGCTGCATTAATGAATCGGCCAACGCGCGGGGAGAGGCGGTTTGCGTA
TTGGGCGCTCTTCCGCTTCCTCGCTCACTGACTCGCTGCGCTCGGTCGTTCGGCTGCGGCGAGCGGTATCAGCTCAC
TCAAAGGCGGTAATACGGTTATCCACAGAATCAGGGGATAACGCAGGAAAGAACATGTGAGCAAAAGGCCAGCAA
AAGGCCAGGAACCGTAAAAAGGCCGCGTTGCTGGCGTTTTTCCATAGGCTCCGCCCCCCTGACGAGCATCACAAAA
ATCGACGCTCAAGTCAGANGTGGCGAAACCCGACAGGACTATAAAGATACCAGGCGTTTCCCCCTGGAAANCTCCC
TCGTGCGCTCTCCTGTTCCGACCCTGCCGCTTACCGGATACCTGTCCGCCTTTCTCCCTTCCGGGAAGCGTGGCGCTT
TCTCATAGCTCACGCTGNAGGNNNNNNNAGTTNCGGNNGTAGGTCGTTCNNNTNCNAGCCTGGGCTGNNNTGC
NNNGAACCCCCCGNTCAGCCCGACNCTGCNNNNNCNGTANTNNCGTNNNANTCNNCCCGGTANNNANNCGACT
TATCNCNNCTGGNAGCAGCCNCNGNN

Event 7243

<u>MADS15</u>

>7243-MADS15-6-M13F_B02.ab1
NNNNNNNNNNNNNNGGCGATTGAATTTAGCGGCCGCGAATTCGCCCTTATGGCCTATGAAAGCAAATCCTTGTCCTT
GGACTCTCCCCAGAGAAAATTGGGTAGGGGAAAGATCGAGATTAAGAAGGCCTATGAACTCTCTGTGCTCTGTGATG
CAGAGGTTGCTCTCATAGTCTTCTCTAACCGTGGCCGCCTCTATGAGTATGCCAACAATAGAAGGGCGAATTCGTTTA
AACCTGCAGGACTAGTCCCTTTAGTGAGGGTTAATTCTGAGCTTGGCGTAATCATGGTCATAGCTGTTTCCTGTGTGA
AATTGTTATCCGCTCACAATTCCACACAACATACGAGCCGGAAGCATAAAGTGTAAAGCCTGGGGTGCCTAATGAGTG
AGCTAACTCACATTAATTGCGTTGCGCTCACTGCCCGCTTTCCAGTCGGGAAACCTGTCGTGCCAGCTGCATTAATGA
ATCGGCCAACGCGCGGGGAGAGGCGGTTTGCGTATTGGGCGCTCTTCCGCTTCCTCGCTCACTGACTCGCTGCGCTC
GGTCGTTCGGCTGCGGCGAGCGGTATCAGCTCACTCAAAGGCGGTAATACGGTTATCCACAGAATCAGGGGATAAC
GCAGGAAAGAACATGTGAGCAAAAGGCCAGCAAAAGGCCAGGAACCGTAAAAAGGCCGCGTTGCTGGCGTTTTTC

CATAGGCTCCGCCCCCCTGACGAGCATCACAAAAATCGACGCTCAAGTCAGAGGTGGCGAAACCCGACAGGACTAT
AAAGATACCAGGCGTTTCCCCCTGGAAGCTCCCTCGTGCGCTCTCCTGTTCCGACCCTGCCGCTTACCGGATACCTGT
CCGCCTTTCTCCCTTCGGGAAGCGTGGCGCTTTCTCATAGCTCACGCTGTNNNATCTCAGTTCGGTGTNNTCGTTCG
CTCCAAGCTGGGCTGTGTGCACGAACCCCCCGTTCA
GCCCGACCGCTGCGCCTTATCCGGTAACTATCGTCTTGAGTCCANNNN

>7243-MADS15-5-M13F_A02.ab1
NNNNNNNNNNNNNNNNNATTGNTTTAGCGGCCGCGAATTCGCCCTTATGGCCTATGAAAGCAAATCCTTGTCCTTG
GACTCTCCCCAGAGAAAATTGGGTAGGGGAAAGATCGAGATTAGAAGGCCTATGAACTCTCTGTGCTCTGTGATGCA
GAGGTTGCTCTCATAGTCTTCTCTAACCGTGGCCGCCTCTATGAGTATGCCAACAATAGAAGGGCGAATTCGTTTAAA
CCTGCAGGACTAGTCCCTTTAGTGAGGGTTAATTCTGAGCTTGGCGTAATCATGGTCATAGCTGTTTCCTGTGTGAAA
TTGTTATCCGCTCACAATTCCACACAACATACGAGCCGGAAGCATAAAGTGTAAAGCCTGGGGTGCCTAATGAGTGA
GCTAACTCACATTAATTGCGTTGCGCTCACTGCCCGCTTTCCAGTCGGGAAACCTGTCGTGCCAGCTGCATTAATGAA
TCGGCCAACGCGCGGGGAGAGGCGGTTTGCGTATTGGGCGCTCTTCCGCTTCCTCGCTCACTGACTCGCTGCGCTC
GGTCGTTCGGCTGCGGCGAGCGGTATCAGCTCACTCAAAGGCGGTAATACGGTTATCCACAGAATCAGGGGATAAC
GCAGGAAAGAACATGTGAGCAAAAGGCCAGCAAAAGGCCAGGAACCGTAAAAAGGCCGCGTTGCTGGCGTTTTTC
CATAGGCTCCGCCCCCCTGACGAGCATCACAAAAATCGACGCTCAAGTCAGAGGTGGCGAAACCCGACAGGACTAT
AAAGATACCAGGCGTTTCCCCCTGGAAGCTCCCTCGTGCGCTCTCCTGTTCCGACCCTGCCGCTTACCGGATACCTGT
CCGCCTTTCTCCCTTCGGGAAGCGTGGCGCTTTCTCATAGCTCACGCTGTANGTATCTCAGTTCGGTGTNNTCGTTCG
CTCCAAGCTGGGCTGTGTGCACGAACCCCCCGTTCAGC
CCGACCGCTGCGCCTTAN

MADS221

>7243MADS221colony6-M13F_G09.ab1
NNNNNNNNNNNNGGGCGATTGNGGCCCTCTAGATGCATGCTCNGAGCGGCCGCCAGTGTGATGGATATCTGCAG
AATTCGCCCTTATGGCCAATGAAAACAAATCCTTGTCCTTGGACTCTCCCCAGAGAAAATTGGGTAGGGGAAAGATC
GAGATTAAAAAGGCCTATGAACTCTCTGGGCTCTGTGATGCAAAGGTTGCTCTCATAGTCTTCTCTAACCGGGGCCGC
CTCTATGAGTATGCCAACAACAAAAGGGCGAATTCCACCACACTGGCGGCCGTTACTAGGGGATCCAAGCTCGGTAC
CAAGCTTGATGCATAGCTTGAGTATTCTATAGGGTCACCTAAATAGCTTGGCGTAATCATGGTCATAGCTGTTTCCTGN
GTGAAATTGTTATCCGCTCACAATTCCACACAACATACAAGCCGGAAGCATAAAGGGTAAAGCCTGGGGGGCCTAAT
GAGTGAGCTAACTCACATTAATTGCGTTGCGCTCACTGCCCGCTTTCCAGTCGGGAAACCTGTCNGGCCANCTGCAT
TAATGAATCGGCCAACGCGCGGGGANAGGCGGTTTGCGNATTGGGCGCTCTTCCGCTTCCTCNCTCACTGACTCNC
TGCGCTCGGNCGTTCGGCTGCGGNNAGCGGNATCANCTCACTCAAAGGCGGTAATACGGTTATCCACANAATCAGG
GGATAACGCAGGAAANAACATGTGANCAAAAGGCCAGCAAAAGGCCAGNAACCGTAAAAAGGCCGCGTTGCTGN
CGTTTTTCCATAGGCTCCGCCCCCCTGACGAGCATCACAAAAATCGACGCTCAAGTCANAGGTGNNAAACCCGACA
GGACTATAANNATACCAGGCGTTTCCCCTNNNNGCTCCCTCGTGCGCTCTCNNTNNACCCTGCCGCTNNCGGATAC
TGTCGCNTNNNCNNGGNANNNNGCGCTTNNCNTAGNTCACGCNNNAGNNTCTCAGTNNNNTAGNCGTNNNNT
NNNCTNGNNTNNNGNNCNACCCCCNNNANNNNACGCTGNNCNNTCGNNACNATCN

>7243MADS221colony8-M13F_H09.ab1
NNNNNNNNNNTAGGGCGATTGGNCCCTCTAGATGCATGCTCGAGCGGCCGCCAGTGTGATGGATATCTGCAGAAT
TCGCCCTTATGGCCAATGAAAACAAATCCTTGTCAATCGACTCTCCCCAGAGAAAATTGGGTAGGGGAAAGATCGAG

ATCAAGCGGATCGAAAACACGACCAATCGTCAAGTGACCTTCTGCAAGAGGCGCAATGGGTTGCAGAAGGCCTATG
AACTCTCTGTGCTCTGTGATGCAGAGGTTGCTCTCATAGTCTTCTCTAACCGTGGCCGCCTCTATGAGTATGCCAACAA
CAGAAGGGCGAATTCCAGCACACTGGCGGCCGTTACTAGTGGATCCGAGCTCGGTACCAAGCTTGATGCATAGCTTG
AGTATTCTATAGTGTCACCTAAATAGCTTGGCGTAATCATGGTCATAGCTGTTTCCTGTGTGAAATTGTTATCCGCTCAC
AATTCCACACAACATACGAGCCGGAAGCATAAAGTGTAAAGCCTGGGGTGCCTAATGAGTGAGCTAACTCACATTAA
TTGCGTTGCGCTCACTGCCCGCTTTCCAGTCGGGAAACCTGTCGTGCCAGCTGCATTAATGAATCGGCCAACGCGCG
GGGAGAGGCGGTTTGCGTATTGGGCGCTCTTCCGCTTCCTCGCTCACTGACTCGCTGCGCTCGGTCGTTCGGCTGCG
GCGAGCGGTATCAGCTCACTCAAAGGCGGTAATACGGTTATCCACAGAATCAGGGGATAACGCAGGAAAGAACATG
TGAGCAAAAGGCCAGCAAAAGGCCAGGAACCGTAAAAAGGCCGCGTTGCTGGCGTTTTTCCATAGGCTCCGCCCCC
CTGACGAGCATCACAAAAATCGACGCTCAAGTCAGAGGTGGCGAAACCCGACAGGACTATAAAGATACCAGGCGTT
TCCCCCTGGAAGCTCCCTCGTGCGCTCTNCTGTTCCGACCCTGCCGCTTACCGGATACCTGTCCGCCTTTCTCCCNNN
NGGAAGCNNNGNGCTTTCTCATAGCTCACNNTGNNNNANNNTCAGTTNNGGNNNN

Event 7187

<u>MADS15</u>

>7187-MADS15-5-M13F_G02.ab1
NNNNNNNNNNNNNNANGGCGATTGATTTAGCGGCCGCGAATTCGCCCTTATGGCCTATGAAAGCAAATCCTTGTCC
TTGGACTCTCCCCAGAGAAAATTGGGTAGGGGAAAGATCGAGATTAAGCGGATCGAAAACACAACGAATCGTCAAG
TGACCTTCTGCAAGAGGCGCAATGGGTTGCTCAAGAAGGCCTATGAACTCTCTGTGCTCTGTGATGCAGAGGTTGCT
CTCATAGTCTTCTCTAACCGTGGCCGCCTCTATGAGTATGCCAACAATAGAAGGGCGAATTCGTTTAAACCTGCAGGA
CTAGTCCCTTTAGTGAGGGTTAATTCTGAGCTTGGCGTAATCATGGTCATAGCTGTTTCCTGTGTGAAATTGTTATCCG
CTCACAATTCCACACAACATACGAGCCGGAAGCATAAAGTGTAAAGCCTGGGGTGCCTAATGAGTGAGCTAACTCAC
ATTAATTGCGTTGCGCTCACTGCCCGCTTTCCAGTCGGGAAACCTGTCGTGCCAGCTGCATTAATGAATCGGCCAACG
CGCGGGGAGAGGCGGTTTGCGTATTGGGCGCTCTTCCGCTTCCTCGCTCACTGACTCGCTGCGCTCGGTCGTTCGGC
TGCGGCGAGCGGTATCAGCTCACTCAAAGGCGGTAATACGGTTATCCACAGAATCAGGGGATAACGCAGGAAAGAA
CATGTGAGCAAAAGGCCAGCAAAAGGCCAGGAACCGTAAAAAGGCCGCGTTGCTGGCGTTTTTCCATAGGCTCCGC
CCCCCTGACGAGCATCACAAAAATCGACGCTCAAGTCAGANGTGGCGAAACCCGACAGGACTATAAAGATACCAGG
CGTTTCCCCCTGGAAGCTCCCTCGTGCGCTCTCCTGTTCCGACCCTGCCGCTTACCGGATACCTGTCCGCCTTTCTCCC
TTCGGGAAGCGTGGCGCTTTCTCATAGCTCACGCTGTAGGTATCTCAGTTC

>7187-MADS15-6-M13F_H02.ab1
NNNNNNNNNNNNNNNNGGCGATTGATTTAGCGGCCGCGAATTCGCCCTTCTATTGTTGGCATACTCATAGAGGCGG
CCACGGTTAGAGAAGACTATGAGAGCAACCTCTGCATCACAGAGCACAGAGAGTTCATAGGCCTTCTTGAGCAACC
CATTGCGCCTCTTGCAGAAGGTCACTTGACGATTCGTTGTGTTTTCGATCCGCTTAATCTCGATCTTTCCCCTACCCAAT
TTTCTCTGGGGAGAGTCCAAGGACAAGGATTTGCTTTCATAGGCCATAAGGGCGAATTCGTTTAAACCTGCAGGACT
AGTCCCTTTAGTGAGGGTTAATTCTGAGCTTGGCGTAATCATGGTCATAGCTGTTTCCTGTGTGAAATTGTTATCCGCT
CACAATTCCACACAACATACGAGCCGGAAGCATAAAGTGTAAAGCCTGGGGTGCCTAATGAGTGAGCTAACTCACAT
TAATTGCGTTGCGCTCACTGCCCGCTTTCCAGTCGGGAAACCTGTCGTGCCAGCTGCATTAATGAATCGGCCAACGCG
CGGGGAGAGGCGGTTTGCGTATTGGGCGCTCTTCCGCTTCCTCGCTCACTGACTCGCTGCGCTCGGTCGTTCGGCTG
CGGCGAGCGGTATCAGCTCACTCAAAGGCGGTAATACGGTTATCCACAGAATCAGGGGATAACGCAGGAAAGAACA
TGTGAGCAAAAGGCCAGCAAAAGGCCAGGAACCGTAAAAAGGCCGCGTTGCTGGCGTTTTTCCATAGGCTCCGCC
CCCCTGACGAGCATCACAAAAATCGACGCTCAAGTCAGANGTGGCGAAACCCGACAGGACTATAAAGATACCAGGC
GTTTCCCCCTGGAAAGCTCCCTCGTGCGCTCTCCTGTTCCGACCCTGCCGCTTACCGGATACCTGTCCGCCTTTCTCCC
TTCNGGAAGCGTG

MADS221

>7187MADS221colony4-M13F_E09.ab1
NNNNNNNNNNNNTNGGGCGATTGGGCCCTCTAGATGCATGCTCGAGCGGCCGCCAGTGTGATGGATATCTGCAGAA
TTCGCCCTTATGGCCAATGAAAACAAATCCTTGTCAATCGACTCTCCCCAGAGAAAATTGGGTAGGGGAAAGATCGA
GATCAAGCGGATCGAAAACACGACCAATCGTCAAGTGACCTTCTGCAAGAGGCGCAATGGGTTGCTCAAGAAGGCC
TATGAACTCTCTGTGCTCTGTGATGCAGAGGTTGCTCTCATAGTCTTCTCTAACCGTGGCCGCCTCTATGAGTATGCCA
ACAACAGAAGGGCGAATTCCAGCACACTGGCGGCCGTTACTAGTGGATCCGAGCTCGGTACCAAGCTTGATGCATA
GCTTGAGTATTCTATAGTGTCACCTAAATAGCTTGGCGTAATCATGGTCATAGCTGTTTCCTGTGTGAAATTGTTATCCG
CTCACAATTCCACACAACATACGAGCCGGAAGCATAAAGTGTAAAGCCTGGGGTGCCTAATGAGTGAGCTAACTCAC
ATTAATTGCGTTGCGCTCACTGCCCGCTTTCCAGTCGGGAAACCTGTCGTGCCAGCTGCATTAATGAATCGGCCAACG
CGCGGGGAGAGGCGGTTTGCGTATTGGGCGCTCTTCCGCTTCCTCGCTCACTGACTCGCTGCGCTCGGTCGTTCGGC
TGCGGCGAGCGGTATCAGCTCACTCAAAGGCGGTAATACGGTTATCCACAGAATCAGGGGATAACGCAGGAAAGAA
CATGTGAGCAAAAGGCCAGCAAAAGGCCAGGAACCGTAAAAAGGNCGCGTTGCTGGCGTTTTTCCATNNCTCCGC
CCCCCTGACGAGCATCACAAAAATCGACGCTCAAGTCANANGTGGCGAAACCCGACAGGACTATAANATACCAGGC
GTTTCCCCCTNGNNGCTCCCTCGTGCGCTCTCCTGTNNACCTGCCGCTACCGGATACNGTCGCNTTCTCCNNNGGA
NCGTGNGCTTTCTCATAGCTNNNNCTGNAGNNNNTCANTNGNGTAGNCNNNGCTCNAGCTGGNNTNNNNGCAN
NN

>7187MADS221colony6-M13F_F09.ab1
NNNNNNNNNNNNNNGGGCGATTGGGCCCTCTAGATGCATGCTCGAGCGGCCGCCAGTGTGATGGATATCTGCAGA
ATTCGCCCTTATGGCCAATGAAAACAAATCCTTGTCAATCGACTCTCCCCAGAGAAAATTGGGTAGGGGAAAGATCG
AGATCAAGCGGATCGAAAACACGACCAATCGTCAAGTGACCTTCTGCAAGAGGCGCAATGGGTTGCTCAAGAAGGC
CTATGAACTCTCTGTGCTCTGTGATGCAGAGGTTGCTCTCATAGTCTTCTCTAACCGTGGCCGCCTCTATGAGTATGCC
AACAACAGAAGGGCGAATTCCAGCACACTGGCGGCCGTTACTAGTGGATCCGAGCTCGGTACCAAGCTTGATGCAT
AGCTTGAGTATTCTATAGTGTCACCTAAATAGCTTGGCGTAATCATGGTCATAGCTGTTTCCTGTGTGAAATTGTTATCC
GCTCACAATTCCACACAACATACGAGCCGGAAGCATAAAGTGTAAAGCCTGGGGTGCCTAATGAGTGAGCTAACTCA
CATTAATTGCGTTGCGCTCACTGCCCGCTTTCCAGTCGGGAAACCTGTCGTGCCAGCTGCATTAATGAATCGGCCAAC
GCGCGGGGAGAGGCGGTTTGCGTATTGGGCGCTCTTCCGCTTCCTCGCTCACTGACTCGCTGCGCTCGGTCGTTCG
GCTGCGGCGAGCGGTATCAGCTCACTCAAAGGCGGTAATACGGTTATCCACAGAATCAGGGGATAACGCAGGAAAG
AACATGTGAGCAAAAGGCCAGCAAAAGGCCAGGAACCGTAAAAAGGNCGCGTTGCTGGCGTTTTTCCATAGGCTC
CGCCCCCCTGACGAGCATCACAAAAATCGACGCTCAAGTCAGAGGTGGCGAAACCCGACAGGACTATAAAGATACC
AGGCGTTTCCCCCTGGAAGCTCCCTCGTGCGCTCTCCTGTNCNACCCTGCCGCTTACCNGATACCTGTCCNCTTTCTC
CCNNNNNNNNGNGNNCTTTCTCATAGCTCNNNCTNNNGGNANNNCNNNNTNNNGGNGNAGGTCNNNNCNN
NCNANNCNNGGGNTGNNNNNCANNAAN

Event 7190

MADS15

>pTOPO_MADS515_7190_colony_2-M13F_B11.ab1

NNNNNNNNNNNGGGCGATTGGGCCCTCTAGATGCATGCTCGAGCGGCCGCCAGTGTGATGGATATCTGCAGAATTC
GCCCTTATGGCCTATGAAAGCAAATCCTTGTCCTTGGACTCTCCCCAGAGAAAATTGGGTAGGGGAAAGATCGAGAT
TAAGCGGATCGAAAACACAACGAATCGTCAAGTGACCTTCTGCAAGAGGCGCAATGGGTTGCTCAAGAAGGCCTAT
GAACTCTCTGTGCTCTGTGATGCAGAGGTTGCTCTCATAGTCTTCTCTAACCGTGGCCGCCTCTATGAGTATGCCAACA

ATAGAAGGGCGAATTCCAGCACACTGGCGGCCGTTACTAGTGGATCCGAGCTCGGTACCAAGCTTGATGCATAGCTT
GAGTATTCTATAGTGTCACCTAAATAGCTTGGCGTAATCATGGTCATAGCTGTTTCCTGTGTGAAATTGTTATCCGCTCA
CAATTCCACACAACATACGAGCCGGAAGCATAAAGTGTAAAGCCTGGGGTGCCTAATGAGTGAGCTAACTCACATTA
ATTGCGTTGCGCTCACTGCCCGCTTTCCAGTCGGGAAACCTGTCGTGCCAGCTGCATTAATGAATCGGCCAACGCGC
GGGGAGAGGCGGTTTGCGTATTGGGCGCTCTTCCGCTTCCTCGCTCACTGACTCGCTGCGCTCGGTCGTTCGGCTGCG
GCGAGCGGTATCAGCTCACTCAAAGGCGGTAATANGGTTATCCACAGAATCNGGGATAACGCNNNAGAACATGTGAGCA
AAAGGCCAGCAAAANGCCANGAACCGTAAAAAGGCCNCGTTGCTGGCN

>pTOPO-MADS15-7190-colony-1-M13F_G10.ab1

NNNNNNNNNNNNGGGCGATTGGGCCCTCTAGATGCATGCTCGAGCGGCCGCCAGTGTGATGGATATCTGCAGAATTCG
CCCTTATGGCCTATGAAAGCAAATCCTTGTCCTTGGACTCTCCCCAGAGAAAATTGGGTAGGGGAAAGATCGAGATTAAGC
GGATCGAAAACACAACGAATCGTCAAGTGACCTTCTGCAAGAGGCGCAATGGGTTGCTCAAGAAGGCCTATGAACTCTCTG
TGCTCTGTGATGCAGAGGTTGCTCTCATAGTCTTCTCTAACCGTGGCCGCCTCTATGAGTATGCCAACAATAGAAGGGCGA
ATTCCAGCACACTGGCGGCCGTTACTAGTGGATCCGAGCTCGGTACCAAGCTTGATGCATAGCTTGAGTATTCTATAGTGT
CACCTAAATAGCTTGGCGTAATCATGGTCATAGCTGTTTCCTGTGTGAAATTGTTATCCGCTCACAATTCCACACAACATACG
AGCCGGAAGCATAAAGTGTAAAGCCTGGGGTGCCTAATGAGTGAGCTAACTCACATTAATTGCGTTGCGCTCACTGCCCGC
TTTCCAGTCGGGAAACCTGTCGTGCCAGCTGCATTAATGAATCGGCCAACGCGCGGGGAGAGGCGGTTTGCGTATTGGGC
GCTCTTCCGCTTCCTCGCTCACTGACTCGCTGCGCTCGGTCGTTCGGCTGCGGCGAGCGGTATCAGCTCACTCAAAGGCGG
TAATACGGTTATCCACAGAATCAGGGGATAACGCAGGAAAGAACATGTGAGCAAAAGGCCAGCAAAAGGCCAGGAACCGT
AAAAAGGNCGCGTTTGCTGGCGTTTTTCCATAGGCTCCGCCCCCCTGACGAGCATCACAAAAATCGACGCTCAAGTCAGAN
GTGGCGAAACCCGACAGGACTATAAAGATACCAGGCGTTTCCCCCTGGAAGCTCCCTCGTGCGCTCTCCTGTTCCGACCCT
GCCGCTTACCGGATACCTGTCCGCCTTTCNNCCTTCGGGAAGCGTGNGCTTTCTCATAGCTCACGCTGTNNNATCTCANN
NGGNGTAGNCNTNGCTCNAGCTGGGCTGNNNGCACGANCCCCGTTCANCCCGACGCTNCGCCTTATCCNGNAACNNN

<u>MADS221</u>

>pTOPO_MADS221_7190_colony_2-M13F_D11.ab1

NNNNNNNNNNGGGNGANTNNTTTAGCGGCCGCGAATTCGCCCTTATGGCCAATGAAAACAAATCCTTGTCAATCGACTC
TCCCCAGAGAAAATTGGGTAGGGGAAAGATCGAGAAGCGGATCGAAAACACGACCAATCGTCAAGTGACCTTCTGCAAGA
AGGCCTATGAACTCTCTGTGCTCTGTGATGCAGAGGTTGCTCTCATAGTCTTCTCTAACCGTGGCCGCCTCTATGAGTATGC
CAACAACAGAAGGGCGAATTCGTTTAAACCTGCAGGACTAGTCCCTTTAGTGAGGGTTAATTCTGAGCTTGGCGTAATCAT
GGTCATAGCTGTTTCCTGTGTGAAATTGTTATCCGCTCACAATTCCACACAACATACGAGCCGGAAGCATAAAGTGTAAAGC
CTGGGGTGCCTAATGAGTGAGCTAACTCACATTAATTGCGTTGCGCTCACTGCCCGCTTTCCAGTCGGGAAACCTGTCGTG
CCAGCTGCATTAATGAATCGGCCAACGCGCGGGGAGAGGCGGTTTGCGTATTGGGCGCTCTTCCGCTTCCTCGCTCACTG
ACTCGCTGCGCTCGGTCGTTCGGCTGCGGCGAGCGGTATCAGCTCACTCAAAGGCGGTAATACGGTTATCCACAGAATCA
NGGGATAACGCANGAAAGAACATGTGAGCAAAAGGCCAGCAAAAGGCCAGGAACCGTAAAAAGGCCGCGTTTGCTGGCG
TTTTTCCATNGGCTCCGCCCCCCTGACGAGCATCACAAAAATCGACGCTCAANTCAGANNGGCGAAACCCGACAGNACTA
TAAAGATACCAGGCGTTN

>pTOPO_MADS221_7190_colony_3-M13F_D06.ab1

NNNNNNNNNNNNNGGCGATTGGGCCCTCTAGATGCATGCTCGAGCGGCCGCCAGTGTGATGGATATCTGCAGAATTCGC
CCTTATGGCCAATGAAAACAAATCCTTGTCAATCGACTCTCCCCAGAGAAAATTGGGTAGGGGAAAGATCGAGATCAAGCG
GATCGAAAACACGACCAATCGTCAAGTGACCTTCTGCAAGAGGCGCAATGGGTTGCTCAAGAAGGCCTATGAACTCTCTGT

GCTCTGTGATGCAGAGGTTGCTCTCATAGTCTTCTCTAACCGTGGCCGCCTCTATGAGTATGCCAACAACAGAAGGGCGAA
TTCCAGCACACTGGCGGCCGTTACTAGTGGATCCGAGCTCGGTACCAAGCTTGATGCATAGCTTGAGTATTCTATAGTGTC
ACCTAAATAGCTTGGCGTAATCATGGTCATAGCTGTTTCCTGTGTGAAATTGTTATCCGCTCACAATTCCACACAACATACGA
GCCGGAAGCATAAAGTGTAAAGCCTGGGGTGCCTAATGAGTGAGCTAACTCACATTAATTGCGTTGCGCTCACTGCCCGCT
TTCCAGTCGGGAAACCTGTCGTGCCAGCTGCATTAATGAATCGGCCAACGCGCGGGGAGAGGCGGTTTGCGTATTGGGCG
CTCTTCCGCTTCCTCGCTCACTGACTCGCTGCGCTCGGTCGTTCGGCTGCGGCGAGCGGTATCAGCTCACTCAAAGGCGGT
AATACGGTTATCCACAGAATCANGGGATAACGCANGAAAGAACATGTGAGCAAAAGGNCAGCAAAAGGNCAGGAACCGT
AAAAAGGCCGCGTTGCTGGCGTTTTTCCATAGGCTCCGCCCCCCTGACGAGCATCACAAAAATCGACGCTCAAGTCA
GAGGTGGCGAAACCCGACAGGACTATAAAGATACCAGGCGTTTCCCCCNGGAAGCTCCNCGTGCGCTCNCTGNNC
NACCTGCNNNTNNNNATNCNGTCGCNTNTCCNNNNANCNNGNNCTTTCNCATANCTCNNGCTGTNGNANCNCA
NTCNNNTNNNNCNNTNNNNNNNNNNCNGNNNGNNNNNNNCNANCCCCGTTCANCCNGNNN

Event 7193

<u>MADS15</u>

>pTOPO-MADS15-548-7193-colony-4-M13F_B09.ab1
NNNNNNNNNNNNNGGGCGATTGATTTAGCGGCCGCGAATTCGCCCTTCTGTTGTTGGCATACTCATAGAGGCGGCC
ACGGTTAGAGAAGACTATGAGAGCAACCTCTGCATCACAGAGCACAGAGAGTTCATAGGCCTTCTTGAGCAACCCAT
TGCGCCTCTTTTCTCTGGGGAGAGTCGATTGACAAGGATTTGTTTTCATTGGCCATAAGGGCGAATTCGTTTAAACCT
GCAGGACTAGTCCCTTTAGTGAGGGTTAATTCTGAGCTTGGCGTAATCATGGTCATAGCTGTTTCCTGTGTGAAATTG
TTATCCGCTCACAATTCCACACAACATACGAGCCGGAAGCATAAAGTGTAAAGCCTGGGGTGCCTAATGAGTGAGCT
AACTCACATTAATTGCGTTGCGCTCACTGCCCGCTTTCCAGTCGGGAAACCTGTCGTGCCAGCTGCATTAATGAATCG
GCCAACGCGCGGGGAGAGGCGGTTTGCGTATTGGGCGCTCTTCCGCTTCCTCGCTCACTGACTCGCTGCGCTCGGT
CGTTCGGCTGCGGCGAGCGGTATCAGCTCACTCAAAGGCGGTAATACGGTTATCCACAGAATCAGGGGATAACGCA
GGAAAGAACATGTGAGCAAAAGGCCAGCAAAAGGCCAGGAACCGTAAAAAGGCCGCGTTGCTGGCGTTTTTCCAT
AGGCTCCGCCCCCCTGACGAGCATCACAAAAATCGACGCTCAAGTCAGAGGTGGCGAAACCCGACAGGACTATAAA
GATACCAGGCGTTTCCCCCTGGAAGCTCCCTCGTGCGCTCTCCTGTTCCGACCCTGCCGCTTACCGGATACCTGTCCG
CCTTTCTCCCTTCGGGAAGCGTGGCGCTTTCTCATAGCTCACGCTGTAGGTATCTCAGTTCGGTGTAGGTCGTTCGCT
CCAAGCTGGGCTGTGTGCACGAACCCCCCGTTCAGCCCGACCGCTGCGCCTTATCCGGTAACTATCGTCTTGAGTCCA
ACCCGGTANANACGACTTATCGCNACTGGCAGCAGCCACTGGNANCAGGATTAGCANANCNANGNATGTAGGNG
GNGCTACNGANTTCNTGANTGGNNGCCTANTACGGCTACNCTANAANAANNGTNTTTGGN

>pTOPO_MADS515_548-7193_colony_1-M13F_C08.ab1
NNNNNNNNNNNNNNNNNGNNGGGCNTCTAGATGCATGCTCGAGCGGCCGCCAGTGTGATGGATATCTGCAGAATT
CGCCCTTTTTCATTTGTTTCTGCAAGTTTCAAACTTTTTGTTGGTGATCTAGAATATAGTAATTAATTAAAGTTCTGGGG
TTTTTATTTTAATTAGAAAGGTGCTTAATTGATTGATCTCTTTGTTCTTATTGATTCAGCTTCCAACTATGGCCTATGAAA
GCAAATCCTTGTCCTTGGACTCTCCCCAGAGAAAATTGGGTAGGGGAAAGATCGAGATTAAGCGGATCGAAAACAC
AACGAATCGTCAAGTGACCTTCTGCAAGAGGCGCAATGGGTTGCTCAAGAAGGCCTATGAACTCTCTGTGCTCTGTG
ATGCAGAGGTTGCTCTCATAGTCTTCTCTAACCGTGGCCGCCTCTATGAGTATGCCAACAATAGGTAATATTATTGCTTC
AATTTACTTGAAGGGCGAATTCCAGCACACTGGCGGCCGTTACTAGTGGATCCGAGCTCGGTACCAAGCTTGATGCA
TAGCTTGAGTATTCTATAGTGTCACCTAAATAGCTTGGCGTAATCATGGTCATAGCTGTTTCCTGTGTGAAATTGTTATC
CGCTCACAATTCCACACAACATACGAGCCGGAAGCATAAAGTGTAAAGCCTGGGGTGCCTAATGAGTGAGCTAACTC
ACATTAATTGCGTTGCGCTCACTGCCCGCTTTCCAGTCGGGAAACCTGTCGTGCCAGCTGCATTAATGAATCGGCCAA

CGCGCGGGGAGAGGCGGTTTGCGTATTGGGCGCTCTTCCGCTTCCTCGCTCACTGACTCGCTGCGCTCGGTCGTTCG
GCTGNNCGAGCGGTATCAGCTCACTCAAAGGCGGTATACGGTTATCACAGAATCNGGGATNACGCAGAAAGAACAT
GTGAGCAAAGGCAGCAAAAGGCCAGGAACCGTAAAAGGCCGCGTTNCTGGCGTTTTCCATNNGCNCCGCCCCCCN
N

MADS221

>pTOPO_MADS221_548_colony_4-M13F_E08.ab1

NNNNNNNNNGGGCGATTGGGCCCTCTAGATGCATGCTCGAGCGGCCGCCAGTGTGATGGATATCTGCAGAATTCG
CCCTTATGGCCAATGAAAACAAATCCTTGTCAATCGACTCTCCCCAGAGAAAATTGGGTAGGGGAAAGATCGAGATC
AAGAAGGCCTATGAACTCTCTGTGCTCTGTGATGCAGAGGTTGCTCTCATAGTCTTCTCTAACCGTGGCCGCCTCTATG
AGTATGCCAACAACAGAAGGGCGAATTCCAGCACACTGGCGGCCGTTACTAGTGGATCCGAGCTCGGTACCAAGCT
TGATGCATAGCTTGAGTATTCTATAGTGTCACCTAAATAGCTTGGCGTAATCATGGTCATAGCTGTTTCCTGTGTGAAAT
TGTTATCCGCTCACAATTCCACACAACATACGAGCCGGAAGCATAAAGTGTAAAGCCTGGGGTGCCTAATGAGTGAG
CTAACTCACATTAATTGCGTTGCGCTCACTGCCCGCTTTCCAGTCGGGAAACCTGTCGTGCCAGCTGCATTAATGAATC
GGCCAACGCGCGGGGAGAGGCGGTTTGCGTATTGGGCGCTCTTCCGCTTCCTCGCTCACTGACTCGCTGCGCTCGG
TCGTTCGGCTGCGGCGAGCGGTATCAGCTCACTCAAAGGCGGTAATACGGTTATCCACAGAATCNGGGGATAACGCA
CGAAAGAACATGTGAGCAAAANNCCAGCAAAANGCCAGGAACCGTAAAANNNCNCGTTTGCTGGCGTTTTTNCAT
AGGCTCCGCCCCCCNGACGAGCATCANAAAATCGACGCTNANTCANAGNNNGAAACCCGACNGNCTATNAAGATA
CCAGNNNTTTCCCCCNNNNNNCNNCCNNNCNNNNNNNN

>pTOPO_MADS221_548_colony_7-M13F_F08.ab1

NNNNNNNNNGGGCGATTGGGCCCTCTAGATGCATGCTCGAGCGGCCGCCAGTGTGATGGATATCTGCAGAATTCG
CCCTTATGGCCAATGAAAACAAATCCTTGTCAATCGACTCTCCCCAGAGAAAATTGGGTAGGGGAAAGATCGAGATC
AGCGGATCGAAAACACGACCAATCGTCAAGTGACCTTCTGCAAGAGGCGCAACCTATGAACTCTCTGTGCTCTGTGA
TGCAGAGGTTGCTCTCATAGTCTTCTCTAACCGTGGCCGCCTCTATGAGTATGCCAACAACAGAAGGGCGAATTCCAG
CACACTGGCGGCCGTTACTAGTGGATCCGAGCTCGGTACCAAGCTTGATGCATAGCTTGAGTATTCTATAGTGTCACC
TAAATAGCTTGGCGTAATCATGGTCATAGCTGTTTCCTGTGTGAAATTGTTATCCGCTCACAATTCCACACAACATACGA
GCCGGAAGCATAAAGTGTAAAGCCTGGGGTGCCTAATGAGTGAGCTAACTCACATTAATTGCGTTGCGCTCACTGCC
CGCTTTCCAGTCGGGAAACCTGTCGTGCCAGCTGCATTAATGAATCGGCCAACGCGCGGGGAGAGGCGGTTTGCGT
ATTGGGCGCTCTTCCGCTTCCTCGCTCACTGACTCGCTGCGCTCGGTCGTTCGGCTGCGGCGAGCGGTATCAGCTCA
CTCAAAGGCGGTAATACGGTTATCCACAGAATCNNGGGATAACGCNNNAAAGAACATGTGAGCAAAANNCAGCAA
AAGGCCAGGAACCGTAAAAANNNCNNNGCTGGNGTTTTCNTNNNTCNCCCCCCNGACNAGCATCACAAAAATCG
ACGCTCAANNTCANNNNGNTNGGCGAAANCCNGANNNNN

Event 7259

MADS15

>7259-MADS15-6-M13F_C02.ab1
NNNNNNNNNNNNNNNGGCGATTGNTTTAGCGGCCGCGAATTCGCCCTTCTATTGTTGGCATACTCATAGAGGCGGC
CACGGTTAGAGAAGACTATGAGAGCAACCTCTGCATCACAGAGCACAGAGAGTTCATAGGCCTTCTCTGGGGAGAG
TCGATTGACAAGGATTTGCTTTCATAGGCCATAAGGGCGAATTCGTTTAAACCTGCAGGACTAGTCCCTTTAGTGAGG
GTTAATTCTGAGCTTGGCGTAATCATGGTCATAGCTGTTTCCTGTGTGAAATTGTTATCCGCTCACAATTCCACACAAC
ATACGAGCCGGAAGCATAAAGTGTAAAGCCTGGGGTGCCTAATGAGTGAGCTAACTCACATTAATTGCGTTGCGCTC
ACTGCCCGCTTTCCAGTCGGGAAACCTGTCGTGCCAGCTGCATTAATGAATCGGCCAACGCGCGGGGAGAGGCGGT
TTGCGTATTGGGCGCTCTTCCGCTTCCTCGCTCACTGACTCGCTGCGCTCGGTCGTTCGGCTGCGGCGAGCGGTATCA
GCTCACTCAAAGGCGGTAATACGGTTATCCACAGAATCAGGGGATAACGCAGGAAAGAACATGTGAGCAAAAGGCC
AGCAAAAGGCCAGGAACCGTAAAAAGGCCGCGTTGCTGGCGTTTTTCCATAGGCTCCGCCCCCCTGACGAGCATCA
CAAAAATCGACGCTCAAGTCAGAGGTGGCGAAACCCGACAGGACTATAAAGATACCAGGCGTTTCCCCCTGGAAGC
TCCCTCGTGCGCTCTCCTGTTCCGACCCTGCCGCTTACCGGATACCTGTCCGCCTTTCTCCCTTCGGGAAGCGTGGCG
CTTTCTCATAGCTCACGCTGTANGTATCTCAGTTCGGTGTAGGTCGTTCGCTCCAAGCTGGGCTGTGTGCACGAACCC
CCCGTTCAGCCCGACCGCTGCGCCTTATCCGGTAACTATCGTCTTGAGTCCAACCCGGTAAGACACGACTTATCGCCA
CTGGCAGCAGCCACTG

>7259-MADS15-7-M13F_D02.ab1
NNNNNNNNNNNNAGGGCGATTGNTTTAGCGGCCGCGAATTCGCCCTTCTATTGTTGGCATACTCATAGAGGCGGCC
ACGGTTAGAGAAGACTATGAGAGCAACCTCTGCATCACAGAGCACAGAGAGTTCATAGGCCTTCTTGAGCAACCCAT
TGCGCCTCTTGCAGAAGGTCACTTGACGATTCGTTGTGTTTTCGATCCGCTTAATCTCGATCTTTCCCCTACCCAATTTT
CTCTGGGGAGAGTCCAAGGACAAGGATTTGCTTTCATAGGCCATAAGGGCGAATTCGTTTAAACCTGCAGGACTAGT
CCCTTTAGTGAGGGTTAATTCTGAGCTTGGCGTAATCATGGTCATAGCTGTTTCCTGTGTGAAATTGTTATCCGCTCAC
AATTCCACACAACATACGAGCCGGAAGCATAAAGTGTAAAGCCTGGGGTGCCTAATGAGTGAGCTAACTCACATTAA
TTGCGTTGCGCTCACTGCCCGCTTTCCAGTCGGGAAACCTGTCGTGCCAGCTGCATTAATGAATCGGCCAACGCGCG
GGGAGAGGCGGTTTGCGTATTGGGCGCTCTTCCGCTTCCTCGCTCACTGACTCGCTGCGCTCGGTCGTTCGGCTGCG
GCGAGCGGTATCAGCTCACTCAAAGGCGGTAATACGGTTATCCACAGAATCAGGGGATAACGCAGGAAAGAACATG
TGAGCAAAAGGCCAGCAAAAGGCCAGGAACCGTAAAAAGGCCGCGTTGCTGGCGTTTTTCCATAGGCTCCGCCCCC
CTGACGAGCATCACAAAAATCGACGCTCAAGTCAGAGGTGGCGAAACCCGACAGGACTATAAAGATACCAGGCGTT
TCCCCCTGGAAGCTCCCTCGTGCGCTCTCCTGTTCCGACCCTGCCGCTTACCGGATACCTGTCCGCCTTTCTCCCTTCG
GGAAGCGTGGCGCTTTCTCATAGCTCACGCTGTNNTATCTCAGTTCGGTGTAGGNCGTTCGCTCCAAGCTGGGCTGT
GTGCACGAANNN

<u>MADS221</u>

>pTOPO-7259-MAD221-colony-6-M13F

NNNNNNNNNNNNNNNGGGCGATTGGGCCCTCTAGATGCATGCTCGAGCGGCCGCCAGTGTGATGGATATCTGCAG
AATTCGCCCTTATGGCCAATGAAAACAAATCCTTGTCAATCGACTCTCCCCAGAGAAAATTGGGTAGGGGAAAGATC
GAGATCAAGCGGATCGAAAACACGACCAATCGTCAAGTGACCTTCTGCAAGAGGCGCAATGGGTTGCTCAAGAAG
GCCTATGAACTCTCTGTGCTCTGTGATGCAGAGGTTGCTCTCATAGTCTTCTCTAACCGTGGCCGCCTCTATGAGTATG
CCAACAACAGAAGGGCGAATTCCAGCACACTGGCGGCCGTTACTAGTGGATCCGAGCTCGGTACCAAGCTTGATGC
ATAGCTTGAGTATTCTATAGTGTCACCTAAATAGCTTGGCGTAATCATGGTCATAGCTGTTTCCTGTGTGAAATTGTTAT
CCGCTCACAATTCCACACAACATACGAGCCGGAAGCATAAAGTGTAAAGCCTGGGGTGCCTAATGAGTGAGCTAACT
CACATTAATTGCGTTGCGCTCACTGCCCGCTTTCCAGTCGGGAAACCTGTCGTGCCAGCTGCATTAATGAATCGGCCA
ACGCGCGGGGAGAGGCGGTTTGCGTATTGGGCGCTCTTCCGCTTCCTCGCTCACTGACTCGCTGCGCTCGGTCGTTC

GGCTGCGGCGAGCGGTATCAGCTCACTCAAAGGCGGTAATACGGTTATCCACAGAATCAGGGGATAACGCANGAAA
GAACATGTGAGCAAAAGGCCAGCAAAAGGCCAGGAACCGTAAAAAGGCCGCGTTGCTGGCGTTTTTCCATNNCTC
CGCCCCCCTGACGAGCATCACAAAAATCGACGCTCAAGTCANAGGTGGCGAAACCCGACAGGACTATAAAGATACC
AGGCGTTTCCCCCTGGAAGCTCCCTCGTGCGCTCTCCTGTTCCNANCCTGCCGCTTACCGGATNNN

>7159MADS221colony3-M13F_C06.ab1
NNNNNNNNNNNNNNNGGNNNNTGGGCCCTCTAGATGCATGCTCGAGCGGCCGCCAGTGTGATGGATATCTGCAGA
ATTCGCCCTTATGGCCAATGAAAACAAATCCTTGTCAATCGACTCTCCCCAGAGAAAATTGGGTAGGGGAAAGATCG
AGATCAAGCGGATCGAAAACACGACCAATCGTCAAGTGACCTTCTGCAAGAGGCGCAATGGGTTGCTCAAGAAGGC
CTATGAACTCTCTGTGCTCTGTGATGCAGAGGTTGCTCTCATAGTCTTCTCTAACCGTGGCCGCCTCTATGAGTATGCC
AACAACAGAAGGGCGAATTCCAGCACACTGGCGGCCGTTACTAGTGGATCCGAGCTCGGTACCAAGCTTGATGCAT
AGCTTGAGTATTCTATAGTGTCACCTAAATAGCTTGGCGTAATCATGGTCATAGCTGTTTCCTGTGTGAAATTGTTATCC
GCTCACAATTCCACACAACATACGAGCCGGAAGCATAAAGTGTAAAGCCTGGGGTGCCTAATGAGTGAGCTAACTCA
CATTAATTGCGTTGCGCTCACTGCCCGCTTTCCAGTCGGGAAACCTGTCGTGCCAGCTGCATTAATGAATCGGCCAAC
GCGCGGGGAGAGGCGGTTTGCGTATTGGGCGCTCTTCCGCTTCCTCGCTCACTGACTCGCTGCGCTCGGTCGTTCG
GCTGCGGCGAGCGGTATCAGCTCACTCAAAGGCGGTAATACGGTTATCCACAGAATCAGGGGATAACGCAGGAAAG
AACATGTGAGCAAAANGCCAGCAAAAGGCCAGGAACCGTAAAAAAGGCCGCGTTGCTGGCGTTTTTCCATNNNCT
CCGCCCCCCTGACNANCNTCACAAAAATCGACGCTCAAGTCAGAGGTGGCGAAACCCGANAGGACTATNNAAATN
CANGCTNTNTCCCCTGGAAGCTCCCTCGTGCGCTCTCCTGNTCCNACCNGCCGCTTANCNGATACCTGNCCGCTTTN
TCCCTTCGGNAGCGNGNGCTTTCNCNNNCTCAGCTGNAGGNANNNNNNTCNGNGTAGGTCNNNNNNNNAANN
NNGNNTGTNTNANNNANCNCCNNNNNNNNNNN
