# Supplementary material for: Apple CRISPR-Cas9—A Recipe for Successful Targeting of AGAMOUS-like Genes in Domestic Apple
Source: Plants (Basel). 2023 Oct 26;12(21):3693. doi: 10.3390/plants12213693 (PMC10649517; doi:10.3390/plants12213693)
Supplement: Supplementary file 1 [file plants-12-03693-s001.zip › supplemental file 2 predicted peptides v1.pdf]

Predicted peptides from selected events

MADS15

>M26_MADS15

MAYESKSLSLDSPQRKLGRGKIEIKRIENTTNRQVTFCKRRNGLLKKAYELSVLCDAEVALIVFSNRGRLYEYANN

>Cas9_MADS15

MAYESKSLSLDSPQRKLGRGKIEIKRIENTTNRQVTFCKRRNGLLKKAYELSVLCDAEVALIVFSNRGRLYEYANN

>525_MADS15

MAYESKSLSLDSPQRKLGRGKIEIKRIENTTNRQVTFCKRRNGLQKAYELSVLCDAEVALIVFSNRGRLYEYANN

>529_MADS15

MAYESKSLSLDSPQRKLGRGKIEIKRIENTTNRQVTFCKRRNGLLYELSVLCDAEVALIVFSNRGRLYEYANN

>523_MADS15

MAYESKSLSLDSPQRKLGRGKIEIKRIENTTNRQVTFCKRRNGLLEGL*

>524_MADS15

MAYESKSLSLDSPQRKLGRGKIEIKRIENTTNRQVTFCKRRNG*

MADS221

>M26_MADS221

MANENKSLSIDSPQRKLGRGKIEIKRIENTTNRQVTFCKRRNGLLKKAYELSVLCDAEVALIVFSNRGRLYEYANN

>Cas9_MADS221

MANENKSLSIDSPQRKLGRGKIEIKRIENTTNRQVTFCKRRNGLLKKAYELSVLCDAEVALIVFSNRGRLYEYANN

>515_MADS221

MANENKSLSIDSPQRKLGRGKIEIKRIENTTNRQVTFCKRRNGLLKKAYELSVLCDAEVALIVFSNRGRLYEYANN

>7243_MADS221

MANENKSLSIDSPQRKLGRGKIEIKRIENTTNRQVTFCKRRNGLQKAYELSVLCDAEVALIVFSNRGRLYEYANN

>516_MADS221

MANENKSLSIDSPQRKLGRGKIEIKRIENTTNRQVTFCKRRNGLLRPVNSLCSVMXRLLSYSSLTVAASMNMP

>553_MADS221

MANENKSLSIDSPQRKLGRGKIEIKRIENTTNRQVTFCQEAQWVAQEGL*

>522_MADS221

MANENKSLSIDSPQRKLGRGKIEIKRIENTTNRQVTFCKRRNGLLEGL*
